# Genome-wide identification and expression analysis of the cyclic nucleotide-gated ion channel (CNGC) gene family in *Saccharum spontaneum*

**DOI:** 10.1186/s12864-023-09307-3

**Published:** 2023-05-25

**Authors:** Nannan Zhang, Huanzhang Lin, Qiaoying Zeng, Danwen Fu, Xiaoning Gao, Jiayun Wu, Xiaomin Feng, Qinnan Wang, Qiuping Ling, Zilin Wu

**Affiliations:** 1 Guangdong Sugarcane Genetic Improvement Engineering Center, Institute of Nanfan & Seed Industry, Guangdong Academy of Sciences, Guangzhou, 510316 China; 2 Zhongkai University of Agriculture and Engineering, Guangzhou, 510225 China; 3 Rice Research Institute, Guangdong Academy of Agricultural Sciences, Guangzhou, 510640 China

**Keywords:** CNGC, *Saccharum spontaneum*, Development, Circadian rhythm, Low-K^+^ stress

## Abstract

**Background:**

Cyclic nucleotide-gated ion channels (CNGCs) are nonselective cation channels that are ubiquitous in eukaryotic organisms. As Ca^2+^ channels, some CNGCs have also proven to be K^+^-permeable and involved in plant development and responses to environmental stimuli. Sugarcane is an important sugar and energy crop worldwide. However, reports on CNGC genes in sugarcane are limited.

**Results:**

In this study, 16 CNGC genes and their alleles were identified from *Saccharum spontaneum* and classified into 5 groups based on phylogenetic analysis. Investigation of gene duplication and syntenic relationships between *S*. *spontaneum* and both rice and *Arabidopsis* demonstrated that the CNGC gene family in *S. spontaneum* expanded primarily by segmental duplication events. Many *SsCNGC*s showed variable expression during growth and development as well as in tissues, suggesting functional divergence. Light-responsive *cis*-acting elements were discovered in the promoters of all the identified *SsCNGC*s, and the expression of most of the *SsCNGC*s showed a diurnal rhythm. In sugarcane, the expression of some *SsCNGC*s was regulated by low-K^+^ treatment. Notably, *SsCNGC13* may be involved in both sugarcane development and its response to environmental stimuli, including response to low-K^+^ stress.

**Conclusion:**

This study identified the CNGC genes in *S*. *spontaneum* and provided insights into the transcriptional regulation of these *SsCNGC*s during development, circadian rhythm and under low-K^+^ stress. These findings lay a theoretical foundation for future investigations of the CNGC gene family in sugarcane.

**Supplementary Information:**

The online version contains supplementary material available at 10.1186/s12864-023-09307-3.

## Introduction

Calcium ions (Ca^2+^) are ubiquitous and important second messengers in all eukaryotes [1] and participate in a variety of physiological, biochemical, and metabolic processes. In plants, Ca^2+^ is involved in plant growth regulation; development; responses to abiotic [2] and biotic [3] factors [4, 5]; and processes such as pollen tube and root hair growth [6], senescence programming [7], responses to low-potassium (K^+^) stress [8] and pathogen-associated molecular pattern (PAMP)-triggered immunity [9–11]. After a stimulus is detected, a specific Ca^2+^ influx occurs immediately and serves as a specific Ca^2+^ signal. The occurrence of Ca^2+^ influx in plant cells is based on Ca^2+^-permeable channels that are located in the plasma membrane and that can deliver Ca^2+^ into the cytoplasm from the extracellular matrix or from intracellular stores [12, 13]. In plants, several putative Ca^2+^-permeable channels have been identified, including cyclic nucleotide-gated channels (CNGCs) and glutamate receptors (GLRs); annexins and several types of mechanosensitive channels [14]; mid-complementing activity channels (MCA) [15, 16]; and hyperosmolality-gated Ca^2+^ permeable channel 1.3 (OSCA1.3) [17, 18]. Notably, the members of CNGC family have proven to be broadly involved in and critical to both development and stress resistance in plants [19].

CNGCs are evolutionarily conserved 3’,5’-cyclic adenosine/guanosine monophosphate (cAMP/cGMP)-gated ion channels that exist widely in animals and plants [20]. All CNGC proteins are mainly composed of six transmembrane domains (TM1-TM6) and a pore region (P) located between TM5 and TM6. Moreover, CNGCs also contain a calmodulin-binding domain (CaMB) and a cyclic nucleotide-binding domain (CNBD). There is a phosphate binding cassette (PBC) and a hinge region adjacent to the PBC in the CNBD [19]. However, there is a difference in CNGC structure between plants and animals. In plants, both CNBD and CaMBD are located in the cytosolic CNGC C-terminal, and there is an overlap at the C-terminal side of CNBD [21]. However, in animals, the two domains are located in the N-terminal and C-terminal, respectively [16]. Interestingly, an N-terminal CaMBD has been identified in AtCNGC12 [22].

To date, the CNGC gene family in many plants has been identified, and the members in various plant species vary in quantity from 9 [23] to 47 [19, 24]. Generally, plant CNGCs have been classified into 5 groups: Groups I, II, III, IVa, and IVb. According to previous reports, CNGC members are involved in responses to a wide range of developmental and environmental stimuli [20].

In *Arabidopsis thaliana*, AtCNGC6 and AtCNGC9, together with the leucine-rich repeat (LRR) RLK CLAVATA1 (CLV1), are essential for the elevation of [Ca^2+^]_cyt_ and for stem cell fate in roots [25]. *AtCNGC16* and *AtCNGC18* were found to primarily be expressed in pollen. Loss of *AtCNGC18* function leads to defects in pollen tube growth and growth into the transmitting tract and results in male sterility [26]. *AtCNGC16* is crucial for pollen tolerance to heat, drought and external calcium chloride during germination and the initiation of pollen tube tip growth. Disruptions of *Atcngc16* have been found to result in a more than 10-fold stress-dependent reduction in seed set [27].

*Arabidopsis CNGC2* and *CNGCb* from *Physcomitrella patens* are reported to control land plant thermal sensing and to have been acquired for thermotolerance. *Atcngc2* and *Ppcngcb* mutant plants show growth retardation and a hyperthermosensitive phenotype [28]. In addition, *AtCNGC6* also mediates heat-induced Ca^2+^ influx, which promotes the expression of *heat shock protein (HSP)* genes and increases thermotolerance [29]. In rice (*Oryza sativa*), *OsCNGC14* and *OsCNGC16* are crucial for Ca^2+^ signals induced by temperature stresses. The null mutant of *Oscngc14* or *Oscngc16* has been shown to display higher accumulation levels of hydrogen peroxide, increased cell death, and reduced survival rates under heat or chilling stress [30]. Moreover, overexpression of *AtCNGC19* and *AtCNGC20* can enhance plant tolerance to salt stress [31].

CNGCs have also been confirmed to contribute to plant immunity by increasing cytosolic Ca^2+^ [3]. First, *AtCNGC2* was found to be involved in plant immunity. The *atcngc2* null mutant was characterized as “*defense, no death 1*” (*dnd1*), which showed a deficient autoimmune phenotype with high salicylic acid (SA) accumulation and a constitutive PATHOGENESIS-RELATED (PR) gene [32]. Another *Arabidopsis* “defense, no death” mutant was characterized as yet another CNGC mutant, the *atcngc4* mutant [33]. *AtCNGC2* and *AtCNGC4* were also confirmed to work together and assemble into a functional Ca^2+^ channel that mediated Ca^2+^ influx after flg22 (a bacterial flagellin peptide that is always used as a PAMP) was recognized by the receptor complex [34]. The rice *OsCNGC9* was also labeled *CELL DEATH and SUSCEPTIBLE to BLAST 1* (*CDS1*), and its deletion was found to impair plant blast resistance. Moreover, overexpression of *OsCNGC9* can enhance rice pattern-triggered immunity (PTI) and resistance to blast [35].

In tomato, *SlCNGC16* is member of group IVb, and silencing of one of these genes enhances resistance to *Pythium aphanidermatum* and *Sclerotinia sclerotiorum* while reducing resistance to tobacco rattle virus [36]. Moreover, the members of group IVb in wheat (*Triticum aestivum* L.), *TaCNGC14* and *TaCNGC16*, contribute to plant resistance against *Puccinia striiformis* f. sp. *tritici* (*Pst*) [24]. CNGC genes in cotton (*Gossypium hirsutum* L.) were also thought to contribute to resistance against *Verticillium dahliae* [37]. AtCNGC20 has proven to be important for regulated immunity, and the gain-of-function mutant *Atcngc20-4* (*AtCNGC20*^*L371F*^) with misregulation of Ca^2+^-permeability exhibits autoimmunity and leads to an increased plant defense response [38]. *AtCNGC19* in the same subfamily as *AtCNGC20* also mediates basal defense signaling to regulate *Pirformospora indica* colonization of *Arabidopsis* roots [39]*.* In addition, *AtCNGC19* has proven to activate herbivory-induced Ca^2+^ influx and plant defense against *Spodoptera litura.* Loss of *AtCNGC19* function results in decreased defense against *S. litura* [40].

Sugarcane (*Saccharum* spp.) is an important C4 graminoid crop worldwide that can be used for renewable fuels and sucrose production. Sugarcane is a polyploid interspecific hybrid with singularly complex genomes. Therefore, studies on functional genes in sugarcane are slow to develop. To the best of our knowledge, there have been few studies on the *CNGC* gene family in sugarcane. In 2018, the allele-defined genome of *Saccharum spontaneum* L. (AP85-441), one ancestor of modern sugarcane, was published and now serves as a resource to accelerate sugarcane functional gene studies [41]. In this study, we identified the members of the *CNGC* gene family in *S. spontaneum* based on genome-wide sequence information. Moreover, a series of bioinformatics analyses and expression profiles of these *CNGC* genes during plant growth and in response to low K^+^ conditions were performed. The results of this study could provide important information and lay a theoretical foundation for further functional characterization of *CNGC* genes in sugarcane.

## Materials and methods

### Plant materials and growth conditions

The sugarcane commercial hybrid YT99-66 (bred by Institute of Bioengineering, Guangdong Academy of Sciences) was used as the experimental material in this study for identification of CNGC genes involved in sugarcane response to low-K^+^ stress. Healthy single-bud sets of YT 99–66 were buried in the sand and cultured in a greenhouse. Forty-five-day after budding, sugarcane seedlings were hydroponically cultured for 1 month and then treated with low-K^+^. The culture medium was replaced every week, and the roots were collected at 0, 6, 12, 24, 48 and 72 h after treatment. All the materials were frozen in liquid nitrogen immediately after collection and stored at − 80 °C. There were three independent replicates in each treatment, and there were 15 seedlings in each group. All the samples were divided into two aliquots, one part for transcriptome sequencing and the other for validating gene expression.

### Identification and sequence analysis of CNGC gene family members in* S. spontaneum*

The *S. spontaneum* L. (AP85-441) genome [41] was used as the reference genome in this study. All the data for *S. spontaneum* used in this study were downloaded from the SGD (Saccharum Genome Database, http://sugarcane.zhangjisenlab.cn/sgd/html/download.html).

The identification of CNGC gene family members in *S. spontaneum* was carried out in three steps. First, the protein sequences of CNGC genes from Arabidopsis [42], rice [43] and maize [44] were retrieved from the Phytozome 12 database (https://phytozome.jgi.doe.gov/pz/portal.html) [45] and used as reference sequences for potential *S. spontaneum* CNGC identification. These reference CNGC sequences were searched against all the *S. spontaneum* protein sequences using National Center for Biotechnology Information (NCBI) BLASTp searches (http://www.ncbi.nlm.nih.gov/) with a threshold *e*-value < e^−5^. Proteins from *S. spontaneum* that were homologous with one of the reference sequences were considered candidate CNGC members. Second, CNGC candidates that contain both the cNMP binding domain (CNBD, Pfam No. PF00027) and ion trans domain (Pfam No. PF00520) were screened using HMMER v5.0.1 software (domE = e^–5^) with the Pfam database (https://www.ebi.ac.uk/interpro/). Finally, protein domains and domain structure analysis of CNGC candidates were performed using the Simple Modular Architecture Research Tool (SMART) database (http://smart.embl-heidelberg.de/) and the InterProScan database (http://www.ebi.ac.uk/Tools/pfa/iprscan5/). Proteins with more than 200 amino acids and a CNBD that contained the PBC and hinge regions were recognized as members of the CNGC gene family in *S. spontaneum* and named SsCNGCs.

The ExPASy Proteomics Server (https://web.expasy.org/protparam/) was employed for protein length, molecular weight, theoretical pI and instability index analysis of SsCNGC proteins. The online tool Softberry (http://linux1.softberry.com/berry.phtml) was used to predict the subcellular location of SsCNGC proteins.

### Multiple sequence alignment and phylogeny analysis

Multiple sequence alignment and phylogenetic analysis of SsCNGCs with all CNGC proteins from *Arabidopsis*, rice and maize were performed using the MUSCLE program [46]. The conserved domains of CNGCs were checked manually. Phylogenetic analysis was performed using MEGA 7.0 software under the MUSCLE model [47]. The bootstrap test was set as 1000 replicates. Scale bars correspond to 0.1 amino acid substitutions.

### Chromosome location, gene structure and protein conserved motif analysis

The exon‒intron structure of *SsCNGC*s was analyzed using the online tool Gene Structure Display Server (GSDS, http://gsds.cbi.pku.edu.cn/) [48] based on genome annotation data downloaded from the SGD database (http://sugarcane.zhangjisenlab.cn/sgd/html/index.html).

The conserved motif analysis of SsCNGCs was performed with Multiple Em for Motif Elicitation (MEME) online software (http://meme-suite.org/tools/meme) [49]. The maximum motif search value was set at 15, and the optimum motif width was 10–100 aa. Other parameters are default.

### Chromosome location, duplication, and syntenic analyses

The chromosomal locations of the *SsCNGC*s were determined by the genome annotation files and mapped using the SVG package of the Perl programming language.

Homology between protein sequences encoded by *SsCNGC*s was analyzed by the BLASTp program. These results were submitted to the duplicate gene classifier script in MCScanXv8.0 software for potential gene duplication events identified with a cutoff *E*-value ≤ 1e^−5^. The collinearity of multiple species was constructed by using McScanXv8.0 software, and the SVG model was drawn using Perl.

### Cis-acting element analysis

According to the genome sequence from the SGD database, 2 kb DNA sequences upstream of the start codon of each SsCNGC were obtained and submitted to the online tool PlantCARE (http://bioinformatics.psb.ugent.be/webtools/plantcare/html/) server [50] for putative *cis-*acting element prediction.

### Expression pattern analysis

Transcription data for different *S. spontaneum* leaf sections and growth and development periods were downloaded from the SGD database. Transcriptome data of sugarcane hybrid YT99-66 root samples treated with low-K^+^ were used for *SsCNGC* gene transcriptional expression under low-K^+^ treatment. Fragments per kilobase per million (*FPKM*) values of *SsCNGC*s extracted from these transcriptome data were normalized by z score and hierarchically clustered by Pheatmap v1.0.8 R package.

To validate the expression of *SsCNGC*s under low-K^+^ stress, total RNA was extracted from the root samples of YT99-66 after low-K^+^ treatment using *RNAiso Plus* (TaKaRa, Japan). The cDNA was obtained using the *PrimeScript™ RT reagent Kit with gDNA Eraser* (Perfect Real Time, Takara, Japan) according to the instruction. RT-qPCR was preformed using cDNA and TB Green® Premix Ex Taq™ II (Tli RNaseH Plus, Takara, Japan) on the *LightCycler 96* (Roch, USA) with primers listed in Supplementary Table 1. The 2^−∆∆CT^ approach was used for quantifying relative gene expression levels. *SsAPRT* was as used as normalization controls.

## Results

### Identification of CNGC genes in *S. spontaneum*

**Fig. 1:**
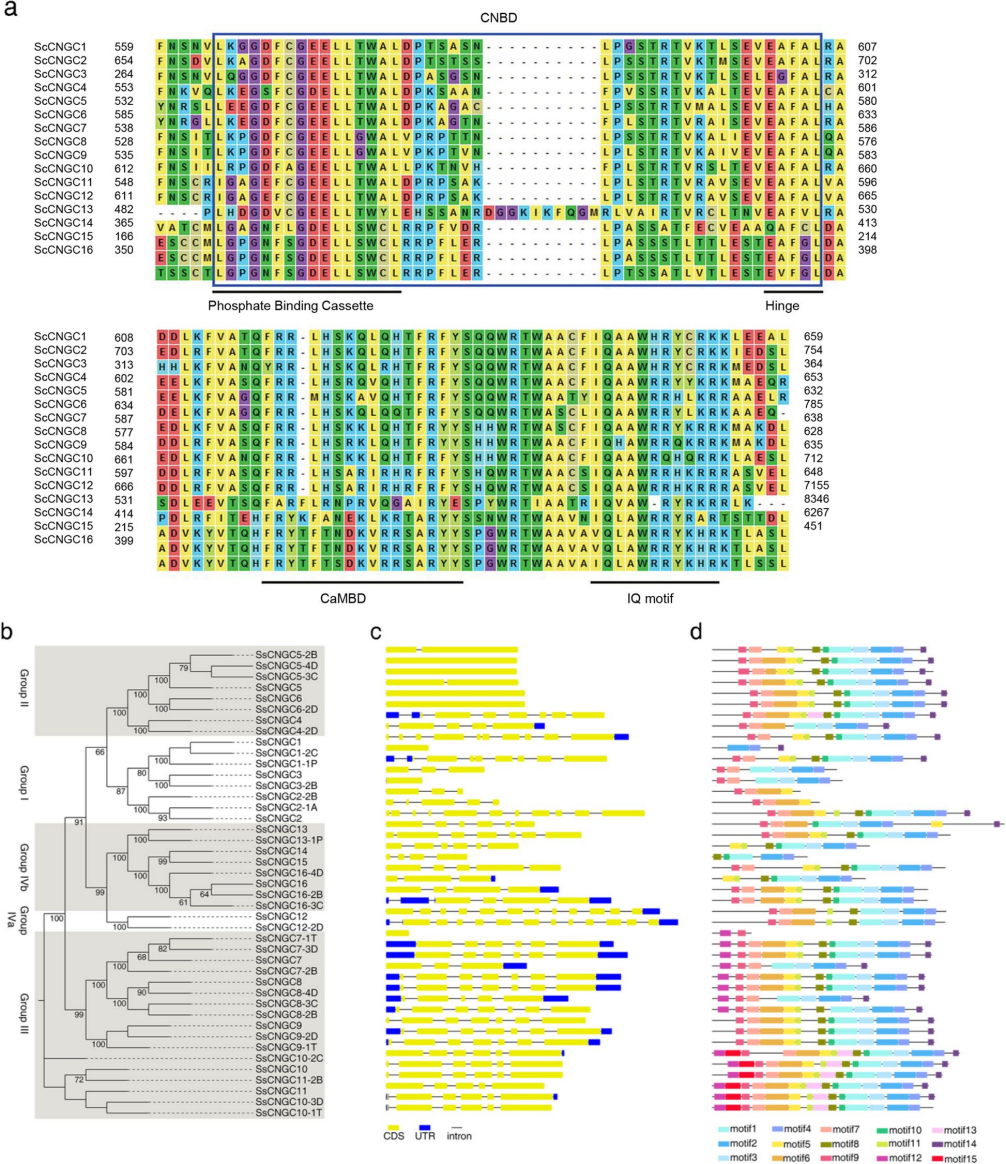
Amino acid sequence alignment, phylogenetic tree, gene structures, and conserved motif analysis of the CNGC gene family members in *S. spontaneum*. **a** Amino acid sequence alignment of SsCNGCs. CNBD is highlighted by blue box, Phosphate Binding Cassette, Hinge, CaMBD and IQ motif are highlighted by black underline. **b** The phylogenetic tree was constructed based on the aa sequence of SsCNGCs using MEGA 7.0 and the Multiple Sequence Comparison by Log-Expectation (MUSCLE) method. **c** Gene structures of *SsCNGC*s. Yellow and blue boxes indicate exons of coding and noncoding regions, respectively; black lines indicate introns. **d** Conserved motifs of SsCNGC proteins were discovered using MEME tools. The order of the motifs is consistent with their position in the protein sequence. Different colored boxes represent different conserved motifs

To identify CNGC genes in *S. spontaneum*, the homologous genes in *Arabidopsis*, rice and maize (*Zea mays*) were obtained using the Protein–Protein Basic Local Alignment Search Tool (BLASTp) algorithm. Homologous genes containing CNGC*-*specific domains, CNBD, CaMBD and IQ motifs as well as a most conserved phosphate binding cassette (PBC) and a “hinge” region in the CNBD were identified as *SsCNGC* genes (Fig. 1a and b). In this study, a total of 16 *SsCNGC* genes with 27 alleles were identified and named *SsCNGC1-16* according to their phylogenetic relationships with *CNGC* genes in rice and based on allelic annotation of the sugarcane genome [41].

Detailed physiological and biochemical information of these 16 *SsCNGC* genes is listed in Table 1. Most of the *SsCNGC* genes identified have 2 ~ 4 alleles except for *SsCNGC14* and *SsCNGC15*, which have only 1 allele (Supplementary Table 2). However, alleles of some *SsCNGC* genes were truncated, such as those of *SsCNGC1-2C, SsCNGC2-1A/2B,* and *SsCNGC3-2B*. The nucleic and amino acid sequences of *SsCNGC* genes and their alleles are shown in Supplementary Files 1 and 2, respectively. The coding sequence (CDS) lengths of *SsCNGC* genes and their alleles ranged from 378 bp (*SsCNGC7-1 T*) to 2478 bp (*SsCNGC2-1P*), with an average length of 1851 bp. The SsCNGC protein length ranged from 126 to 826 amino acids (aa), with an average of length of 633 aa. The predicted molecular weight (Mw) of these SsCNGC proteins ranged from 14.1 to 102.30 kDa, and the theoretical isoelectric point (pI) ranged from 8.65 (*SsCNGC3-2B*) to 10.34 (*SsCNGC15-1B*).

**Table 1:** Overview of CNGC genes in *S. spontaneum*

Name	ID	Chr.	Position	CDS Length (bp)	Protein Length (aa)	No. of Intron	No. of Exon	WM (kDa)	pI	GRAVY
*SsCNGC1*	Sspon.04G0014070-1A	Chr4A	51882842–51906635	2193	731	9	10	83.41	9.25	-0.128
*SsCNGC2*	Sspon.08G0008040-1P	Chr8A	31202765–31214757	2478	826	10	11	94.52	9.49	-0.197
*SsCNGC3*	Sspon.04G0014080-1A	Chr4A	51908179–51911281	1197	399	2	3	46.04	8.78	-0.201
*SsCNGC4*	Sspon.05G0022360-1B	Chr5B	3204292–3211306	2007	717	7	8	81.74	9.09	-0.08
*SsCNGC5*	Sspon.01G0024280-1A	Chr1A	87156289–87158432	2106	702	1	2	80.32	9.44	-0.112
*SsCNGC6*	Sspon.02G0031610-1A	Chr2A	115677914–115680169	2256	752	0	1	85.28	9.53	-0.204
*SsCNGC7*	Sspon.04G0002010-1A	Chr4A	6199865–6205159	2106	702	5	6	79.98	9.04	-0.04
*SsCNGC8*	Sspon.08G0012670-1A	Chr8A	54096149–54100723	2043	681	6	7	78.67	9.07	-0.148
*SsCNGC9*	Sspon.02G0036020-1B	Chr2B	22343268–22348277	2133	711	6	7	82.06	9.12	-0.23
*SsCNGC10*	Sspon.04G0024390-1B	Chr4B	19113351–19116208	2268	756	5	6	87.71	9.23	-0.094
*SsCNGC11*	Sspon.04G0008000-1A	Chr4A	22519276–22521836	2076	692	4	5	80.26	9.53	-0.098
*SsCNGC12*	Sspon.04G0031130-1C	Chr4C	7104138–7112895	2235	745	11	12	85.28	9.04	-0.159
*SsCNGC13*	Sspon.01G0049590-1B	Chr1B	113439670–113443089	2805	934	5	6	102.30	9.19	-0.106
*SsCNGC14*	Sspon.03G0005320-1A	Chr3A	15234428–15236678	1512	504	5	6	57.22	9.79	-0.019
*SsCNGC15*	Sspon.03G0030490-1B	Chr3B	21205167–21206785	915	305	3	4	34.84	10.24	-0.302
*SsCNGC16*	Sspon.07G0004720-1A	Chr7A	12173890–12175659	1473	491	2	3	55.39	9.86	-0.051

*MW* Molecular weight, *pI* the theoretical isoelectric point, *GRAVY* the grand average of hydropathicity

The cluster analysis, gene structures and conserved protein motifs of all *SsCNGC*s and alleles were also investigated. Few common features were found between the *SsCNGC*s within the same group (Fig. 1). Except for *SsCNGC6* and its allele *SsCNGC6-2D*, as well as *SsCNGC1-2C*, *SsCNGC3-2B*, and *SsCNGC5-3C/4D*, all the other *SsCNGCs* and their alleles have introns, with exon numbers ranging from 2 to 13 (Fig. 1c). The conserved motifs of SsCNGC proteins were identified with the online Multiple Em for Motif Elicitation (MEME) program. The details of the sequence logo of motifs are shown in Supplementary Fig. 1. Notably, 93% of SsCNGC proteins contain motif 2 and motif 4, indicating that these two motifs were most common among the various CNGC gene family members. In addition, motif 2 represents the most conserved sequence in the CNBD domain, and the ion trans domain might be composed of motifs 7, 6, 12, 5, 11, and 13 (Fig. 1d).

### Phylogenetic and syntenic analysis of SsCNGCs

**Fig. 2:**
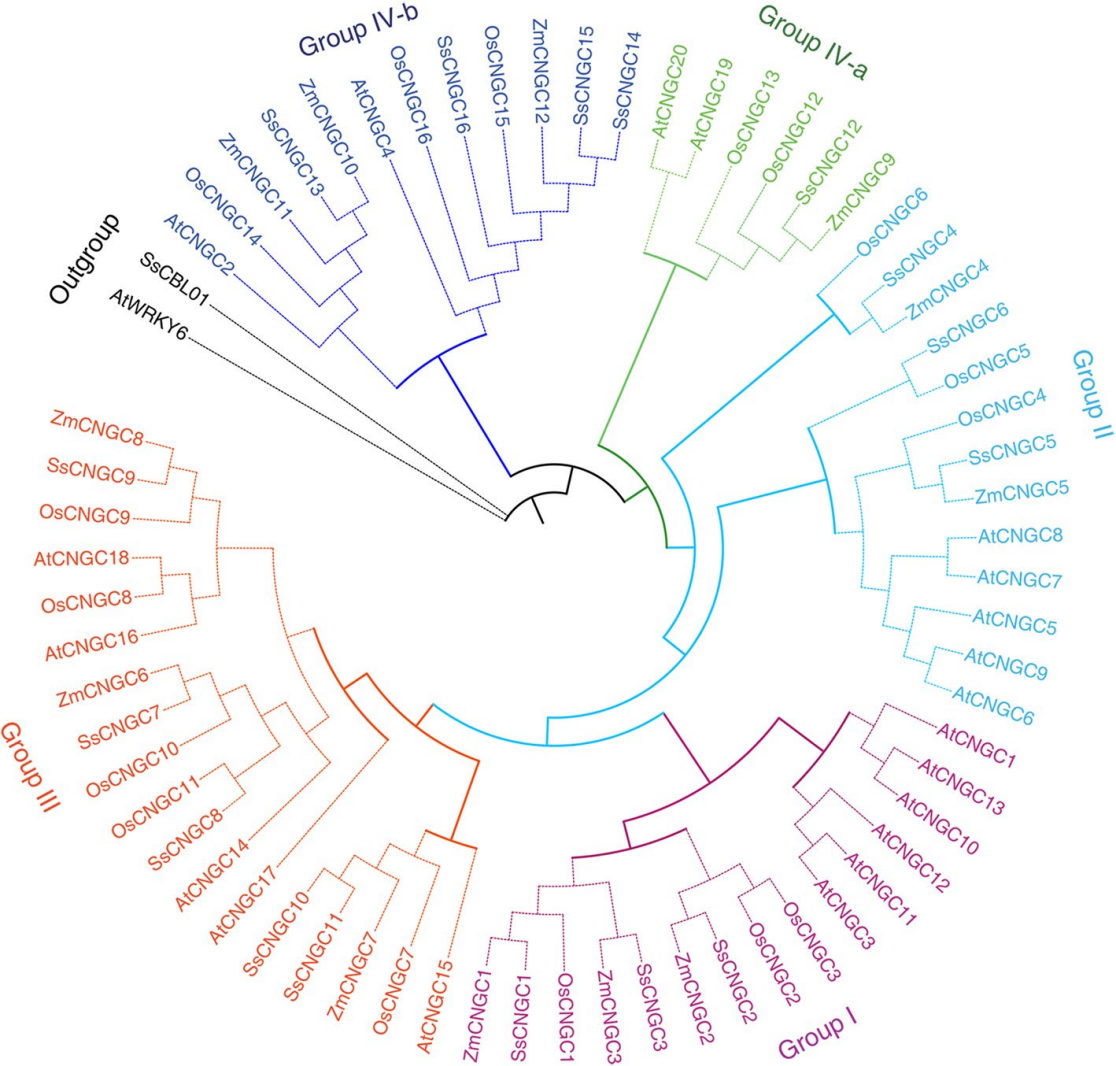
Phylogenetic analysis of CNGCs from *S. spontaneum* and *A. thaliana*, rice and maize. Multiple sequence alignment of 16 putative SsCNGCs with 20 AtCNGCs, 16 OsCNGCs and 12 ZmCNGCs was performed by using MEGA 7.0, which was also used to create the unrooted maximum likelihood tree under the MUSCLE model. The bootstrap test was carried out with 1,000 replicates

To explore the phylogenetic relationship of SsCNGC proteins, an unrooted phylogenetic tree was constructed based on the alignment results of the available full-length amino acid sequences of *Arabidopsis*, rice, maize and *S. spontaneum* CNGCs (Supplementary file 3). As shown in Fig. 2, all the CNGC proteins could be clustered into four groups as described by Jarratt-Barnham et al*.* (2021) [19]. Group IV was divided into two subgroups (groups IVa and IVb). Similar to the CNGCs in rice and maize, the SsCNGCs in each group exhibited a great diversity in number. For example, groups III and IVa contained the most and the fewest members, 5 and 1, respectively. This is basically similar to the quantities in these groups in rice and maize but not to the quantities in *Arabidopsis,* which had the highest CNGC number in group I. *Arabidopsis* and rice are the model plants of dicots and monocots, respectively. In general, all CNGCs and their subgroups are present in dicots and monocots. It is speculated that the appearance of most CNGCs in plants predated monocot-dicot divergence.

**Fig. 3:**
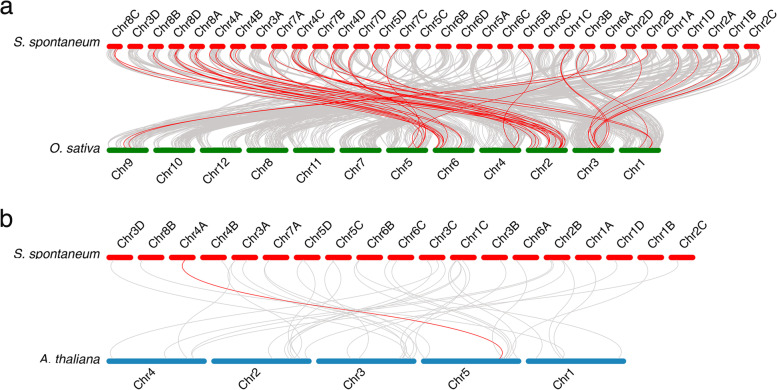
Syntenic analysis of *CNGC* genes between *S. spontaneum* and both rice (**a**) and *Arabidopsis* (**b**). The *S. spontaneum*, rice and *Arabidopsis* chromosomes are represented by red, green and blue bars, respectively. Gray lines in the background indicate the collinear blocks within two different genomes, while the red lines highlight the syntenic *CNGC* gene pairs. Schematic representations were displayed by using the SVG Perl package

To further investigate the origin and evolution of *CNGC*s in *S. spontaneum*, the syntenic relationships between *S. spontaneum* and both rice and *Arabidopsis* were examined using McScanXv8.0 [51]. The results indicated that a great number of syntenic relationship events existed between rice and *S. spontaneum*, including many CNGC gene pairs. This means that many consensuses in *SsCNGC*s may have existed before the species divergence between rice and *S. spontaneum* (Fig. 3a). However, there was only one collinear gene pair between the *Arabidopsis* and *S. spontaneum* CNGC genes, suggesting that the origin of this gene pair was very old (Fig. 3b).

### Chromosome location and duplication events of CNGC family members in *S. spontaneum*

**Fig. 4:**
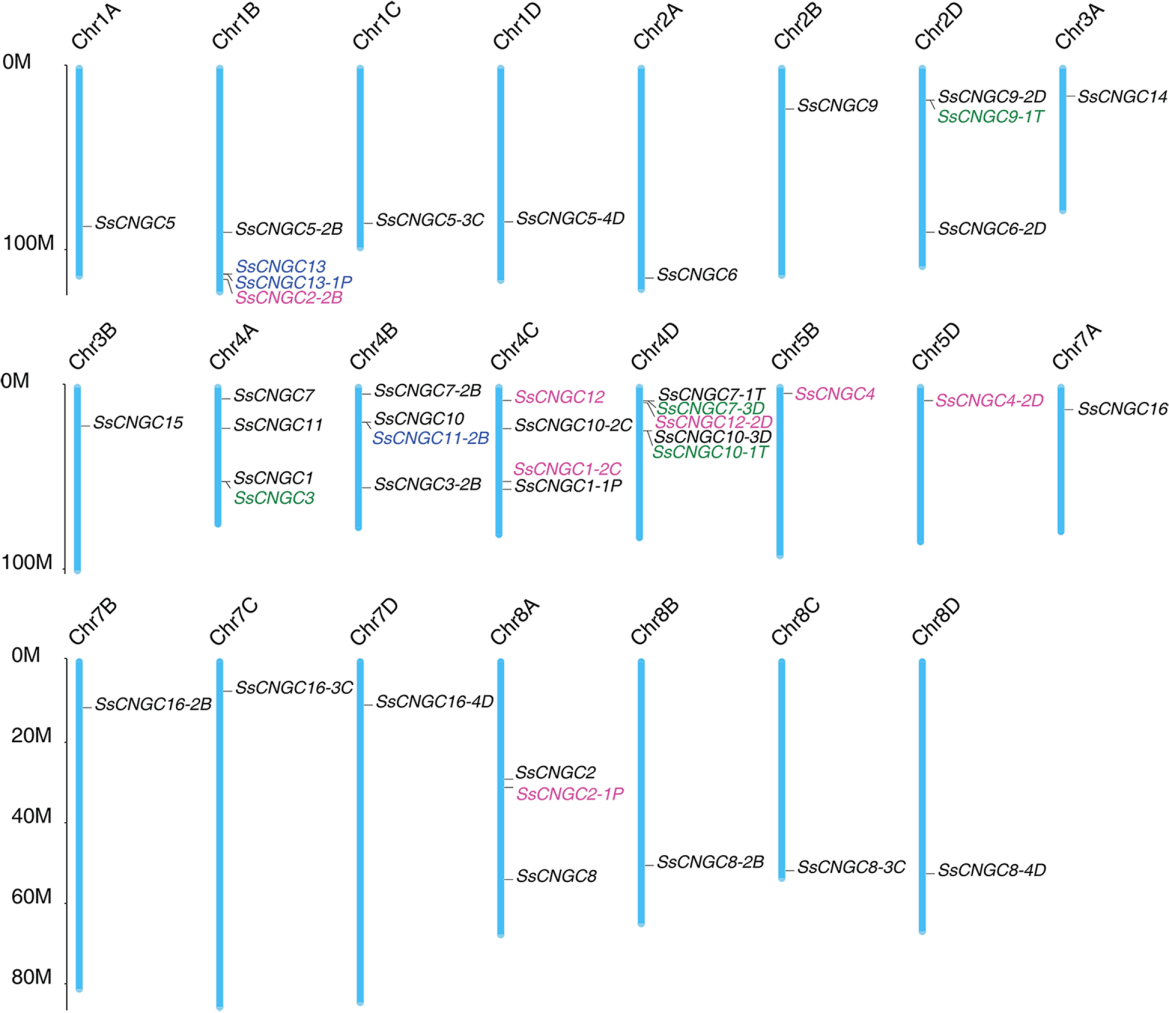
Chromosome location and duplication of *SsCNGC*s in *S. spontaneum*. All *SsCNGC*s and alleles were mapped onto the 23 *S. spontaneum* chromosomes*.* The chromosome number is shown at the top of each chromosome. The scale is in megabases (Mb). The seven dispersed duplication genes are in magenta; the four tandem duplication genes are in green; the three proximal duplication genes are in blue; the twenty-nine segmental duplication genes are in black

The chromosome location information for CNGC gene family members showed that they were unevenly distributed on the 23 *S. spontaneum* chromosomes (Fig. 4). The number of *SsCNGC* genes mapped on each chromosome varied widely and ranged from 1 to 5. Among the 23 chromosomes, Chr4D had 5 *SsCNGC*s, Chr1B and Chr4A/B/C/D each had 4 *SsCNGC*s, and Chr8A and Chr2D had 3 *SsCNGC*s, while only one *SsCNGC* was found to be located on the other chromosomes. Almost all *SsCNGC* genes and their alleles were located on homologous chromosomes, except for *SsCNGC2,* which is located on Chr8A, with two alleles located on Chr8A (*SsCNGC2-1P*) and Chr1B (*SsCNGC2-2B*) respectively.

Gene duplication is responsible for gene family evolution and differentiation and even participates in the occurrence of both evolutionary novelties and increases in biological complexity (including adaptation to stresses and resistance to diseases) as well as in speciation [52–54]. Genome-wide duplication events of *SsCNGC*s were analyzed in this study (Fig. 4). The results indicated that 3 (7%, marked in blue), 4 (9.3%, marked in green) and 7 (16.3%, marked in magenta) *SsCNGC*s were duplicated from proximal, tandem and dispersed duplication events, respectively, and that the other 29 (67.4%) *SsCNGC*s originated from segmental duplication.

### Prediction of *cis*-acting acting regulatory elements in the promoter of *SsCNGC*s

**Fig. 5:**
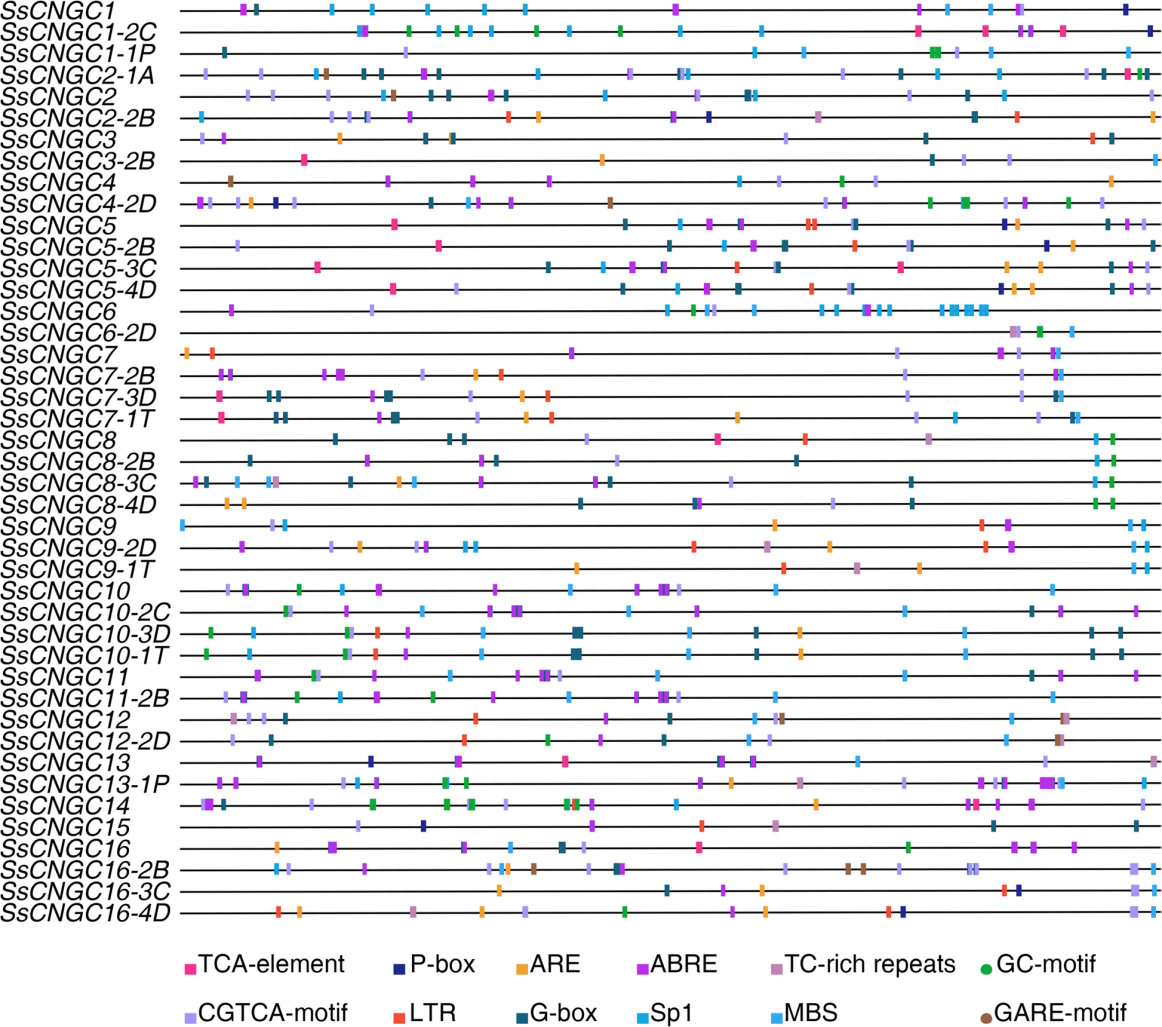
The *cis-*acting elements in the 2 kb 5’-upstream promoter regions of the *SsCNGC*s and alleles

Investigation of *cis-*acting elements in the promoter region was conducted to better elucidate the functions of the *SsCNGC*s. In this study, 2.0 kb sequences upstream from the transcriptional start site of the *SsCNGC*s were extracted from the gff3 file and submitted to the Plant Cis-Acting Regulatory Element (PlantCARE) database for *cis*-element identification. According to the functional annotation, these *cis-*acting elements can be divided into three categories: those involved in development processes, hormone signaling and environmental responses (Supplementary Table 3 and Fig. 5). All the promoter sequences of *SsCNGCs* contained several light-responsive elements such as Sp1, G-box, and ATCT-motif, suggesting that *SsCNGC*s may be involved in the light responses of *S. spontaneum*. Moreover, the promoters of *SsCNGC*s also contained phytohormone responsiveness elements that are always involved in plant development as well as responses to biotic and abiotic stresses. The promoters of all the *SsCNGC*s except for *SsCNGC9-1 T* contained the CGTCA motif, a cis-acting acting regulatory element involved in methyl jasmonate (MeJA) responsiveness. In addition, the abscisic acid (ABA) responsiveness element ABRE was discovered in 40 *SsCNGC*s, although it was absent from *SsCNGC1-1P, SsCNGC6-2D* and *SsCNGC9-1 T*. In addition, the promoters of all the *SsCNGC*s contained several ABRE elements, with an average of 4.4. Additionally, all the *SsCNGCs* contained *cis*-acting acting elements that participate in defense and/or stress responsiveness, including low-temperature response (LTR) *cis*-acting elements involved in low-temperature responsiveness, MYB binding site (MBS) *cis*-acting elements involved in drought inducibility, TC-rich repeat enhancer *cis*-acting elements involved in defense and stress responsiveness, and so on. These results indicate that *SsCNGC*s may perform diverse functions to regulate *S. spontaneum* development and to respond to environmental stresses.

### Expression profiles of *SsCNGC*s across development and leaf segment gradients

**Fig. 6:**
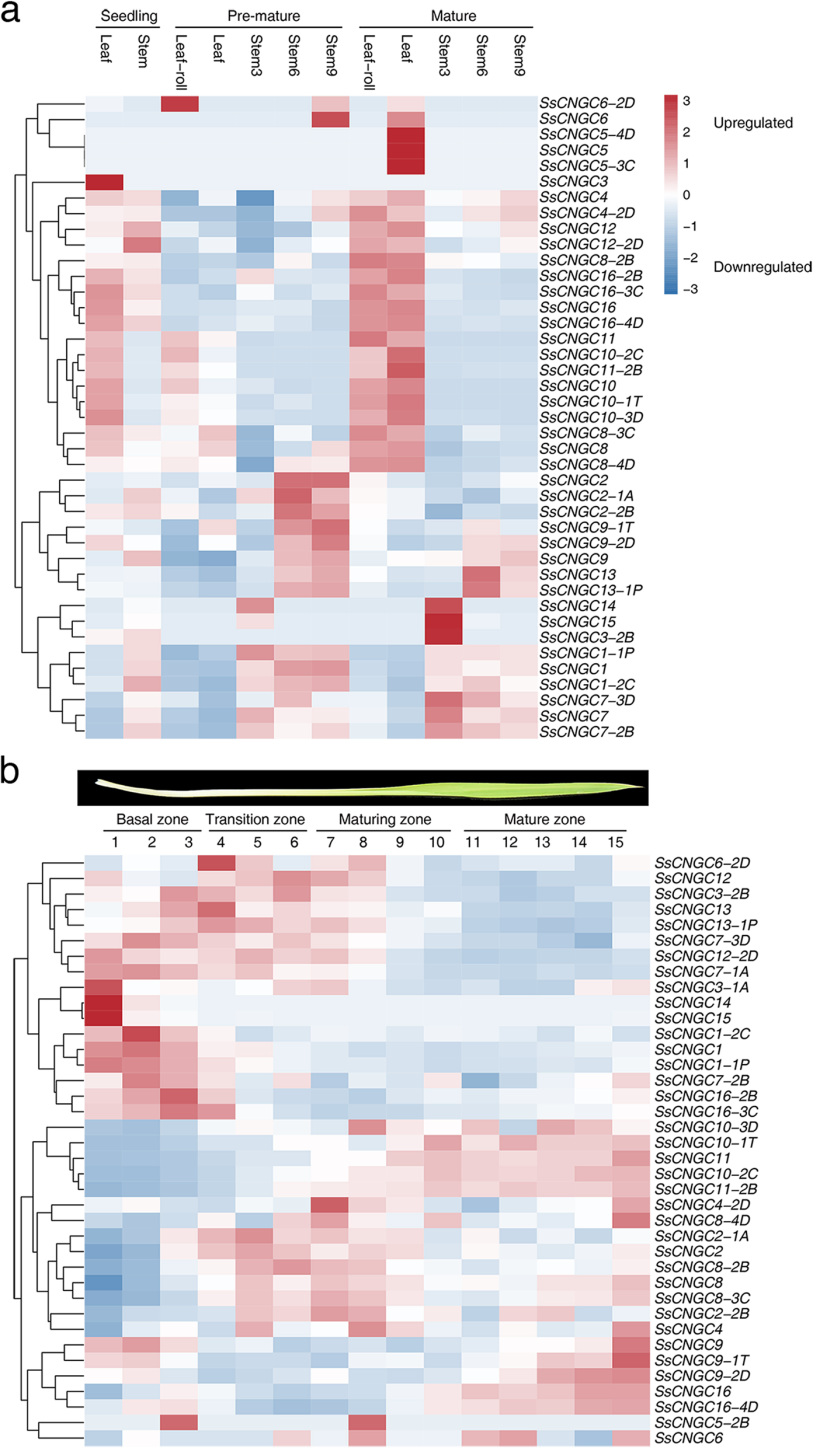
Expression pattern of *SsCNGC*s in *S. spontaneum* in different tissues at three developmental stages (**a**) and across leaf gradients (**b**) based on FPKM*.* Tissues are indicated at the top of each column. Stems 3, 6 and 9 are the 3rd (immature stem), 6th (maturing stem) and 9th (mature stem) internodes from the terminal bud. **b**) The mature leaves of *S. spontaneum* were divided into 15 segments (1–15) and four regions: the basal zone (sink tissue), transitional zone (sink‒source transition), maturing zone and mature zone (fully differentiated, active photosynthetic zone)

Tissue-specific expression patterns are interrelated with the functions of genes. In this study, transcriptome profiles of *SsCNGC*s in different tissues at different developmental stages of *S. spontaneum* were analyzed based on the RNA-seq data from the Saccharum Genome Database (SGD) (http://sugarcane.zhangjisenlab.cn/sgd/html/index.html, Fig. 6a, Supplementary Table 4). The *SsCNGC*s showed similar transcriptional profiles, and most of them showed tissue-specific expression patterns (Fig. 6a). *SsCNGC1*, *2*, *4*, *9*, and *13* were highly expressed in most tissues tested at various expression levels. *SsCNGC1* and *7*, together with their alleles, showed higher expression levels in stem tissues. *SsCNGC2*, *9* and *13·,* together with their alleles, showed significantly higher expression levels in maturing and mature stalk tissues (stem6 and stem9 tissues, the 6th and 9th internodes from the terminal bud) at the premature stage. Interestingly, several *SsCNGC*s, such as *SsCNGC16* and its alleles, showed higher expression levels in leaf roll and leaves at the seedling and mature stages but not at the premature stage. In different sections of mature leaves, *SsCNGC*s showed various expression levels (Fig. 6b). *SsCNGC1* and *7* showed a decreasing trend in expression from the basal zone to the mature zone, while *SsCNGC10*, *11*, *12*, and *16* showed an upward tendency. *SsCNGC2* and *8* were highly expressed in the transition and maturing zones. Transcripts of *SsCNGC14* and *15* showed high accumulation in the tender stems. The expression of *SsCNGC3*, *5* and *6* in the aerial tissues was low and was barely detected (Supplementary Table 4). Therefore, it can be speculated that their effects on growth and development were limited.

### Expression pattern of *SsCNGC*s in relation to the circadian rhythm

**Fig. 7:**
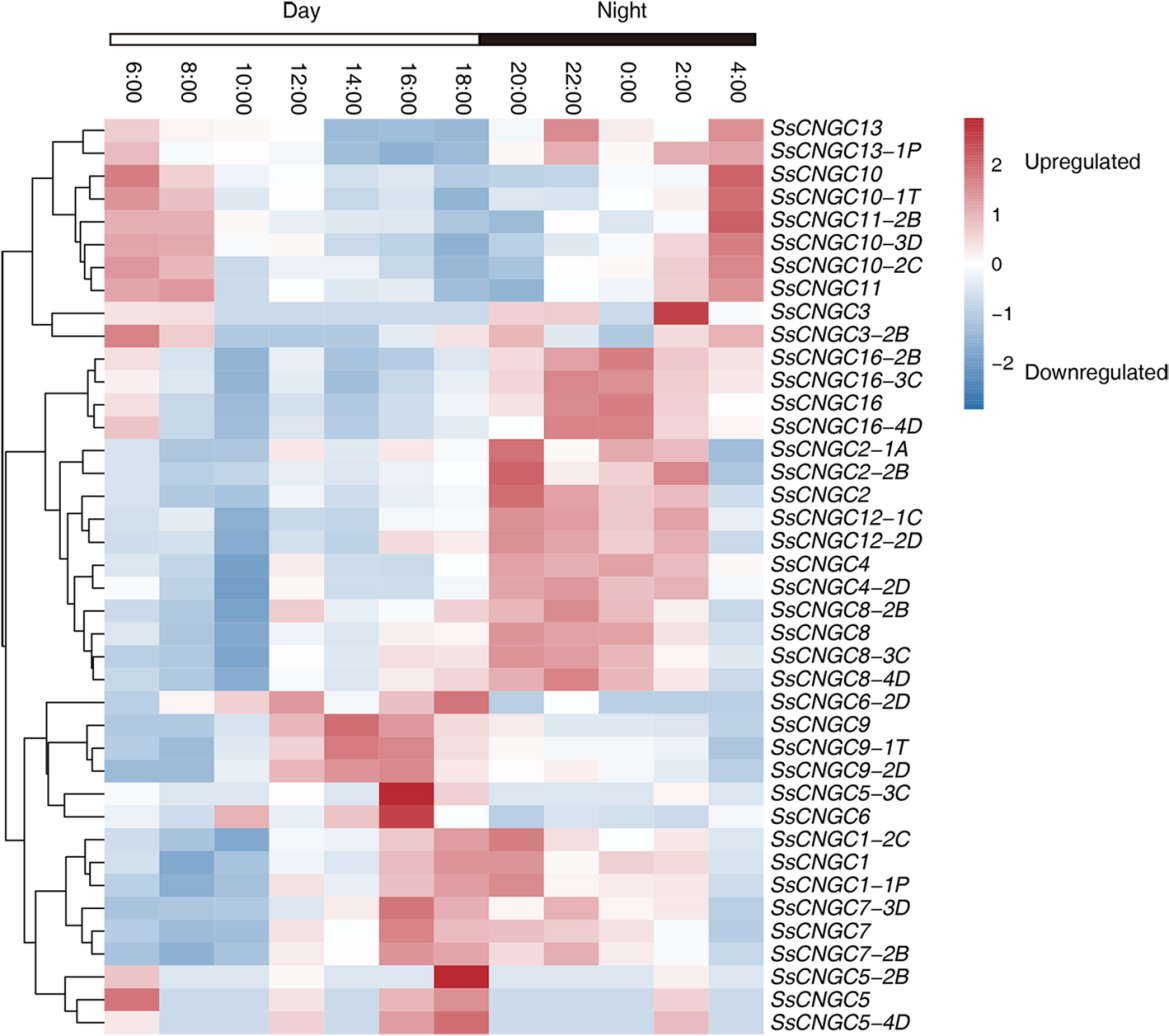
Expression analyses of *SsCNGC*s in relation to the circadian rhythm. The sampling time is indicated at the top of each column

According to the results of the previous analysis, *SsCNGC*s should participate in responses to light intensity changes in *S. spontaneum*. To investigate the roles of *SsCNGC*s in relation to the circadian rhythm, the expression profiles of *SsCNGC*s in mature leaves were analyzed at 2-h intervals (Fig. 7, Supplementary Table 5). The results showed that the expression of most detected *SsCNGC*s was regulated by the circadian rhythm. These *SsCNGC*s could be categorized into Groups 1, 2, and 3 based on their expression patterns, which had higher expression at dawn, afternoon and night, respectively (Fig. 7). Many *SsCNGC*s showed high expression at night, from 20:00 to 0:00 the next day, including *SsCNGC2*, *4*, *8*, *12* and *16*. The expression of *SsCNGC1* and *7* reached their peak values at dusk. Relatively high expression of *SsCNGC10* and *11* began at dawn and persisted from 4:00 to 8:00. Only *SsCNGC9* showed a high expression level in the afternoon. These results can be explained by the effects of light-responsive cis-acting elements at the promoters.

### Expression of ***SsCNGC***s was regulated by K^+^-deficient stress

**Fig. 8:**
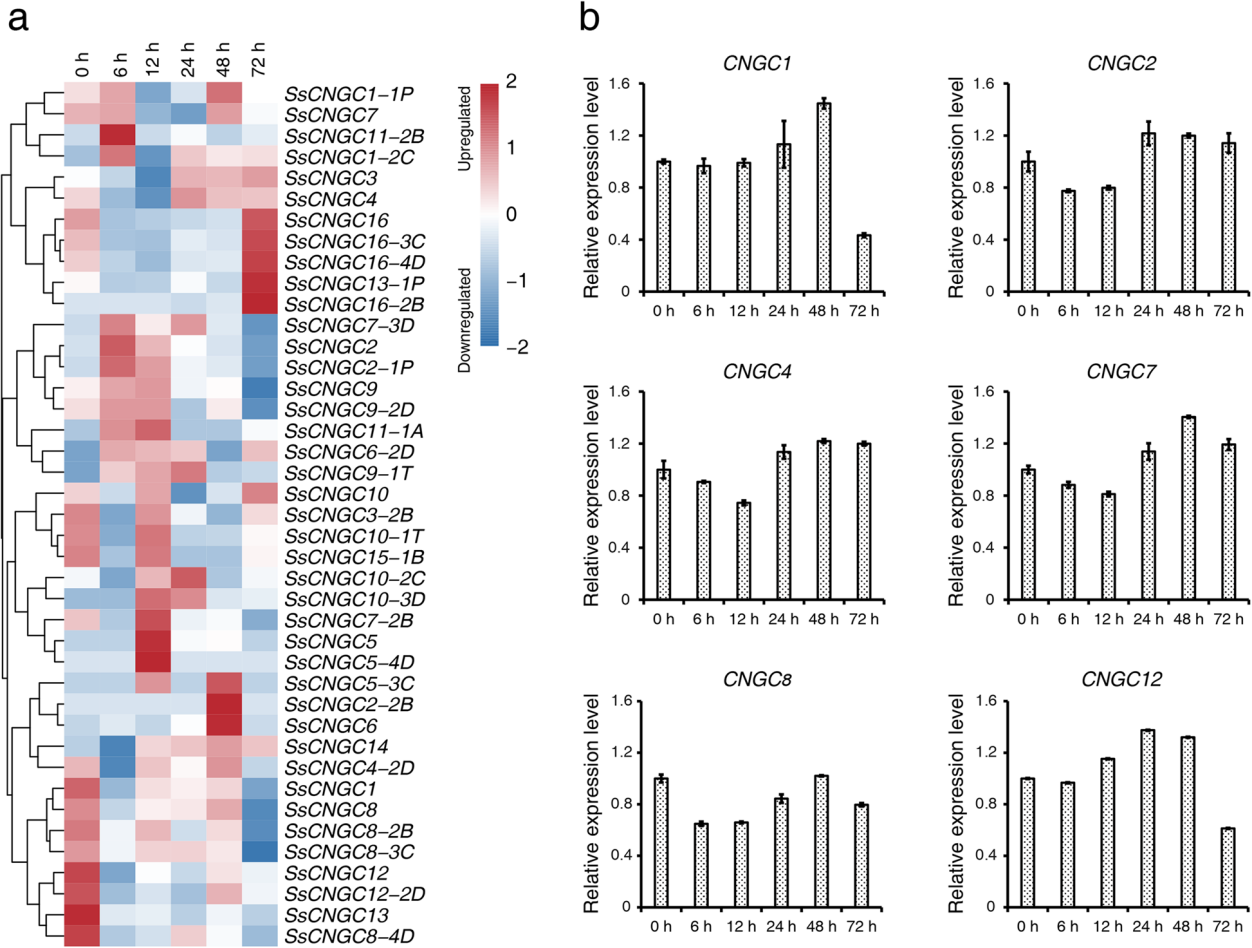
Expression analyses of *SsCNGC*s in the root of sugarcane YT 99–66 after low-K^+^ treatment. (**a**) Heatmap of the relative expression levels of *SsCNGC*s. The sampling time is indicated at the top of each column. (**b**) Relative expression of 6 *SsCNGC*s detected by RT-qPCR

As Ca^2+^ channels, CNGCs are important for channeling extracellular stimuli, including those related to many biotic and abiotic stresses, to the cytoplasm. Accordingly, we investigated the expression profiles of *SsCNGC*s in the sugarcane cultivar YT 99–66 roots under K^+^*-*deficient stress. Generally, low-K^+^ stimuli altered the expression of many *SsCNGC*s (Fig. 8a). The expression patterns of SsCNGC genes and their alleles always showed slight differences, such as *SsCNGC1* and *9*. Under low-K^+^ stress, the expression of *SsCNGC1*, *3*, *9* and *9-2D* was inhibited, while the expression of *SsCNGC1-1P*, *1-2C*, *3-3B* and *9-1* *T* was upregulated at different time points. The expression of *SsCNGC2* and *2-1P* was upregulated under K^+^ starvation except at 72 h after treatment, while *SsCNGC16* and its alleles presented the opposite expression trend. According to these results, a hypothesis is that *CNGC*s are involved in the sugarcane cultivar YT 99-66 response to low-K^+^ stress. In addition, *SsCNGC3*, *5* and *6*, as well as their alleles, were also rarely expressed in roots and were probably not regulated by K^+^ starvation (Supplementary Table 6). To validate the transcriptome data, RT-qPCR were performed to evaluate the expression patterns of 6 of these *SsCNGC*s with relative high-level of transcription (Supplementary Table 6). The results of RT-qPCR were largely consistent with the transcriptome data. For example, *SsCNGC7* exhibited lowest expression at 24 h under low-K^+^ treatment. Expression of *SsCNGC1* and *12* were significantly reduced at 72 h. *SsCNGC8* were down-regulated by low-K^+^ treatment with a restore at 48 h (Fig. 8b). We hypothesized that these three *SsCNGC*s might respond to other specific spatiotemporal conditions. The *SsCNGC*s exhibited varying expression patterns and likely play different roles in the sugarcane cultivar YT 99-66 response to low-K^+^ stress.

## Discussion

Sugarcane is an important sugar crop and a bioenergy source. It is therefore critical to understand the development and responses of sugarcane to environmental stimuli. Ca^2+^ is an essential second messenger that participates in plant responses to environmental stimuli and developmental cues. Stimulus-specific Ca^2+^ signaling is produced based on activating Ca^2+^-permeable channels [55, 56]. As a nonselective, ligand-gated cation channel, CNGCs have been identified across the plant kingdom [20]. CNGCs are permeable to Ca^2+^ and K^+^ and have been confirmed to be involved in plant development and responses to a variety of stresses [57]. However, genome-wide analysis of the CNGC gene family has not been conducted in *Saccharum* due to its complex genetic background. In this study, a total of 16 *CNGC* genes and 27 alleles were initially identified in the genome of *S. spontaneum* (Table 1). All these members of the CNGC family contained typical CNBD, CaMBD and IQ motifs (Fig. 1a). Similar to CNGCs in other plant species, members of the CNGC family in *S. spontaneum* were able to be categorized into 4 groups with divergence in distribution [20]. *SsCNGC*s in Groups II, III and IVa shared similar gene structures and patterns of conserved motifs but the same was not true for members of Groups I and IVb (Figs. 1 and 2). The conserved motifs in SsCNGCs may imply similar modes of interaction with their target proteins.

Membranes of CNGC gene family from *A. thaliana* and *S. spontaneum* share a relatively low amino acid identify (data not shown), and close alignment of most AtCNGCs was not identified in *S. spontaneum*, including *AtCNGC16* and *AtCNGC18* which were proved to be important for pollen development. However, based on the phylogenic tree, the SsCNGC1, 5, 6, and 12 were identified as the close alignments of ZmCNGC1, ZmCNGC5, OsCNGC4, OsCNGC5 and OsCNGC13 respectively (Fig. 2), which were predominantly involved in pollen development [44, 58]. What’s more, the probable pollen-preferred cis-acting regulatory, TCTTYCTCC and GCGGMGGCG [58], were identified in the promoter of *SsCNGC5* and *6* (Supplementary File 4). Accordingly, the SsCNGC5 and 6 are possible to form a homomeric complex like their close alignment, OsCNGC4 and OsCNGC5 [58], and involved in pollen development of sugarcane.*SsCNGC*s were unevenly dispersed across the 23 *S. spontaneum* chromosomes, and the number of genes on each chromosome ranged from 1 to 5 (Fig. 4). Gene duplication contributes to the expression of the gene family [59], and most *SsCNGC*s originated from segmental (67.4%) and dispersed (16.3%) duplication events (Fig. 4). It seems that the expansion of the CNGC family is closely related to the genome duplication of *S. spontaneum*. Synteny analysis also revealed that many SsCNGC genes are located in conserved syntenic blocks between rice and *S. spontaneum*. It is speculated that these SsCNGC genes are crucial for plant development [60, 61].Unlike CNGC genes in other species that arising from tandem duplications are mostly in Groups I and IVa [62], and the 4 *SsCNGC*s with tandem duplication events identified in this study are in Groups I and III (Figs. 2 and 4). Among the 4 tandem duplication SsCNGC gene pairs, *SsCNGC9-2D*/*SsCNGC9-1 T* and *SsCNGC10-3D*/*SsCNGC10-1 T* showed similar gene structures (Fig. 1) and expression patterns under normal conditions (Figs. 4, 6 and 7). For the other two pairs (*SsCNGC7-3D*/*SsCNGC7-1 T*, *SsCNGC1/SsCNGC3*), *SsCNGC7-1 T* and *SsCNGC3* were truncated (Fig. 1) and were rarely expressed in leaves, stems and roots (Supplementary Tables 4, 5 and 6). Genovariation always leads to the functional expansion of genes. This study revealed that the expression patterns of the 4 tandem duplication gene pairs under low-K^+^ conditions were different. This suggests that these genes may exercise different functions in the sugarcane response to low-K^+^ stress.

*SsCNGC1* showed higher expression levels in the stems and basal zone of leaves (Fig. 6 and Supplementary Table 4). The closest homologous genes of *SsCNGC1* in *Arabidopsis*, *AtCNGC3* and *10*, are involved in germination, hypocotyl elongation, Na^+^ and K^+^ uptake and homeostasis [63, 64]. It is speculated that *SsCNGC1* is critical for the regulation of stem elongation and Na^+^ and K^+^ homeostasis. As the homolog gene of *AtCNGC2* and *OsCNGC14*, *SsCNGC13* exhibited higher expression levels in maturing and mature stem tissues and the sink tissue of leaves and has been suggested to impact plant responses to thermal stress, chilling, and pathogens [28, 30, 64].

Researchers have identified the circadian regulation of the CNGC in chicken cone photoreceptors [65, 66]. Zia et al*.* also identified light-responsive cis-regulatory elements in almost CNGC genes in *Citrus recticulata* [67]. However, there have been few studies on the role of *CNGC*s in the plant circadian rhythm or in response to light intensity changes. In this study, light-responsive elements were found in protomers of all the *SsCNGC*s, and the expression of most of the S*sCNGC*s was regulated by circadian rhythm.

As cation channels, some CNGCs in plants have been confirmed to be K^+^-permeable channels, such as *AtCNGC2*, *AtCNGC3*, *AtCNGC4*, *AtCNGC10*, and so forth [19]. *AtCNGC3* and *AtCNGC10*, which have strong K^+^ permeation, are likely to be important for root K^+^ uptake [57, 68]. In this study, we found that the expression of *SsCNGC1*, *1-2C*, *8*, *8-2B*, *8-3C*, *8-4D*, *9-2D*, *12-2D* and *13-1P* was downregulated by low-K^+^ treatment at different levels. Among them, the downregulation of *SsCNGC1-2C* expression was just 6 h after low-K^+^ treatment (Fig. 8 and Supplementary Table 6). Regarding the diversity of gene structure and expression patterns (Figs. 1 and 8), it is speculated that *SsCNGC1* and *1-2C* play divergent roles in the sugarcane response to low-K^+^ stress. After treatment for 72 h, the expression of *SsCNGC13-1P* was upregulated to over threefold the normal level. *SsCNGC13-1P* is a homologous gene of *AtCNGC2* and may be another K^+^-permeable channel in sugarcane. Under low-K^+^ stress, *SsCNGC9-1 T* shared a similar gene structure and pattern of motifs but not a similar expression pattern. *SsCNGC9-1 T* showed increased expression after low-K^+^ treatment. *SsCNGC9* is the homologous gene of *OsCNGC9* that does not have obvious K^+^ conductance. Further studies need to be carried out to explore the roles of *SsCNGC*s in the sugarcane response to low-K^+^ stress. For *Saccharum* hybrid, *S. officinarum* was assumed to contribute to genetic background of high sugar content, and *S. spontaneum* contributed to the stress tolerance and pest and disease resistance [69]. It is possible that roles of SsCNGC genes in sugarcane response to low-K^+^ stress is universal in other hybrid cultivars. The results should provide some theoretical guidance to breeding of K^+^ high-efficiency sugarcane cultivars.

## Conclusions

Altogether, identify and systematic informatics analyses of 16 *CNGCs* and their alleles in *S*. *spontaneum* were carried out firstly, including phylogenetic, chromosome location, gene structure, pattern of conserved motifs, duplication, syntenic analyses, and *cis*-elements in promoter. Moreover, the expression profiles of *SsCNGCs* during development, circadian rhythm and under low-K^+^ stress were investigated. Many SsCNGCs were highly tissue-specific expression during *S*. *spontaneum* development, such as *SsCNGC1* and *13*. And light-responsive elements were found in the promoters of the expression of most *SsCNGC*s could be regulated by circadian rhythm. What’s more, the expression of *SsCNGC13* was also regulated by low-K^+^ treatment, it may participate in *S*. *spontaneum* development and response to low-K^+^ stress.

## Supplementary Information

**Additional file 1. **

**Additional file 2:** **Supplementary file 1.** Nucleotide sequences of the coding region of *SsCNGC*s and alleles.

**Additional file 3:** **Supplementary file 2.** Amino acid sequences of SsCNGCs and alleles.

**Additional file 4:** **Supplementary file 3.** Amino acid sequences of CNGCs from* Arabidopsis thaliana*, *Oryza sativa* and *Zea mays*.

**Additional file 5: Supplementary file 4.** Promoter sequences of *SsCNGC*s and alleles.

**Additional file 6:** **Supplementary Table 1.** Sequence of primers for RT-qPCR.

**Additional file 7:** **Supplementary Table 2.** Detailed physiological and biochemical information of SsCNGC genes and their alleles.

**Additional file 8:** **Supplementary Table 3.** The detailed distribution of *cis*-regulatory elements in the promoters of SsCNGCs.

**Additional file 9:** **Supplemental Table 4.** The expression pattern of *SsCNGC*s based on FPKM in different tissues at different stages.

**Additional file 10:** **Supplementary Table 5.** The expression pattern of *SsCNGC*s based on FPKM during the circadian rhythms.

**Additional file 11:** **Supplementary Table 6.** The expression pattern of SsCNGCs based on FPKM under low-K^+^ stress.

## Data Availability

All RNA-seq data of *S*. *spontaneum* were downloaded from the sugarcane database website (http://sugarcane.zhangjisenlab.cn/sgd/html/index.html). The *S. spontaneum* genome project was deposited into Genbank with accession numbers: QVOL00000000. The RNA-seq data of YT 99–66 is the original data in this study.
