# Supplementary figures and images for: Genome-wide identification and expression analysis of the cyclic nucleotide-gated ion channel (CNGC) gene family in Saccharum spontaneum
Source: BMC Genomics. 2023 May 25;24:281. doi: 10.1186/s12864-023-09307-3 (PMC10214738; doi:10.1186/s12864-023-09307-3)

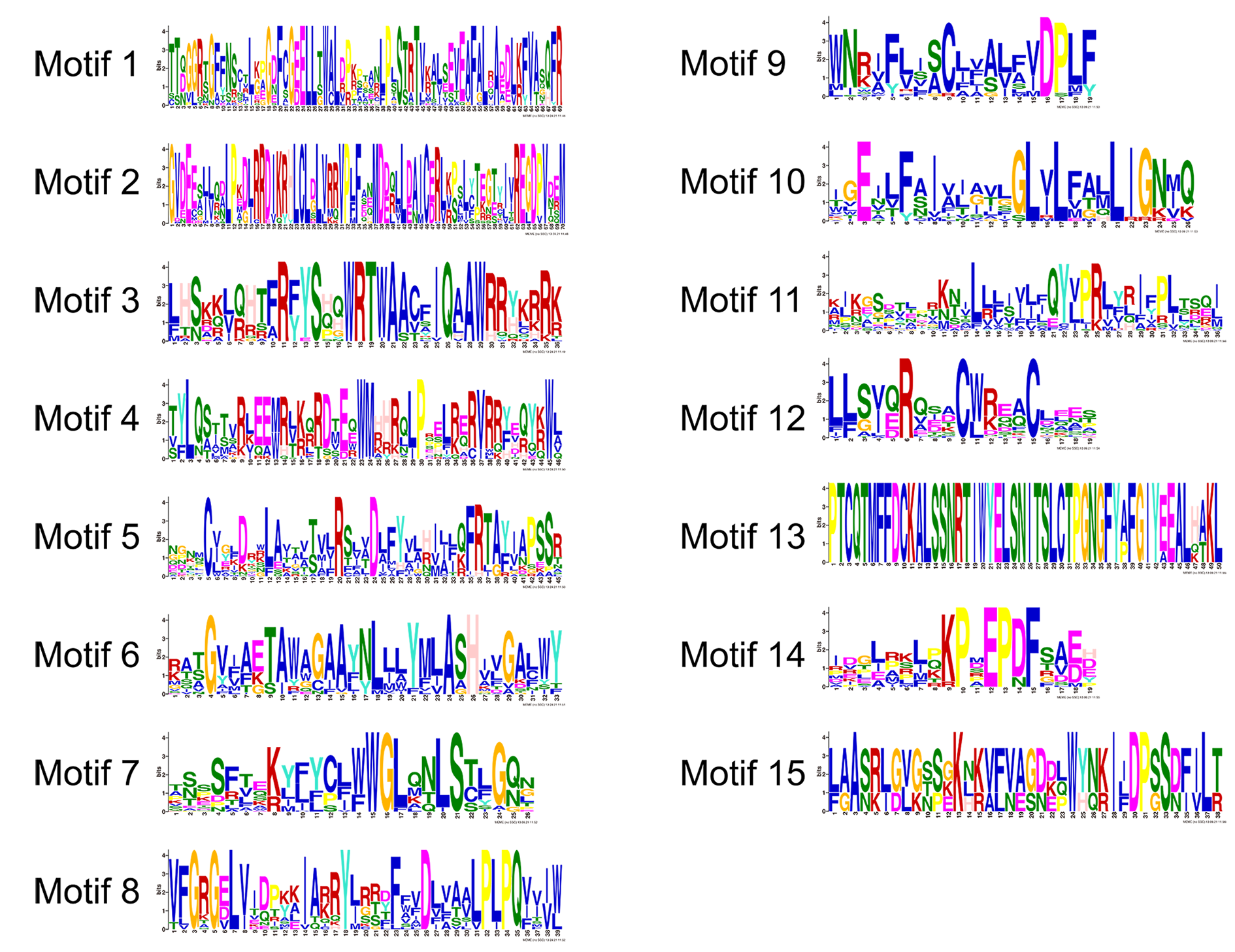

Supplement: Supplementary file 1 — Additional file 1. [file 12864_2023_9307_MOESM1_ESM.tif]
