# Supplementary material for: Genome-wide identification and expression analysis of the cyclic nucleotide-gated ion channel (CNGC) gene family in Saccharum spontaneum
Source: BMC Genomics. 2023 May 25;24:281. doi: 10.1186/s12864-023-09307-3 (PMC10214738; doi:10.1186/s12864-023-09307-3)
Supplement: Supplementary file 2 — Additional file 2: Supplementary file 1. Nucleotide sequences of the coding region of SsCNGCs and alleles. [file 12864_2023_9307_MOESM2_ESM.docx]

Supplementary file 1: Nucleotide sequences of the coding region of *SsCNGC*s and alleles.

>SsCNGC1|Sspon.04G0014070-1A

ATGTTGCGGAATGCCAAAATATCTTGGAATGGCAAACAAAACAGTAGGAAGCTGCATGGGGAAGAAGTCGGAGGAAAGGTTGAAGATTTGATCAGCTACTGCCTTCACTGCTGGCCTGTTCTCCTCTGGAGTTACAAGGAAGCCATGGCGGGCCGGGAGGAGAGATATGTGAGGGTAAATTCAGAAAGAGGGCATAACATCTTTGGCTTGTTAAAGGACAGAACTGCAGGAGCCTTTTCATTCCTGGGGAACTCTTCACATTCTGAAGCTCTAAACAAATCAGGCCCAGAGGAAAAGAAGTCCAAAACAAGAGTTCTTGATCCTCAAGGACCATTTTTGCAGAGATGGAACAAGATATTTGTGATATCATGTCTTTTTGCAGTTTTTGTGGACCCATTGTTCTTGTATATCCCAGTAATTGATGGTGGCAACAACTGCCTGTACTTGGACAAGAAGTTAGAGACCGTAGCAAGTATCCTGCGCTTTTTCACAGATATCTTCTATTTACTCCATATGCTATTTCAGTTCAGAACAGGCTTTATTGCTCCCTCTTCTAGAGTGTTCGGTCGGGGTGTCTTGGTTAAGGACACATTTGCAATAGCAAAGCGATATATATCAACACTGTTCCTGGTGGATTTGTTAGCAGTTCTGCCCCTCCCTCAGGTGTTTGTGTTGGTGGTGCTGCCTACTCTCCAAGGTCCTGAAGTTATGAAGGCAAAAAATGTACTACTGTATGTGCCTCGACTGCTCCGCATAATACCACTTTACCTTCAAATCACGAGATCTGCTGGCATACTTACAGAGACAGCATGGGCTGGTGCTGCTTTCAACCTCATAATTTATATGCTTGCCAGTCATGGCTTTGGAGCTCTTTGGTACATTCTTTCCATCCAGCGAGAAGACACCTGTTGGAGACAAGCATGTATCAATCAGACTGGCTGTGAGCTTACATCTTTATACTGTGGGTATCATCCACTTACAAATAATTCTTTCTTACAAAGTGCATGCCCAACAAATAGCACTTCCAATCCCAATCCAGACCCGAAATTTGGAATCTTTCTACCAGCTCTCCAAAATGTTTCACAATCAACGAGTTTCTTTGAAAAACTATTCTATTGCTTTTGGTGGGGCCTACAGAATCTAAGTTCCCTTGGCCAGAACATGAAAACAAGCACTAATACTTTGGAGAATCTGTTTGCTGTTTTTGTCTCGACATCGGGTTTGGTTCTATTTGCACTACTTATTGGTAATGTGCAGACCTATTTACAGTCAGCTTCTGTGCGTATAGAAGAAATGAGAGTGAAAAGGCGTGATACAGAGCAGTGGATGGCACATAGGTTACTCCCTGAGAATCTCAAGGATCGGATTATGCGCCATGAACAATACAGGTGGCAAGAAACAAGAGGGGTTGACGAAGAGGGCCTTCTTAAAAATCTTCCCAAGGATCTTAGAAGAGAGATAAAGAGACATCTTTGTTTGTCGCTTCTCATGAAGGTTCCAATGTTTGAAAACATGGATGAACAATTGTTGGATGCCATGTGTGATCGTCTGAAGCCTATGCTGTACACAGAAGGAAGCTGCATCATTCGCGAAGGTGACCCAGTGAATGAAATGCTCTTCATCATGAGAGGAACACTAGAGAGTACCACAACAAATGGTGGACAAACTGGCTTCTTCAACTCTAATGTTCTAAAAGGTGGAGACTTCTGTGGTGAAGAGCTCCTCACATGGGCCCTTGACCCCACTTCAGCTTCAAATCTTCCTGGCTCAACTAGGACAGTGAAGACGTTATCTGAAGTTGAAGCTTTTGCTCTGAGGGCTGATGACTTGAAGTTTGTTGCCACGCAATTCAGGAGGCTCCACAGCAAACAACTCCAACATACCTTCCGATTTTACTCACAGCAATGGAGGACCTGGGCTGCTTGCTTCATACAGGCAGCTTGGCACAGATACTGCAGAAAGAAGCTGGAAGAGGCTTTATATGAGAAGGAGAAGAGGTTACAAGCAGCAATTGTAAGTGACGGCACTACTTCGCTCAGTCTTGGTGCAGCGCTCTATGCTTCACGTTTTGCTGGCAACATGATGCGGATCTTACGGAGAAACGCCACCAGAAAGGCCCGTTTGCAGGAAAGAGTACCTGCAAGACTGTTACAAAAGCCAGCAGAACCCAACTTCTTCGCTGAAGATAGCTGA

>SsCNGC1-2C|Sspon.04G0014070-2C

ATGTTTGAAAACATGGATGAACAATTGTTGGATGCCATGTGTGATCGTCTGAAGCCTATGCTGTACACAGAAGGAAGCTGCATCATTCGCGAAGGTGACCCAGTGAATGAAATGCTCTTCATCATGAGAGGAACACTAGAGAGTACCACAACAAATGGTGGACAAACCGGCTTCTTCAACTCTAATGTTCTAAAAGGTGGAGACTTCTGTGGTGAAGAGCTCCTCACATGGGCCCTTGACCCCACTTCAGCTTCAAATCTTCCTGGCTCAACTAGGACAGTGAAGACGTTATCTGAAGTTGAAGCTTTTGCTCTGAGGGCTGATGACTTGAAGTTTGTTGCCACGCAATTCAGGAGGCTCCACAGCAAACAACTCCAACATACCTTCCGATTTTACTCACAGCAATGGAGGACCTGGGCTGCTTGCTTCATACAGGCAGCTTGGCACAGATACTGCAGAAAGAAGCTGGAAGAGGCTTTATATGAGAAGGAGAAGAGGTTACAAGCAGCAATTGTAAGTGACGGCACTACTTCGCTCAGTCTTGGTGCAGCGCTCTATGCTTCACGTTTTGCTGGCAACATGATGCGGATCTTACGGAGAAACGCCACCAGAAAGGCCCGTTTGCAGGAAAGAGTACCTGCAAGACTGTTACAAAAGCCAGCAGAACCCAACTTCTTCGCTGAAGATAGCTGA

>SsCNGC1-1P|Sspon.04G0014070-1P

ATGGCGGGCCGGGAGGAGAGATATGTGAGGGTAAATTCAGAAAGAGGGCATAACATCTTTGGCTTGTTAAAGGACAGAACTGCAGGAGCCTTTTCATTCCTGGGGAACTCTTCACATTCTGAAGCTCTAAACAAATCAGGCCCAGAGGAAAAGAAGTCCAAAACAAGAGTTCTTGATCCTCAAGGACCATTTTTGCAGAGATGGAACAAGATATTTGTGATATCATGTCTTTTTGCAGTTTTTGTGGACCCATTGTTCTTGTATATCCCAGTAATTGATGGTGGCAACAACTGCCTGTACTTGGACAAGAAGTTAGAGACCGTAGCAAGTATCCTGCGCTTTTTCACAGATATCTTCTATTTACTCCATATGCTATTTCAGTTCAGAACAGGCTTTATTGCTCCCTCTTCTAGAGTGTTCGGTCGGGGTGTCTTGGTTAAGGACACATTTGCAATAGCAAAGCGATATATATCAACACTGTTCCTGGTGGATTTGTTAGCAGTTCTGCCCCTCCCTCAGGTGTTTGTGTTGGTGGTGCTGCCTACTCTCCAAGGTCCTGAAGTTATGAAGGCAAAAATTTACTACTGGTTATTATTATTTTATGTGCCTCGACTGCTCCGCATAATACCACTTTACCTTCAAATCACGAGATCTGCTGGCATACTTACAGAGACAGCATGGGCTGGTGCTGCTTTCAACCTTATAATTTATATGCTTGCCAGTCATGGCTTTGGAGCTCTTTGGTACATTCTTTCCATCCAGCGAGAAGACACCTGTTGGAGACAAGCATGTATCAATCAGACTGGCTGTGAGCTTACATCTTTATACTGTGGGTATCATCCACTTACAAATAATTCTTTCTTACAAAGTGCATGCCCAACAAATAGCACTTCCAATCCCAATCCAGACCCGAAATTTGGAATCTTTCTACCAGCTCTCCAAAATGTTTCACAATCAACGAGTTTCTTTGAAAAACTATTCTATTGCTTTTGGTGGGGCCTACAGAATCTAAGTTCCCTTGGCCAGAACATGAAAACAAGCACTGATACTTTGGAGAATCTGTTTGCTGTTTTTGTCTCGACATCGGGTTTGGTTCTATTTGCACTACTTATTGGTAATGTGCAGACCTATTTACAGTCAGCTTCTGTGCGTATAGAAGAAATGAGAGTGAAAAGGCGTGATACAGAGCAGTGGATGGCACATAGGTTACTCCCTGAGAATCTCAAGGATCGGATTATGCGCCATGAACAATACAGGTGGCAAGAAACAAGAGGGGTTGACGAAGAGGGCCTTCTTAAAAATCTTCCCAAGGATCTTAGAAGAGAGATAAAGAGACATCTTTGTTTGTCGCTTCTCATGAAGGTTCCAATGTTTGAAAACATGGATGAACAATTGTTGGATGCCATGTGTGATCGTCTGAAGCCTATGCTGTACACAGAAGGAAGCTGCATCATTCGCGAAGGTGACCCAGTGAATGAAATGCTCTTCATCATGAGAGGAACACTAGAGAGTACCACAACAAATGGTGGACAAACCGGCTTCTTCAACTCTAATGTTCTAAAAGGTGGAGACTTCTGTGGTGAAGAGCTCCTCACATGGGCCCTTGACCCCACTTCAGCTTCAAATCTTCCTGGCTCAACTAGGACAGTGAAGACGTTATCTGAAGTTGAAGCTTTTGCTCTGAGGGCTGATGACTTGAAGTTTGTTGCCACGCAATTCAGGAGGCTCCACAGCAAACAACTCCAACATACCTTCCGATTTTACTCACAGCAATGGAGGACCTGGGCTGCTTGCTTCATACAGGCAGCTTGGCACAGATACTGCAGAAAGAAGCTGGAAGAGGCTTTATATGAGAAGGAGAAGAGGTTACAAGCAGCAATTGTAAGTGACGGCACTACTTCGCTCAGTCTTGGTGCAGCGCTCTATGCTTCACGTTTTGCTGGCAACATGATGCGGATCTTACGGAGAAACGCCACCAGAAAGGCCCGTTTGCAGGAAAGAGTACCTGCAAGACTGTTACAAAAGCCAGCAGAACCCAACTTCTTCGCTGAAGATAGCTGA

>SsCNGC2-1A|Sspon.08G0008040-1A

ATGCGGCGAAGACCCCGTGACCGTAAGCGCGAAGCTTCATCGCATTTCTCCCCAAACTCCACCCGCCTACCCAGCCCCGCCTCGAAGGGGAAAGGACGCGCCCGCGTCCCCAAGTCCAAACCCGAGCTAAATTTGAAGAAAATGATGATGGGAAGAGAGGACAAATATGTGAGATTTCAGGACTGGAGATCAGAGCAGTCTGTTAGTTCTGAGAACATAGTTGCTCCATATAGAGATGATGTTTCAGTATTCAGTTCACTTAAAGAAAGGACTGCCAGGGTTTTTGCATTGCTAGGAAATCTTTTGCACTCAGAAACCTCAAATAGATCAATGCTTGATGAAAGAAAGTCTGCAACAGGAACACTTCATCCTCAAGGGCCATTTCTGCAAAAATGGAACAGGATATTTGTGATATCATGTATATTTGCAGTTTCAGTGGATCCACTGTTCTTGTATATCCCAGTTATCAGTGATGAAAAACCTTGCTGGTATTTGGATAGAAAGTTGGAAAAGGCAGCAAGTGTCCTGCGTTTTTTCACAGATATTTTCTACATACTCCATATCATATTTCAGTTCCGGACAGGCTTTATTGCATCATCTCCTACAACCTTTGGGCGGGGTGTCTTAATCGAAGATAGATATGCAATAACAAAGCGCTACTTATCAACATATTTTTTCATTGATGTCTTTGCTATTCTACCCATCCCTCAGGTTATTATTTTGGTTCTGCTACCTAATCTTCAAGGCTTGAAGATTATGAAAGCCAAAAATGTGTTATTGCTTATAATTATATGCCAGTATGTGCCTCGGCTAATCCGGATAAGGCCACTATACCTCCAAATCACAAGGTCTGCTGGTGTAATTACAGAGACAGCACGGGCTGGTGCTGCTTTCAACCTTTTGCTTTACATGCTTGCCAGCCATAAGTTTGTAAAGAACAGCACCAGGGACAAACGAGGAATCCTACACCTGCAGAGTGCAGATTATGAACAAAGACCTGTCTCAGGCACTCAGACACAACCGAAAAGGTAG

>SsCNGC2|Sspon.08G0008040-1P

ATGCCGTTGCCGGGTCGTGCCGTCCTGCTGGCTGCTGCTGTGGAGTCCACGCCCTATCTTCCCCTCCCGCCACGTCCAGTCCACCCGCCTCATGCGGCGAAGACCGTGACCGTGAGCGCGAAGCTTCATCGCATTTCTCCCCAAACTCCACCCGCCTACCCAGCCCGCCTCGAAGGGGAAAGGACGCGCCCGCGTCCCCAAGTCCAAACCCGAGGAAGAAAGAAAGTGTGGCCGCACGCGGAGGGGTCAGGTGGCAATGAGTTTACAAAAGCTAAATTTGAAGAAAATGATGATGGGAAGAGAGGACAAATATGTGAGATTTCAGGACTGGAGATCAGAGCATCTGTTAGTTCTGAGAACATAGTTGCTCCATATAGAGATGATGTTTCAGTATTCAGTTCACTTAAAGAAAGGACTGCCAGGGTTTTTGCATTGCTAGGAAATCTTTTGCACTCAGAAACCTCAAATAGATCAATGCTTGATGAAAGAAAGTCTGCAACAGGAACACTTCATCCTCAAGGGCCATTTCTGCAAAAATGGAACAGGATATTTGTGATATCATGTATATTTGCAGTTTCAGTGGATCCACTGTTCTTGTATATCCCAGTTATCAGTGATGAAAAACCTTGCTGGTATTTGGATAGAAAGTTGGAAAAGGCAGCAAGTGTCCTGCGTTTTTTCACAGATATTTTCTACATACTCCATATCATATTTCAGTTCCGGACAGGCTTTATTGCATCATCTCCTACAACCTTTGGGCGGGGTGTCTTAATCGAAGATAGATATGCAATAACAAAGCGCTACTTATCAACATATTTTTTCATTGATGTCTTTGCTATTCTACCCATCCCTCAGGTTATTATTTTGGTTCTGCTACCTAATCTTCAAGGCTCGAAGATTATGAAAGCCAAAAATGTGTTATTGCTTATAATTATATGCCAGTATGTGCCTCGGCTAATCCGGATAAGGCCACTATACCTCCAAATCACAAGGTCTGCTGGTGTAATTACAGAGACAGCACGGGCTGGTGCTGCTTTCAACCTTTTGCTTTACATGCTTGCCAGCCATAAGTTTGTAAAGAACAGCACCAGGGACAAACGAGCAATCCTACACCTGCAGAGTGCAGATTATGAACAAAGACCTGTCCTTGGAGCTCTTTGGTACTTGCTTTCCATTCAACGCCAAGATTCCTGCTGGAGACAGCAGTGTAGAAGCAATCCGACATGTGATCTTGCATATTTGTACTGTGGAGATTATGATAACAATGTGAAAAATACTTTCTTAACTACAATTTGCCTACCAAGTAATCAGTCAAACCTTCCAGATCCGTACTTTGGGATTTATGCACCAGCTATAAAAAATGTATCACAGTCAAAAAGTTTCTTTGCGAAATTGTTCTTCTGTGTTTGGTGGGGTCTTCAAAATCTTAGCTCTCTTGGCCAAAACCTGAAAACAAGCACTTATGCATGGGAGAACTTATTCGCAGTTTTTGTCTCAATATCAGGCTTAGTTCTGTTTGCATTGCTGATCGGTAATGTGCAGACCTATTTGCAGTCAGCCTCTCTGAGAATAGAAGAAATGAGAGTTAAAAGCCGTGACACAGATCAGTGGATGTCATATCGACATCTTCCTGAGAATCTCAAGGAAAGAATACGGCGTTATGAACAGTATAGATGGCAAGAAACAAGCGGTGTTGATGAAGAGCAACTCCTTATGAACCTCCCCAAAGATCTTAGGAGGGATATAAAACGACATCTTTGTTTATCGCTTCTCATGAGGGTTCCATTGTTTGAAAATATGGACGATCAGCTTTTGGATGCCATGTGTGACTGCCTAAAGCCCATTCTATACACAGAAGGTAGCTGTGTTATTCGTGAAGGAGATCCGGTAAACGAAATGCTCTTCGTCATGAGGGGAAATCTAATGAGCATGACGACAAATGGTGGAAGAACTGGCTTCTTTAACTCTGATGTTCTGAAAGCTGGAGATTTCTGTGGTGAAGAGCTCCTCACCTGGGCTCTTGACCCCACATCAACATCCAGCCTCCCCAGCTCAACAAGGACAGTGAAGACAATGTCTGAAGTCGAAGCTTTTGCTTTGAGGGCTGAAGACTTAAGGTTTGTGGCAACTCAGTTCCGACGACTCCACAGCAAACAACTCCAGCACACTTTCAGATTCTACTCGCAGCAATGGAGAACCTGGGCTGCCTGTTTCATCCAGGCAGCCTGGCACCGGTACTGCAGAAAGAAGATTGAAGATTCTTTGCGTGAGAAGGAGAAGAGATTGCAATTCGCGATTGCCAACGACAGCTCCACTTCGCTCAGCTTCATGGCAGCACTATATGCTTCACGTTTCGCTGGAAACATGATACGGATCCTGAGGAGAAATGCCACGCGCAAGGCCAGGCTGCAGGAAAGAGTGCCTGCAAGACTGCTGCAGAAACCAGCAGAACCCAACTTTTCCGCAGAAGAGCAGTAA

>SsCNGC2-2B|Sspon.08G0008040-2B

TTTCAGGACTGGAGATCAGAGCAGTCTGTTAGTTCTGAGAACATAGTTGCTCCATATAGAGATGATGTTTCAGTATTCAGTTCACTTAAAGAAAGGACTGCCAGGGTTTTTGCATTGCTAGGAAATCTTTTGCACTCAGAAACCTCAAATAGATCAATGCTTGATGAAAGAAAGTCTGCAACAGGAACACTTCATCCTCAAGGGCCATTTCTGCAAAAATGGAACAGGATATTTGTGATATCATGTATATTTGCAGTTTCAGTGGATCCACTGTTCTTGTATATCCCAGTTATCAGTGATGAAAAACCTTGCTGGTATTTGGATAGAAAGTTGGAAAAGGCAGCAAGTGTCCTGCGTTTTTTCACAGATATTTTCTACATACTCCATATCATATTTCAGTTCCGGACAGGCTTTATTGCATCATCTCCTACAACCTTTGGGCGGGGTGTCTTAATCGAAGATAGATATGCAATAACAAAGCGCTACTTATCAACATATTTTTTCATTGATGTCTTTGCTATTCTACCCATCCCTCAGGTTATTATTTTGGTTCTGCTACCTAATCTTCAAGGCTCGAAGATTATGAAAGCCAAAAATGTGTTATTGCTTATAATTATATGCCAGTATGTGCCTCGGCTAATCCGGATAAGGCCACTATACCTCCAAATCACAAGGTCTGCTGGTGTAATTACAGAGACAGCACGGGCTGGTGCTGCTTTCAACCTTTTGCTTTACATGCTTGCCAGCCATAAGTTTGTAAAGAACAGCACCAGGGACAAACGAGAATCCTACACCTGCAGAGTGCAGATTATGAACAAAGACCTGTCTCAGGCACTCAGACACAACCGA

>SsCNGC3|Sspon.04G0014080-1A

ATGAAGGCCAGAGCAAGAGTTTTTGATCCCCGGGGGCCATTTCTAAAGAAATGGAACAAGATATTTGTGATATCATGCCTTGTTTCAGTCTCCGTGGACTCGCTCTTCTTCTACGCCCCGGCGATCGATGGCGACAACAGCTGCTTGTATCTGGATGACAATCTGCAAAAGATAGCTAGCATCCTGCGCTCTCTCACCGATGCCTTCTATTTACTCCGCGTAATATACCAGTTCAGGACAGGCTTCGCAGCTCCCTCATCTTCTGGAGCGTTTGGTCGCGGTGTCTTGGTTGATGACATGTTGGCAATAGCAAAGCGGTATCTATCGACATATTTTCTGATTGATATCTTATCTATTCTGCCCCTTCCTCAGACCTATTTACAATCAGCCTCTGGGCATATAGAGGAAATGAGAGTGATAAGACGTGACACAGAGCAATGGATGGCATACAGATTACTTCCAGAGCATATCAAGCAACGAATATTGCGTCATGATCAATATAGATGGCAAGAAACACAAGGTATGGATGAAGAGGGTCTTCTTATAAATCTTCCTAAGGATCTCAGGAGGGATATAAAGCGTCATCTTTGTCTATCACTTCTCATGAGGGTTCCAATGTTCGAAAACATGGATGATCAGCTCTTAGATGCCATGTGTGATCGTGTAAAACCGATGCTGTACACAGAAGGAAGCTGCATCATTCGCGAAGGCGACCCAGTCAACGAGATGTTCTTCATCATGAGAGGGAGACTTGAAAGCATGACAACAGACGGTGGGCGAACGGGCTTCTTCAACTCCAATGTTCTCCAAGGCGGCGATTTCTGCGGCGAAGAGCTCCTCACATGGGCTCTGGATCCTGCTTCGGGCTCAAACCTTCCCAGCTCAACCAGGACGGTGAAGACGCTGTCGGAGCTCGAAGGTTTCGCCTTGAGGGCTCATCACCTGAAGTTTGTGGCCAACCAGTACAGGAGGCTCCATAGCAAGCAGCTCCGGCATACCTTCAGATTTTACTCCCAGCAATGGCGTACGTGGGCTGCTTGCTTCATACAGGCAGCTTGGCACAGGTATTGCAGAAGGAAGATGGAGGATAGCCTGCATGAGAAGGAGAGGATGTTCCAAGCAGCAATCGTGACTGACGCCTCTAGCTCTTGCAGCCTTGGCGCGGCGCTCTACGCTGCCCATTTCGCTTCCAACATG

>SsCNGC3-2B|Sspon.04G0014080-2B

ATGAAGGCCAGAGCAAGAGTTCTTGATCCCCGGGGGCCATTTCTACAGAAATGGAACAAGATATTTGTGATATCATGCCTTGTTTCAGTCTCCGTGGACTCACTCTTCTTCTACGCCCCGGCGATCGATGGCGACAACAGCTGCCTGTATCTGGATGACAATCTGCAAAAGATAACCAGCATCCTGCGCTCTCTCACCGATGCCTTCTATTTACTCCGCGTAATATTCCAGTTCAGGACAGGCTTCGCAGCTCCCTCATCTTCTGGAGCGTTTGGTCGCGGTGTCTTGGTTGATGACATGTTGGCAATAGCAAAGCGGTCCTTGGAGCTCTTTGGTACATTCTTGCCATACAACGAGAAGACACCTGTTGGAGAGAAGCTTGTAATAACCAGGATGGTTGTGATCTGGCAACTTTATATTGTGGAAACCTATTTACAATCAGCCTCTGGGCATATAGAGGAAATGAGAGTGATAAGACGTGACACAGAGCAATGGATAGCATACAGATTACTTCCAGAGCATATCAAGCAACGAATATTGCGTCATGATCAATATAGATGGCAAGAAACACAAGGTGTGGATGAAGAGGGTCTTCTTATAAATCTTCCTAAGGATCTCAGGAGGGATATAAAGCGTCATCTTTGTCTATCACTTCTCATGAGGGTTCCAATGTTCGAAAACATGGACGATCAGCTCTTAGACGCCATGTGTGATCGCGTAAAACCCATGCTGTACACAGAAGGAAGCTGTATCATTCGCGAAGGCGACCCAGTCAATGAGATGTTCTTCATCATGAGAGGGAGACTTGAGAGCATGACAACAGACGGTGGGCGAACGGGCTTCTTCAACTCCAATGTTCTCCAAGGCGGCGATTTCTGCGGCGAAGAGCTCCTCACATGGGCTCTGGATCCTGCTTCGGGCTCAAACCTTCCCAGCTCAACCAGGACGGTGAAGACGCTGTCGGAGCTCGAAGGTTTCGCCTTGAGGGCTCATCACCTGAAGTTTGTGGCCAACCAGTACAGGAGGCTCCATAGCAAGCAGCTCCGGCATACCTTCAGATTTTACTCCCAGCAATGGCGTACGTGGGCTGCTTGCTTCATACAGGCAGCTTGGCACAGGTATTGCAGAAGGAAGCTGGAGGATAGCCTGCATGAGAAGGAGAGGATGTTCCAAGCAGCAATCGTGACTGACGCCTCTAGCTCTTGCAGCCTTGGCGCGGCGCTCTACGCTGCCCATTTCGCTTCCAACATG

>SsCNGC4|Sspon.05G0022360-1B

ATGCCCTTTGTCATCCGCTCTCCACCTCCATTGAGTCAGAGGCTGGAAGAGTCAAGTCCTAGGTCGTCTGTACCTTCTGAAGTGGGAGGCAGGAGTACCTTGAGGTCCAGCATGCCTGGGTTTGGTTATGGTTCATTTAATGCACTAAGGTCTTTCTTGTCAGGGGTCTGCAAAAGCTCTGGAAGACTGAAGTCACTTGGACAGTCACTTACTTCTGGTGCTCCTAAGACAGCTTTTGCGGAAGATCTTAAATCATATAAGAGAACTATATTTGATCCCCAGGACAAAATTCTTTTTCAAATGAACTGGGTTTTCTTCTCGTCTTGTCTTTTCGCTGTTGCAGTGGATCCACTATTCTTCTTTCTACCCATCATCAACGATTCAAACTGCATTGGTATAGATAAAAAGTTGGCAGTGACATCAACAATAATACGGACGGTTATTGATTTTGTCTACCTTATACGTGTGTGTCTTCAATTCCGCACTGCTTATGTGGCCCCGTCTTCACGAGTGTTTGGGACTGGTGAGCTTGTGATTGATCCAATGCTAATTGCAAAGCGATACATTAAAAGTTACTTTGCAATGGACTTTGTTGCATTGCTACCACTTCCACAGATTGTTGTTTGGAGATATCTCAACATTCCAGATGGCCCAGATGTACTGACTACAAAAACTGCACTGGTTTGGGTTGTTTTGATTCAGTACATTCCAAGGTTGTTTCGAATATTCCCTGTGACCACAGATTTGAAAAGGACAGCTGGTGTTTTTATTGAAACTGCTTGGGCTGGTGCTGCTTATTATCTTCTATGGTTTATGCTGGCTGGGCATAATGTCGGTACTTTATGGTACTTTTTAACCATAGAACGTGAAGATGATTGCTGGCATCTGTACTGTGATCCCAACGTTGGATGTAATAGCAGCTACTTGTATTGCAATAATAATCATCATGGCAGCTATGATAGTTGGCTTAAGACTAATGGTGCCCAAGTATTCAACATCTGCAATGGTACTCAAGATAATTTCAACTTTGGCATTTATCAGCAAGCACTGGTCTCTGGAATACTTCGTCCAGGAAATTTCATCTCAAAATTATGTTATTGCTTCTGGTGGGGATTGCAAAATCTAAGTACACTTGGTCAAGGGCTTTTGACAAGCACATACACTGGAGAAGTGATATTCTCTATAGCAATATGTGTCCTTGGACTAATTCTTTTTGCTCTCCTCATTGGTAACATGCAGAGCTACCTACAATCTGTTGCTATACGCCTTGAAGAGATGAGAGTTAAGAAACGTGATGCTGAGCAGTGGATGCATCACCGTTCACTGCCACCGGAAATCAGACATCGGGTGAGGAAGTATGAACGTTATCGGTGGTTGGAAACCAGGGGAGTAGATGAAGAAAGTTTGGTTCAAACTCTTCCGAAAGATCTTAGGAGGGATATCAAGCGTCATCTTTGTTTGGGCTTAGTGAAAAGGGTGCCTTTGTTTGAAAATATGGATGAACGATTATTGGATGCAATATGTGAGCGGTTAAGACCTGCACTCTACACAGAAAATGAATTCATTTTGAGAGAAGGTGATCCAGTGGATGAGATGCATTTTATTCTTCATGGTTGTTTGGAGAGTGTAACCACTGATGGTGGACGGAGTGGATTCTTTAACAAGGTCCAGCTAAAGGAAGGATCATTCTGTGGTGATGAGTTGCTCACTTGGGCATTGGATCCCAAGTCAGCTGCTAATTTTCCAGTTTCGAGCAGGACTGTCAAGGCACTCACCGAGGTTGAGGCGTTTGCCCTATGTGCAGAAGAGCTGAAATTCGTGGCCAGTCAGTTCAGGAGGCTGCACAGCAGGCAAGTACAGCACACGTTCCGATTCTATTCCCAGCAATGGAGGACCTGGGCAGCCTGCTTCATCCAAGCGGCATGGCGCCGCTATTACAAGAGGAAGATGGCAGAGCAGCGGCGCAAAGAAGAAGAGGCGGCAAGCCGGCCAAGTAGTAGCCACCCTAGCCTTGGGGCGACTATCTACGCATCTCGTTTCGCGGCCAACGCCATGCGAGGGGTTCACAGGCTTAGAAGCAAGGCTGTCCCTACCATTGTCAGGCTACCGAAACCCCCAGAACCAGATTTTGGTGTCGACGATGCTGACTAA

>SsCNGC4-2D|Sspon.05G0022360-2D

ATGGCAGTGCTGGAAGAGTCAAGTCCTAGGTCGTCTGTACCGTCTGAAGTGGGAGGCAGGAGTACCTTGAGGTCCAGCATGCCTGGGTTTGGTAGTTCATTTAATGCACTAAGGTCTTTCTTGTCAGGGGTCCGCAAAGGCTCTGGAAGACTGAAGTCACTTGGACAGTCACTTACTTCTGGTGCTCCTAAGACAGCTTTTGCAGAAGATCTTAAATCATATAAGAGAACTATATTTGATCCCCAGGACAAAATTCTTTTTCAAATGAACTGGGTTTTCTTCTCGTCTTGTCTTTTCGCTGTTGCAGTGGATCCACTATTCTTCTTTCTACCCATCATCAACGATTCAAACTGCATTGGTATAGATAAAAAGTTGGCAGTGACATCAACAATAATACGGACGGTTATTGATTTTGTCTACCTTATACGTGTGTGTCTTCAATTCCGCACTGCTTATGTTGCCCCGTCTTCACGAGTGTTTGGGACTGGTGAGCTTGTGATTGATCCAATGCTAATTGCAAAGCGATACATTAAAAGTTACTTTGCAATGGACTTTGTTGCATTGCTACCACTTCCACAGATTGTTGTTTGGAGATATCTCAACATTCCAGATGGCCCAGATGTACTGACTACAAAAACTGCACTGGTTTGGGTTGTTTTGATTCAGTACATTCCAAGGTTGTTTCGAATATTCCCTGTGATCACAGATTTGAAAAGGACAGCTGGTGTTTTTATTGAAACTGCTTGGGCTGGTGCTGCTTATTATCTTCTATGGTTTATGCTGGCTGGGCATAGCTACCTACAATCTGTTGCTATACGCCTTGAAGAGATGAGAGTTAAGAAACGTGATGCTGAGCAGTGGATGCATCACCGTTCACTGCCACCGGAAATGAGACATCGGGTGAGGAAGTATGAACGTTATCGGTGGTTGGAAACCAGGGGAGTAGATGAAGAAAGTTTGGTTCAAACTCTTCCGAAAGATCTTAGGAGGGATATCAAGCGTCATCTTTGTTTGGGCTTAGTGAAAAGGGTGCCTTTGTTTGAAAATATGGATGAACGATTATTGGATGCAATATGTGAGCGGTTAAGACCTGCACTCTACACAGAAAATGAATTCATTTTGAGAGAAGGTGATCCAGTGGATGAGATGCATTTTATTCTTCATGGTTGTTTGGAGAGTGTAACCACTGATGGTGGACGGAGTGGATTCTTTAACAAGGTCCAGCTAAAGGAAGGATCATTCTGTGGTGATGAGTTGCTCACTTGGGCATTGGATCCCAAGTCAGCTGCTAATTTTCCAGTTTCGAGCAGGACTGTCAAGGCACTCACCGAGGTTGAGGCGTTTGCCCTATGTGCAGAAGAGCTGAAATTCGTGGCCAGTCAGTTCAGGAGGCTGCACAGCAGGCAAGTACAGCACACGTTCCGATTCTATTCCCAGCAATGGAGGACCTGGGCAGCCTGCTTCATCCAAGCGGCATGGCGCCGCTATTACAAGAGGAAGATGGCAGAGCAGCGGCGCAAAGAAGAAGAGGCGGCAAGCCGTCCAAGTAGTAGCCACCCTAGCCTTGGGGCGACTATCTACGCATCTCGTTTCGCGGCCAACGCCATGCGAGGGGTTCACAGGCTAAGAAGCAAGGCTGTCCCTACCATTGTCAGGCTACCGAAACCCCCAGAACCAGATTTTGGTCTCGACGATGCTGACTAA

>SsCNGC5|Sspon.01G0024280-1A

ATGTCGTACGACCAGTCGGCTTTCCAGATGGACTACGTGGGCGTGGGCGCCGGCGCCGGCGTCGGCGTCAGCGCGTCCCGGCGGCGGTTCATGCCTTCGGAGTCGCTGGCCCGCGGCGTCATCACGCACGGGTCGGCGCAGCTGCGCACGATCGGGCGGTCGATCCGGGCCGGCGCCACCATGGCGGCCGTGTTCCAGGAGGACCTGAAGAACACCTCCCGGCGCATCTTCGACCCGCAGGACCCGGTGCTGGTGCGCCTCAACCGCGCCTTCCTCATCTCCTGCATCGTGGCCATCGCCGTGGACCCCATGTTCTTCTACCTGCCCATGGTCACCGACGAGGGCAACCTGTGCGTGGGCATCGACCGCTGGCTCGCCGTGTCCACCACCGTGGTGCGCAGCGTGGTGGACCTCTTCTTCGTGGGCCGCATCGCGCTGCAGTTCCGCACCGCCTACATCAAGCCGTCCTCCCGGGTGTTCGGGCGCGGCGAGCTGGTGATCGACACCGCGCTCATCGCGCGCCGGTACATGCGCCGCTTCTTCTCCGCCGACCTCGCGTCCGTGCTCCCGCTGCCGCAGGTGGTGATCTGGAAGTTCCTGCACCGGTCCAAGGGCACCGCCGTGCTGGACACCAAGAACAGCCTGCTCTTCATCGTCTTCATCCAGTACGTCCCGCGCGTGGTGCGCATCTACCCCATCTCCTCGGAGCTGAAGCGCACCAGCGGCGTGTTCGCCGAGACCGCCTACGCCGGCGCCGCCTACTACCTGCTGTGGTACATGCTGGCCAGCCACATCGTGGGCGCCTTCTGGTACCTGCTGTCCATCGAGCGCGTGAGCGACTGCTGGCGGAACGCGTGCGACGAGTTCCCCGGGTGCAACCAGATCTACATGTACTGCGGCAACGACCGGCACCTGGGGTTCCTGGAGTGGCGCACCATCACCCGGCAGCATCCCCCCTTCAACTACGGCATCTACTCGCCGGCCGTCACGTCGGACGTGCTCAAGACCAAGGACACCACCTCCAAGCTGCTCTTCTGCCTCTGGTGGGGGCTGGCCAACCTGAGCACCCTCGGGCAGGGGCTCAAGACCAGCATCTACACCGGGGAGGCGCTCTTCTCCATCGCGCTCGCCATCTTCGGCCTCATCCTCATGGCCATGCTCATCGGCAACATCCAGACGTACCTGCAGTCCCTCACCGTGCGCCTGGAGGAGATGCGCGTGAAGCAGCGCGACTCGGAGCAGTGGATGCACCACCGCCTGCTGCCGCCGGAGCTGCGCGAGCGCGTCCGCCGCTACGACCAGTACAAGTGGCTCAACACCCACGGCGTGGACGAGGAGGCGCTGGTGCAGAACCTGCCCAAGGACCTCCGCCGCGACATCAAGCGCCACCTCTGCCTCGGCCTCGTCCGCCGGGTGCCGCTCTTCGCCAACATGGACGAGCGCCTCCTGGACGCCATCTGCGAGCGCCTCAAGCCCAGCCTGTGCACGGAGCGCACCTACATCACCCGGGAGGGCGACCCCGTGGACCAGATGGTGTTCATCATCCGCGGCAGCCTGGAGAGCATCACCACCGACGGCGGGCGCACGGGGTTCTACAACCGCAGCCTGCTCGAGGAGGGGGACTTCTGCGGGGAGGAGCTGCTCACGTGGGCGCTCGACCCCAAGGCCGGCGCCTGCCTGCCGTCGTCCACGCGCACCGTCATGGCGCTCTCGGAGGTGGAGGCCTTCGCGCTGCACGCCGAGGAGCTCAAGTTCGTGGCGGGGCAGTTCCGCCGCATGCACAGCAAGGCGGTGCAGCACACGTTCCGGTTCTACTCCCAGCAGTGGCGCACGTGGGCGGCCACCTACATCCAGGCGGCGTGGCGGCGGCACCTCAAGCGCAGAGCGGCCGAGCTGCGGCGCAGGGAGGACGAGGAGATGGAGGAGGACGAAGGCAAGTCCAACAGAATCAGGACCACCATACTGGTGTCGCGGTTCGCGGCCAACGCCATGCGCGGCGTGCACCGGCAGCGCTCCAGGCGCGCCGTGGCCGTGCCCGTGCCCGTGCCCGAGCTCCTCATGCCCATGCCCAAGCCGCGGGAGCCCGACTTTCGCGACGACTACTAA

>SsCNGC5-2B|Sspon.01G0024280-2B

ATGTCGTACGACCAGTCGGCTTTCCAGATGGACTACGTGGGCGTGGGCGCCGGCGCCGGCGTCGGCGTCAGCGCGTCCCGGCGGCGGTTCATGCCTTCGGAGTCGCTGGCCCGCGGCGTCATCACGCACGGGTCGGCGCAGCTGCGCACGATCGGGCGGTCGATCCGGGCCGGCGCCACCATGGCGGCCGTGTTCCAGGAGGACCTGAAGAACACCTCCCGGCGCATCTTCGACCCGCAGGACCCGGTGCTGGTGCGCCTCAACCGCGCCTTCCTCATCTCCTGCATCGTGGCCATCGCCGTGGACCCCATGTTCTTCTACCTGCCCATGGTCACCGACGAGGGCAACCTGTGCGTGGGCATCGACCGCTGGCTCGCCGTGTCCACCACCGTGGTGCGCAGCGTGGTGGACCTCTTCTTCGTGGGCCGCATCGCGCTGCAGTTCCGCACCGCCTACATCAAGCCGTCCTCCCGGGTGTTCGGGCGCGGCGAGCTGGTGGTGATCTGGAAGTTCCTGCACCGGTCCAAGGGCACCGCCGTGCTGGACACCAAGAACAGCCTGCTCTTCATCGTCTTCATCCAGTACGTCCCGCGCGTGGTGCGCATCTACCCCATCTCCTCGGAGCTGAAGCGCACCAGCGGCGTGTTCGCCGAGACCGCCTACGCCGGCGCCGCCTACTACCTGCTGTGGTACATGCTGGCCAGCCACATCGTGGGCGCCTTCTGGTACCTGCTGTCCATCGAGCGCGTGAGCGACTGCTGGCGGAACGCGTGCGACGAGTTCCCCGGGTGCAACCAGATCTACATGTACTGCGGCAACGACCGGCACCTGGGGTTCCTGGAGTGGCGCACCATCACCCGGCAGGTGATCAACGAGACGTGCGAGCCCAAGCAGGACGGCAGCATCCCCCCCTTCAACTACGGCATCTACTCGCCGGCCGTCACGTCGGACGTGCTCAAGACCAAGGACACCACCTCCAAGCTGCTCTTCTGCCTCTGGTGGGGGCTGGCCAACCTGAGCACCCTCGGGCAGGGGCTCAAGACCAGCATCTACACCGGGGAGGCGCTCTTCTCCATCGCGCTCGCCATCTTCGGCCTCATCCTCATGGCCATGCTCATCGGCAACATCCAGACGTACCTGCAGTCCCTCACCGTGCGCCTGGAGGAGATGCGCGTGAAGCAGCGCGACTCGGAGCAGTGGATGCACCACCGCCTGCTGCCGCCGGAGCTGCGCGAGCGCGTCCGCCGCTACGACCAGTACAAGTGGCTCAACACCCACGGCGTGGACGAGGAGGCGCTGGTGCAGAACCTGCCCAAGGACCTCCGCCGCGACATCAAGCGCCACCTCTGCCTCGGCCTCGTCCGCCGGGTGCCGCTCTTCGCCAACATGGACGAGCGCCTCCTGGACGCCATCTGCGAGCGCCTCAAGCCCAGCCTGTGCACGGAGCGCACCTACATCACCCGGGAGGGCGACCCCGTGGACCAGATGGTGTTCATCATCCGCGGCAGCCTGGAGAGCATCACCACCGACGGCGGGCGCACGGGGTTCTACAACCGCAGCCTGCTCGAGGAGGGGGACTTCTGCGGGGAGGAGCTGCTCACGTGGGCGCTCGACCCCAAGGCCGGCGCCTGCCTGCCGTCGTCCACGCGCACCGTCATGGCGCTCTCGGAGGTGGAGGCCTTCGCGCTGCACGCCGAGGAGCTCAAGTTCGTGGCGGGGCAGTTCCGCCGCATGCACAGCAAGGCGGTGCAGCACACGTTCCGGTTCTACTCCCAGCAGTGGCGCACGTGGGCGGCCACCTACATCCAGGCGGCGTGGCGGCGGCACCTCAAGCGCAGAGCGGCCGAGCTGCGGCGCAGGGAGGACGAGGAGATAGAGGAGGACGAAGGCAAGTCCAACAGAATCAGGACCACCATACTGGTGTCGCGGTTCGCGGCCAACGCCATGCGCGGCGTGCACCGGCAGCGCTCCAGGCGCGCCGTGGCCGTGGCCGTGCCCGAGCTCCTCATGCCCATGCCCAAGCCGCGGGAGCCCGACTTTCGCGACGACTACTAA

>SsCNGC5-3C|Sspon.01G0024280-3C

ATGTCGTACGACCAGTCGGCTTTCCAGATGGACTACGTGGGCGTGGGCGCCGGCGCCGGCGTCGGCGTCAGCGCGTCCCGGCGGCGGTTCATGCCTTCGGAGTCGCTGGCCCGCGGCGTCATCACGCACGGGTCGGCGCAGCTGCGCACGATCGGGCGGTCGATCCGGGCCGGCGCCACCATGGCGGCCGTGTTCCAGGAGGACCTGAAGAACACCTCCCGGCGCATCTTCGACCCGCAGGACCCGGTGCTGGTGCGCCTCAACCGCGCCTTCCTCATCTCCTGCATCGTGGCCATCGCCGTGGACCCCATGTTCTTCTACCTGCCCATGGTCACCGACGAGGGCAACCTGTGCGTGGGCATCGACCGCTGGCTCGCCGTGTCCACCACCGTGGTGCGCAGCGTGGTGGACCTCTTCTTCGTGGGCCGCATCGCGCTGCAGTTCCGCACCGCCTACATCAAGCCGTCCTCCCGGGTGTTCGGGCGCGGCGAGCTGGTGATCGACACCGCGCTCATCGCGCGCCGGTACATGCGCCGCTTCTTCTCCGCCGACCTCGCGTCCGTGCTCCCGCTGCCGCAGGTGGTGATCTGGAAGTTCCTGCACCGGTCCAAGGGCACCGCCGTGCTGGACACCAAGAACAGCCTGCTCTTCATCGTCTTCATCCAGTACGTCCCGCGCGTGGTGCGCATCTACCCCATCTCCTCGGAGCTGAAGCGCACCAGCGGCGTGTTCGCCGAGACCGCCTACGCCGGCGCCGCCTACTACCTGCTGTGGTACATGCTGGCCAGCCACATCGTGGGCGCCTTCTGGTACCTGCTGTCCATCGAGCGCGTGAGCGACTGCTGGCGGAACGCGTGCGACGAGTTCCCCGGGTGCAACCAGATCTACATGTACTGCGGCAACGACCGGCACCTGGGGTTCCTGGAGTGGCGCACCATCACCCGGCAGGTGATCAACGAGACGTGCGAGCCCAAGCAGGACGGCAGCATCCCCTTCAACTACGGCATCTACTCGCCGGCCGTCACGTCGGACGTGCTCAAGACCAAGGACACCACCTCCAAGCTGCTCTTCTGCCTCTGGTGGGGGCTGGCCAACCTGAGCACCCTCGGGCAGGGGCTCAAGACCAGCATCTACACCGGGGAGGCGCTCTTCTCCATCGCGCTCGCCATCTTCGGCCTCATCCTCATGGCCATGCTCATCGGCAACATCCAGACGTACCTGCAGTCCCTCACCGTGCGCCTGGAGGAGATGCGCGTGAAGCAGCGCGACTCGGAGCAGTGGATGCACCACCGCCTGCTGCCGCCGGAGCTGCGCGAGCGCGTCCGCCGCTACGACCAGTACAAGTGGCTCAACACCCACGGCGTGGACGAGGAGGCGCTGGTGCAGAACCTGCCCAAGGACCTCCGCCGCGACATCAAGCGCCACCTCTGCCTCGGCCTCGTCCGCCGGGTGCCGCTCTTCGCCAACATGGACGAGCGCCTCCTGGACGCCATCTGCGAGCGCCTCAAGCCCAGCCTGTGCACGGAGCGCACCTACATCACCCGGGAGGGCGACCCCGTGGACCAGATGGTGTTCATCATCCGCGGCAGCCTGGAGAGCATCACCACCGACGGCGGGCGCACGGGGTTCTACAACCGCAGCCTGCTCGAGGAGGGGGACTTCTGCGGGGAGGAGCTGCTCACGTGGGCGCTCGACCCCAAGGCCGGCGCCTGCCTGCCGTCGTCCACGCGCACCGTCATGGCGCTCTCGGAGGTGGAGGCCTTCGCGCTGCACGCCGAGGAGCTCAAGTTCGTGGCGGGGCAGTTCCGCCGCATGCACAGCAAGGCGGTGCAGCACACGTTCCGGTTCTACTCCCAGCAGTGGCGCACGTGGGCGGCCACCTACATCCAGGCGGCGTGGCGGCGGCACCTCAAGCGCAGAGCGGCCGAGCTGCGGCGCAGGGAGGACGAGGAGATGGAGGAGGACGAAGGCAAGTCCAACAGAATCAGGACCACCATACTGGTGTCGCGGTTCGCGGCCAACGCCATGCGCGGCGTGCACCGGCAGCGCTCCAGGCGCGCCGTGCCCGAGCTCCTCATGCCCATGCCCAAGCCGCGGGAGCCCGACTTTCGCGACGACTACTAA

>SsCNGC5-4D|Sspon.01G0024280-4D

ATGTCGTACGACCAGTCGGCTTTCCAGATGGACTACGTGGGCGTGGGCGCCGGCGCCGGCGTCGGCGTCAGCGCGTCCCGGCGGCGGTTCATGCCTTCGGAGTCGCTGGCCCGCGGCGTCATCACGCACGGGTCGGCGCAGCTGCGCACGATCGGGCGGTCGATCCGGGCCGGCGCCACCATGGCGGCCGTGTTCCAGGAGGACCTGAAGAACACCTCCCGGCGCATCTTCGACCCGCAGGACCCGGTGCTGGTGCGCCTCAACCGCGCCTTCCTCATCTCCTGCATCGTGGCCATCGCCGTGGACCCCATGTTCTTCTACCTGCCCATGGTCACCGACGAGGGCAACCTGTGCGTGGGCATCGACCGCTGGCTCGCCGTGTCCACCACCGTGGTGCGCAGCGTGGTGGACCTCTTCTTCGTGGGCCGCATCGCGCTGCAGTTCCGCACCGCCTACATCAAGCCGTCCTCCCGGGTGTTCGGGCGCGGCGAGCTGGTGATCGACACCGCGCTCATCGCGCGCCGGTACATGCGCCGCTTCTTCTCCGCCGACCTCGCGTCCGTGCTCCCGCTGCCGCAGGTGGTGATCTGGAAGTTCCTGCACCGGTCCAAGGGCACCGCCGTGCTGGACACCAAGAACAGCCTGCTCTTCATCGTCTTCATCCAGTACGTCCCGCGCGTGGTGCGCATCTACCCCATCTCCTCGGAGCTGAAGCGCACCAGCGGCGTGTTCGCCGAGACCGCCTACGCCGGCGCCGCCTACTACCTGCTGTGGTACATGCTGGCCAGCCACATCGTGGGCGCCTTCTGGTACCTGCTGTCCATCGAGCGCGTGAGCGACTGCTGGCGGAACGCGTGCGACGAGTTCCCCGGGTGCAACCAGATCTACATGTACTGCGGCAACGACCGGCACCTGGGGTTCCTGGAGTGGCGCACCATCACCCGGCAGGTGATCAACGAGACGTGCGAGCCCAAGCAGGACGGCAGCATCCCCCCCTTCAACTACGGCATCTACTCGCCGGCCGTCACGTCGGACGTGCTCAAGACCAAGGACACCACCTCCAAGCTGCTCTTCTGCCTCTGGTGGGGGCTGGCCAACCTGAGCACCCTCGGGCAGGGGCTCAAGACCAGCATCTACACCGGGGAGGCGCTCTTCTCCATCGCGCTCGCCATCTTCGGCCTCATCCTCATGGCCATGCTCATCGGCAACATCCAGACGTACCTGCAGTCCCTCACCGTGCGCCTGGAGGAGATGCGCGTGAAGCAGCGCGACTCGGAGCAGTGGATGCACCACCGCCTGCTGCCGCCGGAGCTGCGCGAGCGCGTCCGCCGCTACGACCAGTACAAGTGGCTCAACACCCACGGCGTGGACGAGGAGGCGCTGGTGCAGAACCTGCCCAAGGACCTCCGCCGCGACATCAAGCGCCACCTCTGCCTGGGCCTCGTCCGCCGGGTGCCGCTCTTCGCCAACATGGACGAGCGCCTCCTGGACGCCATCTGCGAGCGCCTCAAGCCCAGCCTGTGCACGGAGCGCACCTACATCACCCGGGAGGGCGACCCCGTGGACCAGATGGTGTTCATCATCCGCGGCAGCCTGGAGAGCATCACCACCGACGGCGGGCGCACGGGGTTCTACAACCGCAGCCTGCTCGAGGAGGGGGACTTCTGCGGGGAGGAGCTGCTCACGTGGGCGCTCGACCCCAAGGCCGGCGCCTGCCTGCCGTCGTCCACGCGCACCGTCATGGCGCTCTCGGAGGTGGAGGCCTTCGCGCTGCACGCCGAGGAGCTCAAGTTCGTGGCGGGGCAGTTCCGCCGCATGCACAGCAAGGCGGTGCAGCACACGTTCCGGTTCTACTCCCAGCAGTGGCGCACGTGGGCGGCCACCTACATCCAGGCGGCGTGGCGGCGGCACCTCAAGCGCAGAGCGGCCGAGCTGCGGCGCAGGGAGGACGAGGAGATGGAGGAGGACGAAGGCAAGTCCAACAGAATCAGGACCACCATACTGGTGTCGCGGTTCGCGGCCAACGCCATGCGCGGCGTGCACCGGCAGCGCTCCAGGCGCGCCGTGCCCGAGCTCCTCATGCCCATGCCCAAGCCGCGGGAGCCCGACTTTCGCGACGACTACTAA

>SsCNGC6|Sspon.02G0031610-1A

ATGTCCGGGTGCGGCTACCGGACCCAGTTCATCAACGGGCGCAGGGAGAAGTTCGTGAGACTCGTCGAGGCAGACGAGCCGGCGGAGTCAGCGACCAGCCCCACTTCCGGCAGCGGCGGCGCCACCATGGAGCACGGCCACGGCAACGGCGGGGGCCACGGCGGCGGCGGCGGCGGGTTCCACATGGACAGCTACTTCTCGGGCAACCCGTCGGCTGCGGCGGCCAAGTTCCGCGCGCGGTCGGTGCGGGTGGCGGCGGGCGTGAAGAACCGGTCGGAGCGGCTGCGGAGCATCGGCCTTGTGTTCCAGGAGGACTTCCGGAAGATGTCCCAGCAGGTGTTCGACCCGCAGGACGCGTTCCTGGCGCGGATGAACCGCGCCTTCGTCTTCGCGTGCATCGTGTCCGTGGCCATCGACCCGCTCTTCCTCTACCTCCTGGCCGTCAAGTACACGGACAAGAACACCTGCATCGGCTTCGACCGCAACCTGGCCACCGTCGCCACCGTGGTGCGCACCGCCGTGGACGCCTTCTACCTGGCGCGGATCGCGCTGCAGTTCCGGACGGCGTACATCGCGCCGTCGTCGCGCGTGTTCGGGCGCGGCGAGCTGGTGATCGACTCGTCCGCCATCGCGCGCCGCTACCTGCGCCGCTTCTTCGTCGTGGACCTGCTGTCCGTGCTGCCGCTGCCGCAGGTCTCCATCTGGAACTTCCTCAACCGGCCCAAGGGCGCGGACCTGCTGCCCACCAAGAACGCGCTGCTCTTCACGGTGCTGTCGCAGTACGTGCCCCGCCTGGTGCGCTTCTACCCCATCACCTCGGAGCTGAAGCGCACCACGGGGGTGTTCGCCGAGACGGCCTTCGGCGGCGCCGCCTTCTACCTGCTGCTCTACATGCTGGCCAGCCACATGGTGGGGGCGTTCTGGTACCTGCTGGCCATCGAGCGGCTGGACGACTGCTGGCGGGACAAGTGCACCAAGCTCAACTTCCACCAGTGCCGCACCTACATGTACTGCGGCGGCGGCAGCCAGGGCCAGTCGGGCTTCCTCGAGTGGCGCACCATGATCCGGCAGGTGCTGGCGCAGGAGTGCGCCCCCGTCGACGGCAGCGGCACGGGGTTCCCCTACGGCATCTACACGACGGCGATCCAGTCCGGGGTCTACTCCACGGAGAACCTGACGGCCAAGATCCTCTTCTGCCTCTGGTGGGGGCTGCAGAACCTCAGCACCATCGGGCAGGGCCTGGAGACGACGCACTACAAGGGGGAGCAGCTCTTCTCCATCACGCTCGCGCTGCTGGGGCTCATCCTCATGGCGCTCCTCATCGGCAACATGCAGACGTACCTGCAGTCCATGACGCTGCGCCTGGAGGAGATGCGGCTCAAGCGCCGGGACTCGGAGCAGTGGATGCACCACCGCGTCCTCCCCGACGAGCTCCGGGAACGGGTGTGGCGGCACAACCAGTACAAGTGGCTCGAGACGCGCGGCGTCGACGAGGACAGCCTCGTGCGCAGCCTGCCCAAGGACCTCCGCCGCGACGTGAAGCGGCACCTCTGCCTCCGCCTCGTCCGCCGCGTCCCGCTCTTCGCCAACATGGACGAGCGGCTCCTCGACGCCATCTGCGAGCGCCTCAAGCCCAGCCTCTGCACCGAGTCCACCTACATCGTGCGCGAGGGGGACCCCGTCGACGAGATGCTCTTCATCATCCGGGGACGGCTCGAGAGCTCCACCACCGACGGCGGACGGATGGGGTTCTACAACCGGGGACTGCTCAAGGAGGGCGACTTCTGCGGCGAGGAGCTGCTCACGTGGGCGCTCGACCCCAAGGCCGGCACCAACTTCCCGCTCTCCACGCGCACCGTCAGGGCCATCTCCGAGGTGGAGGCGTTCGCGCTGCGCGCCGACGAGCTCAAGTTCGTGGCGGGGCAGTTCCGGCGCCTGCACAGCAAGCAGCTGCAGCAGACGTTCCGCTTCTACTCGCAGCAGTGGCGCACCTGGGCGTCCTGCTTAATACAGGCCGCATGGAGGAGGTACCTCAAGCGGAAGGCGGCCGAGCAGCGGCGCCGGGAGGAGGAGATGGAGGCCGACGAGGCCGCCGCGTCCGGGGTCTCCACCAGCCGGTTCAAGACCACGCTCCTCGTATCGCGCTTCGCCAAGAACGCCATGCGCGGCGTGCAGCGCCAGCGCTCCGTCCGAGCCGACAGCCTCATCATGCTGCCCAGGCCGCCGGAGCCGGACTTCGGCTCCATGGACTACTGA

>SsCNGC6-2D|Sspon.02G0031610-2D

ATGTTCGGGTGCGGCTACCGGACCCAGTTCATCAACGGGCGCAGGGAGAAGTTCGTGAGACTCGTCGAGGCAGACGCGCCGGCGGAGTCAGCGACCAGCCCCACTTCCGGCAGCGGCGGCGCCACCATGGAGCACGGCCACGGCAACGGCGGGGGCCACGGCGGCGGCGGCGGCGGGTTCCACATGGACAGCTACTTCTCGGGCAACCCGTCGGCTGCGGCGGCCAAGTTCCGCGCGCGGTCGGTACGTGTGGCGGCGGGCGTGATGAACCGGTCGGAGCGGCTGCGGAGCATCGGCCTGGTGTTCCAGGAGGACTTCCGGAAGATGTCCCAGCAGGTGTTCGACCCGCAGGACGCGTTCCTGGCGCGGATGAACCGCGCCTTCGTCTTCGCGTGCATCGTGTCCGTGGCCATCGACCCGCTCTTCCTCTACCTCCTGGCCGTCAAGTACACGGACAAGAACACCTGCATCGGCTTCGACCGCAACCTGGCCACCGTCGCCACCGTGGTGCGCACCGCCGTGGACGCCTTCTACCTGGCGCGGATCGCGCTGCAGTTCCGGACGGCGTACATCGCGCCGTCGTCGCGCGTGTTCGGGCGCGGCGAGCTGGTGATCGACTCGTCCGCCATCGCGCGCCGCTACCTGCGCCGCTTCTTCGTCGTGGACCTGCTGTCCGTGCTGCCGCTGCCGCAGGTCTCCATCTGGAACTTCCTCAACCGGCCCAAGGGCGCGGACCTGCTGCCCACCAAGAACGCGCTGCTCTTCACGGTGCTGTCGCAGTACGTGCCCCGCCTGGTGCGCTTCTACCCCATCACCTCGGAGCTGAAGCGCACCACGGGGGTGTTCGCCGAGACGGCCTTCGGCGGCGCCGCCTTCTACCTGCTGCTCTACATGCTGGCCAGCCACATGGTGGGGGCGTTCTGGTACCTGCTGGCCATCGAGCGGCTGGACGACTGCTGGCGGGACAAGTGCACCAAGCTCAACTTCCACCAGTGCCGCACCTACATGTACTGCGGCGGCGGCAGCCAGGGCCAGTCGGGCTTCCTCGAGTGGCGCACCATGATCCGGCAGGTGCTGGCGCAGGAGTGCGCCCCCGTCGACGGCAGCGGCACGGGGTTCCCCTACGGCATCTACACGACAGCGATCCAGTCCGGGGTCTACTCCACGGAGAACCTGACGGCCAAGATCCTCTTCTGCCTCTGGTGGGGGCTGCAGAACCTCAGCACCATCGGGCAGGGCCTGGAGACGACGCACTACAAGGGGGAGCAGCTCTTCTCCATCACGCTCGCGCTGCTGGGGCTCATCCTCATGGCGCTCCTCATCGGCAACATGCAGACGTACCTGCAGTCCATGACGCTGCGCCTGGAGGAGATGCGGCTCAAGCGCCGGGACTCGGAGCAGTGGATGCACCACCGCGTCCTCCCGGACGAGCTCCGGGAACGCGTGTGGCGGCACAACCAGTACAAGTGGCTCGAGACGCGCGGCGTCGACGAGGACAGCCTCGTGCGCAGCCTGCCCAAGGACCTCCGCCGCGACGTCAAGCGCCACCTCTGCCTCCGCCTCGTCCGCCGCGTCCCGCTCTTCGCCAACATGGACGAGCGGCTCCTCGACGCCATCTGCGAGCGCCTCAAGCCCAGCCTCTGCACCGAGTCCACCTACATCGTGCGCGAGGGGGACCCCGTCGACGAGATGCTCTTCATCATCCGGGGACGGCTCGAGAGCTCCACCACCGACGGCGGACGGATGGGGTTCTACAACCGGGGACTGCTCAAGGAGGGCGACTTCTGCGGCGAGGAGCTGCTCACGTGGGCGCTCGACCCCAAGGCCGGCACCAACTTCCCGCTCTCCACGCGCACCGTCAGGGCCATCTCCGAGGTGGAGGCGTTCGCGCTGCGCGCCGACGAGCTCAAGTTCGTGGCGGGGCAGTTCCGCCGCCTGCACAGCAAGCAGCTGCAGCAGACGTTCCGCTTCTACTCGCAGCAGTGGCGCACCTGGGCGTCCTGCTTAATACAGGCCGCATGGAGGAGGTACCTCAAACGGAAGGCGGCCGAGCAGCGGCGCCGGGAGGAGGAGATGGAGGCCGACGAGGCCGCCGCGTCCGGGGTCTCCACCAGCCGGTTCAAGACCACGCTCCTCGTATCGCGCTTCGCCAAGAACGCCATGCGCGGCGTGCAGCGCCAGCGCTCCGTCCGAGCCGACAGCCTCATCATGCTGCCCAGGCCGCCGGAGCCGGACTTCGGCTCCATGGACTACTGA

>SsCNGC7|Sspon.04G0002010-1A

ATGGACTGCGATTTGTTCGCTGCGTGGTGGAGCAGCAGCACCAGATTAGTCTCCAGGATTTTTCGGGGGTCAGCAGCTGATGCGCCGGGGCCATCGCCAATGAGGCCAGCCATACCGCTTCACCAGAAGCAGGCGGGGCTCGCTGCTAGCAAGCTAGGCGTGGGGACCTCGAAGAAGCACAGGGCTTTCGTCGCGAGCGATGAGCAGTGGTACAACAAGATATTTGACCCGTCGAGCGACTTCATCTTGACATGGAACCGCATCTTCCTCTTCTCCTGCTTCGTCGCGCTATTTATAGACCCTCTCTACTTCTATGTGCCCAAGATCAGCTACGGCAGCCCCAAATTCTGTGTCGGAACAGACACCCGTTTCGCCGTCGGTGTTACATTCTTCAGATCGATTGCCGATTTATTGTATGTCCTGCACATCATAATAAAGTTCAGAACAGCATATATCAACCCTAGCTCGACTCTGAGGGTGTTTGGAAGGGGAGATCTTGTCACAAATCCCAAGGAAATTGCGTGCAAATATATCAGATCTGACTTAGTTGTTGATGTGGCGGCTGCATTGCCTTTGCCACAGATTATTGTTTGGTTTGTGATACCAGCCATAAAGTATTCCTCTGCTGAGCACAACAATAACATTCTGGTGCTCATAGTTCTTGCTCAGTATCTTCCAAGATTGTATCTGATATTCCCCTTAACTTATGAAATTGTCAAAGCTACCGGAGTTGTTGCAAAGACTGCTTGGGAAGGGGCTGCATACAACATGGTGCTCTATCTGATAGCTAGTCATGTGCTAGGTGCACTATGGTATCTGCTATCTGTTGATCGCCAGACATTCTGCTGGAAGACGAGTTGCTTGAGTGAAACTGATTGTCATATTAAGTACCTAGATTGTGACACGACACTTAATGCTACTTGGGCGAGTACAACTGCTGTCTTCAGTAAATGTAATGCTAGCGATGACACTATTAGTTTTGATTTTGGTATGTTCGGGCCCGCGTTGTCCAATCAAGCCCCTGCTCAAAGTTTTGCAATGAAGTATTTCTATTCCCTCTGGTGGGGGTTGCAGAATTTAAGCTGCTATGGTCAGACTCTTAGTGTGAGTACCTATCTTGGTGAGACACTGTATTGTATATTCTTGGCGGTACTTGGTCTTGTCTTGTTTGCGCATTTGATTGGAAATGTGCAGACCTACCTGCAATCTATTACTGTGAGGGTTGAGGAATGGAGATTAAAGCAAAGAGATACTGAGGAATGGATGAGACATCGTCAACTTCCTTGTGAACTGCGGGAAAGGGTGAGACGATTTATCCAGTACAAGTGGCTTGCAACTAGGGGTGTGAACGAAGAGTCAATATTGCAAGCTCTGCCTGCAGACCTTCGACGTGACATTAAGCGCCACCTTTGCCTGGGTCTTGTTCGACGGGTTCCATTTTTCGCCCAGATGGATGATCAACTTCTTGATGCCATCTGTGAGCGTCTTGTATCATCACTGTGCACAAAAGGCACATACATTGTCCGTGAAGGCGATCCAGTGACTGAGATGCTCTTCATCATCCGTGGAAAACTGGAAAGCTCCACAACAAATGGTGGCCGCACTGGCTTCTTCAATTCAATTACCCTGAAACCTGGTGATTTCTGTGGTGAGGAACTTCTTGGATGGGCTCTTGTCCCCAGGCCTACTACAAACTTGCCATCATCCACTCGGACAGTGAAGGCACTGATAGAAGTGGAGGCCTTTGCACTCCAGGCTGAGGATCTCAAGTTTGTTGCCAGCCAGTTCAGGCGGCTGCACAGCAAGAAGTTGCAACACACTTTCCGGTATTACTCGCACCACTGGAGAACATGGGCCTCATGCTTCATCCAAGCTGCTTGGAGACGGTACAAGAGGAGGAAGATGGCAAAGGACCTGAGTATGAGGGAGTCATTCAATTCTGTCAGATTAGATGAAGTGGATAACGAAGATGACGATTCTCCACCCAAGAATAATCTTGCTCTCAAATTCATAGCTAGAACTAGAAAAGTGCCTCAGAACATGAAAGGGTTGCCTAAGCTAACAAAGCCAGATGAGCCAGATTTCTCAGCTGAACCTGAAGACTAG

>SsCNGC7-2B|Sspon.04G0002010-2B

ATGGACTGCGATTTGTTCGCTGCGTGGTGGAGCAGCAGCACCAGATTAGTCTCCAGGATTTTTCGGGGGTCAGCAGCTGATGCGCCGGGGCCATCGCCATTGAGGCCAGCCATACCGCTTCACCAGAAGCAGGCGGGGCTCGCTGCTAGCAAGCTAGGCGTGGGGACCTCGAAGAAGCACAGGGCTTTCGTCGCGAGCGATGAGCAGTGGTACAACAAGATATTTGACCCGTCGAGCGACTTCATCTTGACATGGAACCGCATCTTCCTCTTCTCCTGCTTCGTCGCGCTATTTATAGACCCTCTCTACTTCTATGTGCCCAAGATCAGCTACGGCAGCCCCAAATTCTGTGTCGGAACAGACACCCGTTTCGCCGTCGGTGTTACATTCTTCAGATCGATTGCCGATTTATTGTATGTCCTGCACATCATAATAAAGTTCAGAACAGCATATATCAACCCTAGCTCGACTCTGAGGGTGTTTGGAAGGGGAGATCTTGTCACAAATCCCAAGGAAATTGCGTGCAAATATATCAGATCTGACTTAGTTGTTGATGTGGCGGCTGCATTGCCTTTGCCACAGACCTACCTGCAATCTATTACTGTGAGGGTTGAGGAATGGAGATTAAAGCAAAGAGATACTGAGGAATGGATGAGACATCGTCAACTTCCTTGTGAACTGCGGGAAAGGGTGAGACGATTTATCCAGTACAAGTGGCTTGCAACTAGGGGTGTGAACGAAGAGTCAATATTGCAAGCTCTGCCTGCAGACCTTCGACGTGACATTAAGCGCCACCTTTGCCTGGGTCTTGTTCGACGGGTTCCATTTTTCGCCCAGATGGATGATCAACTTCTTGATGCCATCTGTGAGCGTCTTGTATCATCACTGTGCACAAAAGGCACATACATTGTCCGTGAAGGCGATCCAGTGACTGAGATGCTCTTCATCATCCGTGGAAAACTGGAAAGCTCCACAACAAATGGTGGCCGCACTGGCTTCTTCAATTCAATTACCCTGAAACCTGGTGATTTCTGTGGTGAGGAACTTCTTGGATGGGCTCTTGTCCCCAGGCCTACTACAAACTTGCCATCATCCACTCGGACAGTGAAGGCACTGATAGAAGTGGAGGCCTTTGCACTCCAGGCTGAGGATCTCAAGTTTGTTGCCAGCCAGTTCAGGCGGCTGCACAGCAAGAAGTTGCAACACACTTTCCGGTATTACTCGCACCACTGGAGAACATGGGCCTCATGCTTCATCCAAGCTGCTTGGAGACGGTACAAGAGGAGGAAGATGGCAAAGGACCTGAGTATGAGGGAGTCATTCAATTCTGTCAGATTAGATGAAGTGGATAACGAAGATGACGATTCTCCACCCAAGAATAATCTTGCTCTCAAATTCATAGCTAGAACTAGAAAAGTGCCTCAGAACATGAAAGGGTTGCCTAAGCTAACAAAGCCAGATGAGCCAGATTTCTCAGCTGAACCTGAAGACTAG

>SsCNGC7-3D|Sspon.04G0002010-3D

ATGGACTGCGATTTGTTCGCTGCGTGGTGGAGCAGCAGCACCAGATTAGTCTCCAGGATTTTTCGGGGGTCAGCAGCTGATGCGCCGGGGCCATCGCCAATGAGGCCAGCCATACCGCTTCACCAGAAGCAGGCGGGGCTCGCTGCTAGCAAGCTAGGCGTGGGGACCTCGAAGAAGCACAGGGCTTTTGTCGCGAGCGATGAGCAGTGGTACAACAAGATATTTGACCCGTCGAGCGACTTCATCTTGACATGGAACCGCATCTTCCTCTTCTCCTGCTTCGTCGCGCTATTTATAGACCCTCTCTACTTCTATGTGCCCAAGATCAGCTACGGCAGCCCCAAATTCTGTGTCGGAACAGACACCCGTTTCGCCGTCGGTGTTACATTCTTCAGATCGATTGCCGATTTATTGTATGTCCTGCACATCATAATAAAGTTCAGAACAGCATATATCAACCCTAGCTCGACTCTGAGGGTGTTTGGAAGGGGAGATCTTGTCACAAATCCCAAGGAAATTGCGTGCAAATATATCAGATCTGACTTAGTTGTTGATGTGGCGGCTGCATTGCCTTTGCCACAGATTATTGTTTGGTTTGTGATACCAGCCATAAAGTATTCCTCTGCTGAGCACAACAATAACATTCTGGTGCTCATAGTTCTTGCTCAGTATCTTCCAAGATTGTATCTGATATTCCCCTTAACTTATGAAATTGTCAAAGCTACCGGAGTTGTTGCAAAGACTGCTTGGGAAGGGGCTGCATACAACATGGTGCTCTATCTGATAGCTAGTCATGTGCTAGGTGCACTATGGTATCTGCTATCTGTTGATCGCCAGACATTCTGCTGGAAGACGAGTTGCTTGAGTGAAACTGATTGTCATATTAAGTACCTAGATTGTGACACGACACTTAATGCTACTTGGGCGAGTACAACTGCTGTCTTCAGTAAATGTAATGCTAGCGATGACACTATTAGTTTTGATTTTGGTATGTTCGGGCCCGCGTTGTCCAATCAAGCCCCTGCTCAAAGTTTTGCAATGAAGTATTTCTATTCCCTCTGGTGGGGGTTGCAGAATTTAAGCTGCTATGGTCAGACTCTTAGTGTGAGTACCTATCTTGGTGAGACACTGTATTGTATATTCTTGGCGGTACTTGGTCTTGTCTTGTTTGCGCATTTGATTGGAAATGTGCAGACCTACCTGCAATCTATTACTGTGAGGGTTGAGGAATGGAGATTAAAGCAAAGAGATACTGAGGAATGGATGAGACATCGTCAACTTCCTTGTGAACTGCGGGAAAGGGTGAGACGATTTATCCAGTACAAGTGGCTTGCAACTAGGGGTGTGAACGAAGAGTCAATATTGCAAGCTCTGCCTGCAGACCTTCGACGTGACATTAAGCGCCACCTTTGCCTGGGTCTTGTTCGACGGGTTCCATTTTTCGCCCAGATGGATGATCAACTTCTTGATGCCATCTGTGAGCGTCTTGTATCATCACTGTGCACAAAAGGCACATACATTGTCCGTGAAGGCGATCCAGTGACTGAGATGCTCTTCATCATCCGTGGAAAACTGGAAAGCTCCACAACAAATGGTGGCCGCACTGGCTTCTTCAATTCAATTACCCTGAAACCTGGTGATTTCTGTGGTGAGGAACTTCTTGGATGGGCTCTTGTCCCCAGGCCTACTACAAACTTGCCATCATCCACTCGGACAGTTAAGGCACTGATAGAAGTGGAGGCCTTTGCACTCCAGGCTGAGGATCTCAAGTTTGTTGCCAGCCAGTTCAGGCGGCTGCACAGCAAGAAGTTGCAACACACTTTCCGGTATTACTCGCACCACTGGAGAACATGGGCCTCATGCTTCATCCAAGCTGCTTGGAGATGGTACAAGAGGAGGAAGATGGCAAAGGACCTGAGTATGAGGGAGTCATTCAATTCTGTCAGATTAGATGAAGTGGATAACGAAGATGACGATTCTCCACCCAAGAATAATCTTGCTCTCAAATTCATAGCTAGAACTAGAAAAGTGCCTCAGAACATGAAAGGGTTGCCTAAGCTAACAAAGCCAAATGAGCCAGATTTCTCAGCTGAACCTGAAGACTAG

> SsCNGC7-1T|Sspon.04G0002010-1T

ATGGACTGCGATTTGTTCGCTGCGTGGTGGAGCAGCAGCACCAGATTAGTCTCCAGGATTTTTCGGGGGTCAGCAGCTGATGCGCCGGGGCCGTCGCCAATGAGGCCAGCCATACCGCTTCACCAGAAGCAGGCGGGGCTCGCTGCTAGCAAGCTAGGCGTGGGGACCTCGAAGAAGCACAGGGCTTTTGTCGCGAGCGATGAGCAGTGGTACAACAAGATATTTGACCCGTCGAGCGACTTCATCTTGACATGGAACCGCATCTTCCTCTTCTCCTGCTTCGTCGCGCTATTTATAGACCCTCTCTACTTCTATGTGCCCAAGATCAGCTACGGCAGCCCCAAATTCTGTGTCGGAACAGACACCCGTTTCGCCGTC

>SsCNGC8|Sspon.08G0012670-1A

ATGAGGTTCGGCTCCGGGAGGGTGGAGGACGAGATGGCGCTCACGAGGCAGAGGACCGTGAGATTCCATGATGAGAGGGCAAAGGCAACTATACCCATTCACCATAAGCAGCATGGGCTGGCTGCTAGCAGGCTCGGCTTGGGAAGTTCAGGGAAAAACAAGGTCTTTGTGGCAGGAGATGACTTGTGGTACAACAAGATTATTGACCCGTCAAGCGACTTCATCTTGACATGGATATACGTCTTCCATGTGTCATGCTTCATTGCTCTGCTCATGGACCCTCTGTATTTCTATGTGCCTGAAATCGATTACAGGCAAACCACTCATTGTGTCAGGAAGGATAGACGCCTAGCCATCATCGTTACTGTATTCCGATCAATTGCCGACCTCTTTTATGTCATCCAGATGATAATTAAGTTCAGGACTGCATACCTTAATCCAAGCTCAAACTTAGGGGTTTTTGGTCGAGGAGATCTCATCACAGATCCTAAGGAAATTGCAAAGCAGTACTTGAGATCTGACTTTGCAGTTGATTTGGTGGCTTCTTTGCCTTTACCACAGATCATTGTTTGGTCTGTGATACCAGCTATCAAATATTCTTCGTCTGAGCATGGCAATGACATGCTGCTTCTGGTTGCTCTTTTCCAGTATATCCTAAGATTATACCTCATCTTTTCCTTGAATGATAAAATAGTCAAAATCACTGGAGCTTTTGCAAAGACTGCTTGGCAAGGAGCTGCATACAATCTGCTGTTATACATGATTGCTAGCCATGTTTTAGGAGCGCTGTGGTACTTTCTATCTGTTGATCGCCAGATCGCTTGCTGGAAAAGTTTTTGCAATGAAAATGATTGCCACAGTCGGTATCTGTATTGTGATGTAAAACCAGATTCGAGTTGGAATGGGACCTTAGTCTTTTCTAGTTGTGATGCTAAGAACACTAACAAATTTGACTTTGGTATGTTCCAGCCATTGTTATCAAACAAAACTCCTAATGAGAGCTTCCTGAAGAAGTATATCTATTGCCTTTGGTGGGGCTTGCAGAATCTAAGTTGCTATGGCCAGACATTGAATGTGAGCACCTTTATTGGTGAGACACTGTATGCTATACTCTTGGCAGTGGTTGGTCTAGTCTTGTTTGCACATCTGATTGGAAAAGTTCAGACCTACCTGCAATCAATCACCGCTAGGGTTGAGGAGTGGAGGCTAAAGCAGAGAGATACTGAGGAGTGGATGAGACATAGGCAACTGCCCCATGAACTACGGGAAAGAGTGAGAAGATTTGTTCATTACAAGTGGCTCGCAACTCGAGGTGTGGATGAAGAATCTATATTGAATGCCCTACCTACAGATCTTTGCCGTGACATCAAGCGCCACCTTTGCCTGGATCTCGTTCGTAGGGTCCCACTTTTCTCCCAGATGGACGATCAACTTCTAGACGCCATATGTGAGCGTCTTGTATCTTCCTTGAGCACAGAGGGCACATATATTGTCCGTGAAGGCGACCCAGTGACTGAGATGTTGTTCATCATTCGTGGTAAGCTGGAAAGCTCCACCACAGATGGCGGCCGAACAGGCTTCTTCAATTCAATCACCCTCAAACCTGGTGATTTCTGTGGCGAAGAGCTCCTCGGATGGGCTCTTGTTCCCAAGCCTACCGTCAACCTGCCGTTATCCACTCGGACAGTGAAGGCGATTGTCGAAGTTGAGGCATTTGCTCTCCAAGCTGATGATCTCAGGTTCGTCGCCAGCCAATTCAGGCGCCTCCACAGCAGGAAGCTGCAGCACACGTTCCGATACTACTCTCACCACTGGAGGACCTGGGCCGCGTGCTTCATCCAGCACGCTTGGCGCCGCCAGAAAAGGAGGAAGATGGCCAAGGACCTGAGTATGAGGGAGTCGTTCTCCTCCATGAGATCATACGAAGGCGACAACTCCCCTGAACAGAATCTCACGCTTAGAAGAGGAGCCAGCATCATCAGAGAACTGCCTAAGTTCAGGAAACCATCGGAGCCAGATTTCTCAGCAGAGCATGACGACTGA

>SsCNGC8-2B|Sspon.08G0012670-2B

ATGAGGTTCGGCTCCGGGAGGGTGGAGGACGAGATGGCGCTCACGAGGCAGAGGACCGTGAGATTCCATGATGAGAGGGCAAAGGCAACTATACCCATTCACCATAAGCAGCATGGGCTGGCTGCTAGCAGGCTCGGCTTGGGAAGTTCAGGGAAAAACAAGGTCTTTGTGGCAGGAGATGACTTGTGGTACAACAAGATTATTGACCCGTCAAGCGACTTCATCTTGACATGGATATGCGTCTTCCGTGTGTCATGCTTCATTGCTCTGCTCATGGACCCTCTGTATTTCTATGTGCCTGAAATCGATTACAGGCAAACCACTCATTGTGTCAGGAAGGATATACGCCTAGCCATCATCGTTACTGTATTCCGATCAATTGTCGACCTCTTTTATGTCATCCAGATGATAATTAAGTTCAGGACTGCATACCTTAATCCAAGCTCAAACTTAGGGGTTTTTGGTCGAGGAGATCTCATCACAGATCCTAAGGAAATCGCAAAGCAGTACTTGAGATCTGACTTTGCAGTTGATTTGGTGGCTTCTTTGCCTTTACCACAGATCATTGTTTGGTCTGTGATACCAGCTATCAAATATTCTTCGTCTGAGCATGGCAATGACATGCTGCTTCTGGTTGCTTTTTTCCAGTATATCCTAAGATTATACCTCATCTTTTCCTTGAATGATAAAATAGTCAAAATTACTGGAGCTTTTGCAAAGACTGCTTGGCAAGGAGCTGCATACAATCTGCTGTTATACATGATTGCTAGCCATGTTTTAGGAGCGCTGTGGTACCTTCTATCTGTTGATCGCCAGATCGCTTGCTGGAAAAGTTTTTGCAATGAAACTGAAACTGATTGCCACACTCAGTATCTGTATTGTGATGTCAAACCAGATTCGAGTTGGAATGGGACCTTAGTATTTTCTAGTTGTGATGCTAAGAACACTAACAAATTTGACTTTGGTATGTTCCAGCCATTGTTATCAAACAAAACTCCTAATGAGAGCTTCCTGAAGAAGTATATCTATTGCCTTTGGTGGGGCTTGCAGAATCTAAGCACCTTTATTGGTGAGACACTGTATGCTATACTCTTGGCAGTGGTTGGTCTAGTCTTGTTTGCACATCTGATTGGAAAAGTTCAGACCTATCTGCAATCAATCACTGCTAGGGTTGAGGAGTGGAGGCTAAAGCAGAGAGATACTGAGGAGTGGATGAGACATAGGCAACTGCCCCATGAACTACGGGAAAGAGTGAGAAGATTTGTTCATTACAAGTGGCTCGCAACTCGAGGTGTGGATGAAGAATCTATATTGAATGCCCTACCTACAGATCTTCGCCGTGACATCAAGCGCCACCTTTGCCTGGATCTCGTTCGTAGGGTCCCACTTTTCTCCCAGATGGACGATCAACTTCTAGACGCCATATGTGAGCGTCTTGTATCTTCCTTGAGCACAGAGGGCACATATATTGTCCGTGAAGGCGACCCAGTGACTGAGATGTTGTTCATCATTCGTGGTAAGCTGGAAAGCTCCACCACAGATGGCGGCCGAACAGGTTTCTTCAATTCAATCACCCTCAAACCTGGTGATTTCTGTGGCGAAGAGCTCCTCGGATGGGCTCTTGTTCCCAAGCCTACCGTCAACCTGCCGTTATCCACTCGGACAGTGAAGGCGATTGTCGAAGTTGAGGCATTTGCTCTCCAAGCTGATGATCTCAGGTTCGTCGCCAGCCAATTCAGGCGCCTCCACAGCAGGAAGCTGCAGCACACGTTCCGATACTACTCTCACCACTGGAGGACCTGGGCCGCGTGCTTCATCCAGCACGCTTGGCGCCGCCAGAAAAGGAGGAAGATGGCCAAGGACCTGAGTATGAGGGAGTCGTTCTCCTCCATGAGATCATACGAAGGCGACAACTCCCCTGAACAGAATCTCGCGCTTAGAAGAGGAGCCAGCATCATCAGAGAACTGCCTAAGTTCAAGAAACCATCGGAGCCAGATTTCTCAGCAGAGCATGATGACTGA

>SsCNGC8-3C|Sspon.08G0012670-3C

ATGAGGTTCGGCTCCGGGAGGGTGGAGGACGAGATGGCGCTCACGAGGCAGAGGACCGTGAGATTCCATGACGAGAGGGCAAAGGCAACTATACCCATTCACCATAAGCAGCATGGGCTGGCTGCTAGCAGGCTCGGCTTGGGAAGTTCAGGGAAAAACAAGGTCTTTGTGGCAGGAGATGACTTGTGGTACAACAAGATTATTGACCCGTCAAGCGACTTCATCTTGACATGGATATGCGTCTTCCGTGTGTCATGCTTCATTGCTCTGCTCATGGACCCTCTGTATTTCTATGTGCCTGAAATCGATTACAGGCAAACCACTCATTGTGTCAGGAAGGATATACGCCTAGCCATCATCGTTACTGTATTCCGATCAATTGTCGACCTCTTTTATGTCATCCAGATGATAATTAAGTTCAGGACTGCATACCTTAATCCAAGCTCAAACTTAGGGGTTTTTGGTCGAGGAGATCTCATCACAGATCCTAAGGAAATCGCAAAGCAGTACTTGAGATCTGACTTTGCAGTTGATTTGGTGGCTTCTTTGCCTTTACCACAGATCATTGTTTGGTCTGTGATACCAGCTATCAAATATTCTTCGTCTGAGCATGGCAATGACATGCTGCTTCTGACCTACCTGCAATCAATCACTGCTAGGGTTGAGGAGTGGAGGCTAAAGCAGACAGATACTGAGGAGTGGATGAGACATAGGCAACTGCCCCATGAACTACGGGAAAGAGTGAGAAGATTTGTTCATTACAAGTGGCTCGCAACTCGAGGTGTGGATGAAGAATCTATATTGAATGCCCTACCTACAGATCTTCGCCGTGACATCAAGCGCCACCTTTGCCTGGATCTCGTTCGTAGGGTCCCACTTTTCTCCCAGATGGACGATCAACTTCTAGACGCCATATGTGAGCGTCTTGTATCTTCCTTGAGCACAGAGGGCACATATATTGTCCGTGAAGGCGACCCAGTGACTGAGATGTTGTTCATCATTCGTGGTAAGCTGGAAAGCTCCACCACAGATGGCGGCCGAACAGGTTTCTTCAATTCAATCACCCTCAAACCTGGTGATTTCTGTGGCGAAGAGCTCCTCGGATGGGCTCTTGTTCCCAAGCTTACCGTCAACCTGCCGTTATCCACTCGGACAGTGAAGACGATTGTCGAAGTTGAGGCATTTGCTCTCCAAGCTGATGATCTCAGGTTCGTCGCCAGCCAATTCAGGCGCCTCCACAGCAGGAAGCTGCAGCACACGTTCCGATACTACTCTCACCACTGGAGGACCTGGGCCGCGTGCTTCATCCAGCACGCTTGGCGCCGCCAGAAAAGGAGGAAGATGGCCAAGGACCTGAGTATGAGGGAGTCGTTCTCCTCCATGAGATCATACGAAGGCGACAACTCCCCTGAACAGAATCTCACGCTTAGAAGAGGAGCCAGCATCATCAGAGAACTGCCTAAGTTCAGGAAACCATCGGAGCCGGATTTCTCAGCAGAGCATGATGACTGA

>SsCNGC8-4D|Sspon.08G0012670-4D

ATGAGGTTCGGCTCCGGGAGGGTGGAGGACGAGATGGCGCTCACGAGGCAGAGGACCGTGAGATTCCATGATGAGAGGGCAAAGGCAACTATACCCATTCACCATAAGCAGCATGGGCTGGCTGCTAGCAGGCTCGGCTTGGGAAGTTCAGGGAAAAACAAGGTCTTTGTGGCAGGAGATGACTTGTGGTACAACAAGATTATTGACCCGTCAAGCGACTTCATCTTGACATGGATATACGTCTTCCATGTGTCATGCTTCATTGCTCTGCTCATGGACCCTCTGTATTTCTATGTGCCTGAAATCGATTACAGGCAAACCACTCATTGTGTCAGGAAGGATAGACGCCTAGCCATCATCGTTACTGTATTCCGATCAATTGCCGACCTCTTTTATGTCATCCAGATGATAATTAAGTTCAGGACTGCATACCTTAATCCAAGCTCAAACTTAGGGGTCTTTGGTCGAGGAGATCTCATCACAGATCCTAAGGAAATTGCAAAGCAGTACTTGAGATCTGACTTTGCAGTTGATTTGGTGGCTTCTTTGCCTTTACCACAGATCATTGTTTGGTCTGTGATACCAGCTATCAAATATTCATCGTCTGAGCATGGCAATGATATGCTGCTTCTGGTTGCTCTTTTCCAGTATATCCTAAGATTATACCTCATCTTTTCCTTGAATGATAAAATAGTCAAAATCACTGGAGCTTTTGCAAAGACTGCTTGGCAAGGAGCTGCATACAATCTGCTGTTATACATGATTGCTAGCCATGTTTTAGGAGCGCTGTGGTACTTTCTATCTGTTGATCGCCAGATCGCTTGCTGGAAAAGTTTTTGCAATGAAAATGATTGCCACACTCGGTATCTGTATTGTGATGTAAAACCAGATTCGAGTTGGAATGGGACCTTAGTCTTTTCTAGTTGTGATGCTAAGAACACTAACAGATTTGACTTTGGTATGTTCCAGCCATTGTTATCAAACAAAACTCCTAATGAGAGCTTCCTGAAGAAGTATATCTATTGCCTTTGGTGGGGCTTGCAGAATCTAAGTTGCTATGGCCAGACATTGAATGTGAGCACCTTTATTGGTGAGACACTGTATGCTATACTCTTGGCAGTGGTTGGTCTAGTCTTGTTTGCACATCTGATTGGAAAAGTTCAGACCTACCTGCAATCAATCACCGCTAGGGTTGAGGAGTGGAGGCTAAAGCAGAGAGATACTGAGGAGTGGATGAGACATAGGCAACTGCCCCATGAACTACGGGAAAGAGTGAGAAGATTTGTTCATTACAAGTGGCTCGCAACTCGAGGTGTGGATGAAGAATCTATATTGAATGCCCTACCTACAGATCTTTGCCGTGACATCAAGCGCCACCTTTGCCTGGATCTCGTTCGTAGGGTCCCACTTTTCTCCCAGATGGACGATCAACTTCTAGACGCCATATGTGAGCGTCTTGTATCTTCCTTGAGCACAGAGGGCACATATATTGTCCGTGAAGGCGACCCAGTGACTGAGATGTTGTTCATCATTCGTGGTAAGCTGGAAAGCTCCACCACAGATGGCGGCCGAACAGGCTTCTTCAATTCAATCACCCTCAAACCTGGTGATTTCTGTGGCGAAGAGCTCCTCGGATGGGCTCTTGTTCCCAAGCCTACCGTCAACCTGCCGTTATCCACTCGGACAGTGAAGGCGATTGTCGAAGTTGAGGCATTTGCTCTCCAAGCTGATGATCTCAGGTTCGTCGCCAGCCAATTCAGGCGCCTCCACAGCAGGAAGCTGCAGCACACGTTCCGATACTACTCTCACCACTGGAGGACCTGGGCCGCGTGCTTCATCCAGCACGCTTGGCGCCGCCAGAAAAGGAGGAAGATGGCCAAGGACCTGAGTATGAGGGAGTCGTTCTCCTCCATGAGATCATACGAAGGCGACAACTCCCCTGAACAGAATCTCACGCTTAGAAGAGGAGCCAGCATCATCAGAGAACTGCCTAAGTTCAGGAAACCATCGGAGCCGGATTTCTCAGCAGAGCATGACGACTGA

>SsCNGC9|Sspon.02G0036020-1B

ATGTTTGGGTCCAGGGTCCAGGATGAGGTGGAGATGCAGAGGAGGCCTAACAACAGGATCTTTCCTGATGAGAGACAAAATCAACCTAAGCCATTGTATCAAACTGCACGGGCTGACAGATTTGGTGCAAATAGAATAGATGTGAAGAATCCTGAGAAGCTTAAGGTGTTAAATGAAGGCAACAAGCCGTGGCACCAGCGTATTCTAGACCCTGGAAGTAATATTGTACTGAGATGGAACAGGGTGTACCTTGTGGCATGTTTGTTTGCTCTTTTTATAGATCCTTTTTTCTATTACCTTCCATTGATTAGACAAAATGACAATGGATATTCATGCGTTGCCAAGGACCAGGGACTGAGCATAAGAATCACTGTCCTACGATCACTTGCTGACTTATTTTACATGCTGAACATAGCAATCAAGTTTCATACTGCATATGTGGATCCAAAGTCCAGAGTCCTTGGAAAGGGAGAGCTTGTTGTGGATATTAAGAAGATTCAACAAAGATATATAAGAACTGATTTCTTTGTAGACATACTTGCAGCTGTGCCACTTCCACAGGTTACTGTGTGGTTAATTATGCCTGCGATAAAAAGCTCTGATTATAACATCCGGAACACTACATTTGCTCTCATAATTGTGATTCAATATGTCATAAGAATGTATCTCATTATCCCTTTAAGCAATCAGATTATCAAAGCTGTTGGAGTAGTTGCAAAGTCAGCTTGGGGGGGAGCAGCATACAATCTTCTGCTCTACATGCTTGCAAGCCATATTACTGGTGCAATATATTACCTTCTCTCCATTGAACGGCAAATTACATGCTGGGATCAGCAGTGCGTTGCGGAGTCCAATTGCAACCTTAGGTTTATAAGTTGTGAGAATAGTGGTTCTGATGATTATTCTGAGTGGGCAAAGAATACAGGGATATTTAACAACTGTGATGCCACTACTCCTAATAATATATCATTTAACTACGGGATGTTTTCTAGTGCACTGAGTAAAGGCGCTGTCTCATCTCCATTCCTTGAGAAGTACTTCTATTGTCTATGGTGGGGCTTGCTGCAGCTTAGTTCAAGCGGAAATCCTCTTGTGACAAGTGCATTTATCACAGAGAACTTATTTGCTATAGCAATTGGCGCTATCAGTCTCATACTCTTTGCTCAGTTGATTGGCAAGATGCAGACATACCTGCAGTCTATCAGTAAAAGGCTTGAAGAGTGGAGGCTGAGGCAAAGGGACATGGATGAGTGGATGAGACACCATCAACTCCCATCTCATCTTCAAGAACGTGTCCGGCGGTTTGTTCAAGTTAAATGGCTTGCTACAAGAGGAGTAGAAGAAGAGTCCATCTTGCAAGCTTTGCCTGCTGACATACGTCGGGATGTGCAGCGTCATCTTTGTTTGGACCTTGTTAGACGTGTACCCTTTTTCTCTGAGATGGATGACCAACTTCTTGATGCCATCTGTGAGCGGCTGGTGTCTTTCCTGTGTCCTGAGAATACATACATCTCCCGCGAGGGTGATCCTGTGAATGAGATGCTTTTTATCATACGCGGGAAACTAGAGAGCTCAACAACAAATGGTGGCCGTAGCAACTTCTTCAACTCTATCATCCTGCGCCCTGGTGATTTCGCAGGTGAAGAGCTGCTCACATGGGCCCTACTTCCCAAGACCAATGTCCACTTCCCGCTTTCAACAAGAACTGTACGGAGCCTCACAGAGGTGGAAGCCTTCGCTCTGCGAGCTGAAGACCTGAAGTTCGTCGCAAACCAGTTCCGAAGGCTTCACAGCAAGAAGCTCCAGCACACATTCCGGTTCTACTCCCATCACTGGAGGACCTGGGCTGCCTGCTTCATCCAAGCTGCATGGCGGCAGCACCAGAGAAGGAAGCTGGCTGAGAGCCTCAGCCGATGGGAGTCATACTCATGGTGGTCAGAGGATCACCCAACTGGTGATAAACCCAGACAGGAGGGCACCTCAAGCGGCGGCACAAGGACGATTGCTGAAGGTGCCATTGCCCAGATGCATAAGCTTGCCTCTGCTTCCAGAAGGTTCCGTACCGAGGACATCGCTATTCGCAGGTTGCAGAAGCCTGATGAGCCCGATTTCTCCGCGGACCATTTTGATTGA

>SsCNGC9-2D|Sspon.02G0036020-2D

ATGTTTGGGTCCAGGGTCCAGGATGAGGTGGAGATGCAGAGGAGGCCTAACAACAGGATCTTTCCTGATGAGAGACAAAATCAACCTAAGCCATTGTATCAAACTGCACGGGCTGACAGATTCGGTGCAAATAGAATAGATGTGAAGAATCCTGAGAAGCTTAAGGTGTTAAATGAAGGCAACAAACCGTGGCACCAGCGTATTCTAGACCCTGGAAGTAATATTGTACTGAGATGGAACAGGGTGTACCTTGTGGCATGTTTGTTTGCTCTTTTTATAGATCCTTTTTTCTATTACCTTCCATTGATTAGACAAAATGACAATGGATATTCATGCGTTGCCAAGGACCAGGGACTGAGCATAAGAATCACTGTCCTACGATCACTTGCTGACTTATTTTACATGCTGAACATAGCAATCAAGTTTCATACTGCATATGTGGATCCAAAGTCCAGAGTCCTTGGAAAGGGAGAGCTTGTTGTGGATATTAAGAAGATTCAACAAAGATATATAAGAACTGATTTCTTTGTAGACATACTTGCAGCTGTGCCACTTCCACAGGTTACTGTGTGGTTAATTATGCCTGCGATAAAAAGCTCTGATTATAACATACGGAACACTACATTTGCTCTCATAATTGTGATTCAATATGTCATAAGAATGTATCTCATCATCCCTTTAAGCAATCAGATTATCAAAGCTGTTGGAGTAGTTGCAAAGTCAGCTTGGGGGGGAGCAGCATACAATCTTCTGCTCTACATGCTTGCAAGCCATATTACTGGTGCAATATATTACCTTCTCTCCATTGAACGGCAAATTACATGCTGGGATCAGCAGTGCGTTGCGGAGTCCAATTGCAACCTTAGGTTTATAAGTTGTGAGAATAGTGGTTCTGATGATTATTCTGAGTGGGCAAAGAAGACAGGGATATTTAACAACTGTGATGCCACTACTCCTAATAATATATCATTTAACTACGGGATGTTTTCTAGTGCACTGAGTAAAGGCGCTGTCTCATCTCCATTCCTTGAGAAGTACTTCTATTGTCTATGGTGGGGCTTGCTGCAGCTTAGTTCAAGCGGAAATCCTCTTGTGACAAGTGCATTTATCACAGAGAACTTATTTGCTATAGCAATTGGCGCTATCAGTCTCATACTCTTTGCTCAGTTGATTGGCAAGATGCAGACATACCTGCAGTCTATCAGTAAAAGGCTTGAAGAGTGGAGGCTGAGGCAAAGGGACATGGATGAGTGGATGAGACACCATCAACTCCCATCTCATCTTCAAGAACGTGTCCGGCGGTTTGTTCAAGTTAAATGGCTTGCTACAAGAGGAGTAGAAGAAGAGTCCATCTTGCAAGCTTTGCCTGCTGACATACGTCGGGATGTGCAGCGTCATCTTTGTTTGGACCTTGTTAGACGTGTACCCTTTTTCTCTGAGATGGATGACCAACTTCTTGATGCCATCTGTGAGCGGCTGGTGTCTTTCCTGTGTCCTGAGAATACATACATCTCCCGGGAGGGTGATCCTGTGAATGAGATGCTTTTTATCATACGTGGGAAACTAGAGAGCTCAACAACAAATGGTGGCCGTAGCAACTTCTTCAACTCTATCATCCTGCGCCCTGGTGATTTCGCAGGTGAAGAGCTGCTCACATGGGCCCTACTTCCCAAGACCAATGTCCACTTCCCGCTTTCAACAAGAACTGTACGGAGCCTCACAGAGGTGGAAGCCTTCGCTCTGCGAGCTGAAGACCTGAAGTTCGTCGCAAACCAGTTCCGAAGGCTTCACAGCAAGAAGCTTCAGCACACATTCCGGTTCTACTCCCATCACTGGAGGACCTGGGCTGCCTGCTTCATCCAAGCTGCATGGCGGCAGCACCAGAGAAGGAAGCTGGCTGAGAGCCTCAGCCGATGGGAGTCATACTCATGGTGGTCAGAGGATCACCCGCCTGGTGATAAACCCAGACAGGAGGGCACCTCAAGCGGTGGCACAAGGACGATTGCTGAAGGTGCCATTGCCCATATGCATAAGCTTGCCTCTGCTTCCAGAAGGTTCCGCACTGAGGACATCACTATTCGCAGGTTGCAGAAGCCTGATGAGCCTGATTTCTCTGCGGACCATTTTGATTGA

>SsCNGC9-1T|Sspon.02G0036020-1T

ATGTTTGGGTCCAGGGTCCAGGATGAGGTGGAGATGCAGAGGAGGCCTAACAACAGGATCTTTCCTGATGAGAGACAAAATCAACCTAAGCCATTGTATCAAACTGCACGGGCTGACAGATTCGGTGCAAATAGAATAGATGTGAAGAATCCTGAGAAGCTTAAGGTGTTAAATGAAGGCAACAAACCGTGGCACCAGCGTATTCTAGACCCTGGAAGTAATATTGTACTGAGATGGAACAGGGTGTACCTTGTGGCATGTTTGTTTGCTCTTTTTATAGATCCTTTTTTCTATTACCTTCCATTGATTAGACAAAATGACAATGGATCTTCATGCGTTGCCAAGGACCAGGGACTGAGCATAAGAATCACTGTCCTACGATCACTTGCTGACTTATTTTACATGCTGAACATAGCAATCAAGTTTCATACTGCATATGTGGATCCAAAGTCCAGAGTCCTTGGAAAGGGAGAGCTTGTTGTGGATATTAAGAAGATTCAACAAAGATATATAAGAACTGATTTCTTTGTAGACATACTTGCAGCTGTGCCACTTCCACAGGTTACTGTGTGGTTAATTATGCCTGCGATAAAAAGCTCTGATTATAACATACGGAACACTACATTTGCTCTCATAATTGTGATTCAATATGTCATAAGAATGTATCTCATCATCCCTTTAAGCAATCAGATTATCAAAGCTGTTGGAGTAGTTGCAAAGTCAGCTTGGGGGGGAGCAGCATACAATCTTCTGCTCTACATGCTTGCAAGCCATATTACTGGTGCAATATATTACCTTCTCTCCATTGAACGGCAAATTACATGCTGGGATCAGCAGTGTGTTGCGGAGTCCAATTGCAACCTTAGGTTTATAAGTTGTGAGAATAGTGGTTCTGATGATTATTCTGAGTGGGCAAAGAAGACAGGGATATTTAACAACTGTGATGCCACTACTCCTAATAATATATCATTTAACTACGGGATGTTTTCTAGTGCACTGAGTAAAGGCGCTGTCTCATCTCCATTCCTTGATAAGTACTTCTATTGTCTATGGTGGGGCTTGCTGCAGCTTAGTTCAAGCGGAAATCCTCTTGTGACAAGTGCATTTATCACAGAGAACTTATTTGCTATAGCAATTGGCGCTATCAGTCTCATACTCTTTGCTCAGTTGATTGGCAAGATGCAGACATACCTGCAGTCTATCAGTAAAAGGCTTGAAGAGTGGAGGCTGAGGCAAAGGGACATGGATGAGTGGATGAGACACCATCAACTCCCATCTCATCTTCAAGAACGTGTCCGGCGGTTTGTTCAAGTTAAATGGCTTGCTACAAGAGGAGTAGAAGAAGAGTCCATCTTGCAAGCTTTGCCTGCTGACATACGTCGGGATGTGCAGCGTCATCTTTGTTTGGACCTTGTTAGACGTGTACCCTTTTTCTCTGAGATGGATGACCAACTTCTTGATGCCATCTGTGAGCGGCTGGTGTCTTTCCTGTGTCCTGAGAATACATACATCTCCCGGGAGGGTGATCCTGTGAATGAGATGCTTTTTATCATACGTGGGAAACTAGAGAGCTCAACAACAAATGGTGGCCGTAGCAACTTCTTCAACTCTATCATCCTGCGCCCTGGTGATTTCGCAGGTGAAGAGCTGCTCACATGGGCCCTACTTCCCAAGACCAATGTCCACTTCCCGCTTTCAACAAGAACTGTACGGAGCCTCACAGAGGTGGAAGCCTTCGCTCTGCGAGCTGAAGACCTGAAGTTCGTCGCAAACCAGTTCCGAAGGCTTCACAGCAAGAAGCTTCAGCACACATTCCGGTTCTACTCCCATCACTGGAGGACCTGGGCTGCCTGCTTCATCCAAGCTGCATGGCGGCAGCACCAGAGAAGGAAGCTGGCTGAGAGCCTCAGCCGATGGGAGTCATACTCATGGTGGTCAGAGGATCACCCGCCTGGTGATAAACCCAGACAGGAGGGCACCTCAAGCGGTGGCACAAGGACGATTGCTGAAGGTGCCATTGCCCATATGCATAAGCTTGCCTCTGCTTCCAGAAGGTTCCGCACTGAGGACATCACTATTCGCAGGTTGCAGAAGCCTGATGAGCCTGATTTCTCTGCGGACCATTTTGATTGA

>SsCNGC10|Sspon.04G0024390-1B

ATGACCTTTTTAGTTCTTATGGCTGAGCACAATGTCGTGGTGAGCGCCTTCCCGCGCGATCAGATAACTGAACAAATCCACGTTACTGAACAAAGGATTCAGCAGGATCAATCAATGTTTATAACGAAAGCCATGTATGCTTCTCTGCGAAATTTCAGGTTTCAGAACGAGATCGAGGTCCAGAGCTTCAGAACAAGCCCTCTGCAGCAGAACCTCAGCAGCAGAAAGCACGGCAGAGCTCACGATCCCAGGAAATGCCGGCTGGGTTTCCGCGGCGGCTGCCTGGAGAAGGCGTGCCGGAACCCGACGCTGAAGGACAGGGTGCTCTCGCGCGCCTTCTCGGAGGAGCTGGAGTCCCTGATGCACGCCGCCGGCAGCAGCCACCTCTTCTTTGACCCGCGCGGGCACCTGATCCACCTGTGGAACAAGATCTTCCTGTCCGCCTGCCTTCTGTCGCTGTTCGTGGACCCGCTGTTCCTGTACCTGACGGGCACGCAGCGGAACAACCACATGTGCATCGAGTTCAAGTACTCGCTGGCGCTCACGCTCTCCATGATCCGCTCGCTGCTGGACCTCTTCTACGCCGCGCACATCCTGTTCCGCTTCCGCACCGCCTTCATCGCGCCGTCGTCGCGCGTGTTCGGGCGGGGCGAGCTCGTCATCCAGCCCTACAAGATCGCCAGGCGGTACCTTGGCCGGACGTTCTGGTTTGACCTCGTCACCGCGCTGCCCCTACCGCAGTTCGTGATCTGGATCGTTATACCGAAGCTGAATGAGTCGCCGACGGCGAACAGGAAGAGCATCCTCCGGTTCAGCATCATCTTCCAGTATCTCCCGCGGCTGTTCCAGATCTTCCCGCTCACGAGCCAGATCGTCGTGGCGACGGGCGTCGTGGCGGAGACGGCGTGGGCCTGCGCCGCGTACAACCTGATCCTCTACATGCTCGCAAGCCACATAGATGCTGATTTGATTTTCTTTTCTTTTTGGACGACGGAACCGGACGAGTATGTACAGGTGCTGGGAGCGCTGTGGTATCTCTTCTCAGTGCAGAGGCAGGAGGCGTGCTGGAGGGAGGCGTGCCTGCTGGAGAGCCCGACGTGCCAGACTATGTTCTTCGACTGCAAGGCGTTGAGCAGCAACAGGACCATCTGGTATGAGCTGAGCAACATCACAAGCCTGTGCACGCCCGGCAACGGGTTCTACGCGTTCGGCATCTACGAGGAGGCGCTGCACGCCAAGCTCACGTCGTCGTCCTTCACCCAGAAGTACTTCTACTGCTTCTGGTGGGGACTCAAGAACCTCAGCTGCTTAGGACAGAATCTGTCGACGAGCTTGTCCATCGGTGAGATAACCTTCGCCATCGTCATCGGCGTTCTTGGGTTGGTGCTGTTTGGCCTGCTCATCGGCAACATGCAATCTTACCTCCAAACAACGATGGTGCGGCTGGAGGAGTGGCGGACGAAGCGGACGGACATGGAGCGGTGGATGCACCACCGGCAGATCCCCCAGCCGCTGAAGCAATGCGTGAGGAGGTACCACCAGTACCAGTGGGTGGCCACGCGCGGCGTCGACGAGGAGGCCCTGCTCCAGGACCTCCCCATGGACATCCGCCGCGACATCAAGCGCCACCTCTGCCTGGACCTCGTCCGGAGGGTGCCCCTGTTCGACGAGATGGACGAGCGGATGCTGGAGGCCATCTGCGAGCGCCTGAGGCCGGCGCTGTACACGCGCGGCACGCGGCTGGTGCGGGAGCTGGACCCCGTCGACTCCATGCTCTTCATCATCCGGGGCTACCTCGACTCCTTCACGACGCAGGGCGGCCGGTCCGGCTTCTTCAACTCGTGCCGCATCGGCGCCGGGGAGTTCTGCGGGGAGGAGCTCCTGACGTGGGCGCTGGACCCGCGGCCCTCGGCGAAGCTGCCGCTGTCCACCCGGACCGTGCGCGCCGTGTCCGAGGTCGAGGCGTTCGCGCTCGTGGCCGACGACCTCCGCTTCGTGGCGTCGCAGTTCCGCCGCCTGCACAGCGCGCGCATCCGCCACAGGTTCCGCTTCTACTCGCACCAGTGGCGCACGTGGGCCGCGTGCTCCATCCAGGCCGCCTGGCGCCGGCACAAGCGGCGCCGCGCGTCCGTGGAGCTCAGGGTGCGCGAGGGCGGCGACGTGCGGACCGCGGGCAGCTTGAGGCGGTCTTGCCGCCACAGCATCGACGGCGAGGCGTCAATTAAGAAGCCCATGGAACCGGACTTCACGGTGGAAGAAGAGGACTGA

>SsCNGC10-2C|Sspon.04G0024390-2C

ATGTATGCTTCTCTGCGAAATTTCAGGTTTCAGAACGAGATCGAGGTCCAGAGCTTCAGAACAAGCCCTCTGCAGCAGAACCTCAGCAGCAGAAAGCACGGCAGAGCTCACGATCCCAGGAAATGCCGGCTGGGTTTCCGCGGCGGCTGCCTGGAGAAGGCGTGCCGGAACCCGACGCTGAAGGACAGGGTGCTCTCGCGCGCCTTCTCGGAGGAGCTGGAGTCCCTGATGCACGCCGCCGGCAGCAGCCACCTCTTCTTTGACCCGCACGGGCACCTGATCCACCTGTGGAACAAGATCTTCCTGTCCGCCTGCCTGCTGTCGCTGTTCGTGGACCCGCTGTTCCTGTACCTGACGGGCACGCAGCGGAACAACCACATGTGCATCGAGTTCAAGTACTCGCTGGCGCTCACGCTCTCCATGATCCGCTCGCTGCTGGACATCTTCTACGCCGCGCACATCCTGTTCCGCTTCCGCACCGCCTTCGTCGCGCCGTCGTCGCGCGTGTTCGGGCAGGGCGAGCTCGTCATCCAGCCCTACAAGATCGCCAGGCGGTACCTTGGCCGGACGTTCTGGTTCGACCTCGTCACCGCGCTGCCCCTGCCGCAGTTCGTGATCTGGATTGTTATACCGAAGCTGAATGAGTCGCTGACGGGCACGCAGCGGAACAACCACATGTGCATCGAGTTCAAGTACTCGCTGGCGCTCACGCTCTCCATGATCCGCTCGCTGCTGGACATCTTCTACGCCGCGCACATCCTGTTCCGCTTCCGCACCGCCTTCGTCGCGCCGTCGTCGCGCGTGTTCGGGCAGGGCGAGCTCGTCATCCAGCCCTACAAGATCGCCAGGCGGTACCTTGGCCGGACGTTCTGGTTCGACCTCGTCACCGCGCTGCCCCTGCCGCAGTTCGTGATCTGGATTGTTATACCGAAGCTGAATGAGTCGCTGACGGCGAACAGGAAGAGCATCCTCCGGTTCAGCATCATCTTCCAGTACCTCCCGCGGCTGTTCCAGATCTTCCCGCTCACGAGCCAGATCGTCATGGCGACGGGCGTCATGGCGGAGACGGCGTGGGCCTGCGCCGCGTACAACCTGATCCTCTACATGCTCGCAAGCCACGTGCTGGGAGCGCTGTGGTATCTCTTCTCGGTGCAGCGGCAGGAGGCGTGCTGGAGGGAGGCGTGCCTACTGGAGAGCCCGACGTGCCAGACCATGTTCTTCGACTGCAAGGCGTTGAGCAGCAACAGGACCATCTGGTATGAGCTGAGCAACATCACAAGCCTGTGCACGCCGGGCAACGGGTTCTACCCGTTCGGCATCTACGCGGAGGCGCTGCAAACCAAGCTCACGTCGTCGTCCTTCACCCAGAAGTACTTCTACTGCTTCTGGTGGGGACTCAAGAACCTCAGCTGCTTAGGACAGAATCTGTCGACGAGCTTGTCCATCGGTGAGATAACCTTCGCCATCGTCATCGGCGTTCTTGGGTTGGTGCTGTTTGGCCTGCTCATCGGCAACATGCAATCTTACCTCCAAACAACGATGGTGCGGCTGGAGGAGTGGCGGACGAAGCGGACGGACATGGAGCGGTGGATGCACCACCGGCAGATCCCCCAGCCGCTGAAGCAGTGCGTGAGGAGGTACCACCAGTACCAGTGGGTGGCCACGCGCGGCGTCGACGAGGAGGCCCTGCTCCAGGACCTCCCCATGGACATCCGCCGCGACATCAAGCGCCACCTCTGCCTGGACCTCGTCCGGAGGGTGCCCCTGTTCGACGAGATGGACGAGAGGATGCTGGAGGCCATCTGCGAGCGCCTGAGGCCGGCGCTGTACACGCGTGGCACGCGGCTGGTGCGGGAGCTGGACCCCGTCGACTCCATGCTCTTCATCATCCGAGGCTACCTCGACTCCTTCACGACGCAGGGCGGCCGGTCGGGGTTCTTCAACTCGTGCCGCATCGGCGCCGGGGAGTTCTGCGGGGAGGAGCTCCTGACGTGGGCGCTGGACCCGCGGCCCTCGGCGAAGCTGCCGCTGTCCACCCGGACCGTGCGGGCCGTGTCCGAGGTCGAGGCGTTCGCGCTCGTGGCCGACGACCTCCGCTTCGTGGCGTCGCAGTTCCGCCGCCTGCACAGCGCGCGCATCCGCCACAGGTTCCGCTTCTACTCGCACCAGTGGCGCACGTGGGCCGCGTGCTCCATCCAGGCCGCCTGGCGCCGGCACAAGCGGCGCCGCGCGTCCGTGGAGCTCAGGGTGCGCGAGGGCGGCGACGTGCGGACCGCGGGCAGCTTGAGGCGGTCTTGCCGCCACAGCATCGACGGCGAGGCGTCAATTAAGAAGCCCATGGAACCGGACTTCACGGTGGAAGAAGAGGACTGA

>SsCNGC10-3D|Sspon.04G0024390-3D

ATGGCATCCGGTGCTTCACGAAATGTCAGGTTTCAGAACGAGATCGAGGTCCAGAGCTTCAGAACAAGCCCTCTGCAGCAGAACCTCAGCAGCAGAAAGCACGGCAGAGCTCACGATCCCAGGAAATGCCGGCTGGGTTTCCGCGGCGGCTGCCTGGAGAAGGCGTGCCGGAACCCGACGCTGAAGGACAGGGTGCTCTCGCGCGCCTTCTCGGAGGAGCTGGAGTCCCTGATGCACGCCGCCGGCAGCAGCCACCTCTTCTTTGACCCGCGCGGGCACCTGATCCACCTGTGGAACAAGATCTTCCTGTCCGCCTGCCTTCTGTCGCTGTTCGTGGACCCGCTGTTCCTGTACCTGACGGGCACGCAGCGGAACAACCACATGTGCATCGAGTTCAAGTACTCGCTGGCGCTCACGCTCTCCATGATCCGCTCGCTGCTGGACCTCTTCTACGCCGCGCACATCCTGTTCCGCTTCCGCACCGCCTTCATCGCGCCGTCGTCGCGCGTGTTCGGGCGGGGCGAGCTCGTCATCCAGCCCTACAAGATCGCCAGGCGGTACCTTGGCCGGACGTTCTGGTTTGACCTCGTCACCGCGCTGCCCCTACCGCAGTTCGTGATCTGGATCGTTATACCGAAGCTGAATGAGTCGCCGACGGCGAACAGGAAGAGCATCCTCCGGTTCAGCATCATCTTCCAGTGTCTCCCGCGGCTGTTCCAGATCTTCCCGCTCACGAGCCAGATCATCATGGCGACGGGCGTCATGGCGGAGACGGCGTGGGCCTGCGCCGCGTACAACCTGATCCTCTACATGCTCGCAAGCCACATAGATGCTGATTTGATTTTCTTTTCTTTTTGGACGACGGAACCGGACGAGTATGTACAGGTGCTGGGAGCGCTGTGGTATCTCTTCTCAGTGCAGAGGCAGGAGGCGTGCTGGAGGGAGGCGTGCCTGCTGGAGAGCCCGACGTGCCAGACTATGTTCTTCGACTGCAAGGCGTTGAGCAGCAACAGGACCATCTGGTATGAGCTGAGCAACATCACAAGCCTGTGCACGCCCGGCAACGGGTTCTACGCGTTCGGCATCTACGAGGAGGCGCTGCACGCCAAGCTCACGTCGTCGTCCTTCACCCAGAAGTACTTCTACTGCTTCTGGTGGGGACTCAAGAACCTCAGCTGCTTAGGACAGAATCTGTCGACGAGCTTGTCCATCGGTGAGATAACCTTCGCCATCGTCATCGGCGTTCTTGGGTTGGTGCTGTTTGGCCTGCTCATCGGCAACATGCAATCTTACCTCCAAACAACGATGGTGCGGCTGGAGGAGTGGCGGACGAAGCGGACGGACATGGAGCGGTGGATGCACCACCGGCAGATCCCCCAGCCGCTGAAGCAGTGCGTGAGGAGGTACCACCAGTACCAGTGGGTGGCCACGCGCGGCGTCGACGAGGAGGCCCTGCTCCAGGACCTCCCCATGGACATCCGCCGCGACATCAAGCGCCACCTCTGCCTGGACCTCGTCCGGAGGGTGCCCCTGTTCGACGAGATGGACGAGCGGATGCTGGAGGCCATCTGCGAGCGCCTGAGGCCGGCGCTGTACACGCGCGGCACGCGGCTGGTGCGGGAGCTGGACCCCGTCGACTCCATGCTCTTCATCATCCGAGGCTACCTCGACTCCTTCACGACGCAGGGCGGCCGGTCGGGGTTCTTCAACTCGTGCCGCATCGGCGCCGGGGAGTTCTGCGGGGAGGAGCTCCTGACGTGGGCGCTGGACCCGCGGCCCTCGGCGAAGCTGCCGCTGTCCACCCGGACCGTGCGCGCCGTGTCCGAGGTCGAGGCGTTCGCGCTCGTGGCCGACGACCTCCGCTTCGTGGCGTCGCAGTTCCGCCGCCTGCACAGCGCGCGCATCCGCCACAGGTTCCGCTTCTACTCGCACCAGTGGCGCACGTGGGCCGCGTGCTCCATCCAGGCCGCCTGGCGCCGGCACAAGCGGCGCCGCGCGTCCGTGGAGCTCAGGGTGCGCGAGGGCGGCGACGTGCGGACCGCGGGCAGCTTGAGGCGGTCTTGCCGCCACAGCATCGACGGCGAGGCGTCAATTAAGAAGCCCATGGAACCGGACTTCACGGTGGAAGAAGAGGACTGA

>SsCNGC10-1T|Sspon.04G0024390-1T

ATGGCATCCGGTGCTTCACGAAATGTCAGGTTTCAGAACGAGATCGAGGTCCAGAGCTTCAGAACAAGCCCTCTGCAGCAGAACCTCAGCAGCAGAAAGCACGGCAGAGCTCACGATCCCAGGAAATGCCGGCTGGGTTTCCGCGGCGGCTGCCTGGAGAAGGCGTGCCGGAACCCGACGCTGAAGGACAGGGTGCTCTCGCGCGCCTTCTCGGAGGAGCTGGAGTCCCTGATGCACGCCGCCGGCAGCAGCCACCTCTTCTTTGACCCGCGCGGGCACCTGATCCACCTGTGGAACAAGATCTTCCTGTCCGCCTGCCTTCTGTCGCTGTTCGTGGACCCGCTGTTCCTGTACCTGACGGGCACGCAGCGGAACAACCACATGTGCATCGAGTTCAAGTACTCGCTGGCGCTCACGCTCTCCATGATCCGCTCGCTGCTGGACCTCTTCTACGCCGCGCACATCCTGTTCCGCTTCCGCACCGCCTTCATCGCGCCGTCGTCGCGCGTGTTCGGGCGGGGCGAGCTCGTCATCCAGCCCTACAAGATCGCCAGGCGGTACCTTGGCCGGACGTTCTGGTTTGACCTCGTCACCGCGCTGCCCCTACCGCAGTTCGTGATCTGGATCGTTATACCGAAGCTGAATGAGTCGCCGACGGCGAACAGGAAGAGCATCCTCCGGTTCAGCATCATCTTCCAGTGTCTCCCGCGGCTGTTCCAGATCTTCCCGCTCACGAGCCAGATCATCATGGCGACGGGCGTCATGGCGGAGACGGCGTGGGCCTGCGCCGCGTACAACCTGATCCTCTACATGCTCGCAAGCCACATAGATGCTGATTTGATTTTCTTTTCTTTTTGGACGACGGAACCGGACGAGTATGTACAGGTGCTGGGAGCGCTGTGGTATCTCTTCTCAGTGCAGAGGCAGGAGGCGTGCTGGAGGGAGGCGTGCCTGCTGGAGAGCCCGACGTGCCAGACTATGTTCTTCGACTGCAAGGCATTGAGCAGCAACAGGACCATCTGGTATGAGCTGAGCAACATCACAAGCCTGTGCACGCCCGGCAACGGGTTCTACCCGTTCGGCATCTACGAGGAGGCGCTGAAAACCAAGCTCACGTCGTCGTCCTTCACCCAGAAGTACTTCTACTGCTTCTGGTGGGGACTCAAGAACCTCAGCTGCTTAGGACAGAATCTGTCGACGAGCTTGTCCATCGGTGAGATAACCTTCGCCATCGTCATCGGCGTTCTTGGGTTGGTGCTGTTTGGCCTGCTCATCGGCAACATGCAATCTTACCTCCAAACAACGATGGTGCGGCTGGAGGAGTGGCGGACGAAGCGGACGGACATGGAGCGGTGGATGCACCACCGGCAGATCCCCCAGCCGCTGAAGCAGTGCGTGAGGAGGTACCACCAGTACCAGTGGGTGGCCACGCGCGGCGTCGACGAGGAGGCCCTGCTCCAGGACCTCCCCATGGACATCCGCCGCGACATCAAGCGCCACCTCTGCCTGGACCTCGTCCGGAGGGTGCCCCTGTTCGACGAGATGGACGAGCGGATGCTGGAGGCCATCTGCGAGCGCCTGAGGCCGGCGCTGTACACGCGCGGCACGCGGCTGGTGCGGGAGCTGGACCCCGTCGACTCCATGCTCTTCATCATCCGAGGCTACCTCGACTCCTTCACGACGCAGGGCGGCCGGTCGGGGTTCTTCAACTCGTGCCGCATCGGCGCCGGGGAGTTCTGCGGGGAGGAGCTCCTGACGTGGGCGCTGGACCCGCGGCCCTCGGCGAAGCTGCCGCTGTCCACCCGGACCGTGCGCGCCGTGTCCGAGGTCGAGGCGTTCGCGCTCGTGGCCGACGACCTCCGCTTCGTGGCGTCGCAGTTCCGCCGCCTGCACAGCGCGCGCATCCGCCACAGGTTCCGCTTCTACTCGCACCAGTGGCGCACGTGGGCCGCGTGCTCCATCCAGGCCGCCTGGCGCCGGCACAAGCGGCGCCGCGCGTCCGTGGAGCTCAGGGTGCGCGAGGGCGGCGACGTGCGGACCGCGGGCAGCTTGAGGCGGTCTTGCCGCCACAGCATCGACGGCGAGGCGTCAATTAAGAAGCCCATGGAACCGGACTTCACG

>SsCNGC11|Sspon.04G0008000-1A

ATGTATGCTTCTCTGCGAAATTTCAGGTTTCAGAACGAGATCGAGGTCCAGAGCTTCAGAACAAGCCCTCTGCAGCAGAACCTCAGCAGCAGAAAGCACGGCAGAGCTCACGATCCCAGGAAATGCCGGCTGGGTTTCCGCGGCGGCTGCCTGGAGAAGGCGTGCCGGAACCCGACGCTGAAGGACAGGGTGCTCTCGCGCGCCTTCTCGGAGGAGCTGGAGTCCCTGATGCACGCCGCCGGCAGCAGCCACCTCTTCTTTGACCCGCGCGGGCACCTGATCCACCTGTGGAGCAAGATCTTCCTGTCCGCCTGCCTTCTGTCGCTGTTCGTGGACCCGCTGTTCCTGTACCTGACGGGCACGCAGCGGAACAACAACGTGTGCATCGAGTTCAAGTACTCGCTGGCGCTCACGCTCTCCATGATCCGCTCGCTGCTGGACCTCTTCTACGCCGCGCACATCCTGTTCCGCTTCCGCACCGCCTTCATCGCGCCGTCGTCGCGCGTGTTCGGGCGGGGCGAGCTCGTCATCCAGCCCTACAAGATCGCCAGGCGGTACCTTGGCCGGACGTTCTGGTTTGACCTCGTCACCGCGCTACCCCTACCGCAGTTCGTGATCTGGATCGTTATACTGAAGCTGAATGAGTCGCCGACGACGAACAGGAAGAGCATCCTCCGGTTCAGCATCATCTTCCAGTATCTCCCGCGGCTGTTCCAGATCTTCCCGCTCACGAGCCAGATCATCATGGCGACGGGCGTCATGGCGGAGACGGCGTGGGCCTGCGCCGCGTACAACCTGATCCTCTACATGCTCGCAAGCCACGTGCTGGGAGCGCTGTGGTATCTCTTCTCAGTGCAGAGGCAGGAGGCGTGCTGGAGGGAGGCGTGCCTGCTGGAGAGCCCGACGTGCCAGACTATGTTCTTCGACTGCAAGGCGTTGAGCAGCAACAGGACCATCTGGTATGAGCTGAGCAACATCACAAGCCTGTGCACGCCCGGCAACGGGTTCTACGCGTTCGGCATCTACGAGGAGGCGCTGCACGCCAAGCTCACGTCGTCGTCCTTCACCCAGAAGTACTTCTACTGCTTCTGGTGGGGACTCAAGAACCTCAGCTGCTTAGGACAGAATCTGTCGACGAGCTTGTCCATCGGTGAGATAACCTTCGCCATCGTCATCGGCGTTCTTGGGTTGGTGCTGTTTGGCCTGCTCATCGGCAACATGCAATCTTACCTCCAAACAACGATGGTGCGGCTGGAGGAGTGGCGGACGAAGCGGACGGACATGGAGCGGTGGATGCACCACCGGCAGATCCCCCAGCCGCTGAAGCAGTGCGTGAGGAGGTACCACCAGTACCAGTGGGTGGCCACGCGCGGCGTCGACGAGGAGGCCCTGCTCCAGGACCTCCCCATGGACATCCGCCGCGACATCAAGCGCCACCTCTGCCTGGACCTCGTCCGGAGGGTGCCCCTGTTCGACGAGATGGACGAGCGGATGCTGGAGGCCATCTGCGAGCGCCTGAGGCCGGCGCTGTACACGCGCGGCACGCGGCTGGTGCGGGAGCTGGACCCCGTCGACTCCATGCTCTTCATCATCCGAGGCTACCTCGACTCCTTCACGACGCAGGGCGGCCGGTCGGGGTTCTTCAACTCGTGCCGCATCGGCGCCGGGGAGTTCTGCGGGGAGGAGCTCCTGACGTGGGCGCTGGACCCGCGGCCCTCGGCGAAGCTGCCGCTGTCCACCCGGACCGTGCGCGCCGTGTCCGAGGTCGAGGCGTTCGCGCTCGTGGCCGACGACCTCCGCTTCGTGGCGTCGCAGTTCCGCCGCCTGCACAGCGCGCGCATCCGCCACAGGTTCCGCTTCTACTCGCACCAGTGGCGCACGTGGGCCGCGTGCTCCATCCAGGCCGCCTGGCGCCGGCACAAGCGGCGCCGCGCGTCCGTGGAGCTCAGGGTGCGCGAGGGCGGCGACGTGCGGACCGCGGGCAGCTTTAGGCGGTCTTGCCGCCACAGCATCGACGGCGAGGCGTCAATTAAGAAGCCCATGGAACCGGACTTCACGGTGGAAGAAGAGGACTGA

>SsCNGC11-2B|Sspon.04G0008000-2B

ATGACCTTTTTAGTTCTTATGGCTGAGCACAATGTCGTGGTGAGCGCCTTCCCGCGCGATCAGATAACTGAACAAATCCACGTTACTGAACAAAGGATTCAGCAGGATCAATCAATGTTTATAACGAAAGCCATGTATGCTTCTCTGCGAAATTTCAGGTTTCAGAACGAGATCGAGGTCCAGAGCTTCAGAACAAGCCCTCTGCAGCAGAACCTCAGCAGCAGAAAGCACGGCAGAGCTCACGATCCCAGGAAATGCCGGCTGGGTTTCCGCGGCGGCTGCCTGGAGAAGGCGTGCCGGAACCCGACGCTGAAGGACAGGGTGCTCTCGCGCGCCTTCTCGGAGGAGCTGGAGTCCCTGATGCACGCCGCCGGCAGCAGCCACCTCTTCTTTGACCCGCGCGGGCACCTGATCCACCTGTGGAACAAGATCTTCCTGTCCGCCTGCCTTCTGTCGCTGTTCGTGGACCCGCTGTTCCTGTACCTGACGGGCACGCAGCGGAACAACCACATGTGCATCGAGTTCCAGTACTCGCTGGCGCTCACGCTCTCCATGATCCGCTCGCTGCTGGACATCTTCTACGCCGCGCACATCCTGTTCCGCTTCCGCACCGCCTTCATCGCGCCGTCGTCGCGCGTGTTCGGGCGGGGCGAGCTCGTCATCCAGCCCTACAAGATCGCCAGGCGGTACCTTGGCCGGACGTTCTGGTTTGACCTCGTCACCGCGCTGCCCCTACCGCAGTTCGTGATCTGGATCGTTATACCGAAGCTGAATGAGTCGCCGACGGCGAACAGGAAGAGCATCCTCCGGTTCAGCATCATCTTCCAGTATCTCCCGCGGCTGTTCCAGATCTTCCCGCTCACGAGCCAGATCGTCGTGGCGACGGGCGTCGTGGCGGAGACGGCGTGGGCCTGCGCCGCGTACAACCTGATCCTCTACATGCTCGCAAGCCACGTGCTGGGAGCGCTGTGGTATCTCTTCTCAGTGCAGAGGCAGGAGGCGTGCTGGAGGGAGGCGTGCCTGCTGGAGAGCCCGACGTGCCAGACTATGTTCTTCGACTGCAAGGCGTTGAGCAGCAACAGGACCATCTGGTATGAGCTGAGCAACATCACAAGCCTGTGCACGCCCGGCAACGGGTTCTACGCGTTCGGCATCTACGAGGAGGCGCTGCACGCCAAGCTCACGTCGTCGTCCTTCACCCAGAAGTACTTCTACTGCTTCTGGTGGGGACTCAAGAACCTCAGCTGCTTAGGACAGAATCTGTCGACGAGCTTGTCCATCGGTGAGATAACCTTCGCCATCGTCATCGGCGTTCTTGGGTTGGTGCTGTTTGGCCTGCTCATCGGCAACATGCAATCTTACCTCCAAACAACGATGGTGCGGCTGGAGGAGTGGCGGACGAAGCGGACGGACATGGAGCGGTGGATGCACCACCGGCAGATCCCCCAGCCGCTGAAGCAATGCGTGAGGAGGTACCACCAGTACCAGTGGGTGGCCACGCGCGGCGTCGACGAGGAGGCCCTGCTCCAGGACCTCCCCATGGACATCCGCCGCGACATCAAGCGCCACCTCTGCCTGGACCTCGTCCGGAGGGTGCCCCTGTTCGACGAGATGGACGAGCGGATGCTGGAGGCCATCTGCGAGCGCCTGAGGCCGGCGCTGTACACGCGCGGCACGCGGCTGGTGCGGGAGCTGGACCCCGTCGACTCCATGCTCTTCATCATCCGGGGCTACCTCGACTCCTTCACGACGCAGGGCGGCCGGTCCGGCTTCTTCAACTCGTGCCGCATCGGCGCCGGGGAGTTCTGCGGGGAGGAGCTCCTGACGTGGGCGCTGGACCCGCGGCCCTCGGCGAAGCTGCCGCTGTCCACCCGGACCGTGCGCGCCGTGTCCGAGGTCGAGGCGTTCGCGCTCGTGGCCGACGACCTCCGCTTCGTGGCGTCGCAGTTCCGCCGCCTGCACAGCGCGCGCATCCGCCACAGGTTCCGCTTCTACTCGCACCAGTGGCGCACGTGGGCCGCGTGCTCCATCCAGGCCGCCTGGCGCCGGCACAAGCGGCGCCGCGCGTCCGTGGAGCTCAGGGTGCGCGAGGGCGGCGACGTGCGGACCGCGGGCAGCTTGAGGCGGTCTTGCCGCCACAGCATCGACGGCGAGGCGTCAATTAAGAAGCCCATGGAACCGGACTTCACGGTGGAAGAAGAGGACTGA

>SsCNGC12|Sspon.04G0031130-1C

ATGACTGACCAGGAGCGAGATGATGTTCCCATGTTGCTAAGAAATGTTGAGCTACCAAGATTTCCTCTAAGAAGTACTTCAATGTGTATACCAGTCAGGGATGATGAATATGAAGAAGATACCTTTGTACCCCATACTGGTCCTCTATTCGTTCAGCCGCCAACTCAGACGGCACCGGGAATTCCATTTACAAGTAGAGACACACCTGATAGGCTTCCTAGACCTTCACAGGGAAAACAAGTCAGCAAGCCACATGCAATCATGCCTGAAGAAATTAGAGGAAATAGGTGGTCTTACAGTGGACAGGTTCCAAAGAATGAACACTTGATGATGTCTGGACCTTTGGGGCAATGTGATAATCCTGACTGTGTGAATTGCCCTCCTGCTTGTAGAAATAAAAGACATTTCCAGAGAGGTTCGAATGCTTTAGACAATAAGATTCATAACATTCTCTATGGTCACAGTGGTGGATGGAAGAAGAAAATTGAGCAGATCATGGCATACATTCCGATTATGAACCCACATGCAAAGCCGGTTCAGCAGTGGAATCAATTCTTTGTCATATCATGCCTGATTGCCATTTTCATTGATCCCCTGTTCTTCTTCTTACTATCAGTACGTCAGGATGGCAACTGCATAGTGTTAAATTGGAACTTTGCTACAGGACTTGCTGTTGTGAGAAGTGTGACTGATGCTATTTATTTCCTGCACATGCTGCTTCAGTTCAGACTGGCTTATGTTGCGCCAGAGTCACGAGTGGTGGGAGCTGGAGACTTGGTTGATGAGCCAAAGAAAGTTGCTATCCATTATCTTTGTGGTTACTTTTTTCTTGATTTCTTCGTTGTGCTTCCACTCCCTCAGGTGATGATACTGCTAGTTGTTCCTAAAGTTGGGTTATCTGCTGCAAACTATGCTAAGAATTTATTGCGTGCCACTGTTCTTCTTCAATATGTGCCCCGTATCATCAGATTTGTACCACTTCTTGATGGTCAGTCCGCCAATGGATTCATATTTGAGTCAGCATGGGCTAATTTTGTGATCAATCTTCTGATGTTTGTTTTGGCGGGACATGTGGTTGGTTCATGTTGGTATCTCTTTGGCTTACAGAGGGTTAACCAATGTCTACGAGATGCTTGTTCTGCATCGACCATTCCATATTGTGACTCTTTTATAGACTGTGGACGTGGCATGGGGAGTGGACTGTACAGACAGCAGTGGTTCAATGACTTGGGTGCAGAAGCTTGTTTTAACACTGGAAATAATGCTACTTTCCAATATGGAATTTATGAGCAAGCTGTTTTGCTAACTACAGAAGACAGCGCTGTAAAACGATATATATATTCATTATTTTGGGGGTTTCAGCAAATAAGTACCTTAGCAGGAAACCTTGTCCCGAGTTACTTTGTATGGGAAGTTCTGTTCACGATGGCTATTATTGGTCTGGGACTGAGATTGGAAATGCAACTTAGGCGCCGTGATGTTGAAAAGTGGATGAGCCATAGACGATTGCCTGAAGATTTGAGAAGGAGGGTTAGACGAGCTGAAAGGTTCACCTGGGCAGCTACTCAAGGAGTGAATGAAGAGGAGCTTTTGAGTAATTTACCTGAAGATATCCAAAGGGACATACGTCGCCACTTCTTCAGATTCCTTAATAAGGTCCGATTATTCACCTTGATGGATTGGCCTATCTTGGATGCAATATGTGACAAATTAAGACAAAACTTATATATTAGTGGAAGTGACATTCTTTATCAAGGTGGTACTGTTGAAAAGATGGTCTTCATAGTGAGAGGGAAGCTGGAAAGCATCAGTGCAGATGGTAGCAAGGCTCCATTACATGATGGAGATGTATGTGGAGAGGAGCTCCTCACCTGGTACTTGGAACATTCTTCAGCGAATAGAGATGGTGGGAAAATTAAATTCCAAGGTATGCGGTTGGTTGCTATACGCACAGTAAGATGTTTAACAAATGTTGAAGCTTTTGTACTCAGAGCAAGTGATCTGGAAGAAGTAACCTCACAGTTTGCTCGATTCTTGCGTAATCCACGAGTGCAGGGAGCAATCAGATATGAATCCCCGTACTGGCGAACCATTGCTGCAACTCGTATTCAAGTTGCATGGAGGTATCGGAAAAGGCGGCTGAAGCGAGCTGAGAAGTCAAGGTTGAGCGAAGAGACTTATACCTCGCATGGGATCTCGACACATGATTCTTTTCAGCGTGGACAGAAGGGATGA

>SsCNGC12-2D|Sspon.04G0031130-2D

ATGACTGACCAGGAGCGAGATGATGTTCCCATGTTGCTAAGAAATGTTGAGCTACCAAGATTTCCTCTAAGAAGTACTTCAATGTGTATACCAGTCAGGGATGATGAATATGAAGAAGACACCTTTGTACCCCATACTGGTCCTCTATTCGTTCAGCCGCCAACTCAGACGGCACCGGGAATTCCATTTACAAGTAGAGACACACCTGATAGGCTTCCTAGACCTTCACAGGGAAAACAAGTCAGCAAGCCACATGCAATCATGCCTGAAGAAATTAGAGGAAATAGGTGGTCTTACAGTGGACAGGTTCCAAAGAATGAACACTTGATGATGTCTGGACCTTTGGGGCAATGTGATAATCCTGACTGTGTGAATTGCCCTCCTGCTTGTAGAAATAAAAGACATTTCCAGAGAGGTTCGAATGCTTTAGACAATAAGATTCATAACATTCTTTATGGTCACAGTGGTGGATGGAAGAAGAAAATTGAGCAGATCATGGCATACATTCCAATTATGAACCCACATGCAAAGCCGGTTCAGCAGTGGAATCAATTCTTTGTCATATCATGCCTGATTGCCATTTTCATTGATCCCCTGTTCTTCTTCTTACTATCAGTACGTCAGGATGGCAACTGCATAGTGTTAAATTGGAACTTCGCTACAGGACTTGCTGTTGTGAGAAGTGTGACTGATGCTATTTATTTCCTGCACATGCTGCTTCAGTTCAGACTGGCTTATGTTGCGCCAGAGTCACGAGTGGTGGGAGCTGGAGACTTGGTTGATGAGCCAAAGAAAGTTGCTATCCATTATCTTTGTGGTTACTTTTTTCTTGATTTCTTCGTTGTGCTTCCACTCCCTCAGGTGATGATACTGCTAGTTGTTCCTAAAGTTGGGTTATCTGCTGCAAACTATGCTAAGAATTTATTGCGTGCCACTGTTCTTCTTCAATATGTGCCCCGTATCATCAGATTTGTACCACTTCTTGATGGTCAGTCCGCCAATGGATTCATATTTGAGTCAGCATGGGCTAATTTTGTGATCAATCTTCTGATGTTTGTTTTGGCGGGACATGTGGTTGGTTCATGTTGGTATCTCTTTGGCTTACAGAGGGTTAACCAATGTCTACGAGATGCTTGTTCTGCATCGACCATTCCATATTGTGACTCTTTTATAGACTGTGGACGTGGCATGGGGAGTGGACTGTACAGACAGCAGTGGTTCAATGACTTGGGTGCAGAAGCTTGTTTTAACACTGGAAATAATGCTACTTTCCAATATGGAATTTATGAGCAAGCTGTTTTGCTAACTACAGAAGACAGCGCTGTAAAACGATATATATATTCATTATTTTGGGGGTTTCAGCAAATAAGTACCTTAGCAGGAAACCTTGTCCCGAGTTACTTTGTATGGGAAGTTCTGTTCACGATGGCTATTATTGGTCTGGGACTGAGATTGGAAATGCAACTTAGGCGCCGTGATGTTGAAAAGTGGATGAGCCATAGACGATTGCCTGAAGATTTGAGAAGGAGGGTTAGACGAGCTGAAAGGTTCACCTGGGCAGCTACTCAAGGAGTGAATGAAGAGGAGCTTTTGAGTAATTTACCTGAAGATATCCAAAGGGACATACGTCGCCACTTCTTTAGATTCCTTAATAAGGTCCGATTATTCACCTTGATGGATTGGCCTATCTTGGATGCAATATGTGACAAATTAAGACAAAACTTATATATTAGTGGAAGTGACATTCTTTATCAAGGTGGTACTGTTGAAAAGATGGTCTTCATAGTGAGAGGGAAGCTGGAAAGCATCAGTGCAGATGGTAGCAAGGCTCCATTACATGATGGAGATGTATGTGGAGAGGAGCTCCTCACCTGGTACTTGGAACATTCTTCAGCGAATAGAGATGGTGGGAAAATTAAATTCCAAGGTATGCGGTTGGTTGCTATACGCACAGTAAGATGTTTAACAAATGTTGAAGCTTTTGTACTCAGAGCAAGTGATCTGGAAGAAGTAACCTCACAGTTTGCTCGATTCTTGCGTAATCCACGAGTGCAGGGAGCAATCAGATATGAATCCCCGTACTGGCGAACCATTGCTGCAACTCGTATTCAAGTTGCATGGAGGTATCGGAAAAGGCGGCTGAAGCGAGCTGAGAAGTCAAGGTTGAGCGAAGAGACTTATACCTCGCATGGGATCTCGACACATGATTCTTTTCAGCGTGGACAGAAGGGATGA

>SsCNGC13|Sspon.01G0049590-1B

ATGCCTCCGCTCGCATTCCTCCGCCGCTCCCTCCCCGCGAGGTTCATGCTCGCCTCCACAACTTCATCATCACTTGGAGGAATCGAGTTCCTCGAGTCTGCGCCAATGCAAAGGCTTCTCGCGCGAGCGTGTGATGGTGCCAGTGGAGTCTGGGGGAGCCCGGGCGTGGCGCGGGACGAGGAGGCGGGAGGCGGCGGCGGCGGGCTGAGCGGCCGGTCGGCGGGGGGTCCGTCCGGGGAGTGCTACGCGTGCACGCAGCCCGGGGTGCCGGCGTTCCACTCCACGACCTGCGACCAGGTGCACTCGCCGGACTGGGACGCCGACGCTGGGTCCTCGCTCGTGCCGGTCCAGGCGCAGCAGTCCCAGCCAGCGGCGTCGGCGTCCGCGGCGGCGCAGCACGCGGGCGCCGCGGCGCGGTGGCTGTTCGGGCCCGTGCTGGACCCGCGGAGCAAGCGCGTGCAGCGCTGGAACCGCTGGATCCTGCTCGGCCGCGCCGCCGCGCTGGCGGTGGACCCGCTCTTCTTCTACGCGCTCTCTATCGGCCGCGCGGGGCAGCCCTGCCTCTACATGGACGCCGGCCTCGCCGCCGCGGTCACCGCGCTCCGGACCTGCGCCGACGTCGCCCACCTCGCGCACGTGCTCCTGCAGTTCCGCCTCGCCTACGTCTCCCGCGAGTCCCTCGTCGTCGGCTGCGGCAAGCTCGTCTGGGACGCCCGCGCCATCGCCGCGCACTACGCCCGCTCCGTCAAGGGCCTCTGCTTCGACCTCTTCGTCATCCTCCCCATCCCGCAGGTCATCTTCTGGCTGGTTATACCAAAGTTAATTAGGGAAGAGCAAGTTAAGCTTAGAATTCCACGTGGACAAGTTGTCGCGGCCGCTTCTATAGTGTCACCTAAAAGCGGCCGCGACAACTTGTCCACTACTTTTGGTAACGATCTGGCCCCTACAAGCAATGGTATTGAGGTGATATTCAGCATAATCAATGTCCTCAGTGGCCTGATGCTCTTCACATTGCTGATCGGAAACATACAGGTGTTTCTGCACGCCGTCCTGGCAAGGAAGAGGAAGATGCAGCTGCGGTTCCGGGACATGGAATGGTGGATGAGACGGAGGCAGCTGCCATCCCGGCTGAGGCAAAGGGTCCGCAAATACGAGCGCGAACGCTGGGCCGCCGTCACGGGAGACGAGGAGATGGAGATGATCAAGGACCTGCCTGAAGGACTCAGGCGGGACATCAAGCGCTACCTCTGCCTCGAGCTGGTTAAGCAGGTTCCGCTGTTCCATGGCATGGACGACCTGATCCTGGACAACATCTGCGACCGGCTGCGGCCTCTGGTGTTCTCCAGCGGGGAGAAGGTGATCCGGGAGGGCGACCCCGTGCAGCGCATGGTGTTCATCCTGCAGGGCAAGCTCCAGAGCACGCAGCCGCTGACTAAGGGCGTGGTGGCGACGTGCATGCTGGGCGCGGGCAACTTCCTAGGCGACGAGCTGCTGTCGTGGTGCCTGCGCCGCCCCTTCGTGGACCGGCTCCCCGCGTCGTCGGCCACGTTCGAGTGCGTGGAGGCGGCGCAGGCGTTCTGCCTCGACGCCCCGGACCTGCGGTTCATCACCGAGCACTTCCGCTACAAGTTCGCCAACGAGAAGCTCAAGCGCACGGCGCGCTACTACTCGTCCAACTGGCGGACGTGGGCCGCCGTCAACATACAGCTCGCGTGGCGCCGGTACAGGGCCCGGACGTCGACGACGGACCTGGCAGCGATGGCCGCGGTGCCATTGGTTGGCGGGCCCGACGACGGGGACCGGCGGCTCAGACACTACGCGGCCATGTTCATGTCGCTCCGGCCGCATGACCACCTAGATGGTGAGCTGGGTGTGCGGCGCGGCGGTGTACGGGCTTCATCTCCGTTCCCCCGGCTTGATCTGTTCCGTCGTGGCACGGCGCCGCTCCTTCTGCTGCCGGGCTCCTTGGAGGCGGTCTTCTTCCTTCTTCCTCTCTGGGCTGTTGTGCTGGTGCTAGTCTGGGATTTGGGTGTTCTTCTTTCAGATCTGATGGATCAAAAAACTAAGCTTGAGATGATGGGTACCCAAGCAGCAACTCTAGTTGTGGTGGTGGTTTCTTTTGTGGTCAGTAGGCTTAGAAGGTTTGTTGTTGCTGCTAACCTCGTGCAAGGTGTGGTGGCTGCTGCTGGTGTAGCTGCAGTTGTGCAGGCAGCTGTAGCAACACCTGTGGATGCCCCTGTCGTTGATGATGTTGTGGGTGATGCAGCAGATGGTGAGGGAGAAGCTGGTGGAGCTAGGCCACCCATGATGTGGAATAACAATACCTTTGGGTTTGTCCTTAAGAGGATGGCTCAGATCTTGTTTGATGGGAGTAGGACTGACAAGCTTTTTAAGCACCACCCAAAGGATGCTGAGTTTTTGAACACCCCTATCAGGTTCTACACTGAGATGCAAACCATATTTGGTAGCACCTTGGCTACTGGTGGAGGGGCATGGCTTCATTGCTCTACACCTGCCTTTGAGGGGAAAACCACCATTGAGTTGGGTGAAGGCAGTAAGGCCACTTTACCTGTGACCTCCATAGCTGGGGGTAAGAGGAAGAGGGTTGCCTTCAGTGAGGATGAGATGCTTATGATGACCAATATGACAGATGCTGTCAACAATGTGGCCAATGCTATGATGAAAGCAGCTGCTACACATGTGGATCCTGCCCTCTACCTTGCTATCATGGAGATGCCTGACTTCTCCATTGAGGCACTCATTGTTGCCTACACTCACCTGCTGGAGAACAAGGCAGTGCATTAA

>SsCNGC13-1P|Sspon.01G0049590-1P

ATGCCTCCGCTCGCATTCCTCCGCCGCTCCCTCCCCGCGAGGTTCATGCTTGCCTCCACAACTTCATCATCACTTGGAGGAATCGAGTTCCTCGAGTTTGCGCCAATGGTTTCGTCTTTGGATCGGAGTCAAAGGCTTCTCGCTCGAGCGTGTGATGGTGGAGTCTGGGGGAGCCCGGGCGTGGCGCGGGACGAGGAGGCGGGAGGCAGCGGCGGCGGGCTGAGCGGCCGGTCGGCGGGGGGTCCGTCCGGGGAGTGCTACGCGTGCACGCAGCCCGGGGTGCCGGCGTTCCACTCCACGACTTGCGACCAAGTGCACTCGCCGGACTGGGACGCCGACGCGGGGTCCTCGCTCGTGCCGGTCCAGGCGCAGCAGTCCCAGCCAGCGGCGTCGGCGTCCGCGGCGGCGCAGCACGCGGGTGCCGCGGCGCGGTGGCTGTTCGGGCCCGTGCTGGACCCGCGGAGCAAGCGCGTGCAGCGCTGGAACCGCTGGATCCTGCTCGGCCGCGCCGCCGCGCTGGCGGTGGACCCGCTCTTCTTCTACGCGCTCTCCATCGGCCGCGCGGGGCAGCCCTGCCTCTACATGGACGCCGGCCTCGCCGCCGCGGTCACCGCGCTCCGGACCTGCGCCGACGTCGCCCACCTCGCGCACGTGCTCCTGCAGTTCCGCCTCGCCTACGTCTCCCGCGAGTCCCTCGTCGTCGGCTGCGGCAAGCTCGTCTGGGACGCCCGCGCCATCGCCGCGCACTACGCCCGCTCCGTCAAGGGCCTCTGCTTCGACCTCTTCGTCATCCTCCCCATCCCGCAGGTCATCTTCTGGCTGGTTATACCAAAGTTAATTAGGGAAGAGCAAGTTAAGCTTATCATGACAATACTGCTGCTCATGTTCATATTTCAATTTCTCCCCAAGGTCTACCATATTATACACATCATGAGGAAAATGCAGAAGGTTACTGGTTACATCTTTGGATCAATATGGTGGGGATTTGGTTTAAATCTATTTGCCTATTTCATTGCTTCTCATATTGCTGGTGGGTGCTGGTATGTTCTTGCAATCCAGCGCATTGCTTCCTGCCTCCAGGAAGAATGCAAGAAGAATAACAGTTGTGATCTAATATCGCTAGCTTGTTCCAAGGAGATATGTTTTCACCCTCCTTGGTCATCGAATGTTAATGGATTCGCATGTGATACGAACATGACCTCCTTTAGTCAACGGAATGTGTCTACTTGTTTAAGTGGAAAAGGGACATTTGCTTATGGAATCTATTTGGGGGCTCTTCCTGTTATATCGAGCAATTCACTTGCTGTCAAAATACTCTATCCTATATTTTGGGGCCTCATGACACTCAGTACTTTTGGTAACGATCTGGCCCCTACAAGCAATGGTATTGAGGTGATATTCAGCATAATCAATGTCCTCAGTGGCCTGATGCTCTTCACATTGCTGATCGGAAACATACAGGTGTTTCTGCACGCCGTCCTGGCAAGGAAGAGGAAGATGCAGCTGCGGTTCCGGGACATGGAATGGTGGATGAGACGGAGGCAGCTGCCATCCCGGCTGAGGCAAAGGGTCCGCAAATACGAGCGCGAACGCTGGGCCGCCGTCACGGGAGACGAGGAGATGGAGATGATCAAGGACCTGCCTGAAGGACTCAGGCGGGACATCAAGCGCTACCTCTGCCTCGAGCTGGTTAAGCAGGTTCCGCTGTTCCATGGCATGGACGACCTGATCCTGGACAACATCTGCGACCGGCTGCGGCCTCTGGTGTTCTCCAGCGGGGAGAAGGTGATCCGGGAGGGCGACCCCGTGCAGCGCATGGTGTTCATCCTGCAGGGCAAGCTCCAGAGCACGCAGCCGCTGACTAAGGGCGTGGTGGCGACGTGCATGCTGGGCGCGGGCAACTTCCTAGGCGACGAGCTGCTGTCGTGGTGCCTGCGCCGCCCCTTCGTGGACCGGCTCCCCGCGTCGTCGGCCACGTTCGAGTGCGTGGAGGCGGCGCAGGCGTTCTGCCTCGACGCCCCGGACCTGCGGTTCATCACCGAGCACTTCCGCTACAAGTTCGCCAACGAGAAGCTCAAGCGCACGGCGCGCTACTACTCGTCCAACTGGCGGACGTGGGCCGCCGTCAACATACAGCTCGCGTGGCGCCGGTACAGGGCCCGGACGTCGACGACGGACCTGGCAGCGATGGCCGCGGGCCATTGGTTGGCGGGCCCGACGACGGGGACCGGCGGCTCAGACACTACGCGGCCATGTTCATGTCGCTCCGGCCGCATGACCACCTAG

>SsCNGC14|Sspon.03G0005320-1A

ATGACTCATGCATGCGGCGTGACCATTTTTGTGCGTTCGCAGGTGGTCGTCTGGGTGGCATCGCCGGCGATGATACGCGCCGGGTCGACGACCGCCGTCATGACCGTGCTGCTGGTGGCGTTCCTGCTCGAGTACCTGCCCAAGATCTACCACTCCGTCCGCGTCCTGCGCCGGATGCAGGACGTCTCCGGCTACCTCTTCGGCACCATCTGGTGGGGGATCGCCCTCAACCTCATGGCCTACTTCGTCGCCGCTCACGCGGTGGGCGCGTGCTGGTACCTGCTCGGCGCGCAGCGAGCCACCAAGTGCCTCAGGGAGCAGTGCGCCCAGGCCGGGAGCGGGTGCGCGCCCTGGGCGCTGGCGTGCGCGGAGCCGCTCTACTACGGGCGCAGCGTGAACGTCGGGGCTGACAGGCTCGCCTGGGCCGGCAACGCCACGGCCAGGGGCACGTGCCTCGACAGCGCCGACAACTACCAGTACGGGGCCTACCAGTGGACGGTCATGCTGGTGGCCAACCCCAGCAGGGTCGAGAGGATTCTGCTCCCCATCTTCTGGGGACTAATGACTCTCAGCACCTTTGGGAATCTGGAGAGCACGACGGAGTGGCTGGAGATCGTGTTCAACATCATCACCATCACCGGCGGACTGATTCTCGTGACAATGCTCATAGGGAACATCAAGGTGTTCCTGAACGCGACGACGTCCAAGAAGCAGGCGATGCACACGCGGCTGCGGGGCGTGGAGCTGTGGATGAAGCGCAAAAACCTGCCCAGGAGCTACCGGCACCGGGTGCGGCAGTACGAGCGGCAGCGGTGGGCGGCCACGCGCGGCGTCGACGAGTGCCGCATCGTCCGTGACCTGCCGGAGGGCCTCCGCCGAGACATCAAGTACCACCTCTGCCTCGGCCTCGTGCGCCAGGTGCCACTGTTCCAACACATGGACGACCTGGTTCTCGAGAACATCTGCGACAGGGTCAAGTCCCTCATATTCCCCAAAGGAGAAGTTATTGTCAGAGAAGGGGACCCAGTGAAGAGGATGATGTTCATCGTGCGCGGCCACCTGCAGAGCAGCCAGGTGCTCCGCAACGGCGCCGAGAGCTGCTGCATGCTGGGGCCGGGCAACTTCAGCGGCGACGAGCTGCTGTCGTGGTGCCTGCGCCGGCCGTTCCTGGAGCGGCTGCCGGCGTCGTCGTCCACGCTGACCACGCTGGAGAGCACGGAGGCCTTCGGCCTGGACGCCGCGGACGTCAAGTACGTCACGCAGCACTTCCGGTACACCTTCACCAACGACAAGGTGCGGCGCAGCGCGCGCTACTACTCGCCCGGGTGGCGCACGTGGGCGGCCGTGGCGGTGCAGCTCGCGTGGCGCCGCTACAAGCACCGCAAGACGCTCGCGTCGCTGTCCTTCATCCGCCCGCGCCGCCCGCTGTCGCGGTGCTCGTCGCTCGGCGAGGAGAAGCTCCGCCTCTATACCGCGCTGCTCACGTCGCCCAAGCCCAACCAGGACGACCTG

>SsCNGC15|Sspon.03G0030490-1B

ATGCTGGTGGCCAACCCCAGCAGGGTCGAGAGGATTCTGCTCCCCATCTTCTGGGGACTAATGACTCTCAGCACCTTTGGGAATCTGGAGAGCACGACGGAGTGGCTGGAGATCGTGTTCAACATCGTCACCATCACCGGCGGACTGATTCTCGTGACAATGCTCATAGGGAACATCAAGGTGTTCCTGAACGCGACGACGTCCAAGAAGCAGGCGATGCACACGCGGCTGCGGGGCGTGGAGCTGTGGATGAAGCGCAAGAACCTGCCCAGGAGCTACCGGCACCGGGTGCGGCAGTACGAGCGGCAGCGGTGGGCGGCCACGCGCGGCGTCGACGAGTGCCGCATCGTCCGTGACCTGCCGGAGGCCTCCGCCGAGACATCAAGTACCACCTCTGCCTCGGCCTCGTGCGCCAGAGAAGGGGACCCAGTGAAGAGGATGATGTTCATCGTGCGCGGCCACCTGCAGAGCAGCCAGGTGCTCCGCAACGGCGCCGAGAGCTGCTGCATGCTGGGGCCGGGCAACTTCAGCGGCGACGAACTGCTGTCGTGGTGCCTGCGCCGGCCGTTCCTGGAGCGGCTGCCGGCGTCGTCGTCCACGCTGACCACGCTGGAGAGCACGGAGGCCTTCGGCCTGGACGCCGCGGACGTCAAGTACGTCACGCAGCACTTCCGGTACACCTTCACCAACGACAAGGTGCGGCGCAGCGCGCGCTACTACTCGCCCGGGTGGCGCACGTGGGCGGCCGTGGCGGTGCAGCTCGCGTGGCGCCGCTACAAGCACCGCAAGACGCTCGCGTCGCTGTCCTTCATCCGCCCGCGCCGGCCGCTGTCGCGGTGCTCGTCGCTCGGCGAGGAGAAGCTCCGCCTCTATACCGCGCTGCTCACGTCGCCCAAGCCCAACCAGGACGACCTG

>SsCNGC16|Sspon.07G0004720-1A

ATGATCCGCGCGGGGTTGACGACGCCGGTGATGACGGTGCTGCTGGTGTCGTTCCTGCTCGAGTACCTGCCCAAGATCTACCACGCGGCGCGCCTGCTCCGGCGGATGCAGGGCCAGTCCGGCTACATCTTCGGCACCATCTGGTGGGGCATCGCGCTCAACCTCATGGCCTACTTCGTCGCCGCCCATGCTGTGGGCGCGTGCTGGTACCTGCTCGGCGTCCAGCGGGCCAGCAAGTGCTTGAAAGAGCAGTGCCTCCAGGCGGCCGCCGGCGGGTGCGCGCGCAGCAGCGCGGTGGCCTGCGCGGCGCCGCTGTACTACGGCGGCGCCCCCTCCTCCACCGGGACCGTCGGCAGCGGCGACAGGCTCGCCTGGGCCCGGAACGCGCAGGCCCGGGGCACGTGCCTGTCCAGCGGCGGCGACAACTACCAGTACGGCGCCTACTCGTGGACGGTGATGCTGGTGGCGAACCCGAGCCGCGTGGAGCGGATGCTGCTCCCTATCTTCTGGGGCCTCATGACGCTTAGCACGTTCGGCAACCTGGAGAGCACGACGGAGTGGGTGGAGATCGTGTTCAACATCGTCACCATCACGGGGGGACTCGTCCTCGTCACCATGCTCATCGGCAACATCAAGGTGTTCCTGAACGCGACCACGTCCAAGAAGCAGGCCATGCACACGCGGCTCCGCAGCGTGGAGTGGTGGATGAAGCGCAAGAACCTGCCGCGGAGCTTCCGCGCCCGGGTGCGCCAGTTCGAGCGCCAGCGGTGGGCCGCCACGCGCGGCGTCGACGAGTGCCAGATCGTGCGCGACCTCCCCGAGGGCCTCCGCCGGGACATCAAGTACCACCTCTGCCTCGACCTCGTCCGCCAGGTCCCCTTCTTCCAGCACATGGACGACCTCGTCCTCGAGAACATCTGCGACAGGGTGAAATCCCTCATCTTCCCCAAGGGAGAAACCATCGTGAGGGAGGGCGACGTGGTGCAGCGGATGCTGTTCATCGTGCGGGGCCACCTGCAGTGCAGCCAGGTGCTGCGGAACGGCGCGACGAGCAGCTGCACGCTGGGGCCAGGCAACTTCAGCGGCGACGAGCTGCTGTCGTGGTGCCTGCGCCGCCCGTTCCTGGAGCGCCTCCCGACGTCGTCGGCGACGCTGGTGACGCTGGAGAGCACCGAGGTCTTCGGCCTGGACGCCGCCGACGTCAAGTACGTGACGCAGCACTTCCGCTACACCTTTACCAGCGACAAGGTGCGCCGCAGCGCGCGCTACTACTCGCCGGGGTGGCGCACCTGGGCGGCCGTCGCCATCCAGCTGGCATGGCGGAGGTACAAGCACCGCAAGACGCTCTCGTCGCTGTCCTTCATCCGCCCGCGGCGCCCGCTGTCCCGCTGCTCGTCGCTCGGGGAGGAGAAGCTCCGCCTGTACACGGCCATCCTCACCTCGCCCAAGCCCAACCAGGACGACGACTTCTAG

>SsCNGC16-2B|Sspon.07G0004720-2B

ATGTCAAGCGGCCTCTCCGCACGCTCGTCGCCTTCCTCCTCCACTGCGTCGCCGTCCGACGACCCGCGGCGGAAGGAGCAAGGACGACATGCGACCAGCGGCAGCGGCCGCCGGAGCCGCTGGCGGCGGCGCGTGCAGTGGCTCGGCGGCGCGGCGTGGGCGCTGGACCCGCGGGCGAGGTGGGTCCGGGACTGGAACCGCGCCTACCTGCTGGCGTGCGCGGCGGGGCTCATGGTGGACCCGCTCTTCCTGTACGCCGTGTCCCTCAGCGGCCCGCTCATGTGCCTCTTCGTCGACGGCTGGCTCGCCGCCGCCGTCACCGCGCTGCGATGCGCCGTGGACGCCATGCACGTGTGGAACGTCGCCACGCAGATCCGCATCGCGCGCGGCGCCGGCGCGGCGGCGCAGGGTAGTAAGCCCGTCGCCGGCGGCGCGGGCGACGAGGAGCAGCAGCAGCAGCAGGGCGCCGAGGAGGACGACGATGAGGAGGCCGCGCGTAAGCTCCCCGAAGACGCGACGCCCAGGAAAGGGATGCTGCTGGACTTCTTCGTCATCCTTCCCGTGATGCAGGTGGTGGTGTGGGTTGCGGCGCCGGCGATGATCCGCGCGGGGTTGACGACGCCGGTGATGACGGTGCTGCTGGTGTCGTTCCTGCTCGAGTACCTGCCCAAGATCTACCACGCGGCGCGCCTGCTCCGGCGGATGCAGGGCCAGTCCGGCTACATCTTCGGCACCATCTGGTGGGGCATCGCGCTCAACCTCATGGCCTACTTCGTCGCCGCCCATGCTGTGGGCGCGTGCTGGTACCTGCTCGGCGTCCAGCGGGCCAGCAAGTGCTTGAAAGAGCAGTGCCTCCAGGCGGCCGCCGGCGGGTGCGCGCGCAGCAGCGCGGTGGCCTGCGCGGCGCCGCTGTACTACGGCGGGGCCCCCTCCTCCACCGGGACCGTCGGCAGCGGCGACAGGCTCGCCTGGGCCCGGAACGCGCAGGCCCGGGGCACGTGCCTGTCCAGCGGCGGCGACAACTACCAGTACGGCGCCTACTCGTGGACGGTGATGCTGGTGGCGAACCCGAGCCGCGTGGAGCGGATGCTGCTCCCTATCTTCTGGGGCCTCATGACGCTTAGCACGTTCGGCAACCTGGAGAGCACGACGGAGTGGGTGGAGATCGTGTTCAACATCGTCACCATCACGGGGGGACTCGTCCTCGTCACCATGCTCATCGGCAACATCAAGGTGTTCCTGAACGCGACCACGTCCAAGAAGCAGGCCATGCACACGCGGCTCCGCAGCGTGGAGTGGTGGATGAAGCGCAAGAACCTGCCGCGGAGCTTCCGCGCCCGGGTGCGCCAGTTCGAGCGCCAGCGGTGGGCCGCCACGCGCGGCGTCGACGAGTGCCAGATCGTGCGCGACCTCCCCGAGGGCCTCCGCCGGGACATCAAGTACCACCTCTGCCTCGACCTCGTCCGCCAGGTCCCCTTCTTCCAGCACATGGACGACCTCGTCCTCGAGAACATCTGCGACAGGGTGAAATCCCTCATCTTCCCCAAGGGAGAAACCATCGTGAGGGAGGGCGACGTGGTGCAGCGGATGCTGTTCATCGTGCGGGGCCACCTGCAGTGCAGCCAGGTGCTGCGGAATGGCGCGACGAGCAGCTGCACGCTGGGGCCAGGCAACTTCAGCGGCGACGAGCTGCTGTCGTGGTGCCTGCGCCGCCCGTTCCTGGAGCGCCTCCCGACGTCGTCGGCGACGCTGGTGACGCTGGAGAGCACCGAGGTCTTCGGCCTGGACGCCGCCGACGTCAAGTACGTGACGCAGCACTTCCGCTACACCTTCACCAACGACAAGGTGCGCCGCAGCGCGCGCTACTACTCGCCGGGGTGGCGCACCTGGGCGGCCGTCGCCATCCAGCTGGCATGGCGGAGGTACAAGCACCGCAAGACGCTCTCGTCGCTGTCCTTCATCCGCCCGCGGCGCCCGCTGTCCCGCTGCTCGTCGCTCGGGGAGGAGAAGCTCCGCCTGTACACGGCCATCCTCACCTCGCCCAAGCCCAACCAGGACGACGACTTCTAG

>SsCNGC16-3C|Sspon.07G0004720-3C

ATGTCAAGCGGCCTCTCCGCACGCTCGTCGCCTTCCTCCTCCACTGCGTCGCCGTCCGACGACCCGCGGCGGAAGGAGCAAGGACGACATGCGACCAGCGGCAGCGGCCGCCGGAGCCGCTGGCGGCGGCGCGTGCAGTGGCTCGGCGGCGCGGCGTGGGCGCTGGACCCGCGGGCGAGGTGGGTCCGGGACTGGAACCGCGCCTACCTGCTGGCGTGCGCGGCGGGGCTCATGGTGGACCCGCTCTTCCTGTACGCCGTGTCCCTCAGCGGCCCGCTCATGTGCCTCTTCGTCGACGGCTGGCTCGCCGCCGCCGTCACCGCGCTGCGATGCGCCGTGGACGCCATGCACGTGTGGAACGTCGCCACGCAGCTCCGCATCGCGCGCGGCGCCGGCGCGGCGGCGCAGGGTAGTAAGCCCGTCGCCGGCGGCGCGGGCGACGAGGAGCAGCAGCAGCAGGGCGCCGAGGAGGACGACGATGAGGAGGCCGCGCGTAAGCTCCCCGAAGACGCGACGCCCAGGAAAGGGATGCTGCTGGACTTCTTCGTCATCCTCCCCGTGATGCAGGTGGTGGTGTGGGTTGCAGCGCCTGCGATGATCCGCGCGGGGTTGACGACGCCGGTGATGACGGTGCTGCTGGTGTCGTTCCTGCTCGAGTACCTGCCCAAGATCTACCACGCGGCGCGCCTGCTCCGGCGGATGCAGGGCCAGTCCGGCTACATCTTCGGCACCATCTGGTGGGGCATCGCGCTCAACCTCATGGCCTACTTCGTCGCCGCCCATGCTGTGGGCGCGTGCTGGTACCTGCTCGGCGTCCAGCGGGCCAGCAAGTGCCTGAAAGAGCAGTGCCTCCAGGCGGCGGCCGGCGGGTGCGCGCGCAGCAGCGCGGTGGCCTGCGCGGCGCCGCTGTACTACGGCGGCTCCCCCTCCTCCGCCGGGACCGTCGGCAGCGGCGACAGGCTCGCCTGGGCCCGGAACGCGCAGGCCCGGGGCACGTGCCTGTCCAGCGGCGGCGACAACTACCAGTACGGCGCCTACTCGTGGACGGTGATGCTGGTGGCGAACCCGAGCCGCGTGGAGCGGATGCTGCTCCCGATCTTCTGGGGCCTCATGACGCTCAGCACGTTCGGCAACCTGGAGAGCACGACGGAGTGGGTGGAGATCGTGTTCAACATCGTCACCATCACGGGGGGACTCGTCCTCGTCACCATGCTCATCGGCAACATCAAGGTGTTCCTGAACGCGACCACGTCCAAGAAGCAGGCCATGCACTCGCGCCTCCGCAGCGTGGAGTGGTGGATGAAGCGCAAGAACCTGCCGCGGAGCTTCCGCGCCCGGGTGCGCCAGTTCGAGCGCCAGCGGTGGGCCGCCACGCGCGGCGTCGACGAGTGCCAGATCGTGCGCGACCTCCCCGAGGGCCTCCGCCGGGACATCAAGTACCACCTCTGCCTCGACCTCGTCCGCCAGGTCCCCTTCTTCCAGCACATGGACGACCTCGTCCTCGAGAACATCTGCGACAGGGTGAAATCCCTCATCTTCCCCAAGGGAGAAACCATCGTGAGGGAGGGCGACGTGGTGCAGCGGATGCTGTTCATCGTGCGGGGCCACCTGCAGTGCAGCCAGGTGCTGCGGAACGGCGCGACGAGCAGCTGCACGCTGGGGCCAGGCAACTTCAGCGGCGACGAGCTGCTGTCGTGGTGCCTGCGCCGCCCGTTCCTGGAGCGCCTCCCGACGTCGTCGGCGACGCTGGTGACGCTGGAGAGCACCGAGGTCTTCGGCCTGGACGCCGCCGACGTCAAGTACGTGACGCAGCACTTCCGCTACACCTTCACCAACGACAAGGTGCGCCGCAGCGCGCGCTACTACTCGCCCGGGTGGCGCACCTGGGCGGCCGTCGCCATCCAGCTGGCATGGCGGAGGTACAAGCACCGCAAGACGCTCTCGTCGCTGTCCTTCATCCGCCCGCGGCGCCCGCTGTCCCGCTGCTCGTCGCTCGGGGAGGAGAAGCTCCGCCTGTACACGGCCATCCTCACCTCGCCCAAGCCCAACCAGGACGACGACTTCTAG

>SsCNGC16-4D|Sspon.07G0004720-4D

ATGTCAAGCGGCCTCTCCGCACGCTCGTCGCCTTCCTCCTCCACTGCGTCGCCGTCCGACGACCCGCGGCGGAAGGAGCAAGGACGACATGCGACCAGCGGCAGCGGCCGCCGGAGCCGCTGGCGGCGGCGCGTGCAGTGGCTCGGCGGCGCGGCGTGGGCGCTGGACCCGCGGGCGAGGTGGGTCCGGGACTGGAACCGCGCCTACCTGCTGGCGTGCGCGGCGGGGCTCATGGTGGACCCGCTCTTCCTGTACGCCGTGTCCCTCAGCGGCCCGCTCATGTGCCTCTTCGTCGACGGCTGGCTCGCCGCCGCCGTCACCGCGCTGCGATGCGCCGTGGACGCCATGCACGTGTGGAACGTCGCCACGCAGATCCGCATCGCGCGCGGCGCCGGCGCGGCGGCGCAGGGTAGTAAGCCCGTCGCCGGCGGCGCGGGCGACGAGGAGGAGCAGCAGCAGGGCGCCGAGGAGGACGACGATGAGGAGGCCGCGCGTAAGCTCCCCGATGACGCGACGCCCAGGAAAGGGATGCTGCTGGACTTCTTCGTCATCCTTCCCGTGATGCAGAACGGAGCTCACCGGGGCGAGGAAGAGGCCAGAAGAGCTTATTCCCCTACGCCATCGCTGCCTCAGCCTCGCCGACGCCGCGCCGCCAAGAAAAGCAAGCCACCGAGGATTGACAACCCCCAACAAACCCTAGCTCCACGGACAAGAAACGACGGCGAGCAAAACCTAGGGCTAGTGGTGGTGTGGGTTGCGGCGCCGGCGATGATCCGCGCGGGGTTGACGACGCCGGTGATGACGGTGCTGCTGGTGTCGTTCCTGCTCGAGTACCTGCCCAAGATCTACCACGCGGCGCGCCTGCTCCGGCGGATGCAGGGCCAGTCCGGCTACATCTTCGGCACCATCTGGTGGGGCATCGCGCTCAACCTCATGGCCTACTTCGTCGCCGCCCATGCTGTGGGCGCGTGCTGGTACCTGCTCGGCGTCCAGCGGGCCAGCAAGTGCCTGAAAGAGCAGTGCCTCCAGGCGGCCGGCGGCGGGTGCGCGCGCAGCAGCGCGGTGGCCTGCGCGGCGCCGCTGTACTACGGCGGCTCCCCCTCCTCCACCGGGACCGTCGGCCGCGGCGACAGGCTCGCCTGGGCCCGGAACGCGCAGGCCCGGGGCACGTGCCTGTCCAGCGGCGGCGACAACTACCAGTACGGCGCCTACTCGTGGACGGTGATGCTGGTGGCGAACCCGAGCCGCGTGGAGCGGATGCTGCTCCCTATCTTCTGGGGCCTCATGACGCTCAGCACGTTCGGCAACCTGGAGAGCACGACGGAGTGGGTGGAGATCGTGTTCAACATCGTCACCATCACGGGGGGACTCGTCCTCGTCACCATGCTCATCGGCAACATCAAGGTGTTCCTGAACGCGACCACGTCCAAGAAGCAGGCCATGCACACGCGGCTCCGCAGCGTGGAGTGGTGGATGAAGCGCAAGAACCTGCCGCGGAGCTTCCGCGCCCGGGTGCGCCAGTTCGAGCGCCAGCGGTGGGCCGCCACGCGCGGCGTCGACGAGTGCCAGATCGTGCGCGACCTCCCCGAGGGCCTCCGCCGGGACATCAAGTACCACCTCTGCCTCGACCTCGTCCGCCAGGTCCCCTTCTTCCAGCACATGGACGACCTCGTCCTCGAGAACATCTGCGACAGGGTGAAGTCCCTCATCTTCCCCAAGGGAGAAACCATCGTGAGGGAGGGCGACGTGGTGCAGCGGATGCTGTTCATCGTGCGGGGCCACCTGCAGTGCAGCCAGGTGCTGCGGAACGGCGCGACGAGCAGCTGCACGCTGGGGCCAGGCAACTTCAGCGGCGACGAGCTGCTGTCGTGGTGCCTGCGCCGCCCGTTCCTGGAGCGCCTCCCGACGTCGTCGGCGACGCTGGTGACGCTGGAGAGCACCGAGGTCTTCGGTCTGGACGCCGCCGACGTCAAGTACGTGACGCAGCACTTCCGCTACACCTTCACCAACGACAAGGTGCGCCGCAGCGCGCGCTACTACTCGCCGGGGTGGCGCACCTGGGCGGCCGTCGCCATCCAGCTGGCATGGCGGAGGTACAAGCACCGCAAGACGCTCTCGTCGCTGTCCTTCATCCGCCCGCGGCGCCCGCTGTCCCGCTGCTCGTCGCTCGGGGAGGAGAAGCTCCGCCTGTACACGGCCATCCTCACCTCGCCCAAGCCTAACCAGGACGACGACTTCTAG
