# Supplementary material for: Genome-wide identification and expression analysis of the cyclic nucleotide-gated ion channel (CNGC) gene family in Saccharum spontaneum
Source: BMC Genomics. 2023 May 25;24:281. doi: 10.1186/s12864-023-09307-3 (PMC10214738; doi:10.1186/s12864-023-09307-3)
Supplement: Supplementary file 3 — Additional file 3: Supplementary file 2. Amino acid sequences of SsCNGCs and alleles. [file 12864_2023_9307_MOESM3_ESM.docx]

Supplementary file 2: Amino acid sequences of SsCNGCs and allels.

>SsCNGC1|Sspon.04G0014070-1A

MLRNAKISWNGKQNSRKLHGEEVGGKVEDLISYCLHCWPVLLWSYKEAMAGREERYVRVNSERGHNIFGLLKDRTAGAFSFLGNSSHSEALNKSGPEEKKSKTRVLDPQGPFLQRWNKIFVISCLFAVFVDPLFLYIPVIDGGNNCLYLDKKLETVASILRFFTDIFYLLHMLFQFRTGFIAPSSRVFGRGVLVKDTFAIAKRYISTLFLVDLLAVLPLPQVFVLVVLPTLQGPEVMKAKNVLLYVPRLLRIIPLYLQITRSAGILTETAWAGAAFNLIIYMLASHGFGALWYILSIQREDTCWRQACINQTGCELTSLYCGYHPLTNNSFLQSACPTNSTSNPNPDPKFGIFLPALQNVSQSTSFFEKLFYCFWWGLQNLSSLGQNMKTSTNTLENLFAVFVSTSGLVLFALLIGNVQTYLQSASVRIEEMRVKRRDTEQWMAHRLLPENLKDRIMRHEQYRWQETRGVDEEGLLKNLPKDLRREIKRHLCLSLLMKVPMFENMDEQLLDAMCDRLKPMLYTEGSCIIREGDPVNEMLFIMRGTLESTTTNGGQTGFFNSNVLKGGDFCGEELLTWALDPTSASNLPGSTRTVKTLSEVEAFALRADDLKFVATQFRRLHSKQLQHTFRFYSQQWRTWAACFIQAAWHRYCRKKLEEALYEKEKRLQAAIVSDGTTSLSLGAALYASRFAGNMMRILRRNATRKARLQERVPARLLQKPAEPNFFAEDS*

>SsCNGC1-2C|Sspon.04G0014070-2C

MFENMDEQLLDAMCDRLKPMLYTEGSCIIREGDPVNEMLFIMRGTLESTTTNGGQTGFFNSNVLKGGDFCGEELLTWALDPTSASNLPGSTRTVKTLSEVEAFALRADDLKFVATQFRRLHSKQLQHTFRFYSQQWRTWAACFIQAAWHRYCRKKLEEALYEKEKRLQAAIVSDGTTSLSLGAALYASRFAGNMMRILRRNATRKARLQERVPARLLQKPAEPNFFAEDS

>SsCNGC1-1P|Sspon.04G0014070-1P

MAGREERYVRVNSERGHNIFGLLKDRTAGAFSFLGNSSHSEALNKSGPEEKKSKTRVLDPQGPFLQRWNKIFVISCLFAVFVDPLFLYIPVIDGGNNCLYLDKKLETVASILRFFTDIFYLLHMLFQFRTGFIAPSSRVFGRGVLVKDTFAIAKRYISTLFLVDLLAVLPLPQVFVLVVLPTLQGPEVMKAKIYYWLLLFYVPRLLRIIPLYLQITRSAGILTETAWAGAAFNLIIYMLASHGFGALWYILSIQREDTCWRQACINQTGCELTSLYCGYHPLTNNSFLQSACPTNSTSNPNPDPKFGIFLPALQNVSQSTSFFEKLFYCFWWGLQNLSSLGQNMKTSTDTLENLFAVFVSTSGLVLFALLIGNVQTYLQSASVRIEEMRVKRRDTEQWMAHRLLPENLKDRIMRHEQYRWQETRGVDEEGLLKNLPKDLRREIKRHLCLSLLMKVPMFENMDEQLLDAMCDRLKPMLYTEGSCIIREGDPVNEMLFIMRGTLESTTTNGGQTGFFNSNVLKGGDFCGEELLTWALDPTSASNLPGSTRTVKTLSEVEAFALRADDLKFVATQFRRLHSKQLQHTFRFYSQQWRTWAACFIQAAWHRYCRKKLEEALYEKEKRLQAAIVSDGTTSLSLGAALYASRFAGNMMRILRRNATRKARLQERVPARLLQKPAEPNFFAEDS*

>SsCNGC2-1A|Sspon.08G0008040-1A

MRRRPRDRKREASSHFSPNSTRLPSPASKGKGRARVPKSKPELNLKKMMMGREDKYVRFQDWRSEQSVSSENIVAPYRDDVSVFSSLKERTARVFALLGNLLHSETSNRSMLDERKSATGTLHPQGPFLQKWNRIFVISCIFAVSVDPLFLYIPVISDEKPCWYLDRKLEKAASVLRFFTDIFYILHIIFQFRTGFIASSPTTFGRGVLIEDRYAITKRYLSTYFFIDVFAILPIPQVIILVLLPNLQGLKIMKAKNVLLLIIICQYVPRLIRIRPLYLQITRSAGVITETARAGAAFNLLLYMLASHKFVKNSTRDKRGILHLQSADYEQRPVSGTQTQPKR*

>SsCNGC2|Sspon.08G0008040-1P

MPLPGRAVLLAAAVESTPYLPLPPRPVHPPHAAKTVTVSAKLHRISPQTPPAYPARLEGERTRPRPQVQTRGRKKVWPHAEGSGGNEFTKAKFEENDDGKRGQICEISGLEIRASVSSENIVAPYRDDVSVFSSLKERTARVFALLGNLLHSETSNRSMLDERKSATGTLHPQGPFLQKWNRIFVISCIFAVSVDPLFLYIPVISDEKPCWYLDRKLEKAASVLRFFTDIFYILHIIFQFRTGFIASSPTTFGRGVLIEDRYAITKRYLSTYFFIDVFAILPIPQVIILVLLPNLQGSKIMKAKNVLLLIIICQYVPRLIRIRPLYLQITRSAGVITETARAGAAFNLLLYMLASHKFVKNSTRDKRAILHLQSADYEQRPVLGALWYLLSIQRQDSCWRQQCRSNPTCDLAYLYCGDYDNNVKNTFLTTICLPSNQSNLPDPYFGIYAPAIKNVSQSKSFFAKLFFCVWWGLQNLSSLGQNLKTSTYAWENLFAVFVSISGLVLFALLIGNVQTYLQSASLRIEEMRVKSRDTDQWMSYRHLPENLKERIRRYEQYRWQETSGVDEEQLLMNLPKDLRRDIKRHLCLSLLMRVPLFENMDDQLLDAMCDCLKPILYTEGSCVIREGDPVNEMLFVMRGNLMSMTTNGGRTGFFNSDVLKAGDFCGEELLTWALDPTSTSSLPSSTRTVKTMSEVEAFALRAEDLRFVATQFRRLHSKQLQHTFRFYSQQWRTWAACFIQAAWHRYCRKKIEDSLREKEKRLQFAIANDSSTSLSFMAALYASRFAGNMIRILRRNATRKARLQERVPARLLQKPAEPNFSAEEQ*

>SsCNGC2-2B|Sspon.08G0008040-2B

FQDWRSEQSVSSENIVAPYRDDVSVFSSLKERTARVFALLGNLLHSETSNRSMLDERKSATGTLHPQGPFLQKWNRIFVISCIFAVSVDPLFLYIPVISDEKPCWYLDRKLEKAASVLRFFTDIFYILHIIFQFRTGFIASSPTTFGRGVLIEDRYAITKRYLSTYFFIDVFAILPIPQVIILVLLPNLQGSKIMKAKNVLLLIIICQYVPRLIRIRPLYLQITRSAGVITETARAGAAFNLLLYMLASHKFVKNSTRDKRESYTCRVQIMNKDLSQALRHNR

>SsCNGC3|Sspon.04G0014080-1A

MKARARVFDPRGPFLKKWNKIFVISCLVSVSVDSLFFYAPAIDGDNSCLYLDDNLQKIASILRSLTDAFYLLRVIYQFRTGFAAPSSSGAFGRGVLVDDMLAIAKRYLSTYFLIDILSILPLPQTYLQSASGHIEEMRVIRRDTEQWMAYRLLPEHIKQRILRHDQYRWQETQGMDEEGLLINLPKDLRRDIKRHLCLSLLMRVPMFENMDDQLLDAMCDRVKPMLYTEGSCIIREGDPVNEMFFIMRGRLESMTTDGGRTGFFNSNVLQGGDFCGEELLTWALDPASGSNLPSSTRTVKTLSELEGFALRAHHLKFVANQYRRLHSKQLRHTFRFYSQQWRTWAACFIQAAWHRYCRRKMEDSLHEKERMFQAAIVTDASSSCSLGAALYAAHFASNM

>SsCNGC3-2B|Sspon.04G0014080-2B

MKARARVLDPRGPFLQKWNKIFVISCLVSVSVDSLFFYAPAIDGDNSCLYLDDNLQKITSILRSLTDAFYLLRVIFQFRTGFAAPSSSGAFGRGVLVDDMLAIAKRSLELFGTFLPYNEKTPVGEKLVITRMVVIWQLYIVETYLQSASGHIEEMRVIRRDTEQWIAYRLLPEHIKQRILRHDQYRWQETQGVDEEGLLINLPKDLRRDIKRHLCLSLLMRVPMFENMDDQLLDAMCDRVKPMLYTEGSCIIREGDPVNEMFFIMRGRLESMTTDGGRTGFFNSNVLQGGDFCGEELLTWALDPASGSNLPSSTRTVKTLSELEGFALRAHHLKFVANQYRRLHSKQLRHTFRFYSQQWRTWAACFIQAAWHRYCRRKLEDSLHEKERMFQAAIVTDASSSCSLGAALYAAHFASNM

>SsCNGC4|Sspon.05G0022360-1B

MPFVIRSPPPLSQRLEESSPRSSVPSEVGGRSTLRSSMPGFGYGSFNALRSFLSGVCKSSGRLKSLGQSLTSGAPKTAFAEDLKSYKRTIFDPQDKILFQMNWVFFSSCLFAVAVDPLFFFLPIINDSNCIGIDKKLAVTSTIIRTVIDFVYLIRVCLQFRTAYVAPSSRVFGTGELVIDPMLIAKRYIKSYFAMDFVALLPLPQIVVWRYLNIPDGPDVLTTKTALVWVVLIQYIPRLFRIFPVTTDLKRTAGVFIETAWAGAAYYLLWFMLAGHNVGTLWYFLTIEREDDCWHLYCDPNVGCNSSYLYCNNNHHGSYDSWLKTNGAQVFNICNGTQDNFNFGIYQQALVSGILRPGNFISKLCYCFWWGLQNLSTLGQGLLTSTYTGEVIFSIAICVLGLILFALLIGNMQSYLQSVAIRLEEMRVKKRDAEQWMHHRSLPPEIRHRVRKYERYRWLETRGVDEESLVQTLPKDLRRDIKRHLCLGLVKRVPLFENMDERLLDAICERLRPALYTENEFILREGDPVDEMHFILHGCLESVTTDGGRSGFFNKVQLKEGSFCGDELLTWALDPKSAANFPVSSRTVKALTEVEAFALCAEELKFVASQFRRLHSRQVQHTFRFYSQQWRTWAACFIQAAWRRYYKRKMAEQRRKEEEAASRPSSSHPSLGATIYASRFAANAMRGVHRLRSKAVPTIVRLPKPPEPDFGVDDAD*

>SsCNGC4-2D|Sspon.05G0022360-2D

MAVLEESSPRSSVPSEVGGRSTLRSSMPGFGSSFNALRSFLSGVRKGSGRLKSLGQSLTSGAPKTAFAEDLKSYKRTIFDPQDKILFQMNWVFFSSCLFAVAVDPLFFFLPIINDSNCIGIDKKLAVTSTIIRTVIDFVYLIRVCLQFRTAYVAPSSRVFGTGELVIDPMLIAKRYIKSYFAMDFVALLPLPQIVVWRYLNIPDGPDVLTTKTALVWVVLIQYIPRLFRIFPVITDLKRTAGVFIETAWAGAAYYLLWFMLAGHSYLQSVAIRLEEMRVKKRDAEQWMHHRSLPPEMRHRVRKYERYRWLETRGVDEESLVQTLPKDLRRDIKRHLCLGLVKRVPLFENMDERLLDAICERLRPALYTENEFILREGDPVDEMHFILHGCLESVTTDGGRSGFFNKVQLKEGSFCGDELLTWALDPKSAANFPVSSRTVKALTEVEAFALCAEELKFVASQFRRLHSRQVQHTFRFYSQQWRTWAACFIQAAWRRYYKRKMAEQRRKEEEAASRPSSSHPSLGATIYASRFAANAMRGVHRLRSKAVPTIVRLPKPPEPDFGLDDAD*

>SsCNGC5|Sspon.01G0024280-1A

MSYDQSAFQMDYVGVGAGAGVGVSASRRRFMPSESLARGVITHGSAQLRTIGRSIRAGATMAAVFQEDLKNTSRRIFDPQDPVLVRLNRAFLISCIVAIAVDPMFFYLPMVTDEGNLCVGIDRWLAVSTTVVRSVVDLFFVGRIALQFRTAYIKPSSRVFGRGELVIDTALIARRYMRRFFSADLASVLPLPQVVIWKFLHRSKGTAVLDTKNSLLFIVFIQYVPRVVRIYPISSELKRTSGVFAETAYAGAAYYLLWYMLASHIVGAFWYLLSIERVSDCWRNACDEFPGCNQIYMYCGNDRHLGFLEWRTITRQHPPFNYGIYSPAVTSDVLKTKDTTSKLLFCLWWGLANLSTLGQGLKTSIYTGEALFSIALAIFGLILMAMLIGNIQTYLQSLTVRLEEMRVKQRDSEQWMHHRLLPPELRERVRRYDQYKWLNTHGVDEEALVQNLPKDLRRDIKRHLCLGLVRRVPLFANMDERLLDAICERLKPSLCTERTYITREGDPVDQMVFIIRGSLESITTDGGRTGFYNRSLLEEGDFCGEELLTWALDPKAGACLPSSTRTVMALSEVEAFALHAEELKFVAGQFRRMHSKAVQHTFRFYSQQWRTWAATYIQAAWRRHLKRRAAELRRREDEEMEEDEGKSNRIRTTILVSRFAANAMRGVHRQRSRRAVAVPVPVPELLMPMPKPREPDFRDDY*

>SsCNGC5-2B|Sspon.01G0024280-2B

MSYDQSAFQMDYVGVGAGAGVGVSASRRRFMPSESLARGVITHGSAQLRTIGRSIRAGATMAAVFQEDLKNTSRRIFDPQDPVLVRLNRAFLISCIVAIAVDPMFFYLPMVTDEGNLCVGIDRWLAVSTTVVRSVVDLFFVGRIALQFRTAYIKPSSRVFGRGELVVIWKFLHRSKGTAVLDTKNSLLFIVFIQYVPRVVRIYPISSELKRTSGVFAETAYAGAAYYLLWYMLASHIVGAFWYLLSIERVSDCWRNACDEFPGCNQIYMYCGNDRHLGFLEWRTITRQVINETCEPKQDGSIPPFNYGIYSPAVTSDVLKTKDTTSKLLFCLWWGLANLSTLGQGLKTSIYTGEALFSIALAIFGLILMAMLIGNIQTYLQSLTVRLEEMRVKQRDSEQWMHHRLLPPELRERVRRYDQYKWLNTHGVDEEALVQNLPKDLRRDIKRHLCLGLVRRVPLFANMDERLLDAICERLKPSLCTERTYITREGDPVDQMVFIIRGSLESITTDGGRTGFYNRSLLEEGDFCGEELLTWALDPKAGACLPSSTRTVMALSEVEAFALHAEELKFVAGQFRRMHSKAVQHTFRFYSQQWRTWAATYIQAAWRRHLKRRAAELRRREDEEIEEDEGKSNRIRTTILVSRFAANAMRGVHRQRSRRAVAVAVPELLMPMPKPREPDFRDDY*

>SsCNGC5-3C|Sspon.01G0024280-3C

MSYDQSAFQMDYVGVGAGAGVGVSASRRRFMPSESLARGVITHGSAQLRTIGRSIRAGATMAAVFQEDLKNTSRRIFDPQDPVLVRLNRAFLISCIVAIAVDPMFFYLPMVTDEGNLCVGIDRWLAVSTTVVRSVVDLFFVGRIALQFRTAYIKPSSRVFGRGELVIDTALIARRYMRRFFSADLASVLPLPQVVIWKFLHRSKGTAVLDTKNSLLFIVFIQYVPRVVRIYPISSELKRTSGVFAETAYAGAAYYLLWYMLASHIVGAFWYLLSIERVSDCWRNACDEFPGCNQIYMYCGNDRHLGFLEWRTITRQVINETCEPKQDGSIPFNYGIYSPAVTSDVLKTKDTTSKLLFCLWWGLANLSTLGQGLKTSIYTGEALFSIALAIFGLILMAMLIGNIQTYLQSLTVRLEEMRVKQRDSEQWMHHRLLPPELRERVRRYDQYKWLNTHGVDEEALVQNLPKDLRRDIKRHLCLGLVRRVPLFANMDERLLDAICERLKPSLCTERTYITREGDPVDQMVFIIRGSLESITTDGGRTGFYNRSLLEEGDFCGEELLTWALDPKAGACLPSSTRTVMALSEVEAFALHAEELKFVAGQFRRMHSKAVQHTFRFYSQQWRTWAATYIQAAWRRHLKRRAAELRRREDEEMEEDEGKSNRIRTTILVSRFAANAMRGVHRQRSRRAVPELLMPMPKPREPDFRDDY

>SsCNGC5-4D|Sspon.01G0024280-4D

MSYDQSAFQMDYVGVGAGAGVGVSASRRRFMPSESLARGVITHGSAQLRTIGRSIRAGATMAAVFQEDLKNTSRRIFDPQDPVLVRLNRAFLISCIVAIAVDPMFFYLPMVTDEGNLCVGIDRWLAVSTTVVRSVVDLFFVGRIALQFRTAYIKPSSRVFGRGELVIDTALIARRYMRRFFSADLASVLPLPQVVIWKFLHRSKGTAVLDTKNSLLFIVFIQYVPRVVRIYPISSELKRTSGVFAETAYAGAAYYLLWYMLASHIVGAFWYLLSIERVSDCWRNACDEFPGCNQIYMYCGNDRHLGFLEWRTITRQVINETCEPKQDGSIPPFNYGIYSPAVTSDVLKTKDTTSKLLFCLWWGLANLSTLGQGLKTSIYTGEALFSIALAIFGLILMAMLIGNIQTYLQSLTVRLEEMRVKQRDSEQWMHHRLLPPELRERVRRYDQYKWLNTHGVDEEALVQNLPKDLRRDIKRHLCLGLVRRVPLFANMDERLLDAICERLKPSLCTERTYITREGDPVDQMVFIIRGSLESITTDGGRTGFYNRSLLEEGDFCGEELLTWALDPKAGACLPSSTRTVMALSEVEAFALHAEELKFVAGQFRRMHSKAVQHTFRFYSQQWRTWAATYIQAAWRRHLKRRAAELRRREDEEMEEDEGKSNRIRTTILVSRFAANAMRGVHRQRSRRAVPELLMPMPKPREPDFRDDY*

>SsCNGC6|Sspon.02G0031610-1A

MSGCGYRTQFINGRREKFVRLVEADEPAESATSPTSGSGGATMEHGHGNGGGHGGGGGGFHMDSYFSGNPSAAAAKFRARSVRVAAGVKNRSERLRSIGLVFQEDFRKMSQQVFDPQDAFLARMNRAFVFACIVSVAIDPLFLYLLAVKYTDKNTCIGFDRNLATVATVVRTAVDAFYLARIALQFRTAYIAPSSRVFGRGELVIDSSAIARRYLRRFFVVDLLSVLPLPQVSIWNFLNRPKGADLLPTKNALLFTVLSQYVPRLVRFYPITSELKRTTGVFAETAFGGAAFYLLLYMLASHMVGAFWYLLAIERLDDCWRDKCTKLNFHQCRTYMYCGGGSQGQSGFLEWRTMIRQVLAQECAPVDGSGTGFPYGIYTTAIQSGVYSTENLTAKILFCLWWGLQNLSTIGQGLETTHYKGEQLFSITLALLGLILMALLIGNMQTYLQSMTLRLEEMRLKRRDSEQWMHHRVLPDELRERVWRHNQYKWLETRGVDEDSLVRSLPKDLRRDVKRHLCLRLVRRVPLFANMDERLLDAICERLKPSLCTESTYIVREGDPVDEMLFIIRGRLESSTTDGGRMGFYNRGLLKEGDFCGEELLTWALDPKAGTNFPLSTRTVRAISEVEAFALRADELKFVAGQFRRLHSKQLQQTFRFYSQQWRTWASCLIQAAWRRYLKRKAAEQRRREEEMEADEAAASGVSTSRFKTTLLVSRFAKNAMRGVQRQRSVRADSLIMLPRPPEPDFGSMDY*

>SsCNGC6-2D|Sspon.02G0031610-2D

MFGCGYRTQFINGRREKFVRLVEADAPAESATSPTSGSGGATMEHGHGNGGGHGGGGGGFHMDSYFSGNPSAAAAKFRARSVRVAAGVMNRSERLRSIGLVFQEDFRKMSQQVFDPQDAFLARMNRAFVFACIVSVAIDPLFLYLLAVKYTDKNTCIGFDRNLATVATVVRTAVDAFYLARIALQFRTAYIAPSSRVFGRGELVIDSSAIARRYLRRFFVVDLLSVLPLPQVSIWNFLNRPKGADLLPTKNALLFTVLSQYVPRLVRFYPITSELKRTTGVFAETAFGGAAFYLLLYMLASHMVGAFWYLLAIERLDDCWRDKCTKLNFHQCRTYMYCGGGSQGQSGFLEWRTMIRQVLAQECAPVDGSGTGFPYGIYTTAIQSGVYSTENLTAKILFCLWWGLQNLSTIGQGLETTHYKGEQLFSITLALLGLILMALLIGNMQTYLQSMTLRLEEMRLKRRDSEQWMHHRVLPDELRERVWRHNQYKWLETRGVDEDSLVRSLPKDLRRDVKRHLCLRLVRRVPLFANMDERLLDAICERLKPSLCTESTYIVREGDPVDEMLFIIRGRLESSTTDGGRMGFYNRGLLKEGDFCGEELLTWALDPKAGTNFPLSTRTVRAISEVEAFALRADELKFVAGQFRRLHSKQLQQTFRFYSQQWRTWASCLIQAAWRRYLKRKAAEQRRREEEMEADEAAASGVSTSRFKTTLLVSRFAKNAMRGVQRQRSVRADSLIMLPRPPEPDFGSMDY*

>SsCNGC7|Sspon.04G0002010-1A

MDCDLFAAWWSSSTRLVSRIFRGSAADAPGPSPMRPAIPLHQKQAGLAASKLGVGTSKKHRAFVASDEQWYNKIFDPSSDFILTWNRIFLFSCFVALFIDPLYFYVPKISYGSPKFCVGTDTRFAVGVTFFRSIADLLYVLHIIIKFRTAYINPSSTLRVFGRGDLVTNPKEIACKYIRSDLVVDVAAALPLPQIIVWFVIPAIKYSSAEHNNNILVLIVLAQYLPRLYLIFPLTYEIVKATGVVAKTAWEGAAYNMVLYLIASHVLGALWYLLSVDRQTFCWKTSCLSETDCHIKYLDCDTTLNATWASTTAVFSKCNASDDTISFDFGMFGPALSNQAPAQSFAMKYFYSLWWGLQNLSCYGQTLSVSTYLGETLYCIFLAVLGLVLFAHLIGNVQTYLQSITVRVEEWRLKQRDTEEWMRHRQLPCELRERVRRFIQYKWLATRGVNEESILQALPADLRRDIKRHLCLGLVRRVPFFAQMDDQLLDAICERLVSSLCTKGTYIVREGDPVTEMLFIIRGKLESSTTNGGRTGFFNSITLKPGDFCGEELLGWALVPRPTTNLPSSTRTVKALIEVEAFALQAEDLKFVASQFRRLHSKKLQHTFRYYSHHWRTWASCFIQAAWRRYKRRKMAKDLSMRESFNSVRLDEVDNEDDDSPPKNNLALKFIARTRKVPQNMKGLPKLTKPDEPDFSAEPED*

>SsCNGC7-2B|Sspon.04G0002010-2B

MDCDLFAAWWSSSTRLVSRIFRGSAADAPGPSPLRPAIPLHQKQAGLAASKLGVGTSKKHRAFVASDEQWYNKIFDPSSDFILTWNRIFLFSCFVALFIDPLYFYVPKISYGSPKFCVGTDTRFAVGVTFFRSIADLLYVLHIIIKFRTAYINPSSTLRVFGRGDLVTNPKEIACKYIRSDLVVDVAAALPLPQTYLQSITVRVEEWRLKQRDTEEWMRHRQLPCELRERVRRFIQYKWLATRGVNEESILQALPADLRRDIKRHLCLGLVRRVPFFAQMDDQLLDAICERLVSSLCTKGTYIVREGDPVTEMLFIIRGKLESSTTNGGRTGFFNSITLKPGDFCGEELLGWALVPRPTTNLPSSTRTVKALIEVEAFALQAEDLKFVASQFRRLHSKKLQHTFRYYSHHWRTWASCFIQAAWRRYKRRKMAKDLSMRESFNSVRLDEVDNEDDDSPPKNNLALKFIARTRKVPQNMKGLPKLTKPDEPDFSAEPED*

>SsCNGC7-3D|Sspon.04G0002010-3D

MDCDLFAAWWSSSTRLVSRIFRGSAADAPGPSPMRPAIPLHQKQAGLAASKLGVGTSKKHRAFVASDEQWYNKIFDPSSDFILTWNRIFLFSCFVALFIDPLYFYVPKISYGSPKFCVGTDTRFAVGVTFFRSIADLLYVLHIIIKFRTAYINPSSTLRVFGRGDLVTNPKEIACKYIRSDLVVDVAAALPLPQIIVWFVIPAIKYSSAEHNNNILVLIVLAQYLPRLYLIFPLTYEIVKATGVVAKTAWEGAAYNMVLYLIASHVLGALWYLLSVDRQTFCWKTSCLSETDCHIKYLDCDTTLNATWASTTAVFSKCNASDDTISFDFGMFGPALSNQAPAQSFAMKYFYSLWWGLQNLSCYGQTLSVSTYLGETLYCIFLAVLGLVLFAHLIGNVQTYLQSITVRVEEWRLKQRDTEEWMRHRQLPCELRERVRRFIQYKWLATRGVNEESILQALPADLRRDIKRHLCLGLVRRVPFFAQMDDQLLDAICERLVSSLCTKGTYIVREGDPVTEMLFIIRGKLESSTTNGGRTGFFNSITLKPGDFCGEELLGWALVPRPTTNLPSSTRTVKALIEVEAFALQAEDLKFVASQFRRLHSKKLQHTFRYYSHHWRTWASCFIQAAWRWYKRRKMAKDLSMRESFNSVRLDEVDNEDDDSPPKNNLALKFIARTRKVPQNMKGLPKLTKPNEPDFSAEPED*

>SsCNGC7-1T|Sspon.04G0002010-1T

MDCDLFAAWWSSSTRLVSRIFRGSAADAPGPSPMRPAIPLHQKQAGLAASKLGVGTSKKHRAFVASDEQWYNKIFDPSSDFILTWNRIFLFSCFVALFIDPLYFYVPKISYGSPKFCVGTDTRFAV

>SsCNGC8|Sspon.08G0012670-1A

MRFGSGRVEDEMALTRQRTVRFHDERAKATIPIHHKQHGLAASRLGLGSSGKNKVFVAGDDLWYNKIIDPSSDFILTWIYVFHVSCFIALLMDPLYFYVPEIDYRQTTHCVRKDRRLAIIVTVFRSIADLFYVIQMIIKFRTAYLNPSSNLGVFGRGDLITDPKEIAKQYLRSDFAVDLVASLPLPQIIVWSVIPAIKYSSSEHGNDMLLLVALFQYILRLYLIFSLNDKIVKITGAFAKTAWQGAAYNLLLYMIASHVLGALWYFLSVDRQIACWKSFCNENDCHSRYLYCDVKPDSSWNGTLVFSSCDAKNTNKFDFGMFQPLLSNKTPNESFLKKYIYCLWWGLQNLSCYGQTLNVSTFIGETLYAILLAVVGLVLFAHLIGKVQTYLQSITARVEEWRLKQRDTEEWMRHRQLPHELRERVRRFVHYKWLATRGVDEESILNALPTDLCRDIKRHLCLDLVRRVPLFSQMDDQLLDAICERLVSSLSTEGTYIVREGDPVTEMLFIIRGKLESSTTDGGRTGFFNSITLKPGDFCGEELLGWALVPKPTVNLPLSTRTVKAIVEVEAFALQADDLRFVASQFRRLHSRKLQHTFRYYSHHWRTWAACFIQHAWRRQKRRKMAKDLSMRESFSSMRSYEGDNSPEQNLTLRRGASIIRELPKFRKPSEPDFSAEHDD*

>SsCNGC8-2B|Sspon.08G0012670-2B

MRFGSGRVEDEMALTRQRTVRFHDERAKATIPIHHKQHGLAASRLGLGSSGKNKVFVAGDDLWYNKIIDPSSDFILTWICVFRVSCFIALLMDPLYFYVPEIDYRQTTHCVRKDIRLAIIVTVFRSIVDLFYVIQMIIKFRTAYLNPSSNLGVFGRGDLITDPKEIAKQYLRSDFAVDLVASLPLPQIIVWSVIPAIKYSSSEHGNDMLLLVAFFQYILRLYLIFSLNDKIVKITGAFAKTAWQGAAYNLLLYMIASHVLGALWYLLSVDRQIACWKSFCNETETDCHTQYLYCDVKPDSSWNGTLVFSSCDAKNTNKFDFGMFQPLLSNKTPNESFLKKYIYCLWWGLQNLSTFIGETLYAILLAVVGLVLFAHLIGKVQTYLQSITARVEEWRLKQRDTEEWMRHRQLPHELRERVRRFVHYKWLATRGVDEESILNALPTDLRRDIKRHLCLDLVRRVPLFSQMDDQLLDAICERLVSSLSTEGTYIVREGDPVTEMLFIIRGKLESSTTDGGRTGFFNSITLKPGDFCGEELLGWALVPKPTVNLPLSTRTVKAIVEVEAFALQADDLRFVASQFRRLHSRKLQHTFRYYSHHWRTWAACFIQHAWRRQKRRKMAKDLSMRESFSSMRSYEGDNSPEQNLALRRGASIIRELPKFKKPSEPDFSAEHDD*

>SsCNGC8-3C|Sspon.08G0012670-3C

MRFGSGRVEDEMALTRQRTVRFHDERAKATIPIHHKQHGLAASRLGLGSSGKNKVFVAGDDLWYNKIIDPSSDFILTWICVFRVSCFIALLMDPLYFYVPEIDYRQTTHCVRKDIRLAIIVTVFRSIVDLFYVIQMIIKFRTAYLNPSSNLGVFGRGDLITDPKEIAKQYLRSDFAVDLVASLPLPQIIVWSVIPAIKYSSSEHGNDMLLLTYLQSITARVEEWRLKQTDTEEWMRHRQLPHELRERVRRFVHYKWLATRGVDEESILNALPTDLRRDIKRHLCLDLVRRVPLFSQMDDQLLDAICERLVSSLSTEGTYIVREGDPVTEMLFIIRGKLESSTTDGGRTGFFNSITLKPGDFCGEELLGWALVPKLTVNLPLSTRTVKTIVEVEAFALQADDLRFVASQFRRLHSRKLQHTFRYYSHHWRTWAACFIQHAWRRQKRRKMAKDLSMRESFSSMRSYEGDNSPEQNLTLRRGASIIRELPKFRKPSEPDFSAEHDD*

>SsCNGC8-4D|Sspon.08G0012670-4D

MRFGSGRVEDEMALTRQRTVRFHDERAKATIPIHHKQHGLAASRLGLGSSGKNKVFVAGDDLWYNKIIDPSSDFILTWIYVFHVSCFIALLMDPLYFYVPEIDYRQTTHCVRKDRRLAIIVTVFRSIADLFYVIQMIIKFRTAYLNPSSNLGVFGRGDLITDPKEIAKQYLRSDFAVDLVASLPLPQIIVWSVIPAIKYSSSEHGNDMLLLVALFQYILRLYLIFSLNDKIVKITGAFAKTAWQGAAYNLLLYMIASHVLGALWYFLSVDRQIACWKSFCNENDCHTRYLYCDVKPDSSWNGTLVFSSCDAKNTNRFDFGMFQPLLSNKTPNESFLKKYIYCLWWGLQNLSCYGQTLNVSTFIGETLYAILLAVVGLVLFAHLIGKVQTYLQSITARVEEWRLKQRDTEEWMRHRQLPHELRERVRRFVHYKWLATRGVDEESILNALPTDLCRDIKRHLCLDLVRRVPLFSQMDDQLLDAICERLVSSLSTEGTYIVREGDPVTEMLFIIRGKLESSTTDGGRTGFFNSITLKPGDFCGEELLGWALVPKPTVNLPLSTRTVKAIVEVEAFALQADDLRFVASQFRRLHSRKLQHTFRYYSHHWRTWAACFIQHAWRRQKRRKMAKDLSMRESFSSMRSYEGDNSPEQNLTLRRGASIIRELPKFRKPSEPDFSAEHDD*

>SsCNGC9|Sspon.02G0036020-1B

MFGSRVQDEVEMQRRPNNRIFPDERQNQPKPLYQTARADRFGANRIDVKNPEKLKVLNEGNKPWHQRILDPGSNIVLRWNRVYLVACLFALFIDPFFYYLPLIRQNDNGYSCVAKDQGLSIRITVLRSLADLFYMLNIAIKFHTAYVDPKSRVLGKGELVVDIKKIQQRYIRTDFFVDILAAVPLPQVTVWLIMPAIKSSDYNIRNTTFALIIVIQYVIRMYLIIPLSNQIIKAVGVVAKSAWGGAAYNLLLYMLASHITGAIYYLLSIERQITCWDQQCVAESNCNLRFISCENSGSDDYSEWAKNTGIFNNCDATTPNNISFNYGMFSSALSKGAVSSPFLEKYFYCLWWGLLQLSSSGNPLVTSAFITENLFAIAIGAISLILFAQLIGKMQTYLQSISKRLEEWRLRQRDMDEWMRHHQLPSHLQERVRRFVQVKWLATRGVEEESILQALPADIRRDVQRHLCLDLVRRVPFFSEMDDQLLDAICERLVSFLCPENTYISREGDPVNEMLFIIRGKLESSTTNGGRSNFFNSIILRPGDFAGEELLTWALLPKTNVHFPLSTRTVRSLTEVEAFALRAEDLKFVANQFRRLHSKKLQHTFRFYSHHWRTWAACFIQAAWRQHQRRKLAESLSRWESYSWWSEDHPTGDKPRQEGTSSGGTRTIAEGAIAQMHKLASASRRFRTEDIAIRRLQKPDEPDFSADHFD*

>SsCNGC9-2D|Sspon.02G0036020-2D

MFGSRVQDEVEMQRRPNNRIFPDERQNQPKPLYQTARADRFGANRIDVKNPEKLKVLNEGNKPWHQRILDPGSNIVLRWNRVYLVACLFALFIDPFFYYLPLIRQNDNGYSCVAKDQGLSIRITVLRSLADLFYMLNIAIKFHTAYVDPKSRVLGKGELVVDIKKIQQRYIRTDFFVDILAAVPLPQVTVWLIMPAIKSSDYNIRNTTFALIIVIQYVIRMYLIIPLSNQIIKAVGVVAKSAWGGAAYNLLLYMLASHITGAIYYLLSIERQITCWDQQCVAESNCNLRFISCENSGSDDYSEWAKKTGIFNNCDATTPNNISFNYGMFSSALSKGAVSSPFLEKYFYCLWWGLLQLSSSGNPLVTSAFITENLFAIAIGAISLILFAQLIGKMQTYLQSISKRLEEWRLRQRDMDEWMRHHQLPSHLQERVRRFVQVKWLATRGVEEESILQALPADIRRDVQRHLCLDLVRRVPFFSEMDDQLLDAICERLVSFLCPENTYISREGDPVNEMLFIIRGKLESSTTNGGRSNFFNSIILRPGDFAGEELLTWALLPKTNVHFPLSTRTVRSLTEVEAFALRAEDLKFVANQFRRLHSKKLQHTFRFYSHHWRTWAACFIQAAWRQHQRRKLAESLSRWESYSWWSEDHPPGDKPRQEGTSSGGTRTIAEGAIAHMHKLASASRRFRTEDITIRRLQKPDEPDFSADHFD*

>SsCNGC9-1T|Sspon.02G0036020-1T

MFGSRVQDEVEMQRRPNNRIFPDERQNQPKPLYQTARADRFGANRIDVKNPEKLKVLNEGNKPWHQRILDPGSNIVLRWNRVYLVACLFALFIDPFFYYLPLIRQNDNGSSCVAKDQGLSIRITVLRSLADLFYMLNIAIKFHTAYVDPKSRVLGKGELVVDIKKIQQRYIRTDFFVDILAAVPLPQVTVWLIMPAIKSSDYNIRNTTFALIIVIQYVIRMYLIIPLSNQIIKAVGVVAKSAWGGAAYNLLLYMLASHITGAIYYLLSIERQITCWDQQCVAESNCNLRFISCENSGSDDYSEWAKKTGIFNNCDATTPNNISFNYGMFSSALSKGAVSSPFLDKYFYCLWWGLLQLSSSGNPLVTSAFITENLFAIAIGAISLILFAQLIGKMQTYLQSISKRLEEWRLRQRDMDEWMRHHQLPSHLQERVRRFVQVKWLATRGVEEESILQALPADIRRDVQRHLCLDLVRRVPFFSEMDDQLLDAICERLVSFLCPENTYISREGDPVNEMLFIIRGKLESSTTNGGRSNFFNSIILRPGDFAGEELLTWALLPKTNVHFPLSTRTVRSLTEVEAFALRAEDLKFVANQFRRLHSKKLQHTFRFYSHHWRTWAACFIQAAWRQHQRRKLAESLSRWESYSWWSEDHPPGDKPRQEGTSSGGTRTIAEGAIAHMHKLASASRRFRTEDITIRRLQKPDEPDFSADHFD*

>SsCNGC10|Sspon.04G0024390-1B

MTFLVLMAEHNVVVSAFPRDQITEQIHVTEQRIQQDQSMFITKAMYASLRNFRFQNEIEVQSFRTSPLQQNLSSRKHGRAHDPRKCRLGFRGGCLEKACRNPTLKDRVLSRAFSEELESLMHAAGSSHLFFDPRGHLIHLWNKIFLSACLLSLFVDPLFLYLTGTQRNNHMCIEFKYSLALTLSMIRSLLDLFYAAHILFRFRTAFIAPSSRVFGRGELVIQPYKIARRYLGRTFWFDLVTALPLPQFVIWIVIPKLNESPTANRKSILRFSIIFQYLPRLFQIFPLTSQIVVATGVVAETAWACAAYNLILYMLASHIDADLIFFSFWTTEPDEYVQVLGALWYLFSVQRQEACWREACLLESPTCQTMFFDCKALSSNRTIWYELSNITSLCTPGNGFYAFGIYEEALHAKLTSSSFTQKYFYCFWWGLKNLSCLGQNLSTSLSIGEITFAIVIGVLGLVLFGLLIGNMQSYLQTTMVRLEEWRTKRTDMERWMHHRQIPQPLKQCVRRYHQYQWVATRGVDEEALLQDLPMDIRRDIKRHLCLDLVRRVPLFDEMDERMLEAICERLRPALYTRGTRLVRELDPVDSMLFIIRGYLDSFTTQGGRSGFFNSCRIGAGEFCGEELLTWALDPRPSAKLPLSTRTVRAVSEVEAFALVADDLRFVASQFRRLHSARIRHRFRFYSHQWRTWAACSIQAAWRRHKRRRASVELRVREGGDVRTAGSLRRSCRHSIDGEASIKKPMEPDFTVEEED*

>SsCNGC10-2C|Sspon.04G0024390-2C

MYASLRNFRFQNEIEVQSFRTSPLQQNLSSRKHGRAHDPRKCRLGFRGGCLEKACRNPTLKDRVLSRAFSEELESLMHAAGSSHLFFDPHGHLIHLWNKIFLSACLLSLFVDPLFLYLTGTQRNNHMCIEFKYSLALTLSMIRSLLDIFYAAHILFRFRTAFVAPSSRVFGQGELVIQPYKIARRYLGRTFWFDLVTALPLPQFVIWIVIPKLNESLTGTQRNNHMCIEFKYSLALTLSMIRSLLDIFYAAHILFRFRTAFVAPSSRVFGQGELVIQPYKIARRYLGRTFWFDLVTALPLPQFVIWIVIPKLNESLTANRKSILRFSIIFQYLPRLFQIFPLTSQIVMATGVMAETAWACAAYNLILYMLASHVLGALWYLFSVQRQEACWREACLLESPTCQTMFFDCKALSSNRTIWYELSNITSLCTPGNGFYPFGIYAEALQTKLTSSSFTQKYFYCFWWGLKNLSCLGQNLSTSLSIGEITFAIVIGVLGLVLFGLLIGNMQSYLQTTMVRLEEWRTKRTDMERWMHHRQIPQPLKQCVRRYHQYQWVATRGVDEEALLQDLPMDIRRDIKRHLCLDLVRRVPLFDEMDERMLEAICERLRPALYTRGTRLVRELDPVDSMLFIIRGYLDSFTTQGGRSGFFNSCRIGAGEFCGEELLTWALDPRPSAKLPLSTRTVRAVSEVEAFALVADDLRFVASQFRRLHSARIRHRFRFYSHQWRTWAACSIQAAWRRHKRRRASVELRVREGGDVRTAGSLRRSCRHSIDGEASIKKPMEPDFTVEEED*

>SsCNGC10-3D|Sspon.04G0024390-3D

MASGASRNVRFQNEIEVQSFRTSPLQQNLSSRKHGRAHDPRKCRLGFRGGCLEKACRNPTLKDRVLSRAFSEELESLMHAAGSSHLFFDPRGHLIHLWNKIFLSACLLSLFVDPLFLYLTGTQRNNHMCIEFKYSLALTLSMIRSLLDLFYAAHILFRFRTAFIAPSSRVFGRGELVIQPYKIARRYLGRTFWFDLVTALPLPQFVIWIVIPKLNESPTANRKSILRFSIIFQCLPRLFQIFPLTSQIIMATGVMAETAWACAAYNLILYMLASHIDADLIFFSFWTTEPDEYVQVLGALWYLFSVQRQEACWREACLLESPTCQTMFFDCKALSSNRTIWYELSNITSLCTPGNGFYAFGIYEEALHAKLTSSSFTQKYFYCFWWGLKNLSCLGQNLSTSLSIGEITFAIVIGVLGLVLFGLLIGNMQSYLQTTMVRLEEWRTKRTDMERWMHHRQIPQPLKQCVRRYHQYQWVATRGVDEEALLQDLPMDIRRDIKRHLCLDLVRRVPLFDEMDERMLEAICERLRPALYTRGTRLVRELDPVDSMLFIIRGYLDSFTTQGGRSGFFNSCRIGAGEFCGEELLTWALDPRPSAKLPLSTRTVRAVSEVEAFALVADDLRFVASQFRRLHSARIRHRFRFYSHQWRTWAACSIQAAWRRHKRRRASVELRVREGGDVRTAGSLRRSCRHSIDGEASIKKPMEPDFTVEEED*

>SsCNGC10-1T|Sspon.04G0024390-1T

MASGASRNVRFQNEIEVQSFRTSPLQQNLSSRKHGRAHDPRKCRLGFRGGCLEKACRNPTLKDRVLSRAFSEELESLMHAAGSSHLFFDPRGHLIHLWNKIFLSACLLSLFVDPLFLYLTGTQRNNHMCIEFKYSLALTLSMIRSLLDLFYAAHILFRFRTAFIAPSSRVFGRGELVIQPYKIARRYLGRTFWFDLVTALPLPQFVIWIVIPKLNESPTANRKSILRFSIIFQCLPRLFQIFPLTSQIIMATGVMAETAWACAAYNLILYMLASHIDADLIFFSFWTTEPDEYVQVLGALWYLFSVQRQEACWREACLLESPTCQTMFFDCKALSSNRTIWYELSNITSLCTPGNGFYPFGIYEEALKTKLTSSSFTQKYFYCFWWGLKNLSCLGQNLSTSLSIGEITFAIVIGVLGLVLFGLLIGNMQSYLQTTMVRLEEWRTKRTDMERWMHHRQIPQPLKQCVRRYHQYQWVATRGVDEEALLQDLPMDIRRDIKRHLCLDLVRRVPLFDEMDERMLEAICERLRPALYTRGTRLVRELDPVDSMLFIIRGYLDSFTTQGGRSGFFNSCRIGAGEFCGEELLTWALDPRPSAKLPLSTRTVRAVSEVEAFALVADDLRFVASQFRRLHSARIRHRFRFYSHQWRTWAACSIQAAWRRHKRRRASVELRVREGGDVRTAGSLRRSCRHSIDGEASIKKPMEPDFT

>SsCNGC11|Sspon.04G0008000-1A

MYASLRNFRFQNEIEVQSFRTSPLQQNLSSRKHGRAHDPRKCRLGFRGGCLEKACRNPTLKDRVLSRAFSEELESLMHAAGSSHLFFDPRGHLIHLWSKIFLSACLLSLFVDPLFLYLTGTQRNNNVCIEFKYSLALTLSMIRSLLDLFYAAHILFRFRTAFIAPSSRVFGRGELVIQPYKIARRYLGRTFWFDLVTALPLPQFVIWIVILKLNESPTTNRKSILRFSIIFQYLPRLFQIFPLTSQIIMATGVMAETAWACAAYNLILYMLASHVLGALWYLFSVQRQEACWREACLLESPTCQTMFFDCKALSSNRTIWYELSNITSLCTPGNGFYAFGIYEEALHAKLTSSSFTQKYFYCFWWGLKNLSCLGQNLSTSLSIGEITFAIVIGVLGLVLFGLLIGNMQSYLQTTMVRLEEWRTKRTDMERWMHHRQIPQPLKQCVRRYHQYQWVATRGVDEEALLQDLPMDIRRDIKRHLCLDLVRRVPLFDEMDERMLEAICERLRPALYTRGTRLVRELDPVDSMLFIIRGYLDSFTTQGGRSGFFNSCRIGAGEFCGEELLTWALDPRPSAKLPLSTRTVRAVSEVEAFALVADDLRFVASQFRRLHSARIRHRFRFYSHQWRTWAACSIQAAWRRHKRRRASVELRVREGGDVRTAGSFRRSCRHSIDGEASIKKPMEPDFTVEEED*

>SsCNGC11-2B|Sspon.04G0008000-2B

MTFLVLMAEHNVVVSAFPRDQITEQIHVTEQRIQQDQSMFITKAMYASLRNFRFQNEIEVQSFRTSPLQQNLSSRKHGRAHDPRKCRLGFRGGCLEKACRNPTLKDRVLSRAFSEELESLMHAAGSSHLFFDPRGHLIHLWNKIFLSACLLSLFVDPLFLYLTGTQRNNHMCIEFQYSLALTLSMIRSLLDIFYAAHILFRFRTAFIAPSSRVFGRGELVIQPYKIARRYLGRTFWFDLVTALPLPQFVIWIVIPKLNESPTANRKSILRFSIIFQYLPRLFQIFPLTSQIVVATGVVAETAWACAAYNLILYMLASHVLGALWYLFSVQRQEACWREACLLESPTCQTMFFDCKALSSNRTIWYELSNITSLCTPGNGFYAFGIYEEALHAKLTSSSFTQKYFYCFWWGLKNLSCLGQNLSTSLSIGEITFAIVIGVLGLVLFGLLIGNMQSYLQTTMVRLEEWRTKRTDMERWMHHRQIPQPLKQCVRRYHQYQWVATRGVDEEALLQDLPMDIRRDIKRHLCLDLVRRVPLFDEMDERMLEAICERLRPALYTRGTRLVRELDPVDSMLFIIRGYLDSFTTQGGRSGFFNSCRIGAGEFCGEELLTWALDPRPSAKLPLSTRTVRAVSEVEAFALVADDLRFVASQFRRLHSARIRHRFRFYSHQWRTWAACSIQAAWRRHKRRRASVELRVREGGDVRTAGSLRRSCRHSIDGEASIKKPMEPDFTVEEED*

>SsCNGC12|Sspon.04G0031130-1C

MTDQERDDVPMLLRNVELPRFPLRSTSMCIPVRDDEYEEDTFVPHTGPLFVQPPTQTAPGIPFTSRDTPDRLPRPSQGKQVSKPHAIMPEEIRGNRWSYSGQVPKNEHLMMSGPLGQCDNPDCVNCPPACRNKRHFQRGSNALDNKIHNILYGHSGGWKKKIEQIMAYIPIMNPHAKPVQQWNQFFVISCLIAIFIDPLFFFLLSVRQDGNCIVLNWNFATGLAVVRSVTDAIYFLHMLLQFRLAYVAPESRVVGAGDLVDEPKKVAIHYLCGYFFLDFFVVLPLPQVMILLVVPKVGLSAANYAKNLLRATVLLQYVPRIIRFVPLLDGQSANGFIFESAWANFVINLLMFVLAGHVVGSCWYLFGLQRVNQCLRDACSASTIPYCDSFIDCGRGMGSGLYRQQWFNDLGAEACFNTGNNATFQYGIYEQAVLLTTEDSAVKRYIYSLFWGFQQISTLAGNLVPSYFVWEVLFTMAIIGLGLRLEMQLRRRDVEKWMSHRRLPEDLRRRVRRAERFTWAATQGVNEEELLSNLPEDIQRDIRRHFFRFLNKVRLFTLMDWPILDAICDKLRQNLYISGSDILYQGGTVEKMVFIVRGKLESISADGSKAPLHDGDVCGEELLTWYLEHSSANRDGGKIKFQGMRLVAIRTVRCLTNVEAFVLRASDLEEVTSQFARFLRNPRVQGAIRYESPYWRTIAATRIQVAWRYRKRRLKRAEKSRLSEETYTSHGISTHDSFQRGQKG*

>SsCNGC12-2D|Sspon.04G0031130-2D

MTDQERDDVPMLLRNVELPRFPLRSTSMCIPVRDDEYEEDTFVPHTGPLFVQPPTQTAPGIPFTSRDTPDRLPRPSQGKQVSKPHAIMPEEIRGNRWSYSGQVPKNEHLMMSGPLGQCDNPDCVNCPPACRNKRHFQRGSNALDNKIHNILYGHSGGWKKKIEQIMAYIPIMNPHAKPVQQWNQFFVISCLIAIFIDPLFFFLLSVRQDGNCIVLNWNFATGLAVVRSVTDAIYFLHMLLQFRLAYVAPESRVVGAGDLVDEPKKVAIHYLCGYFFLDFFVVLPLPQVMILLVVPKVGLSAANYAKNLLRATVLLQYVPRIIRFVPLLDGQSANGFIFESAWANFVINLLMFVLAGHVVGSCWYLFGLQRVNQCLRDACSASTIPYCDSFIDCGRGMGSGLYRQQWFNDLGAEACFNTGNNATFQYGIYEQAVLLTTEDSAVKRYIYSLFWGFQQISTLAGNLVPSYFVWEVLFTMAIIGLGLRLEMQLRRRDVEKWMSHRRLPEDLRRRVRRAERFTWAATQGVNEEELLSNLPEDIQRDIRRHFFRFLNKVRLFTLMDWPILDAICDKLRQNLYISGSDILYQGGTVEKMVFIVRGKLESISADGSKAPLHDGDVCGEELLTWYLEHSSANRDGGKIKFQGMRLVAIRTVRCLTNVEAFVLRASDLEEVTSQFARFLRNPRVQGAIRYESPYWRTIAATRIQVAWRYRKRRLKRAEKSRLSEETYTSHGISTHDSFQRGQKG*

>SsCNGC13|Sspon.01G0049590-1B

MPPLAFLRRSLPARFMLASTTSSSLGGIEFLESAPMQRLLARACDGASGVWGSPGVARDEEAGGGGGGLSGRSAGGPSGECYACTQPGVPAFHSTTCDQVHSPDWDADAGSSLVPVQAQQSQPAASASAAAQHAGAAARWLFGPVLDPRSKRVQRWNRWILLGRAAALAVDPLFFYALSIGRAGQPCLYMDAGLAAAVTALRTCADVAHLAHVLLQFRLAYVSRESLVVGCGKLVWDARAIAAHYARSVKGLCFDLFVILPIPQVIFWLVIPKLIREEQVKLRIPRGQVVAAASIVSPKSGRDNLSTTFGNDLAPTSNGIEVIFSIINVLSGLMLFTLLIGNIQVFLHAVLARKRKMQLRFRDMEWWMRRRQLPSRLRQRVRKYERERWAAVTGDEEMEMIKDLPEGLRRDIKRYLCLELVKQVPLFHGMDDLILDNICDRLRPLVFSSGEKVIREGDPVQRMVFILQGKLQSTQPLTKGVVATCMLGAGNFLGDELLSWCLRRPFVDRLPASSATFECVEAAQAFCLDAPDLRFITEHFRYKFANEKLKRTARYYSSNWRTWAAVNIQLAWRRYRARTSTTDLAAMAAVPLVGGPDDGDRRLRHYAAMFMSLRPHDHLDGELGVRRGGVRASSPFPRLDLFRRGTAPLLLLPGSLEAVFFLLPLWAVVLVLVWDLGVLLSDLMDQKTKLEMMGTQAATLVVVVVSFVVSRLRRFVVAANLVQGVVAAAGVAAVVQAAVATPVDAPVVDDVVGDAADGEGEAGGARPPMMWNNNTFGFVLKRMAQILFDGSRTDKLFKHHPKDAEFLNTPIRFYTEMQTIFGSTLATGGGAWLHCSTPAFEGKTTIELGEGSKATLPVTSIAGGKRKRVAFSEDEMLMMTNMTDAVNNVANAMMKAAATHVDPALYLAIMEMPDFSIEALIVAYTHLLENKAVH

>SsCNGC13-1P|Sspon.01G0049590-1P

MPPLAFLRRSLPARFMLASTTSSSLGGIEFLEFAPMVSSLDRSQRLLARACDGGVWGSPGVARDEEAGGSGGGLSGRSAGGPSGECYACTQPGVPAFHSTTCDQVHSPDWDADAGSSLVPVQAQQSQPAASASAAAQHAGAAARWLFGPVLDPRSKRVQRWNRWILLGRAAALAVDPLFFYALSIGRAGQPCLYMDAGLAAAVTALRTCADVAHLAHVLLQFRLAYVSRESLVVGCGKLVWDARAIAAHYARSVKGLCFDLFVILPIPQVIFWLVIPKLIREEQVKLIMTILLLMFIFQFLPKVYHIIHIMRKMQKVTGYIFGSIWWGFGLNLFAYFIASHIAGGCWYVLAIQRIASCLQEECKKNNSCDLISLACSKEICFHPPWSSNVNGFACDTNMTSFSQRNVSTCLSGKGTFAYGIYLGALPVISSNSLAVKILYPIFWGLMTLSTFGNDLAPTSNGIEVIFSIINVLSGLMLFTLLIGNIQVFLHAVLARKRKMQLRFRDMEWWMRRRQLPSRLRQRVRKYERERWAAVTGDEEMEMIKDLPEGLRRDIKRYLCLELVKQVPLFHGMDDLILDNICDRLRPLVFSSGEKVIREGDPVQRMVFILQGKLQSTQPLTKGVVATCMLGAGNFLGDELLSWCLRRPFVDRLPASSATFECVEAAQAFCLDAPDLRFITEHFRYKFANEKLKRTARYYSSNWRTWAAVNIQLAWRRYRARTSTTDLAAMAAGHWLAGPTTGTGGSDTTRPCSCRSGRMTT*

>SsCNGC14|Sspon.03G0005320-1A

MTHACGVTIFVRSQVVVWVASPAMIRAGSTTAVMTVLLVAFLLEYLPKIYHSVRVLRRMQDVSGYLFGTIWWGIALNLMAYFVAAHAVGACWYLLGAQRATKCLREQCAQAGSGCAPWALACAEPLYYGRSVNVGADRLAWAGNATARGTCLDSADNYQYGAYQWTVMLVANPSRVERILLPIFWGLMTLSTFGNLESTTEWLEIVFNIITITGGLILVTMLIGNIKVFLNATTSKKQAMHTRLRGVELWMKRKNLPRSYRHRVRQYERQRWAATRGVDECRIVRDLPEGLRRDIKYHLCLGLVRQVPLFQHMDDLVLENICDRVKSLIFPKGEVIVREGDPVKRMMFIVRGHLQSSQVLRNGAESCCMLGPGNFSGDELLSWCLRRPFLERLPASSSTLTTLESTEAFGLDAADVKYVTQHFRYTFTNDKVRRSARYYSPGWRTWAAVAVQLAWRRYKHRKTLASLSFIRPRRPLSRCSSLGEEKLRLYTALLTSPKPNQDDL

>SsCNGC15|Sspon.03G0030490-1B

MLVANPSRVERILLPIFWGLMTLSTFGNLESTTEWLEIVFNIVTITGGLILVTMLIGNIKVFLNATTSKKQAMHTRLRGVELWMKRKNLPRSYRHRVRQYERQRWAATRGVDECRIVRDLPEASAETSSTTSASASCAREGDPVKRMMFIVRGHLQSSQVLRNGAESCCMLGPGNFSGDELLSWCLRRPFLERLPASSSTLTTLESTEAFGLDAADVKYVTQHFRYTFTNDKVRRSARYYSPGWRTWAAVAVQLAWRRYKHRKTLASLSFIRPRRPLSRCSSLGEEKLRLYTALLTSPKPNQDDL

>SsCNGC16|Sspon.07G0004720-1A

MIRAGLTTPVMTVLLVSFLLEYLPKIYHAARLLRRMQGQSGYIFGTIWWGIALNLMAYFVAAHAVGACWYLLGVQRASKCLKEQCLQAAAGGCARSSAVACAAPLYYGGAPSSTGTVGSGDRLAWARNAQARGTCLSSGGDNYQYGAYSWTVMLVANPSRVERMLLPIFWGLMTLSTFGNLESTTEWVEIVFNIVTITGGLVLVTMLIGNIKVFLNATTSKKQAMHTRLRSVEWWMKRKNLPRSFRARVRQFERQRWAATRGVDECQIVRDLPEGLRRDIKYHLCLDLVRQVPFFQHMDDLVLENICDRVKSLIFPKGETIVREGDVVQRMLFIVRGHLQCSQVLRNGATSSCTLGPGNFSGDELLSWCLRRPFLERLPTSSATLVTLESTEVFGLDAADVKYVTQHFRYTFTSDKVRRSARYYSPGWRTWAAVAIQLAWRRYKHRKTLSSLSFIRPRRPLSRCSSLGEEKLRLYTAILTSPKPNQDDDF*

>SsCNGC16-2B|Sspon.07G0004720-2B

MSSGLSARSSPSSSTASPSDDPRRKEQGRHATSGSGRRSRWRRRVQWLGGAAWALDPRARWVRDWNRAYLLACAAGLMVDPLFLYAVSLSGPLMCLFVDGWLAAAVTALRCAVDAMHVWNVATQIRIARGAGAAAQGSKPVAGGAGDEEQQQQQGAEEDDDEEAARKLPEDATPRKGMLLDFFVILPVMQVVVWVAAPAMIRAGLTTPVMTVLLVSFLLEYLPKIYHAARLLRRMQGQSGYIFGTIWWGIALNLMAYFVAAHAVGACWYLLGVQRASKCLKEQCLQAAAGGCARSSAVACAAPLYYGGAPSSTGTVGSGDRLAWARNAQARGTCLSSGGDNYQYGAYSWTVMLVANPSRVERMLLPIFWGLMTLSTFGNLESTTEWVEIVFNIVTITGGLVLVTMLIGNIKVFLNATTSKKQAMHTRLRSVEWWMKRKNLPRSFRARVRQFERQRWAATRGVDECQIVRDLPEGLRRDIKYHLCLDLVRQVPFFQHMDDLVLENICDRVKSLIFPKGETIVREGDVVQRMLFIVRGHLQCSQVLRNGATSSCTLGPGNFSGDELLSWCLRRPFLERLPTSSATLVTLESTEVFGLDAADVKYVTQHFRYTFTNDKVRRSARYYSPGWRTWAAVAIQLAWRRYKHRKTLSSLSFIRPRRPLSRCSSLGEEKLRLYTAILTSPKPNQDDDF*

>SsCNGC16-3C|Sspon.07G0004720-3C

MSSGLSARSSPSSSTASPSDDPRRKEQGRHATSGSGRRSRWRRRVQWLGGAAWALDPRARWVRDWNRAYLLACAAGLMVDPLFLYAVSLSGPLMCLFVDGWLAAAVTALRCAVDAMHVWNVATQLRIARGAGAAAQGSKPVAGGAGDEEQQQQGAEEDDDEEAARKLPEDATPRKGMLLDFFVILPVMQVVVWVAAPAMIRAGLTTPVMTVLLVSFLLEYLPKIYHAARLLRRMQGQSGYIFGTIWWGIALNLMAYFVAAHAVGACWYLLGVQRASKCLKEQCLQAAAGGCARSSAVACAAPLYYGGSPSSAGTVGSGDRLAWARNAQARGTCLSSGGDNYQYGAYSWTVMLVANPSRVERMLLPIFWGLMTLSTFGNLESTTEWVEIVFNIVTITGGLVLVTMLIGNIKVFLNATTSKKQAMHSRLRSVEWWMKRKNLPRSFRARVRQFERQRWAATRGVDECQIVRDLPEGLRRDIKYHLCLDLVRQVPFFQHMDDLVLENICDRVKSLIFPKGETIVREGDVVQRMLFIVRGHLQCSQVLRNGATSSCTLGPGNFSGDELLSWCLRRPFLERLPTSSATLVTLESTEVFGLDAADVKYVTQHFRYTFTNDKVRRSARYYSPGWRTWAAVAIQLAWRRYKHRKTLSSLSFIRPRRPLSRCSSLGEEKLRLYTAILTSPKPNQDDDF*

>SsCNGC16-4D|Sspon.07G0004720-4D

MSSGLSARSSPSSSTASPSDDPRRKEQGRHATSGSGRRSRWRRRVQWLGGAAWALDPRARWVRDWNRAYLLACAAGLMVDPLFLYAVSLSGPLMCLFVDGWLAAAVTALRCAVDAMHVWNVATQIRIARGAGAAAQGSKPVAGGAGDEEEQQQGAEEDDDEEAARKLPDDATPRKGMLLDFFVILPVMQNGAHRGEEEARRAYSPTPSLPQPRRRRAAKKSKPPRIDNPQQTLAPRTRNDGEQNLGLVVVWVAAPAMIRAGLTTPVMTVLLVSFLLEYLPKIYHAARLLRRMQGQSGYIFGTIWWGIALNLMAYFVAAHAVGACWYLLGVQRASKCLKEQCLQAAGGGCARSSAVACAAPLYYGGSPSSTGTVGRGDRLAWARNAQARGTCLSSGGDNYQYGAYSWTVMLVANPSRVERMLLPIFWGLMTLSTFGNLESTTEWVEIVFNIVTITGGLVLVTMLIGNIKVFLNATTSKKQAMHTRLRSVEWWMKRKNLPRSFRARVRQFERQRWAATRGVDECQIVRDLPEGLRRDIKYHLCLDLVRQVPFFQHMDDLVLENICDRVKSLIFPKGETIVREGDVVQRMLFIVRGHLQCSQVLRNGATSSCTLGPGNFSGDELLSWCLRRPFLERLPTSSATLVTLESTEVFGLDAADVKYVTQHFRYTFTNDKVRRSARYYSPGWRTWAAVAIQLAWRRYKHRKTLSSLSFIRPRRPLSRCSSLGEEKLRLYTAILTSPKPNQDDDF
