# Supplementary material for: Genome-wide identification and expression analysis of the cyclic nucleotide-gated ion channel (CNGC) gene family in Saccharum spontaneum
Source: BMC Genomics. 2023 May 25;24:281. doi: 10.1186/s12864-023-09307-3 (PMC10214738; doi:10.1186/s12864-023-09307-3)
Supplement: Supplementary file 4 — Additional file 4: Supplementary file 3. Amino acid sequences of CNGCs from Arabidopsis thaliana, Oryza sativa and Zea mays. [file 12864_2023_9307_MOESM4_ESM.docx]

Supplementary file 3: Amino acid sequences of CNGCs from *Arabidopsis thaliana*, *Oryza sativa* and *Zea mays*.

>AtCNGC1

MNFRQEKFVRFQDWKSDKTSSDVEYSGKNEIQTGIFQRTISSISDKFYRSFESSSARIKLFKRSYKSYSFKEAVSKGIGSTHKILDPQGPFLQRWNKIFVLACIIAVSLDPLFFYVPIIDDAKKCLGIDKKMEITASVLRSFTDVFYVLHIIFQFRTGFIAPSSRVFGRGVLVEDKREIAKRYLSSHFIIDILAVLPLPQMVILIIIPHMRGSSSLNTKNMLKFIVFFQYIPRFIRIYPLYKEVTRTSGILTETAWAGAAFNLFLYMLASHVFGAFWYLFSIERETVCWKQACERNNPPCISKLLYCDPETAGGNAFLNESCPIQTPNTTLFDFGIFLDALQSGVVESQDFPQKFFYCFWWGLQNLSSLGQNLKTSTYIWEICFAVFISIAGLVLFSFLIGNMQTYLQSTTTRLEEMRVKRRDAEQWMSHRLLPENLRKRIRRYEQYKWQETRGVDEENLLSNLPKDLRRDIKRHLCLALLMRVPMFEKMDEQLLDALCDRLQPVLYTEESYIVREGDPVDEMLFIMRGKLLTITTNGGRTGFLNSEYLGAGDFCGEELLTWALDPHSSSNLPISTRTVRALMEVEAFALKADDLKFVASQFRRLHSKQLRHTFRYYSQQWKTWAACFIQAAWRRYIKKKLEESLKEEENRLQDALAKEACGSSPSLGATIYASRFAANILRTIRRSGSVRKPRMPERMPPMLLQKPAEPDFNSDD

>AtCNGC2

MPSHPNFIFRWIGLFSDKFRRQTTGIDENSNLQINGGDSSSSGSDETPVLSSVECYACTQVGVPAFHSTSCDQAHAPEWRASAGSSLVPIQEGSVPNPARTRFRRLKGPFGEVLDPRSKRVQRWNRALLLARGMALAVDPLFFYALSIGRTTGPACLYMDGAFAAVVTVLRTCLDAVHLWHVWLQFRLAYVSRESLVVGCGKLVWDPRAIASHYARSLTGFWFDVIVILPVPQAVFWLVVPKLIREEKVKLIMTILLLIFLFQFLPKIYHCICLMRRMQKVTGYIFGTIWWGFALNLIAYFIASHVAGGCWYVLAIQRVASCIRQQCMRTGNCNLSLACKEEVCYQFVSPTSTVGYPCLSGNLTSVVNKPMCLDSNGPFRYGIYRWALPVISSNSLAVKILYPIFWGLMTLSTFANDLEPTSNWLEVIFSIVMVLSGLLLFTLLIGNIQVFLHAVMAKKRKMQIRCRDMEWWMKRRQLPSRLRQRVRRFERQRWNALGGEDELELIHDLPPGLRRDIKRYLCFDLINKVPLFRGMDDLILDNICDRAKPRVFSKDEKIIREGDPVQRMIFIMRGRVKRIQSLSKGVLATSTLEPGGYLGDELLSWCLRRPFLDRLPPSSATFVCLENIEAFSLGSEDLRYITDHFRYKFANERLKRTARYYSSNWRTWAAVNIQMAWRRRRKRTRGENIGGSMSPVSENSIEGNSERRLLQYAAMFMSIRPHDHLE

>AtCNGC3

MMNPQRNKFVRFNGNDDEFSTKTTRPSVSSVMKTVRRSFEKGSEKIRTFKRPLSVHSNKNKENNKKKKILRVMNPNDSYLQSWNKIFLLLSVVALAFDPLFFYIPYVKPERFCLNLDKKLQTIACVFRTFIDAFYVVHMLFQFHTGFITPSSSGFGRGELNEKHKDIALRYLGSYFLIDLLSILPIPQVVVLAIVPRMRRPASLVAKELLKWVIFCQYVPRIARIYPLFKEVTRTSGLVTETAWAGAALNLFLYMLASHVFGSFWYLISIERKDRCWREACAKIQNCTHAYLYCSPTGEDNRLFLNGSCPLIDPEEITNSTVFNFGIFADALQSGVVESRDFPKKFFYCFWWGLRNLSALGQNLKTSAFEGEIIFAIVICISGLVLFALLIGNMQKYLQSTTVRVEEMRVKRRDAEQWMSHRMLPDDLRKRIRKYEQYKWQETKGVEEEALLSSLPKDLRKDIKRHLCLKLLKKVPWFQAMDDRLLDALCARLKTVLYTEKSYIVREGEPVEDMLFIMRGNLISTTTYGGRTGFFNSVDLVAGDFCGDLLTWALDPLSSQFPISSRTVQALTEVEGFLLSADDLKFVATQYRRLHSKQLRHMFRFYSVQWQTWAACFIQAAWKRHCRRKLSKALREEEGKLHNTLQNDDSGGNKLNLGAAIYASRFASHALRNLRANAAARNSRFPHMLTLLPQKPADPEFPMDET

>AtCNGC4

MATEQEFTRASRFSRDSSSVGYYSEEDNTEEEDEEEEEMEEIEEEEEEEEEEDPRIGLTCGGRRNGSSNNNKWMMLGRILDPRSKWVREWNKVFLLVCATGLFVDPLFLYTLSVSDTCMCLLVDGWLALTVTALRSMTDLLHLWNIWIQFKIARRWPYPGGDSDGDTNKGGGTRGSTRVAPPYVKKNGFFFDLFVILPLPQVVLWVVIPSLLKRGSVTLVVSVLLVTFLFQYLPKIYHSIRHLRRNATLSGYIFGTVWWGIALNMIAYFVAAHAAGACWYLLGVQRSAKCLKEQCENTIGCDLRMLSCKEPVYYGTTVMVLDRARLAWAQNHQARSVCLDINTNYTYGAYQWTIQLVSSESRLEKILFPIFWGLMTLSTFGNLESTTEWSEVVFNIIVLTSGLLLVTMLIGNIKVFLHATTSKKQAMHLKMRNIEWWMKKRHLPIGFRQRVRNYERQRWAAMRGVDECEMVQNLPEGLRRDIKYHLCLDLVRQVPLFQHMDDLVLENICDRVKSLIFTKGETIQKEGDAVQRMLFVVRGHLQSSQLLRDGVKSCCMLGPGNFSGDELLSWCLRRPFVERLPPSSSTLVTLETTEAFGLDAEDVKYVTQHFRYTFVNEKVKRSARYYSPGWRTWAAVAVQLAWRRYKHRLTLTSLSFIRPRRPLSRCASLGEDKLRLYAAILTSPKPNPDDFDDY

>AtCNGC5

MAGKRENFVRVDDLDSRLPSSSVAFQQNYASNFSGQLHPIHASNETSRSFKKGIQKGSKGLKSIGRSLGFGVYRAVFPEDLKVSEKKIFDPQDKFLLYCNKLFVASCILSVFVDPFFFYLPVINAESKCLGIDRKLAITASTLRTFIDVFYLAHMALQLRTAYIAPSSRVFGRGELVIDPAQIAKRYLQRWFIIDFLSVLPLPQIVVWRFLQSSNGSDVLATKQALLFIVLVQYIPRFLRVLPLTSELKRTAGVFAETAWAGAAYYLLLYMLASHIVGAFWYLLALERNDACWQEACIDAGNCSTDFLYCGNQNMDGYAVWNRAKESVLKSKCRADLDDNNPPFDFGIYTQALSSGIVSSQNFIVKYCYCLWWGLQNLSTLGQGLETSTYPMEIIFSISLAISGLILFALLIGNMQTYLQSLTIRLEEMRVKRRDSEQWMHHRMLPQDLRERVRRYDQYKWLETRGVDEEYLVQNLPKDLRRDIKRHLCLALVRRVPLFKSMDDKLLDAICMRLKPCLFTESTYLVREGDPVDEMLFIIRGRLESVTTDGGRSGFFNRSLLKEGEFCGEELLTWALDPKSGVNLPSSTRTVKALTEVEAFALTSEELKFVASQFRRLHSRQVQHTFRFYSHQWRTWAACFIQAAWRRYCKRKKMEEAEAEAAAVSSSTAGPSYSIGAAFLATKFAANALRTIHRNRNTKIRDLVKLQKPPEPDFTAD

>AtCNGC6

MFDTCGPKGVKSQVISGQRENFVRLDSMDSRYSQSSETGLNKCTLNIQGGPKRFAQGSKASSGSFKKGFRKGSEGLWSIGRSIGLGVSRAVFPEDLEVSEKKIFDPQDKFLLLCNKLFVASCILAVSVDPLFLYLPFINDKAKCVGIDRKLAIIVTTIRTVIDSFYLFHMALRFRTAYVAPSSRVFGRGELVIDPAQIAKRYLQQYFIIDLLSVLPVPQIIVWRFLYTSRGANVLATKQALRYIVLVQYIPRFLRMYPLSSELKRTAGVFAETAWAGAAYYLLLYMLASHVSIQFSCTILYPFLCSHKFMCMQIVGALWYLLALERNNDCWSKACHNNQNCTRNFLFCGNQNMKGYAAWDNIKVSYLQLKCPVNVPEDEEPPFDFGIYLRALSSGIVSSKNFVSKYFFCLWWGLQNLSTLGQGLETSTYPGEVIFSITLAIAGLLLFALLIGNMQTYLQSLTIRLEEMRVKRRDSEQWMHHRMLPPELRERVRRYDQYKWLETRGVDEENLVQNLPKDLRRDIKRHLCLALVRRVPLFENMDERLLDAICERLKPCLFTEKSYLVREGDPVNEMLFIIRGRLESVTTDGGRSGFYNRSLLKEGDFCGDELLTWALDPKSGSNLPSSTRTVKALTEVEAFALIADELKFVASQFRRLHSRQVQHTFRFYSQQWRTWAACFMQAAWRRYIKRKKLEQLRKEEEEEEAAAASVIAGGSPYSIRATFLASKFAANALRSVHKNRTAKSTLLLSSTKELVKFQKPPEPDFSAEDH

>AtCNGC7

MYKSQYISGQREKFVRLDDIDSSSSPATGMMMQRNCFGFNLKNRGGEKKKASKSFREGVKKIRSEGLITIGKSVTRAVFPEDLRITEKKIFDPQDKTLLVWNRLFVISCILAVSVDPLFFYLPIVDNSGSSCIGIDTKLAVTTTTLRTIVDVFYLTRMALQFRTAYIAPSSRVFGRGELVIDPAKIAERYLTRYFVVDFLAVLPLPQIAVWKFLHGSKGSDVLPTKTALLNIVIVQYIPRFVRFIPLTSELKKTAGAFAEGAWAGAAYYLLWYMLASHITGAFWYMLSVERNDTCWRFACKVQPDPRLCVQILYCGTKFVSSGETEWIKTVPELLKSNCSAKADDSKFNYGIYGQAISSGIVSSTTFFSKFCYCLWWGLQNLSTLGQGLQTSTFPGEVLFSIAIAIAGLLLFALLIGNMQTYLQSLTVRLEEMRIKRRDSEQWMHHRSLPQNLRERVRRYDQYKWLETRGVDEENIVQSLPKDLRRDIKRHLCLNLVRRVPLFANMDERLLDAICERLKPSLFTESTYIVREGDPVNEMMFIIRGRLESVTTDGGRSGFFNRGLLKEGDFCGEELLTWALDPKAGSNLPSSTRTVKALTEVEAFALEAEELKFVASQFRRLHSRQVQQTFRFYSQQWRTWASCFIQAAWRRYSRRKNAELRRIEEKEEELGYEDEYDDESDKRPMVITRSESSSRLRSTIFASRFAANALKGHRLRSSESSKTLINLQKPPEPDFDAE

>AtCNGC8

MYKSQYISGHREKFVRLDDTDSRVSMSSNATGMKKRSCFGLFNVTSRGGGKTKNTSKSFREGVKIGSEGLKTIGKSFTSGVTRAVFPEDLRVSEKKIFDPQDKTLLLWNRMFVISCILAVSVDPLFFYLPIVDNSKNCIGIDSKLAVTTTTLRTIIDVFYLTRMALQFRTAYIAPSSRVFGRGELVIDPAKIAERYLTRYFIVDFLAVLPLPQIAVWKFLHGSKGTDVLPTKQALLHIVITQYIPRFVRFIPLTSELKKTAGAFAEGAWAGAAYYLLWYMLASHITGAFWYMLSVERNDTCLRSACKVQPDPKVCVQILYCGSKLMSSRETDWIKSVPDLFKNNCSAKSDESKFNYGIYSQAVSSGIVSSTTFFSKFCYCLWWGLQNLSTLGQGLQTSTYPGEVLFSIAIAVAGLLLFALLIGNMQTYLQSLTVRLEEMRIKRRDSEQWMHHRSLPQNLRERVRRYDQYKWLETRGVDEENIVQSLPKDLRRDIKRHLCLNLVRRVPLFANMDERLLDAICERLKPSLYTESTYIVREGDPVNEMLFIIRGRLESVTTDGGRSGFFNRGLLKEGDFCGEELLTWALDPKAGSNLPSSTRTVKALTEVEAFALEAEELKFVASQFRRLHSRQVQQTFRFYSQQWRTWAACFIQAAWRRHLRRKIAELRRKEEEEEEMDYEDDEYYDDNMGGMVTRSDSSVGSSSTLRSTVFASRFAANALKGHKLRVTESSKSLMNLTKPSEPDFEALDTDDLN

>AtCNGC9

MLDCGKKAVKSQVISGRLEKFVRLDSMDSRYSQTSDTGLNRCTLNLQGPTRGGGAQGNNVSSGSFKKGFRKGSKGLWSIGRSIGLGVSRAVFPEDLKVSEKKIFDPQDKFLLLCNKLFVTSCILAVSVDPLFLYLPFVKDNEKCIGIDRKLAIIATTLRTVIDAFYLFHMALRFRTAFVAPSSRVFGRGELVIDPAQIAKRYLQQYFIIDFLSVLPLPQIVVWRFLYISKGASVLATKRALRSIILVQYIPRFIRLYPLSSELKRTAGVFAETAWAGAAYYLLLYMLASHIVGAIWYLLALERYNGCWTKVCSNSSLDCHRNFLFCGNEKMDGYAAWTTIKDSVLQLNCPVNTTDNPPFDFGIYLRALSSGIVSSKSFVSKYFFCLWWGLQNLSTLGQGLETSTYPGEVIFSIALAIAGLLLFALLIGNMQTYLQSLTIRLEEMRVKRRDSEQWMHHRMLPPELRERVRRYDQYKWLETRGVDEENLVQNLPKDLRRDIKRHLCLALVRRVPLFENMDERLLDAICERLKPCLYTESSYLVREGDPVNEMLFIIRGRLESVTTDGGRSGFFNRSLLKEGDFCGEELLTWALDPKSGSNLPSSTRTAKALTEVEAFALIADELKFVASQFRRLHSRQVQHTFRFYSQQWRTWAAIFIQAAWRRYVKKKKLEQLRKEEEEGEGSVTSIRATFLASKFAANALRKVHKNRIEAKSTIELVKYQKPSEPDFSADDTS

>AtCNGC10

MAFSHDNRVRFKDEGKPLSSEYGYGRKARPSLDRVFKNVKWGFKKPLSFPSHKDPDHKETSSVTRKNIINPQDSFLQNWNKIFLFACVVALAIDPLFFYIPIVDSARHCLTLDSKLEIAASLLRTLIDAFYIIHIVFQFRTAYIAPSSRVFGRGELVDDAKAIALKYLSSYFIIDLLSILPLPQIVVLAVIPSVNQPVSLLTKDYLKFSIIAQYVPRILRMYPLYTEVTRTSGIVTETAWAGAAWNLSLYMLASHVFGALWYLISVEREDRCWQEACEKTKGCNMKFLYCENDRNVSNNFLTTSCPFLDPGDITNSTIFNFGIFTDALKSGVVESHDFWKKFFYCFWWGLRNLSALGQNLQTSKFVGEIIFAISICISGLVLFALLIGNMQKYLESTTVREEEMRVRKRDAEQWMSHRMLPEDLRKRIRRYEQYRWQETRGVEEETLLRNLPKDLRRDIKRHLCLDLLKKVPLFEIMDEQLLDAVCDRLRPVLYTENSYVIREGDPVGEMLFVMRGRLVSATTNGGRSGFFNAVNLKASDFCGEDLLPWALDPQSSSHFPISTRTVQALTEVEAFALTAEDLKSVASQFRRLHSKQLQHTFRFYSVQWRTWSVSFIQAAWRRYCRRKLAKSLRDEEDRLREALASQDKEHNAATVSSSLSLGGALYASRFASNALHNLRHNISNLPPRYTLPLLPQKPTEPDFTANHTTDP

>AtCNGC11

MNLQRRKFVRLDSTGVDGKLKSVRGRLKKVYGKMKTLENWRKTVLLACVVALAIDPLFLFIPLIDSQRFCFTFDKTLVAVVCVIRTFIDTFYVIHIIYYLITETIAPRSQASLRGEIVVHSKATLKTRLLFHFIVDIISVLPIPQVVVLTLIPLSASLVSERILKWIILSQYVPRIIRMYPLYKEVTRAFGTVAESKRVGAALNFFLYMLHSYVCGAFWYLSSIERKSTCWRAACARTSDCNLTVTDLLCKRAGSDNIRFLNTSCPLIDPAQITNSTDFDFGMYIDALKSGVLEVKPKDFPRKFVYCFWWGLRNISALGQNLETSNSAGEIFFAIIICVSGLLLFAVLIGNVQKYLQSSTTRVDEMEEKKRDTEKWMSYREIPEYLKERIRRFEDYKWRRTKGTEEEALLRSLPKDLRLETKRYLFLKLLKKVPLLQAMDDQLLDALCARLKTVHYTEKSYIVREGEPVEDMLFIMRGNLISTTTYGGRTGFFNSVDLIAGDSCGDLLTWALYSLSSQFPISSRTVQALTEVEGFVISADDLKFVATQYRRLHSKQLQHMFRFYSLQWQTWAACFIQAAWKRHCRRKLSKALREEEGKLHNTLQNDDSGGNKLNLGAAIYA

>AtCNGC12

MNHRRSKFARIDSMGVDGKLKSVRGRLKKVYGKMKTLENWRKTVLLACVVALAIDPLFLFIPLIDSQRFCFTFDKTLVAVVCVIRTFIDTFYVIHIIYYLITETIAPRSQASLRGEIVVHSKATLKTRLLFHFIVDIISVLPIPQVVVLTLIPLSASLVSERILKWIILSQYVPRIIRMYPLYKEVTRAFGTVAESKWAGAALNLFLYMLHSYVFGAFWYLSSIERKSKCWRAACARTSDCNLTVTDLLCKRAGSDNIRFLNTSCPLIDPAQITNSTDFDFGMYIDALKSGVLEVKPKDFPRKFVYCFWWGLRNISALGQNLETSNSAGEIFFAIIICVSGLLLFAVLIGNVQKYLQSSTTRVDEMEEKRRDTEKWMSYRVIPEYLKERIRRFEDYKWRETKGTEEEALLRSLPKDLRLETKRYLYLDMLKRVPWLNIMDDGWLLEAVCDRVKSVFYLANSFIVREGHPVEEMLIVTRGKLKSTTGSHEMGVRNNCCDLQDGDICGELLFNGSRLPTSTRTVMTLTEVEGFILLPDDIKFIASHLNVFQRQKLQRTFRLYSQQWRSWAAFFIQAAWRKHCKRKLSKTRDNENIPQGTQLNLASTLYVSRFVSKALQNRRKDTADCSSSPDMSPPVPHKPADLEFAKAEA

>AtCNGC13

MAFGRNNRVRFRDWISEGTEYGYGRNKARPSLNTVLKNVRRGLKKPLSFGSHNKKRDSNSSTTTQKNIINPQGSFLQNWNKIFLFASVIALAIDPLFFYIPIVDGERHCLNLHRNLEIAASVLRTFIDAFYIIHIVFQFRTAYISPSSRVFGRGELVDDPKAIAIKYLSSYFIIDLLSILPLPQLVVLAVIPNVNKPVSLITKDYLITVIFTQYIPRILRIYPLYTEVTRTSGIVTETAWAGAAWNLSLYMLASHVFGALWYLISVEREDRCWREACEKIPEVCNFRFLYCDGNSSVRNDFLTTSCPFINPDDITNSTVFNFGIFTDALKSGIVESDDFWKKFFYCFWWGLRNLSALGQNLNTSKFVGEIIFAVSICISGLVLFALLIGNMQKYLESTTVREEEMRVRKRDAEQWMSHRMLPDDLRKRIRRYEQYKWQETRGVEEENLLRNLPKDLRRDIKRHFCLDLLKKVPLFEIMDEQLLDAVCDKLKPVLYTENSYAIREGDPVEEMLFVMRGKLMSATTNGGRTGFFNAVYLKPSDFCGEDLLTWALDPQSSSHFPISTRTVQALTEVEAFALAADDLKLVASQFRRLHSKQLQHTFRFYSVQWRTWGASFIQAAWRRHCRRKLARSLTEEEDRFRNAITKRERNAASSSSLVATLYASRFASNALRNLRTNNLPLLPPKPSEPDFSLRNP

>AtCNGC14

MIRFLILFFKHKLYFFFWLVDGCDRFYGDEKQTIEVGEKRVPLFKSTTAPFMKQEVLPKKSKTRLKIPRFGRFKVFPENFEIERDKILDPGGDAVLQWNRVFLFWCLVALYVDPLFFFLSSVKRIGRSSCMTTDLKLGIVITFFRTLADLFYVLHIVIKFRTAYVSRTSRVFGRGELVKDPKLIARRYLRSDFIVDLIACLPLPQIVSWFILPSIRSSHSDHTTNALVLIVLVQYIPRLYLIFPLSAEIIKATGVVTTTAWAGAAYNLLQYMLASHILGSAWYLLSIERQATCWKAECHKESVPLQCVTDFFDCGTLHRDDRNNWQNTTVVFSNCDPSNNIQFTFGIFADALTKNVVSSPFLEKYLYCLWFGLQNLSSYGQNLSTSTSVLETMFAILVAIFGLVLFALLIGNMQTYLQSITVRLEEWRLKRRDTEEWMGHRLLPQNLRERVRRFVQYKWLATRGVDEETILHSLPADLRRDIQRHLCLDLVRRVPLFAQMDDQLLDAICERLASSLSTQGNYIVREGDPVTEMLFIIRGKLESSTTNGGRTGFFNSITLRPGDFCGEELLAWALLPKSTVNLPSSTRTVRALEEVEAFALQAGDLKFVANQFRRLHSKKLQHTFRYYSHQWRTWAACFVQVAWRRYKRKKLAKSLSLAESFSSYDEEEAVAVAATEEMSHEGEAQSGAKARHHTSNVKPHFAATILASRFAKNTRRTAHKLKDVEIPMLPKPDEPDFSVDD

>AtCNGC15

MGYGNSRSVRFQEDQEVVHGGESGVKLKFKINGTQINNVKMMSKGKFLKAKVLSRVFSEDLERVKTKILDPRGQTIRRWNKIFLIACLVSLFVDPLFFFLPVMRNEACITIGVRLEVVLTLIRSLADAFYIAQILIRFRTAYIAPPSRVFGRGELVIDSRKIAWRYLHKSFWIHLVAALPLPQVLIWIIIPNLRGSPMTNTKNVLRFIIIFQYVPRMFLIFPLSRQIIKATGVVTETAWAGAAYNLMLYMLASHVLGACWYLLAVERQEACWRHACNIEKQICQYRFFECRRLEDPQRNSWFEWSNITTICKPASKFYEFGIFGDAVTSTVTSSKFINKYFYCLWWGLKNLSSLGQNLATSTYAGEILFAIIIATLGLVLFALLIGNMQTYLQSTTMRLEEWRIRRTDTEQWMHHRQLPPELRQAVRKYDQYKWLATRGVDEEALLISLPLDLRRDIKRHLCFDLVRRVPLFDQMDERMLDAICERLKPALCTEGTFLVREGDPVNEMLFIIRGHLDSYTTNGGRTGFFNSCLIGPGDFCGEELLTWALDPRPVVILPSSTRTVKAICEVEAFALKAEDLQFVASQFRRLHTKQLRHKFRFYSHQWRTWAACFIQAAWRRHRKRKYKTELRAKEEFHYRFEAATARLAVNGGKYTRSGSDSGMMSSIQKPVEPDFSSE

>AtCNGC16

MSNLHLYTSARFRNFPTTFSLRHHHNDPNNQRRRSIFSKLRDKTLDPGGDLITRWNHIFLITCLLALFLDPLYFYLPIVQAGTACMSIDVRFGIFVTCFRNLADLSFLIHILLKFKTAFVSKSSRVFGRGELVMDRREIAIRYLKSEFVIDLAATLPLPQIMIWFVIPNAGEFRYAAHQNHTLSLIVLIQYVPRFLVMLPLNRRIIKATGVAAKTAWSGAAYNLILYLLVSHVLGSVWYVLSIQRQHECWRRECIKEMNATHSPSCSLLFLDCGSLHDPGRQAWMRITRVLSNCDARNDDDQHFQFGMFGDAFTNDVTSSPFFDKYFYCLWWGLRNLSSYGQSLAASTLSSETIFSCFICVAGLVFFSHLIGNVQNYLQSTTARLDEWRVRRRDTEEWMRHRQLPDELQERVRRFVQYKWLTTRGVDEEAILRALPLDLRRQIQRHLCLALVRRVPFFAQMDDQLLDAICERLVPSLNTKDTYVIREGDPVNEMLFIIRGQMESSTTDGGRSGFFNSITLRPGDFCGEELLTWALVPNINHNLPLSTRTVRTLSEVEAFALRAEDLKFVANQFRRLHSKKLQHAFRYYSHQWRAWGTCFIQAAWRRYMKRKLAMELARQEEEDDYFYDDDGDYQFEEDMPESNNNNGDENSSNNQNLSATILASKFAANTKRGVLGNQRGSTRIDPDHPTLKMPKMFKPEDPGFF

>AtCNGC17

MELRKDKLLMFYSEGKESKEAKWAVNDPMSKSYKLSLPSALRPDNLLPGNRLRYTDASKSKSSKVSWYKTILDPGSEIVLKWNWVFIVSCMVALFIDPLYFFVPAIGGDKNYPCARTDTSLSILVTFFRTIADLFYLLHIFIKFRTGFIAPNSSTRVFGRGELVMDPKAIAWRYIKSDFIIDLIATLPLPQIVIWFVISTTKSYRFDHNNNAIALIVLLQYIPRFYLIIPLSSQIVKATGVVTKTAWAGAAYNLLLYMLASHVLGAAWYILSVDRYTSCWKSRCNGEAGQVNCQLYYLDCDSMYDNNQMTWANVTKVFKLCDARNGEFKYGIFGNAITKNVVSSQFFERYFYCLWWGLQQLSSYGQNLSTTMFMGETTFAVLIAIFGLVLFAHLIGNMQTYLQSLTVRLEEWRLKKRDTEEWMRHRQLPEELRNRVRRYEQYKWLATRGVDEEVLLQSLPTDLRRDIQRHLCLDLVRRVPFFSQMDDQLLDAICERLVSSLCTEGTYLVREGDLISEMLFIIRGRLESSTTNGGRTGFFNSIILRPGDFCGEELLSWALLPKSTLNLPSSTRTVRALVEVEAFALRAEDLKFVANQFRRLHSKKLQHTFRFYSHHWRTWAACFIQAAWRRYKRRVMENNLTAIESMENEEGEVGEELVVVEEEECVEESPRTKMNLGVMVLASRFAANTRRGVAAQRVKDVELPRFKKPEEPDFSAEHDD

>AtCNGC18

MNKIRSLRCLLPETITSASTAASNRGSDGSQFSVLWRHQILDPDSNIVTYWNHVFLITSILALFLDPFYFYVPYVGGPACLSIDISLAATVTFFRTVADIFHLLHIFMKFRTAFVARSSRVFGRGELVMDSREIAMRYLKTDFLIDVAAMLPLPQLVIWLVIPAATNGTANHANSTLALIVLVQYIPRSFIIFPLNQRIIKTTGFIAKTAWAGAAYNLLLYILASHVLGAMWYLSSIGRQFSCWSNVCKKDNALRVLDCLPSFLDCKSLEQPERQYWQNVTQVLSHCDATSSTTNFKFGMFAEAFTTQVATTDFVSKYLYCLWWGLRNLSSYGQNITTSVYLGETLFCITICIFGLILFTLLIGNMQSSLQSMSVRVEEWRVKRRDTEEWMRHRQLPPELQERVRRFVQYKWLATRGVDEESILHSLPTDLRREIQRHLCLSLVRRVPFFSQMDDQLLDAICGCLVSSLSTAGTYIFREGDPVNEMLFVIRGQIESSTTNGGRSGFFNSTTLRPGDFCGEELLTWALMPNSTLNLPSSTRSVRALSEVEAFALSAEDLKFVAHQFKRLQSKKLQHAFRYYSHQWRAWGACFVQSAWRRYKRRKLAKELSLHESSGYYYPDETGYNEEDEETREYYYGSDEEGGSMDNTNLGATILASKFAANTRRGTNQKASSSSTGKKDGSSTSLKMPQLFKPDEPDFSIDKEDV

>AtCNGC19

MAHTRTFTSRNRSVSLSNPSFSIDGFDNSTVTLGYTGPLRTQRIRPPLVQMSGPIHSTRRTEPLFSPSPQESPDSSSTVDVPPEDDFVFKNANLLRSGQLGMCNDPYCTTCPSYYNRQAAQLHTSRVSASRFRTVLYGDARGWAKRFASSVRRCLPGIMNPHSKFVQVWTRVLAFSSLVAIFIDPLFFFLLLIQQDNKCIAIDWRATKVLVSLRSITDLIFFINILLQFRLAYVAPESRIVGAGQLVDHPRKIARHYFRGKFLLDMFIVFPIPQIMILRIIPLHLGTRREESEKQILRATVLFQYIPKLYRLLPLLAGQTSTGFIFESAWANFVINLLTFMLAGHAVGSCWYLSALQRVKKCMLNAWNISADERRNLIDCARGSYASKSQRDLWRDNASVNACFQENGYTYGIYLKAVNLTNESSFFTRFSYSLYWGFQQISTLAGNLSPSYSVGEVFFTMGIIGLGLLLFARLIGNMHNFLQSLDRRRMEMMLRKRDVEQWMSHRRLPEDIRKRVREVERYTWAATRGVNEELLFENMPDDLQRDIRRHLFKFLKKVRIFSLMDESVLDSIRERLKQRTYIRSSTVLHHRGLVEKMVFIVRGEMESIGEDGSVLPLSEGDVCGEELLTWCLSSINPDGTRIKMPPKGLVSNRNVRCVTNVEAFSLSVADLEDVTSLFSRFLRSHRVQGAIRYESPYWRLRAAMQIQVAWRYRKRQLQRLNTAHSNSNR

>AtCNGC20

MASHNENDDIPMLPISDPSSRTRARAFTSRSRSVSLSNPTSSIEGFDTSTVVLGYTGPLRTQRRPPLVQMSGPLTSTRKHEPLFLPHPSSDSVGVSSQPERYPSFAALEHKNSSEDEFVLKHANLLRSGQLGMCNDPYCTTCPSYYNRKAAQIPTSRVSALFDSTFHNALYDDAKGWARRFASSVNRYLPGIMNPHAKEVQTWTKFFALSCLLAIFIDPLFFFLIKVQEQNKCIMIDWPMTKAFVAVRSVTDVIFTMNILLQFRLAYVARESTVVGAGQLVSHPKKIALHYLKGKFFLDLFIVMPLPQILILWIIPAHLGASGANYAKNLLRAAVLFQYIPKLYRLLPFLAGQTPTGFIFESAWANFVINLLTFMLAGHVVGSCWYLFGLQRVNQCLRNACGNFGRECQDLIDCGNGNSSVLVRATWKDNASANACFQEDGFPYGIYLKAVNLTNHSNLFTRYSYSLFWGFQQISTLAGNQVPSYFLGEVFFTMGIIGLGLLLFALLIGNMQNFLQALGKRNLEMTLRRRDVEQWMSHRRLPDGIRRRVREAERFNWAATRGVNEELLFENMPDDLQRDIRRHLFKFLKKVRIFSLMDEPILDAIRERLKQRTYIGSSTVLHRGGLVEKMVFIVRGEMESIGEDGSVLPLYEGDVCGEELLTWCLERSSVNPDGTRIRMPSKGLLSSRNVRCVTNVEAFSLSVADLEDVTSLFSRFLRSHRVQGAIRYDSPYWRLRAARQIQVAWRYRRRRLHRLCTPQSSYSL

>OsCNGC1

MTAKNILMVIVICQYVPRLIRIIPLYLQITRSAGIITETAWAGAAFNLLIYMLASHVLGALWYLLSIQREDTCWKDACSRHDGCDSGSLFCGSNAARNNSFLQDFCPTNGTDNADPTFGIYLPALQNVSQSTSFFEKLFYCFWWGLQNLSSLGQNLKTSTYTWENLFAVFVSTSGLVLFALLIGNVQTYLQSASVRIEEMRVKRRDTEQWMAHRLLPDNLKERILRHEQYRWQETRGVDEEGLLSNLPKNLRREIKRHLCLSLLMRVPMFENMDEKLLDAMCDRLKPMLYTEGSCIIREGDPVNEMLFIMRGNLESMTTNGGQTGFFNSNIIKGGDFCGEELLTWALDPTSASNLPSSTRTVKTLSEVEAFALRADDLKFVATQFRRLHSKQLQHTFRFYSQQWRTWAACFIQAAWHRYCRKKLEDTLFEKEKRLQAAIVSDGSSSLSLGAALYASRFAGNMMRILRRNATRKARLQERVPARLLQKPAEPNFFAEDQ*

>OsCNGC2

MMMGREDKYVRFEDWRSEQSVMSPRRHNALSSLKERTAGVFAFLGNLVHSETLKRLVLHERKLTTRTLHPQGPFLQSWNKIFVLSCIFAVSVDPLFFYIPVINDNNTCWYLDKKLEITASVLRFFTDIFYILHIIFQFRTGYIASSLTTFGRGVLVEDRYAIAKRYLSTYFLIDVFAVLPLPQVVILVVLPNLGGSEVTKAKNILMFIVICQYVPRLIRIRPLYLQITRSAGVITETPWAGAVLNLLIYLLASHVLGALWYLLSIERKDACWRDMCSNNSTVCNQAYLYCGDKENSILRTACLPIDSNDIDPNFGIYVPALNNVSQSTNFLAKLFYCVWWGLQNLSSLGQNLKTSTYAWENLFAVFVSISGLVLFALLIGNVQTYLQSAHLREEEMRVKSRDTDQWMSYRLLPENLKERIRRHEKYRWHQTSGVDEELLLMNLPKDLRRAIKRHLCLSLLMRVPMFENMDDQLLNALCDRLKPVLYTEGSCIIREEDPVNEMLFIMRGNLMSMTTNGGRTGFFNSDVLKGGDFCGEELLTWALDPTSVSSLPSSTRTVKTMSEVEAFALRAEDLKFVATQFRRLHSKQLQHTFKFYSQHWRTWAACFIQAAWHRYCRKKIEDSLREKEKRLQFAIVNDGATTLSFRAAIYASRFAGNMMRILRRNATRKARLKESVPARLLQKPAEPNFAAEEQ*

>OsCNGC3

MRVKSRDTDQWMSYRLLPENLKERIRRHEKYRWHQTSGVDEELLLMNLPKDLRRAIKRHLCLSLLMRVPMFENMDDPLLDALCDHLKPVLYTEGSCIIREEDPVYEMLFIMRGNLMSMTTDGGITGFFKSDVLKGGDFCGEELLTWALDPTSVSRLPSSTRTVETMSEVEAFALTAEDLKFVATQFRRLYRKQLRHTFRAPLNCSSEIVEEQENTLF*

>OsCNGC4

MSYASGGGGGGELATKRSAFHIDYGGGVSLRRLAQPEALARGMITQGSAQLRTLGRSLRTGAAMAVVFQEDLKNTSRKIFDPQDRLLVRLNRSFVVSCIVSIAVDPVFFYAPQVTANGGNLCVGISRDLAISASVVRTVVDLFFAARIVLQFRTAYIAPSSRVFGRGELVIDTAQIAARYFRRFFAADLLSVLPLPQIVIWKFLHRSKGAAVLSTKDALLIIVFLQYIPRVVRIYPLSSELKRTSGAFAESAYAGAAYYLLWYMLASHIVGASWYLLSIERVSDCWKKACNEFPGCNKIYMYCGNDHQKGFLEWRTITRQYINETCEPRDGVMPFNYGIYTPAVRSDVIKSNDFTSKLLYCLWWGLANLSTLGQGLQTSIYTGEALFSIFLATFGLILMAMLIGNIQTYLQSMTVRLEEMRVKRRDSEQWMHHRLLPQELRERVRRYDAYKWVNTRGVDEEVLVANLPKDLRRDIKRHLC

LGLVRRVPLFANMDERLLDAICERLRPALYTERTFIIREGDPVDQMLFIIRGCLESITTDGGRSGFFNRSLLEESDFCGEELLTWALDPKAGLSLPSSTRTVRALSEVEAFALHSDELKFVAGQFRRMHSKQVQHTFRFYSQQWRTWAATYIQAAWRRHLKRRAAELRRREEEEEEAAAIRSSTGLKTTMLVSRFAANAMRGVHRQRSRRADEVLMMPMPKPSEPDFGADY*

>OsCNGC5

MFGSCGGGYRTQTINGRKGTFVRLEQQEDQERQPAATYTMDGSGGGGRVQHVMDSYFSSAPKIRTRSVRMAAAGVMSIGGYRAERLKSIGRVFQEDLTNMSQKIFDPQDAFLVRMNRLFVMACIVSVAVDPLFFYLPAVTATDSNTCIGFDRGLATGATAVRSAIDLFYLARIALQFRTAYIAPSSRVFGRGELVIDPAAIARRYVRRFFVVDLLSVLPLPQIPIWNFLHRPKGADLLPTKNALLFIVLVQYIPRLVRFYPITSELKRTTGVFAETAFAGAAYYLLLYMLASHMVGAFWYLLSIERLDDCWRENCRVLKFHQCKKYMYCGGGNLGQSGFLEWRTMIRQVLVMECAPADEAGTGFQYGIFTTAIQSGVVSTTNLVAKVLFCLWWGLQNLSTVGQGLKTTHYKGEALFAIFLAVFGLILMALLIGNMQTYLQSMTLRLEEMRLRRRDSEQWMRHRVLPVDLQERVWRHDQYRWLETRGVDEDSLVRSLPKDLRRDVKRHLCLRLVRRVPLFANMDERLLDAICERLKPSLCTEATYILREGDPVDEMLFIIRGRLESSTTDGGRMGFFNRGLLKEGDFCGEELLTWALDPKAAANLPLSTRTVKAISEVEAFALHADELKFVAGQFRRLHSKQLQQTFRFYSQQWRTWASCFIQAAWRRHLKRRAAEQRRREEEEEEEAASASSSCQITTTVLVSRFAKNAMRGAQRQRSRRDANLIVLPKPPEPDFQTMEY*

>OsCNGC6

MFDSAHKAQYIDGQREMFKRLDESSPRSSVPSEVGGRSTLKFSMPSFGYDSFNPVRSFLSGVRKGSGRLKSLRQSLTSGAPKTAFAEDLKSFKKTIFDPQEKFLFQMNWFCFLSCVFAVAVDPLFFFLPIIDGDDKSSCIGIDKKLAVTSTIIRTILDLVYLIRVFLQFRTAYVAPSSRVFGTGELVIDPMRIAIRYLKSYFVMDFFALLPLPQIVVWRYLHTLDGPDVPSTKNALVWVVLFQYIPRLLRIFPVTKDLKRTAGVFIETAWLGAAYYLLWFMLAGHNVGTLWYFLTIEREDSCWRSNCHSNDGCNKSYLYCSDNHTGNYTSWLSKRTELLSACSTNSFQFGIFEQALVSGILRPGNFISKICYCFWWGLQNLSTLGQGLQTSIYPGEVLFSIAICVIGLILFALLIGNMQTYLQSVAIRLEEMRVKKRDAEQWMHHRSLPPQIRERVRRYERYRWLETRGVDEENLVQTLPKDLRRDIKRHLCLGLVKRVPLFENMDERLLDAICERLRPTLYTENEYILREGDPVDEMHFILHGCLESETTDGGRSGFFNKVQLKEGAFCGDELLTWALDPKSAANFPASTRTVKALTEVEAFALCAEELKFVASQFRRLHSRQVQHTFRFYSQHWRTWAACFIQAAWRRYYKRKMAEQHRKEEEAANRQSSSSHHPSLAATIYASRFAANALRGVHRLRSRASPTIVRLPKPPEPDFAVDEAD*

>OsCNGC7

MACNGSRAVRFQNDMELPHWKTSSVPECTSSSRSTKHGKAQHQQQQHHDPRKWRRGGGGGGSLKDRVLSRAFSEELESLMSSGANHLFFDPRGQLIHLWSKIFLAACLASLFVDPLFLYLTGTRQNMCIELKYSLAFTLSMIRSLLDLFYAAHIFFRFRTAFIAPSSRVFGRGELVIQPCKIARRYLAGTFWFDLVTALPLPQFVIWIVIPKLKESATANRKNILRFSIIFQYLPRLFQIFPLSRQIVMATGVMTETAWAGAAYNLILYMLASHVLGALWYLFSVQRQEACWREACHVEGPSCQTLFFDCKTVSSNRTMWYELSNITSLCTPSNGFYQFGIYGEALDNGLTSSSFTQKYFYCFWWGLKNLSCLGQNLSTSLFIGEITFATVIGVLGLVLFALLIGNMQATMVRLEEWRTKRTDMERWMNHRQIPQPLKQCVRRYHQYKWLATRGVDEEALLEDLPMDIRRDIKRHLCLDLVRRVPLFDEMDERMLEAICERLRPALYTRGTRLVRELDPVDSMLFIIRGYLDSYTTQGGRSGFFNSCRIGAGEFCGEELLPWALDPRPAASLPLSTRTVRAVSEVEAFALVADDLRFVASQFRRLHSARIRHRFRFYSHQWRTWAACFIQAAWRRNKRRRASMELRMREGGEARPGGSVRCRRHSCDGKALIKKPMEPDFTVEEED*

>OsCNGC8

MDMQIGVGVTAVRTVADLFYLAHMILKFRTAFVAPSSRVFGRGELVRDPDQIAIRYLKNDFIIDLAAMLPIPQVIIWFVIPAVNNSSANHTNNTLSMIVLIQYIPRVFLIVSLNSKIVKSSGVVTRTAWAGAAYNLLLYTLASHVLGALWYLLSIERQYTCWMDVCTRENGTNPAIPKCYMSYLDCKTLEDPIRMDWHSRSEIDHQCLLPEATYVYGLFADALNLDVAKVNFWDKYLYCLWWGFRNLSSYGQNLENSTYRGETIFCILICIMGLVFFSHLIGNMQTYLQSMTVRLEEWRVKRRDIEEWMRHRQLPLELQERVRRFFQYKWLATRGVDEESILQSLPLDLRREIQRHLCLALVRRVPFFSQMDEQLLDAICERLVSSLSTKDAYIVREGDPVSEMLFVIRGELESSTTDGGRTNFFSSITLRPGDFCGEELLTWALMPNPSLNFPQSTRTVRSVTEVEAFALRAEDLKYVANQFKRLHSKRLQHAFRYYSHQWRSWGACFVQGAWRRYKKRKLARELSKQEELYYMQGQGGDDGDGHDDSDSAPLLGAGVGAGGDHRDGAAAGAAHLGATFLASKFAKNTKKSAAAHHGKARMEDVSSIKFPKLAKPDEPDFSLSSDDVL*

>OsCNGC9

MFGSRVQDEVEMQRRTTNRIFPDERQDQFKLPFQAARADRFGVNRIDAKTTEKIKVISEGNIPWHRRILDPGSSMVLMWNRVFLGSCLFALFIDPFFYYLPLVHVLDESTNRSCIAKDRRLSITITVLRTFADLFYMLNIMVKFHTAYVDPKSRVLGKGELVLDLKKIQRRYLRTDFFIDLLATIPLPQVTVWIIMPSIKNSDYNIRNTTFALVIMIQYIFRMYLIVPLSNQIIKAAGVVAKSAWLGAAYNLLYYMLASHITGAIYYLLSIERQITCWNQQCLNESCSFNFISCDNTGSSSYLTWGKNTSIFDNCDPNRNSSANPPPFNYGMFSTALSKGAVSAPFLEKYFFCLWWGLLQLSSSGNPLQTSAYIAENTFAIAIGALSLVLFAQLIGNMQTYLQSISKRLEEWRLRQRDMEEWMRHHQLPDELQDRVRRFVQVKWLATRGVEEESILQALPADIRRDVQRHLCLDLVRRVPFFSEMDYQLLDAICERLVSFLCPERTYISREGDPVNEMLFVIRGKLESSTTNGGRSNFFNSIILRPGDFAGEELLTWALLPKTNVHFPLSTRTVQSLTEVEAFALRAEDLKFVANQFRRLHSKKLQHTFRFYSHHWRTWAACFIQAAWRQHQRRKLAESLSRWESYSWWPEEHPPADKPKQEGTSSSTKTIAESAIAQMHKFASASRRFRADDTAIRRLQKPDEPDFSADHFD*

>OsCNGC10

MFGAGKVDDEMALKRQRTVRFYDEKAKPTIPTHQKQAGFAASKLGVASSGKNKIFVPGEELWYKRILDPSSDFILTWNHIFLFSCFVALFIDPLYFYVPKISYGTPNSCIGTDRHLAITVTFFRSISDLLYFTHIIIKFRTAYINPSSTMRVFGRGDLITDPKEIAWQYLRSDFVVDAVAALPLPQILIWFVIPAIKYSTDEHNNNILVLIVLAQYFPRLYLIFPLTYEIVKTTGVVAKTAWQGAAYNMLLYMIASHVLGALWYLLSVDRQTACWKSNCKNETGCDIKFLDCDVIPNQNWASKTAIFNTCDATNTSISFDYGMFQPALFNQAPGQRFLMKYFYSLWWGLQNLSCYGQTITVSTYIGETLYCIFLAVLGLVLFAHLIGNVQTYLQSITVRVEEWRLKQRDTEEWMRHRQLPHELRERVRRFIQYKWLATRGVNEESILQALPADLRRDIKRHLCLGLVRRVPFFSQMDNQLLDAICERLVSSLCTQGTYIVREGDPVTEMLFIIRGKLESSTTNGGRTGFFNSTTLKSGDFCGEELLGWALVPKPTVNLPSSTRTVKALIEVEAFALQAEDLKFVANQFRRLHSKRLQHTFRYYSHHWRTWASCFIQAAWRRYKRRKMARDLSMRESFCSMRSDDSNGEDDSPPKQNLAMKIMSGSRKGPQNMKELPKLRKPDEPDFSAEPCE*

>OsCNGC11

MFGSRRVKDEMELRKQRTVRFHEERAKPTIPTHQKQAGLATSKLGLGISEKNKIFLAGNELWYKKIIDPSSDFILTWNYVLRIACFVALFMDPLYFYVPKIYYGTPNSCIGRDTRLAIIVTVFRSITDLFYVLQIIIKFRTAYINPSSTLGVFSRGDLVTDPGNIAKHYLRSSFVVDLVASLPLPQIIIWSVIPSVKYSLSEHDDDILLLIALFQYVLRLYLVFSLNSKIVEVTGAFSKTAWQGAAYNLLLYMIASHVLGALWYLLSVDRQTACWEKYCSKEAGCQNRYLACDIQSDSNWKISTAIFNKCDATNKTIDFDFGMFTPLLSNQAPDQGFLKKFFYCLWWGLQNLSCYGQTLTVSTYIGETLYAIFLAVLGLVLFAHLIGNVQTYLQSITARVEEWRIKQRDTEEWMRHRQLPQKLRERVRRFVHYKWLATRGVDEESILKALPADLRRDIKRHLCLDLVCRVPFFSQMDGQLLDAICERLVSSLSTVGTYIVREGDPVTEMLFIIRGKLESSTTDGGRTGFFNSITLKTGDFCGEELLGWALVPKPTVNLPSSTRTVKTIVEVEAFALRAEDLKFVASQFRRLHSRKLQHTFRYYSHHWRTWAACFIQAAWRRYKRRRLAKDLSIRESFFSRRSFEDDGSPEHSLVLNAVRKGAHIIKELPKFRKPSEPDFSAEHDD*

>OsCNGC12

MSDQERDDIPMLLRNVELPTFPPRSTSMCIPVRDDEYEEDTFVPHTGPLFVQPPTQTAAAGIPFTNTPDMPPRPPQGKQVNKPHAIMPEEIGGNRWSYSGNVPKNEHLMMSGPLGQCDDPDCVNCPPACKNKRHFHRGSSTLDSKFHNFLCEHGGGWKKEIERFLSRIPVMNPHAKVVQQWNQFFVISCLVAIFIDPLFFFLLSVQKDNKCIVLNWHFATALAVVRSVTDAIYFLHMLLQFRLAYVAPESRVVGAGDLVDEPKKIAVRYLRGYFLLDFFVVLPLPQVMILLVIPKYVGLSTANYAKNLLRITVLLQYVPRIIRFVPLLGGQSDSSANGFIFESAWANFVINLLMFVLAGHVVGSCWYLFGLQRVNQCLRNACSASKIPSCDGFIDCGRGINIGKQNQLSRQQWFNDSASTACFDTGDNGFHYGIYEQAVLLTTEDNAVKRYIYSLFWGFQQISTLAGNLVPSYFAWEVLFTMAIIGLGLLLFALLIGNMQNFLQALGRRRLEMQLRRRDVEQWMSHRRLPEDLRRRVRRA

ERFTWAATQGVNEEELLSNLPEDIQRDIRRHFFRFLNKVRLFTLMDWPILDAICDKLRQNLYISGSDILYQGGPVEKMVFIVRGKLESISADGSKAPLHEGDVCGEELLTWYLEHSSANRDGGRMRFHGMRLVAIRTVRCLTNVEAFVLRASDLEEVTSQFSRFLRNPRVQGAIRYESPYWRTIAATRIQVAWRYRNRRLKRAGMSKLNDQSYNSALERGARECDARQHGRV*

>OsCNGC13

MSGQERDDVPMLELQRFPTRSVSMCIPVRDDIYEDSIISHSGPIFTPAPTQYTSVAIPSGNRDMLDKLPRPKVKSKPHVVTPEEVGISNWPYDQHVPKNKHLMMYSEPLGLCDNPDCVDCPRACKNKRHFQRSLAPFDNKFHNILYGYGDRWKKKAGHYLSYIPIMKPHDKAVHRWNQFFVISCLLAIFNDPLFFFLLSVDKDYKCIVFNWNFAIALAVGRSVTDAIYFLHMLLQFRLAYVAPESRVVGTGDLVDEPMKIAMRYLRGFFVLDLFVVLPLPQVMILLVIPKYVGLSSANYAKNLLRATVLLQYVPRIIRFVPLLGGQSTNGFIFESAWSTFVINLLMFVLAGHVVGSCWYLFGLQRVNQCLRDSCAASNISKALCNNCTDCGITGINRTNWLNNSDLTGCFDTKSGNFPYGIYQQAVLLTTEPGLKRYIYSLFWGFQQISTLAGNLIPSYFVWEVIFTMAIIGLGLLLFALLIGSMQNFLQALGKRRLEMQLRRRDVEQWMSHRRLPEDLRRRVRSAERFSWVATRGVNEEELLSNLPEDIQRGIRRHFFGFLKKVRLFNLMDNATWDAICDKLRQNLYITGSDILYQGGPVEKMVFIVRGRLESISADGNKSPLQEGDVCGEELLSWYLEQSSVNRDGGKIKLHGMRLVAIRTVRCLTNVEAFVLRARDLEEVTSQFSRFLRNPLVLGTIRYESPYWKNLAANRIQVAWRYRKRRLKRAEMQRLQ*

>OsCNGC14

MPSLSFLRFLSGRSLADVCDGVKRRLGLGDDEGRDEEAGLAGGSSRPAAAAAVAGPPGECYACTQPGVPSFHSTTCDQVHSPDWDADAGSSLVPVQAQPSAAHHAAAAAARWVFGPVLDPRSKRVQRWNRWILLARAAALAVDPLFFYALSIGRAGQPCVYMDAGLAAAVTALRTAADLAHLAHVLLQFRVAYVSRESLVVGCGKLVWDPRAIAAHYARSLKGLWFDLFVILPIPQVIFWLVIPKLIREEQIKLIMTMLLLLFLLQFLPKVYHSIYIMRKMQKVTGYIFGTIWWGFGLNLFAYFIASHIAGGCWYVLAIQRVASCLQEECKIKNTCNLTSLACSKEMCFHLPWSDKNGLACNLTSFGQQNIPDCLSGNGPFAYGIYKGALPVISSNSLAVKILYPIFWGLMTLSTFGNDLEPTSNWLEVIFSIINVLSGLMLFTLLIGNIQVFLHAVLARKRKMQLRFRDMEWWMRRRQLPSRLRQRVRKYERERWAAITGDEEMEMIKDLPEGLRRDIKRYLCLELVKQVPLFHGMDDLILDNICDRLRPLVFSSGEKVIREGDPVQRMVFVLQGKLRSTQPLAKGVVATCMLGAGNFLGDELLSWCLRRPSLDRLPASSATFECVETAQAFCLDAPDLRFITEQFRYKFANEKLKRTARYYSSNWRTWAAVNIQLAWRRYKARTTTDLASAAQPPSAGGPDDGDRRLRHYAAMFMSLRPHDHLE*

>OsCNGC15

MASSSAAAASSAHGVGVVQRLWLEEQERKPPPKRGGGKRRWAWAPLEPRRAGWWAREWDRAYLLACAAGLMVDPLFLYAVSVSGPLMCVFLDGWFAAAVTVLRCTVDAMHAWNLLMRLRAAVRPPEEDDGADEEVAAERGAGGNGGGPAPAQVARPVSRKGLMLDMFVILPVMQVIVWVAAPAMIRAGSTTAVMTVLLVSFLFEYLPKIYHAVRLLRRMQNTYVFGTIWWGIALNLMAYFVAAHAVGACWYLLGAQRATKCLKEQCAQGGSGCAPGALACAAPLYYGGAVGGVGADRLAWALDASARGTCLDSGDNYQYGAYKWTVMLVANPSRLEKILLPIFWGLMTLSTFGNLASTTEWLEIVFNIITITGGLILVTMLIGNIKVFLNAATSKKQAMQTRLRGVEWWMKRKKLPQSFRHRVRQHERQRWAATRGVDECRIVRDLPEGLRRDIKYHLCLDLVRQVPLFQHMDDLVLENICDRVKSLVFPKGEIIVREGDPVQRMLFIVRGHLQSSQVLRTGATSCCTLGPGNFSGDELLSWCMRRPFLERLPASSSTLVTMESTEAFGLEAADVKYVTQHFRYTFTNDRVRRSARYYSHGWRTWAAVAVQLAWRRYKHRKTLASLSFIRPRRPLSRCSSLGEEKLRLYTAILTSPKPNPNQDDLV*

>OsCNGC16

MSGELSTRASTSSSSSSPPGDARGPEHGGTPRGEVSSKRRLVLRRRQRWRRLGGGAAASWAAADPRARWVREWNRAYLLACAAGLMVDPLFLYAVSLSGPLMCVFLDGWLAAAVTALRCMVDAMHAWNIVTQLRVSRAGRERACAAGPDEEQPEAEAAAPAPAADADAAASNKLRDHGRYRKWLVLDFFVILPVMQVVVWVAAPAMIRAGSTTAVMTVMLVAFMLEYLPKIYHSVVFLRRMQNQSGHIFGTIWWGIALNLIAYFVAAHAVGACWYLLGVQRATKCLKEQCLLAGLPACASSTAAVACVDPLYYGAAVASVGGDRLAWGGNATARNVCLSSGDNYQYGAYKWTVMLVSNPSRLEKMLLPIFWGLMTLSTFGNLESTTEWVEIVFNIMTITGGLILVTMLIGNIKVFLNATTSKKQAMQTRLRGLEWWMEHKGVPHGFRQRVRQFERQRWAATRGVDECQIVRDLPEGLRRDIKYHLCLDLVRQVPLFHHMDDLVLENICDRVKSLIFPKGEIIVREGDPVQRMLFIVRGHLQCSQVMRNGATSWCTLGPGNFSGDELLSWCMRRPFMERLPASSSTLVTAESTEAFGLEAGDVKYVTQHFRYTFTSDKVRRSARYYSHGWRTWAAVAVQLAWRRYKHRKTLASLSFIRPRRPLSRCSSLGEEKLRLYTAILTSPKPNQDDDF*

>ZmCNGC1 GRMZM2G148118

MAGREERYVRFHDWKSEQSVSVISDRVVSEKGHNIFGLLKDRTAGAFSFLGNSSHSEALNKLGLGEKSKTKVLDPQGPFLQRWNKIFVISCLFAVFVDPLFLYVPVIDGGNNCLYLDKKLETTASILRFFTDIFYLLHILFQFRTGFIAPSSRVFGRGALVKDTFAIAKRYLSTLFLVDFLAVLPLPQVFVLVVLPKLQGPEVMKAKIVLLVIIICQYVPRLLRIIPLYLQITRSAGILTETAWAGAAFNLIIYMLASHGFGALWYILSIQREDTCWRQACINQTGCDPTSLYCGYHSLANNSFLQNACPTNSTANPDPIFGIFLPALQNVSQSTSFFEKLFYCFWWGLQNLSSLGQNMKTSTNTLENLFAVFVSTSGLVLFALLIGNVQTYLQSASVRIEEMRVKRRDTEQWMAHRLLPENLKDRIMRHEQYRWQETRGVDEEGLLKNLPKDLRREIKRHLCLSLLMKVPMFENMDEQLLDAMCDRLKPMLYTEGSCIIREGDPVNEMLFIMRGTLESTTTNGGQTGFFNSNVLKGGDFCGEELLTWALDPTSASNLPGSTRTVKTLSEVEAFALRADDLKFVATQFRRLHSKQLQHTFRFYSQQWRTWAACFIQAAWHRYCRKKLEEALYEKEKRLQAAIVSDGTTSLSLGAALYASRFAGNMMRILRRNATRKARLQERVPARLLQKPAEPNFFAEDS

>ZmCNGC2 GRMZM2G129375

MLDERKSTTGTLHPQGPFLQKWNRIFVISCIFAVSVDPLFLYIPVINDEKPCWYLDRKLEKAASVLRFFTDIFYILHIIFQFRTGFIASSHTTFGRSVLIEDRYAITKRYLSTYFFIDVFAILPIPQVIILVVLPNLHGSKVMKAKNVLMLIIICQYVPRLIRIRPLYLQITRSAGVITETARAGAAFNLLLYMLASHVLGALWYLLSIQRQDSCWRQYCRGNSTCDPAYLYCGDYDKDGKNAFLTTNCLLSNQSNLPDPYFGIYAPAIKNVSRSKSFFAKLFFCVWWGLQNLSSLGQNLKTSTYTWENLFAVFVSISGLVLFALLIGNVQTYLQSASLRVEEMRVKSRDTDQWMSYRHLPENLKERIRRYEQYRWQETSGVDEEQLLMNLPKDLRRDIKRHLCLKLLMRVPLFENMDEQLLDAMCDCLKPILYTEGSCVIREGDPVNEMLFVMRGNLMSMTTNGGRTGFFNSDVLKAGDFCGEELLTWALDPTSTSSLPSSTRTVKTMSEVEAFALRAEDLRFVATQFRRLHSKQLQHTFRFYSQQWRTWAACFIQAAWHRYCRKKIEDSLREKEKRLQFAIANDSSTSLSFMAALYASRFAGNMIRILRRNATRKARLQERVPARLLQKPAEPNFSAEEQ

>ZmCNGC3 GRMZM2G066269

MKPTSARVLDPRGSFLQTWNKVFVISCLVSVSVDSLFLYAPAIDGDIGCLYLDDKLEKIACLLRSLTDALYLLRMAFQFSTAFAAPTPPGAFGRGVLVDDLLAIAKHYLSTYFLVDVLAILPLPQVFVWVVRPHLQSSEVMNAKNVLMFMILLQYVPRLVRIIPLYLEITRSAGTVVDTAWPGAAFNLLVYILASHVLGALWYILAIQREDTCWREACNSQEGCDLASLYCGSTASGNNSTFLQDACPTDGDGADVDPIFGIYLPALQNVSQSSGFFQKLFYCFWWGLQNLCSYGQNLKTSTYIWENLFAVFVSMSGLVLFALLIGNVQTYLQSASGHIEEMRVRRRDMEQWMSYRLLPEHIKERILRHHQYRWQETQGVDEEGLLVNLPKDLRRDIKRHLCLSLLKRVPMFENMDDQLLDAMCDRVKPMLYTEGSHIVREGDPVNEMFFIMRGRLESTTTDGGRAGFFNSNVLEGGDFCGEELLTWALDPASGSNLPSSTRTARTLSEVEGFSLRARHLRFVASQYRRLHSKQLRHTFRFYSHQWRTWAACFVQAAWHRYCRRRLEEGVREKERMFRAAAVTDISSSRSLGAALYAAHFARNMVRTLRRNAARKARLLDTVSSRLLQKPAEPNFFAEED

>ZmCNGC4 GRMZM2G023037

MFDSTQKAQYMDGHRERFIRLDESSPRSSVPSEVGGRSTLRSSMPGFGYGPFNALRSFLSGGSGRLKSLRQSLTSGAPKTAFAEDLKSYKRTIFDPQDKLLFRMNWVFFSSCLFAVAVDPLFFFLPIINDSNCIGIDKKLAVTSTIIRTVIDFVYLIRVCLQFRTAYVAPSSRVFGTGELVIDPMLIAKRYIKSYFAMDFVALLPLPQIVVWRYLHIPDGPDVLTTKTALVWVVLIQYIPRLLRIFPVITDLKRTAGVFIETAWAGAAYYLLWFMLAGHNVGTLWYFLTIEREDDCWHLYCDDPNFGLGCNSSYLYCNNHHHGSYDSWLTNNSAQVFNMCNGGQDNPFNFGIYEQALVSKILSPGNFISKLCYCFWWGLQNLSTLGQGLLTSTYPGEVLFSIAICVLGLILFALLIGNMQSYLQSVAIRLEEMRVKKRDAEQWMHHRSLPLDIRHRVRKYERYRWLETRGVDEETLVQTLPKDLRRDIKRHLCLGLVKRVPLFENMDERLLDAICERLRPALYTENEFILREGDPVDEMHFILHGCLESVTTDGGRSGFFNKVQLKEGSFCGDELLTWALDPKSAANFPVSSRTVQALTEVEAFALCAEELKFVASQFRRLHSRQVQHTFRFYSQQWRTWAACFIQAAWRRYYKRKMAEQRRKEEEAASRPSSSHPSLGATIYASRFAANAMRGVHRLRSKAVPTIVRLPKPPEPDFGVDDAD

>ZmCNGC5 GRMZM2G077828

MSYDQSAFQVDYMGVGAGAGVSASRRRFMPSESLARGVITHGSAQLRTIGRSLRAGATMAAVFQEDLKNTSRRIFDPQDPVLVRLNRAFFISCIVAIAVDPMFFYLPMVTDEGNLCVGIDRWLAISTTVVRCVVDLFFLGRIALQFRTAYIKPSSRVFGRGELVIDTALIARRYMRRFFSADLMSVLPLPQVVIWKFLHRSKGTAVLDTKNSLLFIVFIQYVPRVVRIYPISSELKRTSGVFAETAYAGAAYYLLWYMLASHIVGAFWYLLSIERVSDCWRNACDEFPGCNQIYMYCGNDRQLGFLEWRTITRQVINETCEPKRDGSIPFNYGIYSPAVVSDVLKSKDTTSKLLFCLWWGLANLSTLGQGLKTSIYTGEALFSIALAIFGLILMAMLIGNIQTYLQSLTVRLEEMRVKQRDSEQWMHHRLLPPELRERVRRYDQYKWLNTHGVDEEALVQNLPKDLRRDIKRHLCLGLVRRVPLFANMDERLLDAICERLKPSLCTEHTYITREGDPVDQMVFIIRGSLESITTDGGRTGFYNRSLLEEGDFCGEELLTWALDPKAGACLPSSTRTVMALSEVEAFALHAEELKFVAGQFRRMHSKAVQHTFRFYSQQWRTWAATYIQAAWRRHLKRRAAELRRREDEELEEDEGKSNRIRTTILVSRFAANAMRGVHRQRSRRAVAVSELLMPMPKPREPDFGDDY

>ZmCNGC6 GRMZM2G005791

MMIIIKTYLQSITVRVEEWRLKQRDTEEWMRHRQLPCELRERVRRFIQYKWLATRGVNEESILHALPADLRRDIKRHLCLGLVRRVPFFSQMDDQLLDAICERLVSSLCTKGTYIVREGDPVTEMLFIIRGKLESSTTNGGRTGFFNSITLKPGDFCGEELLGWALVPRPTTNLPSSTRTVKALIEVEAFALQAEDLKFVASQFRRLHSKKLQHTFRYYSHHWRTWASCFIQAAWRRYKRRKMAKDLSMRESFNSVRLDEVDNEDDDSPPKNSLALKFIARTRKVPQNMKELPKITKPDEPDFSAEPED

>ZmCNGC7 GRMZM2G068904

MSAASPRNFRFQNEIEVQSFRTSPLQSLSRKHGKAHDPRKCRLGFRGGCLEKACRNRKPMLKDRVLSRAFSEELESLMHAAGGSHLFFDPRGQLIHLWNKIFLSACLLSLFVDPLFLYLTGTQRNTCVEFKDSLALTLSMVRSLLDLFYAAHILFRFRTAFIAPSSRVFGRGELVIQPYEIARRYLGRTFWFDLVTALPLPQFVIWIVIPRLNEYSRTANTKNILRFSIIFQYLPRLFQIFPLSGRIVMATGVMTETAWAGAAYNLILYMLASHVLGALWYLFSVQRQEACWREACLLVSPTSQTMFFDCKALSSNRTIWYELSNITTSRCTPGNGFYPFGIYEEALYAKLTSSSFTQKYFYCFWWGLKNLSSLGQNLSTSLFIGEITFAIVVGVLGLVLFGLLIGNMQSYLQATMVRLEEWRTKRTDMERWMHHRQIPQPLKQCVRRYHQYQWVATRGVDEEALLQDLPMDIRRDIKRHLCLDLVRRVPLFDEMDERMLDAICERLRPALYTRGTRLMRELDPVDSMLFIIRGYLDSYTTQGGRSGFFNSCRIGAGEFCGEELLTWALDPRPAAKLPLSTRTVRAVSEVEAFALVADDLRFVASQFRRLHSARIRHRFRFYSHQWRTWAACFIQAAWRRYKRRRASMELRVREVRAGGSLLRSRRHSIEGKASIRKPMEPDFTVEEED

>ZmCNGC8 GRMZM2G135651

MFGSRVQDEVEMQRRPNNRIFPDERQNQSKSLYQTTRADRFGANRIDLKNPEKLKVLNESNKPWHQRILDPGSNIVLRWNRVYLVACLFALFIDPFFYYLPLIRQNGNGSSCVAKDQGLSIRITVLRSLADLFYMLNIAIKFHTAYVDPKSRVLGKGELVVDIKKIQRRYIRTDFFVDILAAVPLPQVTVWLIMPAIKSSDYNIRNTTFALVIVIQYVIRMYLIIPLSNQIIKAVGVVAKSAWGGAAYNLLLYMLASHITGAIYYLLSIERQITCWDQQCVAEYNDTHCNFSFISCENNGSNDYSVWANKTKVFANCDATNSSISFNYGMFSSALSKGAVSSPFLEKYFFCLWWGLLQLSSSGNPLVTSAFITENAFAIAIGAISLILFAQLIGKMQTYLQSISKRLEEWRLRQRDMDEWMRHHQLPSHLQERVRRFVQVKWLATRGVEEESILQALPADIRRDVQRHLCLDLVRRVPFFSEMDNQLLDAICERLVSFLCPENTYISREGDPVNEMLFIIRGKLESSTTNGGRSNFFNSIILRPGDFAGEELLTWALLPKTNVHFPLSTRTVRSHTEVEAFALRAEDLKFVANQFRRLHSKKLQHTFRFYSHHWRTWAACFIQAAWRQHQRRKLAESLSRWESYSWWSAEDHPTGDKPRQEGTSSGGGGTRTIAEGAIAHMHKLASASRRFRTEDIAIRRLQKPDEPDFSADHFD

>ZmCNGC9 GRMZM2G141642

MTDQERDDVPMLLRNVELPRFPLRSTSMCIPVRDDDYEEDTFVPHTGPLFVQPSTQTAPGISFTGRDTPDRLPKPSQGKQVSKPHAIMPEEIRGNKWSYSGQVPKNEHLMMSGPLGQCDNPDCVNCPPACKNRRHFQRGSNALDNKIHNILYGHSRGWKKKIEQIMACIPIMNPHAKPVQRWNQFFVISCLIAIFIDPLFFFLLSVRQDGNCIVLNWEIATALAVVRSVTDAIYFLHMLLQFRLAYVAPESRVVGAGDLVDEPKKVAIHYLRGYFLLDFFVVLPLPQVMILLVVPKVGLSAANYAKNLLRVTVLLQYVPRIIRFVPLLDGQSTNGFIFESAWANFVINLLMFILAGHVVGSCWYLFGLQRVNQCLRDACSISTIPYCDSFIDCGRGIGSGLYRQQWFNDSGAEACFNTGNDATFQYGIYEQAVLLTTEDSAVKRYIYSLFWGFQQISTLAGNLVPSYFIWEVLFTMAIIGLGLLLFALLIGNMQNFLQALGRRRLEMQLRRRDVEKWMSHRRLPEDLRRRVRRAERFTWAATQGVNEEELLSNLPEDIQRDIRRHFFRFLNKVRLFTLMDWPILDAICDKLRQNLYISGSDILYQGGTVEKMVFIVRGKLESISADGSKAPLHDGDVCGEELLTWYLEHSSANRDGGKIKFQGMRLVAIRTVRCLTNVEAFVLRASDLEEVTSQFARFLRNPRVQGAIRYESPYWRTIAATRIQVAWRYRKRRLKRAEKSRLSEETYASLGS

>ZmCNGC10 GRMZM5G858887

MPPLAFLRRYLPARLLARACDGGVRGSPGVARDEEAGGSGGLSGRSAGAPSGECYACTQPGVPAFHSTACDQVHSPDWDADAGSSLVPVQAQQQAQPAAAAAQHAARWLFGPVLDPRSKRVQRWNRWILLGRAAALALDPLFFYALSIGRAGRPCLYLDAGLAAAVTALRTCADVAHLAHVLLQFRLAYVSRESLVVGCGKLVWDARAIAAHYARSVKGLCFDLFVILPIPQVIFWLVIPKLIREERVRLIMTILLLMFIFQFLPKVYHSIHIMRKMQKVTGYIFGSIWWGFGLNLFAYFIASHIAGGCWYVLAIQRIASCLQEECKKNNSCDLISLACSKEICFHPPWSSNVNGFACDTNMTSFSQRNVSTCLSGKGSFAYGIYLGALPVISSNSLAVKILYPIFWGLMTLSTFGNDLAPTSNGIEVIFSIINVLSGLMLFTLLIGNIQVFLHAVLARKRKMQLRFRDMEWWMRRRQLPSRLRQRVRKYERERWAAVTGDEEMEMIKDLPEGLRRDIKRYLCLELVKQVPLFHGMDDLILDNICDRLRPLVFSSGEKVIREGDPVQRMVFILQGKLRSTQPLTKGVVATCMLGAGNFLGDELLSWCLRRPFVDRLPASSATFECVEAAQAFCLDAPDLRFITEHFRYKFANEKLRRTARYYSSNWRTWAAVNIQLAWRRYRARASTDLAAMAAPPLAGGPDDGDRRLRHYAAMFMSLRPHDHLE

>ZmCNGC11 GRMZM2G074317

MPRLAFLRRSLPARLLARACGGGGGGDQGSPDQVARDEEAGGSGGMSGRSSAGGPSGGECYACTQPGVPAFHSTTCDQVHSPDWDADAGSSLVPVQGQAQAAAAAAAPRQRHAARWLLGPVLDPRSRRVQRWNRWILLGRAAALAVDPLFFYALSIGRAGQPCLYMDAGLASAVTALRTCADVAHLAHVLLQLRLAYVSRESLVVGCGKLVWDARAVAAHYARSVKGLCFDLFVILPIPQVIFWLVIPKLIREEQVKLIMTILLLMFIFQFLPKVYHSIHIMRKMQKVTGYIFGSIWWGFGLNLFAYFIASHIAGGCWYVLAIQRIASCLQEECKRNNSCDLISLACSKEICFHPPWSSNVNGFACDTNMTSFSQQNVSTCLSGKGSFAYGIYLGALPVISSNSLAVKILYPIFWGLMTLSTFGNDLAPTSNGIEVIFSIINVLSGLMLFTLLIGNIQVFLHAVLARKRKMQLRFRDMEWWMRRRQLPSRLRQRVRKYERERWAAVTGDEEMEMIKDLPEGLRRDIKRYLCLELVKQVPLFHGMDDLILDNICDRLRPLVLSSGEKVIREGDPVQRMVFILQGKLRSTQPLTKGVVATCMLGAGSFLGDELLSWCLRRPFVDRLPASSATFECVEAAQAFCLGAPDLRFITEHFRYNFANEKLKRTARYYSSNWRTWAAVNIQLAWRRYRARTSADLAAPPLVGGPDDGDRRLRHYAAMFMSLRPHDHLE

>ZmCNGC12 GRMZM2G090528

MPVPAELSPPSPHGAPRTRAGEQDDSTLPPTAAATATATRGGTGRKRRRGARDPRASWAWETEWDRAYLLACAAGLLVDPLFLYAVAVSAPLMCVFLDAWFAAAVTALRCAVDAMHASNLLLRLRGACSPRREDTDEEEAQPGRDGGVPGRGTRSKEGVFLDVLVILPVMQVVVWVATPAMIRAGSTTDVMIVLLTAFLLEYLPKIYHAVRVLRRMQGVSGYLFGTIWWGIALNLMAYFVAAHAVGACWYLLGAQRATKCLREQCAQAGSGCAPWALACAEPLYYGATASSVGAARLAWAGNATARGTCLDSADNYQYGAYQWTVMLVANPSRVERVLLPIFWGLMTLSTFGNLESTTEWLEIVFNIVTITGGLILVTMLIGNIKVFLNATTSKKQAMHTRLRSVELWMKRKDLPRSYRHRVRQYERQRWAATRGVDECRIVRDLPEGLRRDIKYHLCLGLVRQVPLFQHMDDLVLENICDRVKSLIFPKGEVIVREGDPVKRMLFIVRGHLQSSQVLRNGAESCCMLGPGNFSGDELLSWCLRRPFLERLPGSSSTLATLESTEAFGLDAADVKYVTQHFRYTFTNDKVRRSARYYSPGWRTWAAVAVQLAWRRYKHRKTLASLSFIRPRRPLSRCSSLGEEKLRLYTALLTSPKPNQDDLL
