# Supplementary material for: Genome-wide identification and expression analysis of the cyclic nucleotide-gated ion channel (CNGC) gene family in Saccharum spontaneum
Source: BMC Genomics. 2023 May 25;24:281. doi: 10.1186/s12864-023-09307-3 (PMC10214738; doi:10.1186/s12864-023-09307-3)
Supplement: Supplementary file 5 — Additional file 5: Supplementary file 4. Promoter sequences of SsCNGCs and alleles. [file 12864_2023_9307_MOESM5_ESM.docx]

Supplementary file 4: Promoter sequences of *SsCNGC*s and alleles.

>SsCNGC1

AGCAAAAACGTCGCTGTCGAAACTGTCCCCGTCCAACGGTGAACTCTAACCATAATAAATGCTAGTAATAAATCAGCCTCCTCTTATCTCTTCCTCCGCTATTCTCCGAGCGAGCAAGGCCGAGGCGGCGTGGCGCGGGCGCGGCCGGCGAGCACGACCAGGCGGGTGTCCGCGGCCGCGGCGATGCGGGCGAGCGCGGCGGGAGCGGCCGGGCCGCGCGCGGCACGAGCGGCCACGGCGGCGCGGGCGAGTGCGGCGGGCACGGTCAGCGCAGCGGCGCGGCCCGCGCGAGCAGGCGGCCGAGCGGGGCGGGCGCGGCCACCGCGGCATGGCCAGCGCGAGCGGCTCGGCGGCGCAGCACGGCCGAACGGGCCGACGCGGCGGAGCAGGGCGGGTCGCGCAGGCCGCGGCGGCGCGACATGGCCAGCGCGAGCAGCAGCGGTCCGGCGGGAGCGCGCAGCAGCGGTCCGGCGAGCAGCGGGTGCGTCTTCGCGGCGGTGCGGGCGGGCGCGGGCACTGCGGCGGGCGCGGCTGCGGCGGCACGCGCAGCGCAGTGTGGCGGACGGCGGCGCGGGCTGCGCCGCTGCGCGGACGACGAGCACGCGCGCGCTCGGCCTGGGCGGACGGCGAGCACGCGTGGTGGTGGACGACGAGCACGCGCGCGTGCAGCCGAGGGCGACCAGGGCGAGCGCGGTGGTGCGGGCGGGCACGGCGTGCGTGTCTGCGGCGGCGCGGCCACGGCGGCGCAGGCGAGCGCGGCGGGCGCGGCGTGCGTGTCTGCGGCGGCGCGGGCGAGCGCGGTGGGCGCGGCGGGCGCGGTGGCGGCGGCACGGGCGAGCGCGGCGGGCAATCAATGCCATGGACGCCGGTCGTTTCTCCCCATCGGCATTTTCATCGTTTCTTGCTGCCTCGTTGCGAGGGAAGACGCGGTTCCTTGCAGGAGAAACGATTTCTTGCTCGCGCTCTCTCTTTCTTCGTTGATTTTCTCGCCGCATTCAATTTTCGCGTACGTGGAGCCTTCTTCAATGCAGATCGAAACCGGCTATTAAATGCCGTTGGGACTGCCCTTATTCCTGCTCACAAACTTGACATAAATTATTATAAAGCCCACTAAAGTTGTTTTTGCCTCCACCAGAGAAGATGCTTCACAAGAGAAGCCGGAGTCAAAATCTATTAGGAGAAGTTTCCTTCCAAACTAACCTTTAACATATAGACAAATTTTGCTCTGCAATATTGAAGAATTGACTAGCCCACACCGCCAACATCAAAATTTGTTAGGTTCCATTACGAAATCATGATCATTTGTTGTAGGACATTGCGACAATTATTTTGTTAATTTTCTCTGATGTAAGGAGAGAGAAGGTGGGAAGATGTCACTTTTGTCATTGTCTTCAGCCTCTTGCTCTCTCTCCTGATGCTTGCCGTCTAGTTCAGACACACGGGCACGAGGTTGCCGGGCACCGAGGCGGTGCCGCCGTGAACCATTCCAGCTTTGAGGGATAGAACGTGGTTGGGAGCGACTCGGTGGCGGCTGGACGCAGGCAGCAAAGACGGTGAGGCCCAACTGCGGATCCGCGCACGCGGCGCGCGTGTGGGTCCGATGCGCAGTGAGGTAGCAGCGACAAGGCGAGCGCTGGCGCAGCTTGGCGCAACTGCAGACCACACGGCGAGTCACGGCGGTGGTGATAGGGCGAGCACTGGAGCAACGTGATGTGACGCGGCACAGGTCGCGCAGCGAGGCACGATGGCGACGATGACAAGGCGAGCACAGGTGTAGCGCGGCGCGGCGCGGCTTGGGGTGCTCGGCGACTTGGCGAGGCACCATGGCGGCGACAGAGGGAGTGCAGGCACAACACTATGACTAGACGACAAACATTAAAACATTGCAAAAAAAAAAGACGACAAACGTTATAATTATTAAAAGCAAAAGGACGAAGACAAAGGCAAAAAAAATGTTCTCACCTTCTCTCTTCTCATATATGAGAAAATCATTTAAATAGA

>SsCNGC1-2C

GATGCAGGTGCCATTGGCGACCATGATGGAAGAAGGACAGGAAGGGTGGGGAGGATGGAGAGAAGAAAGTAGACTCGCATCGGGTGTGGTGTGGAAAGTGGCGCCCGAGTCGGCGATCCAATCAGGACTGGTGGTGGTGCTGAAGGAGCAAGCCAAGGCAGCCGGGTCCCAACCGACAAGCCCAGGCGAGGCACCGGAAGGTGAAGTGGGGTGAAATCCCGGGGAAGCACCAGTGACCATTGCCGCCGACGGGCGAATGCCACTCTGTGTAGGCAGGGACGACTGTGAGGCAGGAAGGTGGAGGTTGCCGACAGGAAGGAGGACGGGCGCGCCCTAGAGAGCAGGAGCCGCGCCAAAGGGTGGGGCGGCAAGGCCGCCTGGCGCTGCGGCAGCAGAGGCGCGCACGGGATCAGCAGCAGTGGTGCCCAAGGGTCCTGCGCCATAGAGTCCGGCGTCCTGGGGTCCCCCGTCCGCACCCGTGCCCGCTACCGGAACGCGACCAGAACTGGGAGGGTGGCAGCACAGGGCGGCAGGAGTTGAGGTGGAAGAGAGGCCGGAAACCGGGGGCCACTGCTGCGGAACGGAGAAGGGCGGGAGGAGAAGCGCCCCACTCCTGGGGGAGCAGAAACAGGGGCGGCTTGGTGCTGGACTGCCCAGCAGGCCCGCTCCAGAGTTAGCGGCAGAGGAGAGCAGGGGAAAAACAGCCGAGGGGGTGCGGACAGCCGGGGGAAAAACAGGGGTGCGGGTGAACTGGAGCGGAGCCAGACATCCTCGTAGATGAGGCCGAGGCGGGCGGGCGTGATGGGGTACGCGCGCGCCAGGCCCGGGAGCACGGGCACATAGACGAGGCGCTCCTGCAGCGCAACCAGCGCCGCTAGGCCGGCCACAGCCACGCCCCCGGCGCTGTACGCCAGCGCCTTCAGCCACCCCACCATCTCCCCTCGGCTGCAGCTGCTGGGGAAGAGGTGAGGGGCGCCCGGCGGCCAGCTGGGGCGTGGAGGCAGCAGCGGCGGGCTGGGCGGCGCCCAGATCAGGGCCGCCAGGTGCTGGAGCGAGCAGCCCCCTGGATGCAGCAGGAGAAGGGCAGGAGGAGGAGAGGGAAGGGCAGGGGCTGTAGAAGGAGGGGTGGGGAGGAGGAGCGCCGGCCATGGCCTCCTGGCCGGCGGCGGCAGCCACCAGGAGGGGCGGCGGCTGGACAGGGGAGAGGAGAGAAAAAAAAACTAGGCTCTGATTACCATGCTAGAAGTTGTGATTCATTCATTTGGGCTAACCCTAGAAGGGTAGCTATATAGGTACCATACATGGGCCACATGGGCCTAGCCTCATGGGCCTAATACAAACACACTCCAACATGAAGTTTGAACTGTGTATGTGAGGAGCTTGTTCATACAATTCATGCCTTGAAGAAACAATAATGTTATCACTTGACCGCCTTCATCCACTCCAACAGCACTGTCATGCTAGATGGAGCTTGATTCAATTTATGTGCTGATTGCAGACGCAGAAGAGGGGAAAGCCCAATAGATAATGTTCTTGGGTAATAGATATTGACAGAAAAGGAAATTCTGAGATTAGCTTCCTGTGCTAGCCTATGCGGCATGGACGTTCACTTTCTCTTGAATCTCTGCAGAGCTTTGCCAACTTTTTTAAAAGGAAATTAAGTTATTCTAGAAGGTTTCATTCTATCATCTCAATGCCAGTTCCCTTCAAGACGTGTCTGAATTGCAATATGACGTGTTTCAGGAAATTCCTTACATGAGCTTTGATTGCATGGCCATAGTGTTGTCAAAAAAAAAAAAAGATTGCATGACCATAGAAAGTTGAATAGCAGTTAATAACATTTGAAGACTTTATTGAGCCTATAAATTTCCGGATGGGATATATACCTTTTATACTTGATTCAATATTACTTTCTTTTAATGTCATGTTTTAATGAACAGCTGCCATACCTTACTATGTTATTAGCATGGTTAACCTTTTGCTGTTGTTTGCAGGTTCCAA

>SsCNGC1-1P

CTTTCCAACAACTCCTAATCTATGCTCTTAAAATTTATGCACTTGATAAATCCTCTCCCGTTGCACATAACTTTGCGCGTAGTAGCGGTCGTGCGGATCCCATGCAGATTGGTCAATCTAAAGGTGGAAGAGTGTGGAAAAGAATAAGAAGTAGGAAGGGGTTTCTGATGCAAATTTACGAGAGAGAGAAAGAACGTATAAAAATGGTTATGATAATTTAGAAATAAGTGTAGGATTTCTGATGTAAAATTGTCTTCTATGTTTTAGAAGTGGATTATTAGAAACTCTTGGAGATGACTTTTTTTTTCTGCCAATATCGTTTTAGGAGTTATAAAACTACATGTTTGTAGGAGAAAAAAATAGGATGCTGTTAGAGATGCTCTAAAGTTAAAACTTCCTTATTTGTTCACTTTTAAAAATTTTCGACCCTAACTGACATTTTCGTAAGTTTGGCATGTGACGGTGTTAACTTTAAGGAAAAAAGACAATGTTGCCCCTCCATTGTTTTCCAGTCAAGTTGTTTCATGAGTGCTCATTTTTTTCTGCTAGCGTTATTTTTGTCATTCCAAGATATTTTGCCACCACATTTTTTTTCTTTTAGCACCATTTATTTTGTTCTCATTTTTTGCTGTAGGAATGCCAAAATATCTTGGAATGGCAAACAAAACAGTAGGAAGCTGCATGGGGAAGAAGTCGGAGGAAAGGGTAAAATTGTCTTTTTTTCCTTAAAGTTAACAGAAAAAAAATAAGTGCCCATGACTGGAAAACAAAGGAGGAATAAAATTGTCTTTTTCCCCTTAAAGTTAACACCATCACATGCCAAACTAATGAAAATGTCAGTTAGGGAAGGAAATTTTTAAGAGTAGATATAAACAGAAAGATTTGAACTTTTTGGGTGCTAAGGGAGAGGAAGTTTTTTTATGTTTAATAGGGAATTTTTCTTAATATAATTCGGCAATTCTCGTTTCTTTAAAAGAAAATATAATTTTTTGATGGTACGCAGTACAAGCAGTTCTCCTTAAAATAACGCGGCCTCGCTTTGGGGTGCCTTCTTCATTTTCACCAAACAATCCGGTACTACTTGTCTGCTTCCTCGCCCTCGCTGCCGCGCTGTGTCTCTCGTGCGGCAGCTCGGCGAGACGGCGACCGCCGACCGCCGCCGCCGCCGCCGCCCTATGCCAGCTGCCCGTCGGTCCCTTCACCAGGTCTTCGTCGGGTCCATCACGATTAAGCTATTTGGCTATTTGTATTCGGATCTGATCCGAGTACAACTGCATACTTGTGTCTACTAACTTCGATTGTCTTTTGCAAAAATTAAAATGTCCTCTTACTTAGTCAGCTTTTGATTCAATTGACCTTGGTTACTTGCATAGTCCCTTCCCTGATCTGCTGCTCATTGGATTGCTTCTTGCTTATGCCCGAGGATTCTATTGCTTCCTACGGCATTTGCTTCTCCGCTCTCTCTCTCTCTCTCTCTCTCTCTCTCTCTCTCTCTCTCTCTCTCCCGTGCGGCAGCTGGGAGACAGCCGACCCCCGCCGCCGGCGCCCTACGCCGGCTGGCCGGGTCTTCGTCGGTCCTTTCGTCAGGTCTTCGTTGGGTGCTTCACAATTAAGTTATTTGGCTATTTGTATTCGGATCTGATCTGAGCACAACTGCATACATGCTTACCAACTTCGATTTTTTTTTGAAAAAAATTAAAATGTGCTTTTACTTGGTCCGCCTTTGATTCAATTGACATTGGTAACTTACACATAGTCCCTTCCTTGATCTGCGCTCATTCGATTGCTTCTTTTGCTTATGCCCGAGGATTCTTACCCATTTCGCTTGCTAGGCAGTTACTGAGAATCTATGTTTTGCACCATCTGCTTTTCGAAGGCTCTTACACCTTCGTGGTCGGCGATTTTCAGGGAGGCTGATACTTCCATGCTGCAGTTGAAGAAGATTTGATCAGCTACTGCCTTCACTGCTGGCCTGTTCTCCTCTGAGTTACAAGGAAGCCA

>SsCNGC2

TCATGCTATGGAACAGTTCCAGGCCGCGGTCGACCATGCCGGAGTGGCTGCAGGCTGCTAGTGCGGCGATGAAGGTGACCTCGTTCGGCTTAGCCTCGTCGCTCGCCACCATGCGGTCGAACAGCGCGATGGCCTCGTCACCGAGCCCATGCATGCCGTAGGCCATGATGAGAACGTTCCAGGTGATGACGTTCCTCCTCGGCAGCCGGTCGAACACGGCCCTCGACAGCGCCAGGCAGCCGCACTTGGCGTACATGTCCACCAGCGCGCTCCCGACGGCGACGTCCGAGTCCAAGGCGTGACGCACCGCGTAGCCGTGGATCTCCTTCCCCCTCGCTGGCGCCGCAAGCATCGCGCAGCCAGGGAGCAGGGTCATGAGTGTGATGTTGTTGGGCATCACCGGCTCTTCATCCGCCCCGGCAATGCCGTCTTCTGTTGCCGCATCCGTGAATCTCCCTTGCTGCTGCATCTCCCGGACCAGCTGGAACGCGTCGCGGATGTGGCCCTGCACGACGCAGCCGGTGATGAGAGTGTTCCAAGAGACCACGTCGCGGGGCTCGATCGCGGCGAAGATCCACCGCGCAGCGTCCATGTCGCCGAGGCGTGCGTACAGGTCCATGAGCGCGTTCTGCACGAACGGGTTGTCCGCCATGCCGCGCTTCACGACGTACCCGTGCACCGCTTCCTTGCCGGCGAAGGTCTCCGAGCGCGCGCACGCGGGCAGCACGCCCGCAATGGTGGTCTCGCTGGGTACGACGCCGGCCTCAGCCTCCATCCGCGCGAAGAGCTCCAGTGCGTCCTCGTCCATGCCAGCCTGCGCATACCCGCAGACCATGGCGTTCCACAGCCCGAGCTGGCGGTGGCCGCCCGGGACCATGTCGAACACCCGCCTCGCCGCGCCCACCCGCTCGTGGCTCGCGTACATGTCCACGAGCGCGCTGGCGACGAACGAGTTGGCGGCGAGGTCGGTGTCCTTGAGGACGTAGGCGTGCATCTCCCGGCCGAGGGAGAGCATCTCCAGCTGCGAGCACGCCGGGAGCGCACTCGCGAACGTGACGCCGTCCGGGCGCACCCCGCGCGCCACCATGTCGTAAAGCACCTCGATGGCCTCGCCGCAGCGCCCGCTCTGCACCAGCAGGCTGACCATGGTGTTCCACGTGACGACGCCGCCGCCCGGCGCGTCGGTGGCGCCGGCGGAGCCGAAGAGCGTCTGCGCGTCGTTGACTAGGCCGAGGCGCGCGTACATGGAGAGCAGCGCGTTGAACGCGAACCGTTCGTCGCCGTCCAGGAACCCGTTCTTGAGAGCGAACGCGTGCGCCTCACGGCCGAGGCGCGTGTCCTCAGCGAGGTGGGAGCATGCGAGCAGGACGCTGACAAGCGTGAATGAGGTGAGCGGGTGGCCCTCCTGGAGCATATCGCGGAGCGCGTCGAGCGCCGGGAGCCAGCGGCGGAAGAGGCAGAGCGCGGCGATGAGGGAGTTAAGGTGACGGCGTCGCGGCTGGGCATGGCATCGAAGAGCGCGAGCGCGGCGGTGAGGTCGCCGCATCGCGCGTAGGCGGTGAGGAGCGCGTTGGAGACGGCCGGGGTGGGCCCATCGAGTAGGTCGTGGCGCAGCGCAGCGCCGTGAATGGACCGGACGGCTGTGAGGGAGCGGAGCGCGGCAGCGGACTTGGCGGCGGGCGGGAGCGCGAAGCGGTCGAGCGGCGCTGAGTGGGAGGAGGACGCCGCCGACAGGGATGATAGCGCGTGGAGAGCGGCGGCGTGGTTGCCCGCGGCAGTGAGAGAGCGGATAGTGGCGGCGGTGGGGGATGCGGTAGGGGTGGCGTGGGTGGTGGAGGTGGGGAGGTGATAGGGAGGGGCGTGGCGGCCATGGAGCGGCGGAGGGGATTGGCCGATTCGGATTTGGGAGCTCAGCTAGAGCCGGCTAGGAGCTCTGCTTCAGCATCGAGTCCATTCGAGTCGAGTGTTTGGGTGTGGATGACGACAGGATAAGCCTGCGAA

>SsCNGC2-1A

TCACCGAGCCCATGCATGCCGTAGGCCATGATGAGAACGTTCCAGGTGATGACGTTCCTCCTCGGCAGCCGGTCGAACACGGCCCTCGACAGCGCCAGGCAGCCGCACTTGGCGTACATGTCCACCAGCGCGCTCCCGACGGCGACGTCCGAGTCCAAGGCGTGACGCACCGCGTAGCCGTGGATCTCCTTCCCCCTCGCTGGCGCCGCAAGCATCGCGCAGCCAGGGAGCAGGGTCATGAGTGTGATGTTGTTGGGCATCACCGGCTCTTCATCCGCCCCGGCAATGCCGTCTTCTGTTGCCGCATCCGTGAATCTCCCTTGCTGCTGCATCTCCCGGACCAGCTGGAACGCGTCGCGGATGTGGCCCTGCACGACGCAGCCGGTGATGAGAGTGTTCCAAGAGACCACGTCGCGGGGCTCGATCGCGGCGAAGATCCACCGCGCAGCGTCCATGTCGCCGAGGCGTGCGTACAGGTCCATGAGCGCGTTCTGCACGAACGGGTTGTCCGCCATGCCGCGCTTCACGACGTACCCGTGCACCGCTTCCTTGCCGGCGAAGGTCTCCGAGCGCGCGCACGCGGGCAGCACGCCCGCAATGGTGGTCTCGCTGGGTACGACGCCGGCCTCAGCCTCCATCCGCGCGAAGAGCTCCAGTGCGTCCTCGTCCATGCCAGCCTGCGCATACCCGCAGACCATGGCGTTCCATGGCCCGAGCTGGCGGTGGCCGCCCGGGACCATGTCGAACACCCGCCTCGCCGCGCCCACCCGCTCGTGGCTCGCGTACATGTCCACGAGCGCGCTGGCGACGAACGAGTTGGCGGCGAGGTCGGTGTCCTTGAGGACGTAGGCGTGCATCTCCCGGCCGAGGGAGAGCATCTCCAGCTGCGAGCACGCCGGGAGCGCACTCGCGAACGTGACGCCGTCCGGGCCCACCCCGCGCGCCACCATGTCGTAAAGCACCTCGACGGCCTCGCCGCAGCGCCCGCTCTGCACCAGCAGGCTGACCATGGTGTTCCACGTGACGACGCCGCCGCCCGGCGCGTCGGTGGCGCCGGCGGAGCCGAAGAGCGTCTGCGCGTCGTTGACTAGGCCCAGGCGCGCGTACATGGAGAGCAGCGCGTTGAACGCGAACCGTTCGTCGCCGTCCAGGAACCCGTTCTTGAGAGCGAACGCGTGCGCCTCACGGCCGAGGCGCGTGTCCTCAGCGAGGTGGGAGCATGCGAGCAGGACGCTGACAAGCGTGAATGAGGTGAGCGGGTGGCCCTCCTGGAGCATATCGCGGAGCGCGTCGAGCGCCGGGAGCCAGCGGCGGAAGAGGCAGAGCGCAGCGATGAGGGAGTTGAAGGTGACGGCGTCGCGGCTGGGCATGGCATCGAAGAGCGCGAGTGCGGCGGTGAGGTCGCCGCATCGCGCGTAGGCGGTGAGGAGCGCGTTGGAGACGGCCGGGGTGGGCCCATCGAGTAGGTCGTGGCGCAGCGCAGCGCCGTGAATGGACCGGACGGCTGTGAGGGAGCGGAGCGCGGCAGCGGACTTGGCGGCGGGCGGGAGCGCGAAGCGGTCGAGCGGCGCTGAGTGGGAGGAGGACGCCGCCGACAGGGATGATAGCGCGTGGAGAGCGGCGGCGTGGTTGCCCGCGGCAGTGAGAGAGCGGATAGTGGCGGCGGTGGGCGGATGCGGTAGGGGTGGCGTGGGTGGTGGAGGTGGGGAGGTGATAGGGAGGGGCGTGGCGGCCATGGAGCGGCGGAGGGGATTGGCCCGATTCGGATTTGGGAGCTCAGCTAGAGCCGGCTAGGGAGCTCTGCTTCAGCATCGAGTCCATTCGAGTCGAGTGTTTGGGTGTGGATGACGACAGGATAAGACTGCGAATGCCGTTGCCGGGTCGTGCCGTCCTGCTGTCTGCTGCTGTGGTGTCCACGGTCCACGCCCCATCTTTTGTTTTCCTCCTGTCCTTCCCCCGCCAACCCGCCACGTCCAGTCCACCACCGCGGCACCACCATCA

>SsCNGC2-2B

CGGCTGTGAGGGAGCGGAGCGCGGCAGCGGACTTGGCGGCGGGCGGGAGCGCGAAGCGGTCGAGCGGGGCTGAGTGGGAGGAGGACGCCGCCGACAGGGATGATAGCGCGTGGAGAGCGGCGGCGTGGTTGCCCGCGGCAGTGAGAGAGCGGATAGTGGCGGCGGTGGGGGATGCGGTAGGGGTGGCGTGGGTGGTGGAGGTGGGGAGGTGATAGGGAGGGGCGTGGCGGCCATGGAGCGGCGGAGGGGATTGGCCGATTCGGATTTGGGAGCTCAGCTAGAGCCGGCTAGGGAGCTCTGCTTCAGCGTCAAGTCCAATCGACTCGAGTGTTTGGGTGTGGATGACGACAGGATAAGCCTGCGAATGCCGTGGCCGGGTCGTGACGTCCTGCTGGCTGCTGCTGTGGAGTCCACGGTCCACGCCCTATCTTTTGTTTTCCTCCTGTCCTTTCCCCGCCAGCCCGCCACGTCCAGTCCACCACCACGGCACCACCATCATGCGGCGAAGACCCCGTGACCGTGAGCGCGAAGCTTCATCGCATTTCTCCCCAAACTCCACCCGCCTACCCAGCCCCGCCTCGAAGGGGAAAGGACGCGCCCGCGTCCCCAAGTCCAAACCCGAGGTAAATCAACCTCCTGCTGTTCCTGCTCCTTCGGTTTGATTCATTTCGGCTTTGCGCTAAGCCGTGGCGTTCAATTCTGCGGTTCGGATAAACGCGGGCGTGCTTGGTTTCCGCGCAATTTGGTGTCGCGTGACTGGTGGATTGTGCCCTAGCTTTCGCTTGGGTTTTGATGCGGGGAACAAATATCTGTACGGCTGGCTGTTCTCTTCTCCATGCTTTATTTTGATCCGTTGTCTAGGTGGAGCTTGTGGCATGTAGGTTGGTGTTTGGGCGATTTGGGGTTTCCTTATTTTGGGGCAAATTCAGGCCGTTGGTGGCGGCATACTGTTAAGAGTTCTACTGTTATATTATATACAAGTCTTTTCTTTTTCGCGAGCGTAAACGTGTTTCATCAATATAGGAAGAAAGAAAGTGTGGCCGCACGCGGAGGGGTCAGGTGGCAATGAGTTTACAAAAGGTTATTACAATTTCTGAGACTGAGAGGCTTATTTTGGAATGTTGTGGGTTATGCTGTTACTCCCAGGCTATGTTTGGGAGCAATTATATTTAGATCATCAAAGATTATTTTCTCTGATACTGGATCTCAATTGGCTGTTTTGACATTGCTAAATTTCTTTCTTTTATCTTCTGATTACTGAAAATACTTTACAGGACAGTTTGCATGCCAGTGTTGGTGTTCTTACTTGGCCTGTGATTATAATCATCCGGTACCTTGATGTATGTTGATATCACGCTTGAAGAGTTGATCTACTCGTGTTAGCAGCTAAATTTGAAGAAAATGATGATGGGAAGAGAGGACAAATATGTGAGGTTAGCCATCCCCTGTTGCTTGAGAAGTTGAGATAGTTTCTAGAGTACATTATTTTGTTGAAGTGTTTTGCGTTTATAGCATGTTATGTTAGCAACACAAAAGCATGTGAAGAATGTGATAGTATTCTAAGTATGGAAACATGTCTATTGCAAGTGAGCATTTGAATTAGATCATCGAGATGGCCACATGGACCATGGACATCAGATGCAGACTGAAATTATCCATGTCTCTTATGTGAGCCATCAGAAAATGCCCACAAAAATATGTGCTTTCGGTAGCAACATTTGCTTCTAGCTTCTAGCCTGCACCCTCTTTCCTGAATTGTTCTATCCCCAAAGCATAGCATGCGACACAGGATATAATGAATGGGGCCCAACTAGACTAATGAAGTAAGAACAGAATTTTAGGCCTTTGGCAACCTGATTACTTAAAATAAAAGGCACTGGGATCCAATTATGGTCTTGAGTAAAGAATGAAGACCAGTTCATTGGTGATTTGGGGAACTTTTAACATGCAACAATTGTCCTGACTACTGAGTGTTTTGTTTGGTTTATTATCTGTGCAGAT

>SsCNGC3

TTTATATGAGAAGGAGAAGAGGTTACAAGCAGCAATTGTAAGTGACGGCACTACTTCGCTCAGTCTTGGTGCAGCGCTCTATGCTTCACGTTTTGCTGGCAACATGATGCGGATCTTACGGAGAAACGCCACCAGAAAGGCCCGTTTGCAGGAAAGAGTACCTGCAAGACTGTTACAAAAGCCAGCAGAACCCAACTTCTTCGCTGAAGATAGCTGAACTTGGACCCTGTAACAAGCAGGCATTCATGATCCAACCGGTAAAATTGTTCACAATTTAAGATTGGATGCTGTAGATAAACAGATCAGGCGCTCTAATGCCGTCTGGTTTAAATCATGTAATTTAGTGTATATTTCGATTGTATACATGTTGCATGTATTCTAAACATCCGAATTCCTCTGGTGCATTTGAAAAAAAAAGAGAAGATGCTGTGAATGTAATCGATCAATTGTTTGTTAACTTATTTCTGATAAGGATGTTTGTTAACTTTGGATATTCTCACGTGTGATTCTGTGGCGAAGTTTGATAGGATCAAAAGTGTTAATAATTTCAAACCACGTGTACATGTGGTAAATTTATGTCTTCTAGCCAGTGGCGGATGCAGGATGAGAATGAAGGGGGGGCTAAAACAATGGAGATGTTGATTTGCACGAAGATTTAACGGTGATTTGAGGTTTTTGCTACAGTGTTTTAGTGCTAAATCAAGTCATTAGGGGGGGCTGGAGCCCACCCAGCCCCCCCTGTAGATCCGCCGCTGCTTCTAGCATATATTTTTTGAGACCAGAGGACCGGTGGAATTTCAAGAAAAAAGCATAATTTTAGGAAAGAATCGCCCTGCCAAAAATAAGACCTATTTAAAGCTCATCAACTCATTAGTTTTTCAATTGTTTTGTCATTCAACTACCAAAACCCACTAGAGTACCTGGATGCCTTAGTAGTGTGTGTGTGTGTTTTGTCTCTCTTCTAAGGCTTCTTTGTACAGTCTTTGTTTCTGTTTAATATAATAATTTCAGGGCATCCCTGCTGTATTTTCTGTTAAAAAAAAGCTAGAGTATTCATTTTCAATGTCATATGTCTTATATCTTGTGCCTACTATCTCATGTTATGTGAACAATCATTTGAATTTTGGCTATAACAATGACTGTTCTGGCCTATAAATTACATGTCTTTCAAAATACTCTCTTATGTCATGTGAACAATGTTTTATCAAATCTTACAGTGTTATCAGCTTATTTGACGACAGAAATAAAGAAAAAGGGTTCGGGCTCCGTTCTTGGTTCACTACTGTCAACATCAGACGCTATTTATTAGCCATTCTGTTCCGTGTCCAGTGGACAGAGCCTAGTGAGCAGGAGAAGCTGGAAGCCCTCGAATTGCTGGCGCCGGCTGACCCAATCGAATCGAGTCCAGGAGTGAGGAGTCCAGCATGCACGGTGAAGCAGACTATCCAAGGTTGTCCTGTTGAGCTTGGTCTTCTCCGCCACCTCGTCGACCCTTGTCCTTTGTAAATGTCCACATCCACGTCCCTGCTCCGTCGTTCCCAACTCCGCCACCTCGGCCGCCACGCCTCCTCGAAAGAAAAGCTGATTCTTTCGAGGTTTTGTTCTCCGTACGTCGGCGATTGGCACTTTGGCAGGTGATTGTCATTCAGAGAGGCGTAAGCTTCACGAATGGAGTAAACTGTTTCTGCCTTGCCGCCGGAAGTGAGGAACCAATGGCACTGTCACTGCGACTGCAAGGGGTAAGCTTCTCTGATCCTATCCTGCATTCATGCATTGCAATGCAATCTTTTGTGAGCTCTATCAAGTTCACGCTGGAGTGGACTGAATCCCCAAGTTTCGATTGGTGATCAATCTTTGCCGAAATGTCTTACTCTGATAACTAAATTTCGTGTGCAGCACGTGCAGGGTTTCAGAACTGGAGATCAGAGCAATTCATTGGATCGGATGACATTGTAACCTGCTAGTGCTAGCATCCTGCGCTGGAAAAAGGAAAGAAAGAAA

>SsCNGC3-2B

GCGACCACATCTCCAAGGTTCAAAGGTTATGACCGCAAAAAACGTATTGATGTTCATAGTCTTGTTCCAATATGTGCCCCGACTGACCAGAATAATACCACTCTACCTTGAAATCACGAGATCAGCTGGTACAGTAATGGATACGGCATGGCCGGGTGCTGCCTTTAACCTTCTAGTTTATATACTCGCTAGTCATGTAAGTCCTCATCTAATCATTTATCAATACTGTTGAAATATAAGTGACCTGTCCACCTTTTTTTTTTCTTATCAACTTAGTCTTTGGGCTGAACTAGTCAGTGCATGCTAGAGGTCTCGATGGCAAATCTCATACAGGCTAAGCCTCTAGATTAACAATTAATTTGCTACATATATCACATTGTGCATGGTCAAGATAGTACTAACCGATGCATTGACTTGTTACAGGTCCTTGGAGCTCTTTGGTACATTCTTGCCATACAACGAGAAGACACCTGTTGGAGAGAAGCTTGTAATAACCAGGATGGTTGTGATCTGGCAACTTTATATTGTGGAAGTACTGCATTTGGAAACAACACTTTCTTACAAGATGCTTGCTCAATAAATGGGGGCGCCGATGTAGATCCAATCTTTGGAATTTATCTACCAGTTCTCCAGAATGTTTCACAATCAACAGGTTTCTTCGAAAAATTATTCTACTGCTTTTGGTGGGGGCTACAAAATCTATGGTACACCCCCAACCCACACACACTACCACCAAAGACTAATGCCCTGGGACTTGTACTTATGTGTTGCCATTTTTATTGCAGTTCCTATGGTCAAAACCTTAAAACAAGCACTTACGTATGGGAGAATCTGTTTGCTGTTTTTGTCTCAATGTCTGGTTTACTTTTGTTTGCTCTCCTTATAGGAAATGTGCAGGTACAGTTTTAACTTTTAAACATTGATGAAATTTTATTGTTATCTGTGCTTTTAGATTAAGTGTCAATTTATTTAAAGTAGATGTTATGTAGCTCTGTATTTGCTGTCTAGAGATGATGTCGCTCCTAGTAGGCTAATCCTTCAGGATTCTATCCTTCCAAGACTTTATAAGAATCTCATGGCCTTATGCTGGACAACATAATCATGTTTGCTGCCTCTAATCATAATTGATACCGTACCAGCTTGGCTATTATCAAACTCTTATCAAAATAGCATGGACTAAATCAACTTTAAGATCTTTGTTTCATTGTTTGCAAGAAATTCAAGTGACATTTTTGATACAATCCTGAATGAGCTAGCTATGAAAACTCATTTCTCTATAAGAATTGGCCATAGAGATTTATCCTTAAAATGATTCCGTATGTTTTGACTTCGTTGTACTTATGCTAGCTATATGCAAGAGGTCATTGTTTAAGGAGTAAGTCTGGACAACATGTTGAATTATATAACTAGTGATGATGTTTGACCTGATCATATTTCTCCAAATTTATTTTCTTTTTCTTGGTTGGATTGAATTGTTGCAGACCTATTTACAATCAGCCTCTGGGCATATAGAGGAAATGAGAGTGATAAGACGTGACACAGAGCAATGGATAGCATACAGATTACTTCCAGAGCATATCAAGCAACGAATATTGCGTCATGATCAATATAGATGGCAAGAAACACAAGGTGTGGATGAAGAGGGTCTTCTTATAAATCTTCCTAAGGATCTCAGGAGGGATATAAAGCGTCATCTTTGTCTATCACTTCTCATGAGGGTACTTCCCTATACCTTTAAGCTTTATTTATTGATAGTCATAAGGAATTGCTTTGCCCTTTGTCCCATTCGATATGTGAGACTCGACCCCATCATGATTCCAATAATGGTTACACGATTCACGCACTGTTTAAACATTACACAATGGTATATCAATTTCAATTTAATCGGCCGCATTCCCCGTTTAACATTTAGCGTTTAGAATAACACTAGTTAATATGTATCACATCGATCCAAAGAAATTAATTTAGTTAATTGACATTAATTCCAACTGGTGTTGCAGG

>SsCNGC4

ACTAACTGGTATTTTTGAAAGCTCATTAGGCTGTAGCCATTTATAGCATTATTAATTGACTAAACCTTTTCTAATCCTGCAGTGGTTGGTTATTCAAGTCAACAGAACTCGTGAACCCACTATTTGCACTACTGGATGCAGAGTTTGCTCATAGTTTAGCTGTTAAAGCTGCTGCCCATGGATTTGTTCCAAGAGAAAAGAGACCTGATCCACCAGTTCTTGGTCGCACGATGACACCATACGCCGTTCGACACCGACGAGGCGAACAACGCAGGCGGGTATGATCTTTTTTATCATCGTTTGCCAAAAGTTTTGACATTTTATTTGATTGAAAACTCTGACAAAAGCTTGTAATACGTTTATATAAATGAATTATACTAATCACTTTATATATATGCCCTAATTCTTGATGGCTAGCTGTACGTGTTCATCATGCATAGGTGGGTACGAGAGGTAGATTAGATAAATCGTTTTAAATTAGTTTGACTTAGATTAGATTAGACAAATCGTATGATTACTTTTTTTTTACGGGAGGTACTGGTATTCTTTTGCGGCCTGCAGGCAAATCAAAGAATAGTAAATAAAGATGCATCCACGTGCATACCGGATTAGGGGTCATTCCTTGATGGGTACCTCAGAGCCACAGATCTTGGCTCCGAGCTCGGAGCCACAGATCTTGGCTCCGAGCTCGGCGCCAAGATCTGTGGCTCCGAGCTCGGCACCAAGATCTATGGCTCCGAGGTACCGGCCACGTCAGCGCCACCTCGGATGCCATGTTGTGCGCGGTCAGACACCTCGGAGTCAAGATCTATGGCGCCGAGATGTGTTACCTCGGAGCCATGAGTTCTGGCGCCGATCCCCAGGGTCCAAAGATGAGTTTACGTCGTCCAGGGAACCAAATGTAATTTTTTTTTTAAAAAGCTAAACAGTAAAAAATTCGGTCCCCGTCCCCGTGTCACGGTCGGAGTAACCGAAGGAGGGCAGAGGCCTCGTTGACTGTGTCGCTCGCCTGTCGCCTCGCCTCCCCGTCCGCTCCGCTCCCGTGCTTTCCCCTTTGCTCGCTCGCGGACCCTCCGACCCCGACTCCGACTCCCAGCGGCGCACTCGCGCCAGCAAGCTCGAATCCGCACGCCGCCGCCGCCCCACGGCGCCCAGGTAACCGTCTAACCCTCTCGGTTCCTCGAAATACTTCCCGGATCCCTTCCACCGCGCGGAGTTCGTCAGGAAACCGTCCGTTTGATTCTGGGAGCTATGGTTCACGCTCTCTTCCTAGACGAGTAGATCTGAATTAGCCTGCGAGGACGACGACCCCAGTACTTCGGCTGCTACCGCCGTTATTAGTCGCGCGGGGGAAACGGTGGCGTAGCCCTGCGTCTTCTCATGCCGCGGCACCCCTAATCGTGGAAGTCAAACAATGACGACGATTACCCAGCGCTGCTCGTACAGAGCTTAGCTGTAGCTTCACTATCTATTTGCTTGATATTGCTGGCTGCTGTAGAACTCTATATTTAGGGGCTTAGGGCGTTACTGTCATGCAAATGGCAGTGTTTGTATGACACCTACAGTACTTGCCATTTCTGTATGCCCATGCTTTATCCGTGAGGTGTATTACTCTTTTGTTGTGGGTGTTGATTGTGCTTGTTGGTCAGTGTATTACTGTTTTTTGCGGGTATTGATTGTGCTTGTTGGCCAATTGTATCAGGATCGGGGTGTTTCTGGTGTGCAGGGGTGGGTGGTTGGCTTCTCAGAACATGTAACAAGTGTTGATATGAAAATAAGTTCCTGGAATAGTGGGTTCATTAGCAGTTTCGCACAACTTTGAAGCTGCAGAGGATTTCAAATGCCCTTTGTCATCCGCTCTCCACCTCCATTGAGTCAGAGGTATGCTGCTGTGCTGGTTTTCTTTGACATCGATTTCATCATGCTTTTAGTTTCGAAATTTCTGCAGTATCTGTAAATTGCGGTCTCTGAATGCTTGATTTAGGGTCTGTATCCCTAATT

>SsCNGC4-2D

CGAAGCAGATCAGATCCATATATGGCCGCTCACGCTGGCACGTGGCGGCGTGGATGGCTGACGATGGTCATGAGCACATGGATGGCTCCAAGGCTCGCGTGTTGCCGAATCAAGATGACGTCGAGTCGATATAAAAAAAAAAAACCAATCTATGATTAGCAAAATCAAAATAGAGATTGATTTGATTCTATCAAAAGGCAAATAAAATATAGGATTTCATCGTGTCTTGTTGACGCAGGTCGACGAGGATGGCTGCGACCGTGGCCTTCGCATCCATGTACGCGTGTACCGTGGCCTTCGCATCCATGTACGCGTGTACTTGTTGATGCGCTAGCTATTAAGCTTTGAATCGATGAGTCGTCCTTCCCCAGTATAGAGGCCGTGGGCCATGGCCGACGGTGCATGCGTGCCTGTGGCCAGTAGGTGTCGTAGTGCTCAATCCAGATGGAAAACAAGAACCAATCCATGATTAGTATAGGCATGAATCATCAACAAATTAAGAATAGCCCACGACCGGATCGGTGATGATTAGCACCACAAAAAAGAGATCAAATCAATAAAAGTAGTATAGGAACAAATTCACCCAGTTGGAAGAGGTGTTGATAACGTGTTAAGAATCGGCCTATCCACGGATTGTCAGATCCGTGTTTCGTTCCTGGTCCTAAGAAAAAGACGTGTAGAAGTTGTAGATTGATTCCGTCTCCCTTGTCTAATCCCAAAAACAATACACAAGAATACACAGGAGACGCAGGTATACTGTGGAACTCATATCTCTTCTTTCATTATAGATCGGTACAGATTCCTTTATATAGTCTTAACCCTTCAGGGGTATATTTGGTATGAGCCCACAATAATATTAGGGTTATTGTTTCTCAACAGACAGAACATTCTCATGCTTCTCCAACAAAGGGGTGTTTAGTTTCTCCCAAAAAAAAATTTCACCCCATCACGGACACATGCATGAAGTATTAAATGTAGACGAAAAAAATAACTAATTACACAGATTGCGATAAATTTACGAGACGAATCTTCTGAGCCTAATTAATCCATGATTTGACAATGTTGTGCTACAGTAACATGTGCTAATGATGGATTAATTAGGCTTAATAAATTCGTCTCGCGGTTTTTAGGCGAATTATGTAATTAGTTTTTTTATTAGTATCAAACACCCCTTCCGATATCGCATGAAACATGAACTAAACACTCCTAAAAAGAAGAAAAATAAAATTTGCCTGGGCTGGGATGACAGGGCCGACCAGCACAATGGGGAATAAATTCCCTGGGCAGCCCACCAGGTCCAGACCACAGCTCCCTGACGGCCCACTAGCTCCCATGCGGCCTCACGGGCCTCGACGTGCGACGGCAGCCGCGGTCAATTTGCGGCTGCGCCTCTTGTGCTCGTCCCCGTCCCCGTGTCGAACGGTTCACGGTCGGAGTAACCGAAGGAGGGCAGAGGCCTCGTTGACTGTGTCGCTCGCCTCCCCGTCCGCTCCGCTCCCGTGCTTTCCCCTTTGCTCGCTCGCGGACCCCCGACCCCGACTCCGACTCCCAGCGGCGCACTCGCGCCAGCAAGCTCGAATCCGCACGCCGCCGCCGCCCCCGCCCCCGGGCGCCCAGGTAACCGTCTAACCCTCTCGGTTCCTCGAAATACTTCCCGGATCCCTTCCACCGCGCGGAGTTCGTCAGGAAACCGTCCGTTTGATTCTGGGAACTATGGTTCACGTCCTCTTCCTAGACGAGTAGATCTGAATTAGCCTGCGAGGACGACGACCCCAGTACTTCGGCTGCTACCGCCGTTATTAGTCGCGCGGGGGAAACGGTGGCGTAGCCCTGCGTCTTCTCATGCCGCGGCACCCCTAATCGTGGAAGTCAAACAGTGACGACGATTACCCAGCGCTGCTCGTGCAGAGCTTAGCTGTAGCTTCACTATCTATTTGCTTGATATTGCTGGCTGCTGTAGAACTCTATATTTAGGGGCTTAGGGCGTTACTGTCATGCAAA

>SsCNGC5

GTTCCTCCATATCCTCATCAACGTCTTCATCACTCTCGTGATCCTCGGAAGCAACACCCGCTTCCATGTCCTCTTCGTCCACCTCGTCTTCGTCGTCTTCATCATCATATTCCTCCTCATTTTCATCATCCTCCCCTGCGGCACCAAACTCTTCCCCAATGTTTTGTTCACTGCGACTACTCCTTCGATCTTTGCCCTTGCCCTTTCCAACCTCCTCCATCCCCATATGCATCAGGTGCAGATACTGATACCTTATCCCCATCATGGGGTGCTTGTCCACAAGCATATGCCGCCGGTATGATTCCCTGAGAACTACTGTCTGTGTCCTCAGCTTGTGTGACAAGTAGAAAATCCCAGGGTGCCTAGCCACCACCTTCCTGAACCCCGGTGGCAGCCGCAGCGCCTCCCCGAGCTTGACCAGCACCTCCTTCTCCATCTTCTTCCCCACTGTGAGACTCAGGACCTCGTGCAGCACCGCCACGGTCCTCTTCTCGGTGATGTCACTCCTTGGCGCCAGGTGTGACCCATCCTCATAGGGCGAGATGTAGGGCAGCCTCTGCCACTCGTCCAGCCATTTGCGCACCTTCTTGTCCAGCTCGAATCCTCTGGGGAACGAGAGTGGGAAGGCGACCGCGTCGCCGACCTTGTAGCCGCCGGTGCGCTGTGCGTAGGCCTGCATCGCCGACACGGCGAGGTCCTTCCTGTAGCAGACGAGCTCGAGGAAGCTGCCGTCCGGGGAGAGCGCGAAGTAGTCCGGGTAGCTGGGGAGCAGCGAGCGTTGGAAGTCCGACGCGAGGCCGAGGTCGAGCCGGAGGCGCGCGACGAGGCGGAGCGGGAGCGCGCGGGAGGGGGCGAGCATGAGCAGCCGAAGCAGGCGGTCTGCGGCGTCGGGGAGGGTGGCGTCCACGACCTGCGCCTCGGCGGCGTGCAGCGCCGAGAGCCGGGGCGTCGGGGAGACCTCGATGGGGTGCGGCGAGAGCGCGAACGCCGAGGGGTATTCCCGCAGGAAGCGCAGCGGGCGGTACGGGAAGGGGATTGTGGAGGGCAGGGAGTGCAGTGGCACGGCGTGCGGCGGCGGCGTGGCGGCGAGGAGCGCGTCCTTGGTGAGAAGGAACGGCACCAGGTCGCCCTCCCGCTCCACGACGTGGTCGAGGGCCCGGTCTCGCACCCACGGCACGCGCGCCTCGAGGAGGCCCCGCGCCGGCACTAGAAGCGCCGCCGGTACACGCCGCAGTGGCGGCAGAGGCATCTCTTCGTGGGGTACTGGGGTTTCCCGGTTTCGGGAGGCGGCCGAAATGCGGCGGCGAGTGCGAACAGGAAGGGGAGGGAGATCGGACGGTGGCCGGTTGTGTCTATTTTTAGGCGGGGCCGTCAGTCGTGGGTGGGTTTGGACCGTGCGGTGCCGAGAGCCTCGGCCCAATTGAGGTCCAGTGGCTTCTTTTTTTTATAAGAGGTCCAGTGGCTTGGCGCAACGGTGGGGAGTGAATGAAACGGCGGGCAAATAGGCAAAAATCTGGCCGTTTCAGCGTCGATTGATGCGGTGATTTGGGAGGAAACTGCGACGTCTCAGGTGATTATACTTCATGTTTCAATGTCCACATCAACAACAGTGCTTGTTTTAAACCTGGATTCTCGCCGTTCGTCGGGCGAATTTCTCTCTGCCCAGCAGCGGATTGCCCTTTTGATGATGATTGTGCAAGAATTTGGTTTTCTGCTACATCGATTAGAAATCCACCCATCTTGGTCTGTCGATAAAATTTTTGTTTGGTCTTCGACGCAGGACGAACTGACCATAATCCTCCGCGACGAAGCGCGAGTGGGCATGGCACGATTGATGGCGACATGATGAATGAATCGAACAGATCCCCTGGAGACCCTGCCCCGGAGCGTCGTGCCCGGTTGCGGCAGCTCATCGCCGGCCATAAGCACACGTTCATGCGCATTGCTTCCCTAGGTTCAATTTGACGACGCCGACGCCGACGCCGACCTTCGGTCCAGGAT

>SsCNGC5-2B

TTCCCTGCACATTCATGCAGTCCAAACAAAGAGATGTACAAGCACCTTCCCTTGCAGAGTGGTTGCCAGTTCAAAATCAATGAGAAATTTGTTCCTCCATATCCTCATCAACGTCGTCATCACTCTCGTGATCCTCGGAAGCAACACCCGCTTCCATGTCCTCTTCGTCCACCTTGTCTTCGTCGTCTTCATCATCATATTCCTCCTCATTTTCATCATCCTCCCCTGCGGCACCAAACTCTTCCCCAATGTTTTGTTCACTGCGACTACTCCTTCGATCTTTGCCCTTACCCTTTCCAACCTCCTCCATCCCCATATGCATCAGGTGCAGATACTGATACCTTATCCCCATCATGGGGTGCTTGTCCACAAGCATATGCCGCCGGTATGATTCCCTGAGAACTACTGTCTGTGTCCTCAGCTTGTGTGACAAGTAGAAAATCCCAGGGTGCCTAGCCACCACCTTCCTGAACCCCGGTGGCAGCCGCAGCGCCTCCCCGAGCTTGACCAGCACCTCCTTCTCCATCTTCTTCCCCACTGTGAGACTCAGGACCTCGTGCAGCACCGCCACGGTCCTCTTCTCGGTGATGTCACTCCTTGGCGCCAGGTGTGACCCATCCTCATAGGGCGAGATGTAGGGCAGCCTCTGCCACTCGTCCAGCCATTTGCGCACCTTCTTGTCCAGCTCGAATCCTCTGGGGAACGAGAGTGGGAAGGCGACCGCGTCGCCGACCTTGTAGCCGCCGGTGCGCTGTGCGTAGGCCTGCATCGCCGACACGGCGAGGTCCTTCCTGTAGCAGACGAGCTCGAGGAAGCTGCCGTCCGGGGAGAGCGCGAAGTAGTCCGGGTAGCTGGGGAGCAGCGAGCGTTGGAAGTCCGACGCGAGGCCGAGGTCGAGCCGGAGGCGCGCGACGAGGCGGAGCGGGAGCGCGCGGGAGGGGGCGAGCATGAGCAGCCGAAGCAGGCGGTCTGCGGCGTCGGGGAGGGTGGCGTCCACGACCTGCGCCTCGGCGGCGTGCAGCGCCGAGAGCCGGGGCGTCGGGGAGACCTCGATGGGGTGCGGCGAGAGCGCGAACGCCGAGGGGTATTCCCGCAGGAAGCGCAGCGGGCGGTAGGGGAAGGGGATTGTGGAGGGCAGGGAGTGCAGTGGCACGGCGTGCGGCGGCGGCGTGGCGGCGAGGAGCGCGTCCTTGGTGAGAAGGAACGGCACCAGGTCGCCCTCCCGCTCCACGACGTGGTCGAGGGCCCGGTCTCGCACCCACGGCACGCGCGCCTCGAGGAGGCCCCGCGCCGGCACTAGAAGCGCCGCCGGTACACGCCGCAGTGGCGGCAGAGGCATCTCTTCGTGGGGGTACTGGGGTTTGGGAGGCGGCCGAAATGCGGCGGCGAGTGCGAACAGGAAGGGGAGGGAGATCGGACGGTGGCCGGTTGTGTCTATTTTTAGGTTTTTTTTTTAGCAGAGTGTCTATTTTTAGGTGGGGCCGTCAGTCGTGGGTGGGTTTGGACCGTGCGGTGCCGAGAGCCTCGGCCCAATTGAGGTCCAGTGGCTTGCAGCAACGGTGGGGAGTGAATGAAACGGCGGGCAAATAGGCAAAAATCTGGCCGTTTCAACGTCGATTGATGCGGTGATTTGGGAGGAAACTGCGACGTCTCAGGTGATTATACTTCATGTTTCAATGTCCATCAACAACAACAGTGCTTGTTTTAAACCTGGATTCTCGCCGTTCGTCGGGCGAATTTCTCTCTTCCCAGCAGCGGATTGCCCTTTTGATGATGATTGGGGTTTTCTGCTACATCGATTAGAAATCCACCCATCTTGGTTTGTCGATCAAATTTTTGTTTGGTCTTCGACGCAGGAGAGATGAACGCGCTCACGAACTGACCATAATCCTCCGCGACGAAGCGCGAGTGGGCATGGCACGATTGATGGCGACATGATGAATGAATCGAACAGATCCCCTGGAGGCCCTGCCCCGGAGCGTCGTGCCCGGCTGCGGCAGC

>SsCNGC5-3C

AATGTTTTGTTCACTGCGACTACTCCTTCGATCTTTGCCCTTGCCCTTTCCAACCTCCTCCATCCCCATATGCATCAGGTGCAGATACTGATACCTTATCCCCATCATGGGGTGCTTGTCCACAAGCATATGCCGCCGGTATGATTCCCTGAGAACTACTGTCTGTGTCCTCAGCTTGTGTGACAAGTAGAAAATCCCAGGGTGCCTAGCCACCACCTTCCTGAACCCCGGTGGCAGCCGCAGCGCCTCCCCGAGCTTGACCAGCACCTCCTTCTCCATCTTCTTCCCCACTGTGAGACTCAGGACCTCGTGCAGCACCGCCACGGTCCTCTTCTCGGTGATGTCACTCCTTGGCGCCAGGTGTGACCCATCCTCATAGGGCGAGATGTAGGGCAGCCTCTGCCACTCGTCCAGCCATTTGCGCACCTTCTTGTCCAGCTCGAATCCTCTGGGGAACGAGAGTGGGAAGGCGACCGCGTCGCCGACCTTGTAGCCGCCGGTGCGCTGTGCGTAGGCCTGCATCGCCGACACGGCGAGGTCCTTCCTGTAGCAGACGAGCTCGAGGAAGCTGCCGTCCGGGGAGAGCGCGAAGTAGTCCGGGTAGCTGGGGAGCAGCGAGCGTTGGAAGTCCGACGCGAGGCCGAGGTCGAGCCGGAGGCGCGCGACGAGGCGGAGCGGGAGCGCGCGGGAGGGGGCGAGCATGAGCAGCCGAAGCAGGCGGTCTGCGGCGTCGGGGAGGGTGGCGTCCACGACCTGCGCCTCGGCGGCGTGCAGCGCCGAGAGCCGGGGCGTCGGGGAGACCTCGATGGGGTGCGGCGAGAGCGCGAACGCCGAGGGGTATTCCCGCAGGAAGCGCAGCGGGCGGTACGGGAAGGGGATTGTGGAGGGCAGGGAGTGCAGTGGCACGGCGTGCGGCGGCGGCGTGGCGGCGAGGAGCGCGTCCTTGGTGAGAAGGAACGGCACCAGGTCGCCCTCCCGCTCCACGACGTGGTCGAGGGCCCGGTCTCGCACCCACGGCACGCGCGCCTCGAGGAGGCCCCGCGCCGGCACTAGAAGCGCCGCCGGTACACGCCGCAGTGGCGGCAGAGGCATCTCTTCGTGGGTACTGGGGTTTCCCGGTTTTGGGAGGCGGCCGAAATGCGGCGGCGAGTGCGAACAGGAAGGGGAGGGAGATCGGACGGTGGCCGGTTGTGTCTATTTTTAGGTGGGGCCGTCAGTCGTGGGTGGGTTTGGACCGTGCGGTGCCGAGAGCCTCGGCCCAATTGAGGTCCAGTGGCTTGCCGCAACGGTGGGGAGTGGCGGGCAAATAGGCAAAAATCTGGCCGTTTCAACGTCGATTGATGCGGTGATTTGGGAGGAAACTGCGACGTCTCAGGTGATTATACTTCATGTTTCAATGTCCACATCAATAGGGATGAAAACGATCGGAAACGATCGATAGGAGGCCAAATCATTTTCGTTTTCACATCTTTTTCTCGAAAACGAAATCGATATGATATTGTCGGAAACGAATACGGCGCCGATATTCCGGTAATTTCGAAAACGATAGTAGTCGATCGGAAAATACATCGATAATGATCGGAATCCATGAGAACGGTATCTAAAAAACGATAAACACGCCAAACTGTAGAACATGAGAAGATTCATATAACTCGACCTATCTCACATGACAATAGTAAATAAAACCACAATTGACAAGATCACAAGCTGAAATCACACAATTCACACAATCTTAGGTCTCAAATAGTGGCAAACCACGCAACAGTATGTCAATTCTTGACAAATATACAAATAAACGATCATGTTCTAAGTTGACTCGACATAGTGGGCATGGCACGATTGATGGCGACATGATAATGAATCGAACAGATCCCCTGGAGGCCCTGCCCCGGAGCGTCGTGCCCGGTTGCGGCAGCTCATCGCCGGCCATAAGCACACGTTCATGCGCATTGCTTCCCTAGGTTCAATTTGACGACGCCGACGCCGACCTTCGGTCCAGGA

>SsCNGC5-4D

TTCCTCCATATCCTCATCAACGTCTTCATCACTCTCGTGATCCTCGGAAGCAACACCCGCTTCCATGTCCTCTTCGTCCACCTCGTCTTCGTCGTCTTCATCATCATATTCCTCCTCATTTTCATCATCCTCCCTGCGGCACCAACTCTTCCCCAATGTTTTGTTCACTGCGACTACTCCTTCGATCTTTGCCCTTGCCCTTTCCAACCTCCTCCATCCCCATATGCATCAGGTGCAGATACTGATACCTTATCCCCATCATGGGGTGCTTGTCCACAAGCATATGCCGCCGGTATGATTCCCTGAGAACTACTGTCTGTGTCCTCAGCTTGTGTGACAAGTAGAAAATCCCAGGGTGCCTAGCCACCACCTTCCTGAACCCCGGTGGCAGCCGCAGCGCCTCCCCGAGCTTGACCAGCACCTCCTTGTCCATCTTCTTCCCACTGTGAGACTCAGGACCTCGTGCAGCACCGCCACGGTCCTCTTCTCGGTGATGTCACTCCTTGGCGCCAGGTGTGACCCATCCTCATAGGGCGAGATGTAGGGCAGCCTCTGCCACTCGTCAGCCATTTGCGCACCTTCTTGTCCAGCTCGAATCCTCTGGGGAACGAGAGTGGGAAGGCGACCGCGTCGCCGACCTTGTAGCCGCCGGTGCGCTGTGCGTAGGCCTGCATCGCCGACACGGCGAGGTCCTTCCTGTAGCAGACGAGCTCGAGGAAGCTGCCGTCCGGGGAGAGCGCGAAGTAGTCCGGGTAGCTGGGGAGCAGCGAGCGTTGGAAGTCCGACGCGAGGCCGAGGTCGAGCCGGAGGCGCGCGACGAGGCGGAGCGGGAGCGCGCGGGAGGGGGCGAGCATGAGCAGCCGAAGCAGGCGGTCTGCGGCGTCGGGGAGGGTGGCGTCCACGACCTGCGCCTCGGCGGCGTGCAGCGCCGAGAGCCGGGGCGTCGGGGAGACCTCGATGGGGTGCGGCGAGAGCGCGAACGCCGAGGGGTATTCCCGCAGGAAGCGCAGCGGGCGGTACGGGAAGGGGATTGTGGAGGGCAGGGAGTGCAGTGGCACGGCGTGCGGCGGCGGCGTGGCGGCGAGGAGCGCGTCCTTGGTGAGAAGGAACGGCACCAGGTCGCCCTCCCGCTCCACGACGTGGTCGAGGGCCCGGTCTCGCACCCACGGCACGCGCGCCTCGAGGAGGCCCCGCGCCGGCACTAGAAGCGCCGCCGGTACACGCCGCAGTGGCGGCAGAGGCATCTCTTCGTGGGTACTGGGGTTTCCCGGTTTTGGGAGGCGGCCGAAATGCGGCGGCGAGTGCGAACAGGAAGGGGAGGGAGATCGGACGGTGGCCGGTTGTGTCTATTTTTAGGGGGCCGTCAGTCGTGGGTGGGTTTGGACCGTGCGGTGCCGAGAGCCTCGGCCCAATTGAGGTCCAGTGGCTTCTTTTTTTTATAAGAGGTCCAGTGGCTTGCCGCAACGGTGGGGAGTGAATGAAACGGCGGGCAAATAGGCAAAAATCTGGCCGTTTCAGCGTCGATTGATGCGGTGATTTGGGAGGAAACTGCGACGTCTCAGGTGATTATACTTCATGTTTCAATGTCCACATCAACAACAGTGCTTGTTTTAAACCTGGATTCTTGCCGTTCGTCGGGCGAATTTCTCTCTGCCCAGCAGCGGATTGCCCCTTTTGATGATGATTGTGCAAGAATTTGGTTTTCTGCTACATCGATTAGAAATCCACCCATCTTGGTTTGTCGATCAAATTTTTGTTTGGTCTTCGACGCAGGAGAGATGAACGCACTCACGAACTGACCATAATCCTCCGCGACGAAGCGCGAGTGGGCATGGCACGATTGATGGCGACATGATAAATGAATCGAACAGATCCCCTGGAGGCCCTGCCCCGGAGCGTCGTGCCCGGTTGCGGCAGCTCATCGCCGGCCATAAGCACACGTTCATGCGCATTGCTTCCCTAGGTTCAATTTGACGACGCCGACGCCGACCTTCGGTCCAG

>SsCNGC6

GATGTCATTGGTGGTGCGGATCATGCGCTCGGCCTTGCCGTTCTGAGATGAGGTATACGGGCAAGACATGCGGAGCTGGACACCATGCGAAAGGAAGAAAGCACGTGAGGTGGAGTTATCGAACTCACGGCCATTGTCGCACTGGACAGCCTTGATGGTAAGGCCGAACTGAGTGGACACCCAAGCAAAGAAGTGGCTGAGAGTGGGGAACGCCTCGGACTTGGCACGCAGGGGAAAGGTCCAGGAATAATGGGAGAAATCATCAACCACCACCAAATAGTACTTATAGCCAGACATGCTGAGAACGGGAGAAGTCCACAAGTCGCAATGAATAAGATCAAAGACACGAGTGGTATGTGAGGAAGAGGACTGAAAGGGAAGGCGAACATGACGGCCGAGCTGGCATGCATGACACAAGTGATCATCGTGAGCCCGAGAGCACCGAATGTCGGAACTACGACTGAGCTGTGTCAGGGCATCCCGGCCAGGATGGCCGAGGCGACGGTGCCAAGTGGTGGTGGAAGAAGTGGCGGCGAAAGCGGCTGAGGAAGGCGAAGGAGGTGAAACAGACGCCGGAAACTGAAGGGTATAGAGAGGCCCAGTGCTGTTACATCGGAGCAAAGGACACCGGGAGGCCAAATCCCGCACGGTGAGACCAGAGGGGTCAAACTCAACCGAACAAGAATTGTCCGTTGTGAACCGGCGAATAGAGAGAAGGTTGTGGACCATACCGGGAGCCACAAGAACATCAGAGATATGAAAGGAGCCGTGGGGACTGGCAGTGCCCACGGAGGAGACAGGAATGCAAGCGCCATTGGCGACCATGATGGATGAGGGACAGGAAGGGTGTGGAGGACGAAGAGAAGAAAGTAGACTCGCATCGGGCGTGGTGTGGAAAGTGGCGCCCGAATCGGCGATCCAATGAGGCCCCCCCACCAGCGGCCCCGGCGCTCGCGGCCATGGTGGTGTCGGTGGCACGAGCGGAAGCGAGGGCGGCGGCGAGGGTGGAGATGGGCTGGAGTGGGTTGGAGGCCGTGCCATCGCCCCCGGTGAGCAGCAGATCGGCTGCAGCAGTTGCAGCGGTGGCGTCACCAGGAAGGGCAGGGTCAACGGCGTCGGCGGGATCGGCCGGATGGGCCGGGTGGGCTGCTGCCGCTGGAGCAACATCAACTGTGAGGGTCAGAGGAGACCCTCCAGGTGCACCCCAGCCCGCAGCAGAGAGGAGGGGGGCTGCAGGCCGCATCCAGGGGAGAAGGGCGCCAGCGCCGGCACCAGGCATCCAGGGGAGATGGGCGCCAGGCAGCTGGGCGGCCCCAGCAGGGGCGCCAGGCAGCTGGGCGGCCCCAGCAGGGGGCTTGGCCGGCTCGGCCCCCTGGCCAAAGGCGCCAGGATGGGCGGCCCCGTGGCCGGCGGCGCTAGGGAGGGCGGCCCCCTGGCCAGGGAGGGCGGCGGCGGCGGCAGCAGAGGAATAGGGCTGGGCGCGGAGGGGCAGCAGCAAACGGGGCCCAGGAAGTAGGGTGGGAGAGGGGGGGGAAGGAGAAGGGAGCAGAGGGCGGCGGCGGCTGGAAGGCCGCCCAGTGGGGCGGCGGCGGCTGGGTGGCCGCCCAGGGGGGCGGCAGCGGCAGGGAGGCCGCCCAGTGGGGCGGCAGCGGCTGGGTGGAAGGTTCCCAGGAGGGAGATGGGAGAGAGGAAGGTGGGGAAGGAGGAGGAGGTGGCCAGCCGGCCATGGGTGGCGGCGGCGCCTGCCTGCCTGGTGCTGGCGGCAGGAAGGGAGGGAGGGGAGAAGGGTTAGGCTGATACCATGTAGAAGATTGTGATTCTATTACTTAGGGCTAACCCTAGAAGGGTAGCTATATATAGTTCCATACATGGGCCACATGGGCCTTAGCCTCATGGGGCCTAAACATACTCCAACACTCACCAAGGCACCACGCTCGCGCGCCACGGCGCCGTCCCGAATGGTGGTGGACGATGATGTCCCGTAACCCAGGAGCAAGAAGA

>SsCNGC6-2D

ATTTCATTTCATCATTCAATCCTACAACTAGCATACACCACACAAGCATGTATATTGAATTTAAACTTGACAGATGCAATCAAGCATATGAAAATGCATATTTAAATGCATCAAACAAGTTCATGAGCTTGCTCCCCCTACTTGTGTGCTCAAATTTTAATTGACCCTTCCTTTGTCATTTCTCTCCCCCTATGTCCATTTTCCCCCTATCACTTTTGTCTTTGTTTCTCTCCCCCTTTGTCATCAATGACCACAAAGGTTCAATTATAGAAGGTTAAGATTATCAGTGTCAATCAATGGGGTTAGGATCATTTTCTCAAATTTGGTTCAATCAAGATCATTTACCAAAGAAATTTAACTCGGTTTGATCCAATGACAAGCTTCTTCACACCTCATTTTAAGGGTTATCTTGACAATGTTAAGTTAAACCCTTTTAGTTTATTTTCTAGATCAAACACTAGGTTCACAAGCCCACAAACATGTCATATGCTACCACTAGATCAAGTCAACCATAGAAGCAATAGTGGTACCATACAAGCATCAAAATTCATTTGATTTTCATGAATGAGCCTATAAAATATGAGGAATGACTCGATGCACTAAACAAGTCCTTAGCAAAGAATATATGCATGTCAATCAACTATTACCTTCGATTGCTCGAAGGAGAGGCATGTCATATAAGTGGGGGTGCATCAACACATAACTGAGAAATCCAATATGTTCAACTCATTCCTTACCTTGTAAAACTTTTTCTCATCAAGTGGCTTGGTGAATATATCGGTAAGTTAATCTTCGGTGCCCACACTCTCAATTGAGATGTCCCCTTTTTGTTGATGATCTCCTATGAAATGGTGGCGGACATCAATGTGCATTGTTCTTACATGTTGAACCGGCTTGTTAGTGAGCTGATAGCACTCTCATTGTCACATAGCAATGGCACTTGTTTGAACTTGATTCCAAAATCGGCCTTCATGCAAAGAATTTGTGCATAACAATTACCGACGGATATGTATTCGGCTTCGGCGGTTGAAAGTACCACACTATTTTGCTTCTTTGATAACCAATATACAAGTGACCTTCCCAATAGTTGACATATGCCCGATGTGTTCTTTCTCTCTACTTTGCATCCTGCATAATCCAAGTGAGCGAAGACGTCGCTCTTGCGAAGATTGGAGTGGTGGTGGCAAGCTCGACCATGGAGGCTTAGAGCTCGGTGGCGCCATGGGTTTGAGGGACTGTGAGAGCGGCGACACACTAGAGTGAGATGGCTCGGAGAAGGAGGATGCGAGGAAAGAATGGGTCTAATAGCTTTTACAGTCTTACAGTGCAAGCTCCGTTTTTTGGGAGGGTGCCACCAGAATTAAGTGGTGTTCGAGAGAAGAAAGGAAACTAGGAAATGTAAGAAATGATCAGAATAGAAGGTGTAGAGGTGGTAGGAGAAAGGGGAGGACGCATAGATTCCAAGGAAGGTTCGCGGTGGCCCAAGGTCATAGGGATGCCAGATTCAAAAATGTTTTCAAAAATGTTTCACTTGTTTTTGACTTAATCTTTTAAACTTTATGCACATGCATTTGGGGAAGTTCTGGAATACAATGCGCCGAACCCCTAAACAACTAGCCCCGGCCGGAGAAATACACTGACAAATCACTAGTACTCTCTCCATTCATTTATTCCATTTTTAGGTGCCTCGCCTATTCTTTAACCGCCGTCACCTCCGCTTACGGCCTCGCCTCCTTTCACGCCGCGCGCGGCGCGCTCGCAAAAGACTCTGCTCTGCAAGATCGATCGGGAGAGACGAGGATCCGAGAGATCGAGCAACTGCTTGCCTGCTGCATTATCGATCGATCGGTTGATCGAGGAATCGCCTCCGGACAGGACAGCATTAATCGATCTATCGGCCGGCCGATTGATCTCACCAAGGCACCAAGTAAGCTCGGGCGCCACGGCGCCGTCCCGAATGGTGGTGGATGATGTCCCGTAACCCAGGAGCAAGAAGACGAT

>SsCNGC7

AAACCCGTTCAAACCAAATACGACGAAGAATGGCCTATTTCTAAGGTGAAACTTCCAATTTACCGAAATTCATACCAAAAAAGTATTCTATCTGTCATAAAATAACTATTATTTTGCATGTAATCAATCTTTTCTGAGTTTGATAAAAAATACGTAAATATTAATATTTCTAATATGTAATAAGTACTATTAGATTAATAGTGAAATATATTTTATAATAAATATATTAAAGTACAGATGTTAATAATATTTTTTCTATAATAAGTTTAAATTTCTTTTGAATCTTTAAAAATGAGTTGTCAAATTATTTGGAACCGGAGGACAGAATATATTTTTGAGAACTAAGGATAGAATATACGGATAGATAGTAATAATTACCAAAGGAATCTTGTAAGGGCCTGTTTGCATCCCTTAAGAGCATCTTCAACAAATCCCCAATACAACCCCTCATCCTCATAAAACAGAATTATTTGAGGAGATAGATCAATTTTCATGCTCCAACAGTGTCCCAATACCTCCCCCAATTTTTGGTGGCCACAAAAAAAACACCCATCTCCCCTCAAATAAAGGGGCACTTGCAACCTCCCCCAATCCTCTGTTCTTTTGGCGCAAACACACCCATTGGTGATTCTGCACTTGGAAACAATGTCATGCCATCTCTCTTTATATCTTGTTTATGATATATCCTTGTGTGAGTATGTTTAATTTCTCTTTCTTATTGTTGAACAAACTGTTATTGCATTTTCGATCTTTGGTCTTAAGTATGCGACAAACAAGACATCAACAACTTATTTTGACGTGAAAAATATACATGGGTCAATTTGCTTCAACATGGTCCAGAATTTACAATATGGGCCAAAATCACATATTGGGGATGAGATATTGGGGAACTGCAAAAGCATCTCCCCCAATAACCCAAAAAGTAAATATTGGGCTACCCCATACTAAATTATTGGGGGAGTTTTATTGGGGGACTGGTGGAGATGCTCAGCACTAAAAATTAGTATTAATGGATCTAAACCGACCAGTAATAACCTGCTAATTATTAGCTGGGGTTGCTAACTATTAGTTTTATTTAGTAGGTTTGTACCTTCAACTCACTGGCCAATCACCTATTAGTAGCTACTCCAAACACTCCAACAAAAAATTACTAGCTAGCTAAAAAATAGCAGCTATTAGCTAGCTAATTTTTAGCGCTAATGGATCCCCTAAGCCCTCTGAGTCAACTCCGTTCTAGATGAACGCTCGTCTCCCTCCCGGCGACTAGTTGCCACTAAACTTTCATAAGGGTCTGCTAAAAAAAACTAAACTTTCATCAGTGAAAAACTCCATTTCCCGGCGATTACTACCGCCTACCGGCTATCGCCGTGTCCCCAGAGGAAAACTGCATTTACCGTCCGCGCTTAGCACTAACCACAGTGAAAACTGCACCACTAACCCCAGGTTTTAACCCCAATCCCGTCAATTAGCGGCGACTAACAAAGTAATTAAGCCGGTGGCGAAGCGTCCGCATCCGCACCAATCCGGTCCGGGAGTTTCCGCTCCCACGCCCCACGCGCGCCGGCCCCGCTCCTTCCTCGAACTCCTCGCCGCCGGCTCTGCTCCACTCCGCCGACTCGCTTCGCTGTCCACCTCGCCCCTGCTTCCTCCTGATCCCGGATTCGCCTCGCCTCGCGGCGACCCGGTACTCGTCCCCGCCACGAGCGCCGTCAAGCGAAGCCGTTCTTCGCCTGGCCGCGCGCCGCTGCCGCATCTGCCAGGCTCCTTCCGCCGGAGCACGTCGCGGCCAGTTGCTCTCTCGCGCTCCGTCCCCTCGCTCGTCCTCCTCCACCGTCGCGGCGGCGGCGGCGGCGGCCTTCGTCCTCCTCCCGGAAGTGCAGATCCGCACGGGGCTCTCGGATGTATTTCTCCTCTTTGCTGGTAAGGAAGCTCGCTCCGAGGGTTAGTTCCGCTCTGGTCTTCCGTTTCTACGCTGCCAGCAATTAAGTTCCAAGTCGTCGA

>SsCNGC7-1T

AAAGTACCTATATGGAAGATCTATTTTGTGTGTCAAGAGGTGTGTAACCTAAATCTAAGTGATCCGCTCTCTTGGAGACCCATCTTGTTGTTGGAGAAGAAAAAAAAAAAAAAAAAAAGACATCGAACCTTTACCCTATAGTAACGGTTCCAAACAGCCCTTGTATTACTGTATCAGGACTAGGACAAGCAGCACGTGCTGCTGCTGAAGGCACGTAGAGGCGCGATCATCTGAAGGCGAAGCGGAAAGCGACACCCTTCTGTAACGGCACGGCAGCTGACTCCGTCTGCACGGCAGGAGGAAAGAGCACGCACGGCATACACCCTTCTGTATGCTCCTAGCAGGTTCGGTTCGTTGACCTCCTTTCTATCTGCATGAATGGCTTGTGTCTGATGGTGATGGACACGTTTGCATGCTGCGAAGGACGGCACTACGTGTCTACGTGATCTATTTACTACTCAGTGCCTTATTATTGACTCTGTTTGTACATGATGTTTCGTTTTGTTTTTTGCGAATACAAGCTGTACGTACATGTGAAATTTACCTCGTCGCTGCACCGTACAAACATACGGAGTATCCTACTCCGAGCTCCTGCAAGGACGCCGTCACTGGGTCAAGCGTCGCCCTCGATGTCGCCTTGTTTGACTCGTTTCTCGCACCTGCCTTGTGCTATCAGTTTGAAGAACTGAGCCAAACCCGTTCAAACCAAATACGACGAAGAATGGCCTATTTCTAAGGTGAAACTTCCAATTTACCGAAATTCATACCAAAAAAGTATTCTATCTGTCATAAAATAACTATTATTTTGCATGTAATCAATCTTTTCTAAGTTTGATAAAAAATACGTAAATATTAATATTTCTAATATGTAATAAGTACTATTAGATTAATAGTGAAATATATTTTATAATAAATATATTAAAGTACAGATGTTAATAATATTTTTTCTATAATAAGTTTAAATTTCTTTTGAATCTTTAAAAATGAGTTGTCAAATTATTTGGAACCGGAGGACAGAATATATTTTTGAGAATTAAGGATAGAATACACAGATAGATAGTAATAATTACCAAAGGAATCTTGTAAGGGCCTGTTTGCATCCCTTAGCACTAAAAATTAGTACTAATGGATTTAAACCAACCAGTAATAATCTGCTAATTATTAGCTGGGGTTGCTAACTATTAGTTTTATTTAGTAGGTTTGTACCTTCAACTCAATAGCCAATCACCTATTAGTAGCTACTCCAAACACTCCAACAAAAAATTACTAGCTAGCTTAAAAAATAGCAGCTATTAGCTAGCTAATTTTTAGCGCTAATGAATCCCCCTAAGCCCTCTAAGTCAACTCCGTTCTAGATGAACGCTCGTCTCCCTCCCGGCGACTACTACCGCCTACCGGCTATGGCCGTGTCCCCAGAGGAAAACTCCATTTACCGTCCGCGCTTAGCACTAACCACAGTGAAAACTGCACCACTAACCCCAGGTTTTAACCCCAATCCCGTCAATTAGCGGCGACTAACAAAGTAATTAAGCCGGTGGCGAAGCGTCCGCGTCCGCACCAATCCGGTCCGGGAGTTTCCGCCCACCCACGCGCGCCGGCCCCGCTCCTTCCTCCAACTCCTCGCCGCCGGCTCTGCTCCACTCCGCCGACTCGCTTCGCTGTCCACCTCGCCCCTGCTTCCTCCTGATCCCGGATTCGCCTCGCCTCGCCTCGCCGGCGGCCCGGTACTCGTCCCCGCCACGAGCGCCGTCAAGCGAAGCCGTTCTTCGCCTGACCGCGCGCCGCTGCCGCATCTGCCAGGCTCCTTCCGCCGGAGCACGTCGCGGCCAGTTGCTCTCTCGCGCTCCGTCCCCTCGCTCGTCCTCCTCCCGGAAGTGCAGATCCGCACGGGGCTCTCGGATGTATTTCTCCTCTTTGCTGGTAAGGAAGCTCGCTCCGAGGGTTAGTTCCGCTCTGGTCTTCCGTTTCTACGCTGCCAGCAATTAAGTTCCAAGTCGTCGA

>SsCNGC7-2B

AAAAATAGATATTGAACTTTTACCTATAGTAACGGTTCCAAACAGCCCTTGTATTACTGTATCAGGACTAGGACAAGCAGCACGTACTACTGCTGAAGGCACGTAGAGGCGCGATGATCTGAAGGCGAAGCGGAAAGCGACGCCCTTCTGTAACGGCACGGCAGCTGACTCCGTCTCCACGGTAGGAGGAAAGAGCACGCACGGCATACACCCTTCTGTATGCTACTAGCAGGTTCGGTTCGTTGACCTCCTTTCTATCTGCATGAATGGCTTGTGTCTGATGGTGATGGACACGTTTGCATATTGTGAAGGACGGCACTACGTGTCTACGTGATCTATTTACTACTCAGTGCCTTATTATTGACTCTGTTTGTACATGATGTTTCGTTTTGTTTTTTGCGAATACAAGCTGTACGTACATGTGAAATTTACCTCGTCGCTGCACCGTACAAACATACGGAGTATCCTACTCCGAGCTCCTGCAAGGACGCCGTCACTGGGTCAAGCGTCGCCCTCGATGTCGCCTTGTTTGACTCGTTTCTCGCACCCCTTGTGGCTGCCTTGTGCTATCAGTTTGAAGAACTGAGCCAAACCCGTTCAAACCAAATACGACGAAGAATGGCCTATTTCTAAGGTGAAACTTCCAATTTTCCGAAATTCATACCAAAAAAGTATTCTATCTGTCATAAAATAACTATTATTTTGCATGTAATCAATCTTTTCTGAGTTTGATAAAAAATACGTAAATATTAATATTTCTAATATGTAATAAGTACTATTAGATTAATAGTGAAATATATTTTATAATAAATATATTAAAGTACAGATGTTAATAATATTTTTTCTATAATAAGTCTAAATTTCTTTTGAATCTTTAAAAATGAGTTGTCAAATTATTTGGAACCGGAGGACATAATATATTTTTGAGAACTAAGGATAGAATATACAGATAGATAGTAATAATTACCAAAGGAATCTTGTAAGGGCCTGTTTGCATCCCTTAGCACTAAAAATTAGTACTAATGGATCTAAACCGACCAGTAATAACCTGCTAATTATTAGTTGGGGTTGCTAACTATTAGTTTTATTTAGTAGGTTTGTACCTTCAACTCACTAGCCAATCACCTATTAGTAGCTACTCCAAACACTCCAACAAAAAATTACTAGCTAGCTTAAAAAATAGCAGCTATTAGCTAGCTAATTTTTAGCGCTAATGGATCCCCTAAGCCCTGTAAGTCAACTCCGTTCTAGATGAACGCTCGTCTCCCTCCCGGCGACTACTTGCCATTGCCACTAAACTTTCATCAGGGTCTGCTAAAAAAAAACTTTCATCAGTGAAAAACTCCATTTCCCGGCGATTACTACCGCCTACCGGCTATGGCCGTGTCCCCAGAGGAAAACTCCATTTACCGTCCGCGCTTAGCACTAACCACAGTGAAAACTGCACCACTAACCCCAGGTTTTAACCCCAATCCCGTCAATTAGCGGCGACTAACAAAGTAATTAAGCCGGTGGCGAAGCGTCCGCATCCGCACCAATCCGGTCCGGGAGTTTCCGCTCCCACGCCCCACCCTTCCTCGAACTCCTCGCCGCCGGCTCTGCTCCACTCCGCCGACTCGCTTCGCTGTCCACCTCGCCCCTGCTTCCTCCTGATCCCGGATTCGCCTCGCCTCGCCTCGCCGGCGACCCGGTACTCGTCCCCGCCACGAGCGCCGTCAAGCGAAGCCGCTCTTCGCCTGGCCGCGCGCCGCTGCCGCATCTGCCAGGCTCCTTCCGCCGGAGCACGTCGCGGCCAGTTGCTCTCTCGCGCTCCGTCCCCTCGCTCGTCCTCCTCCACCGTCGCGGCGGCGGCGGCCTTCGTCCTCCTCCCGGAAGTGCAGATCCGCACGGGGCTCTCGGATGTATTTCTCCTCTTTGCTGGTAAGGAAGCTCGCTCCGAGGGTTAGTTCCGCTCTGGTCTTCCGTTTCTACGCTGCCAGCAATTAAGTTCCAAGTCGTCGA

>SsCNGC7-3D

TAACTATATGGAAGACTTATTTTAGGTGTCAAGAGAGGTGTAACCTAAATCTAAGTGATCCGCTCTCTTGGAGACCCATCTTGTTGTTGGAGAAAAAAAAAATAGATATCGAACCTTTACCCTATAGTAACGGTTCCAAACAGCCCTTGTATTACTGTATCAGGACTAGGACAAGCAGCACGTACTACTGCTGAAGGCACGTAGAGGCGCGATGATCTGAAGGCGAAGCGGAAAGCGACACCCTTCTGTAACGGCACGGCAGCTGACTCCGTCTCCACGGCAGGAGGAAAGAGCACGCACGGCATACACCCTTCTGTATGCTACTAGCAGGTTCGGTTCGTTGACCTCCTTTCTATCTGCATGAATGGCTTGTGTCTGATGGTGATGGACACGTTTGCATGCTGCGAAGGACGGCACTACGTGTCTACGTGATCTATTTACTACTCAGTGCGTTATTATTGACTCTGTTTGTACATGATGTTTCGTTTTGTTTTTTGCGAATACAAGCTGTACGTACATGTGAAATTTACCTCGTCGCTGCACCGTACAAACATACGGAGTATCCTACTCCGAGCTCCTGCAAGGACGCCGTCACTGGGTCAAGCGTCGCCCTCGATGTCGCCTTGTTTGACTCGTTTCTCGCACTTGTGGCTGCCTTGTGCTATCAGTTTGAAGAACTGAGCCAAACCCGTTCAAACCAAATACGACGAAGAATGGCCTATTTCTAAGGTGAAACTTCCAATTTACCGAAATTCATACCAAAAAAGTATTCTATCTGTCATAAAATAACTATTATTTTGCATGTAATCAATCTTTTCTGAGTTTGATAAAAAATACGTAAATATTAATATTTCTAATATGTAATAAGTACTATTAGATTAATAGTGAAATATATTTTATAATAAATATATTAAAGTACAGATGTTAATAATATTTTTTCTATAATAAGTTTAAATTTCTTTTGAATCTTTAAAAATGAGTTGTCAAATTATTTGGAACCGGAGGACAGAATATATTTTTGAGAACTAAGGATAGAATATACAGATAGATAGTAATAATTACCAAAAGAATCTTGTAAGGGTCTGTTTGCATCCCTTAGCACTAAAAATGAGTACTAATGGATCTAAACCGACCAGTAATAACCTGCTAATTATTAGCTGGGGTTGCTAACTATTAGTTTTATTTAGTAGATTTGTACCTTCAACTCACTGGCCAATCACCTATTAGTAGCTACTCCAAACACTCAAACAAAAATTACTAGCTCGCTAAAAAATAGCAGCTATTAGCTAGCTAATTTTTAGCGCTAATGGATCCCCCTAAGCCCTCTAAGTCAACTCCGTTCTAGATGAACGCTCGTCTCCCTCCCGGCGACTACTACCGCCTACCGGCTATTGCCGTGTCCCCAGAGGAAAACTCCATTTACCGTCCGCGCTTAGCACTAACCACAGTGAAAACTGCACCACGGTTTTAACCCCAATCCCGTCAATTAGCGGCGACTAACAAAGTAATTAAGCCGGTGGCGAAGCGTCCGCATCCGCACCAATCCGGTCCGGGAGTTTCCGCTCCCACGCCCCACCCTTCCTCGAACTCCTCGCCGCCGGCTCTGCTCCACTCCGCCGACTCGCTTCGCTGTCCACCTCGCCCCTGCTTCCTCCTGATCCCGGATTCGCCTCGCGTCGCCGGCGGCCCGGTACTCGTCCCCGCCACGAGCGCCGTCAAGCGAAACTGTTCTTCGCCTGGCCGCGCGCCGCTGCCGCATCTGCTAGGCTCCTTCCGCCGGAGCACGTCGCGGCCAGTTGCTCTCTCGCGCTCCGTCCCCTCGCTCGTCCTCCTCCACCGTCGCGGCGGCGGCGGCCTTCGTCCTCCTCCCGGAAGTGCAGATCCGCACGGGGCTCTCGGATGTATTTCTCCTCTTTGCTGGTAAGGAAGCTCGCTCCGAGGGTTAGTTCCGCTCTGGTCTTCCGTTTCTACGCTGCCAGCAATTAAGTTCCAAGTCGTCGA

>SsCNGC8

CTATTAAGCCTAATTAGTCCAAAATTGAAAGCTTTTTGTCAAATAAGACAAAAGTGCTACAGTGTTGGTTAAACTTTAACATCTCTAACCAAACAGGCCCTAAAAACTACTTTTAACTGTGAATCCAATGATACTAAATCAAAAAAAATTATGGTACAACCTACAAATTCTTGGTGCAAGTGTCAGCCAAAGATTAACAAGCTTGAATCTTGGATTGTGTGTGCCATAGAGAAAGTATTTTTTTGGTCAATCCACTCCATAGACTATTCAAATCCTTAGTTGCAATCAATTTTAAGACAACCTAGCAAGCAATCACGTACACATTGCAACATATCTTACAACTATAGGATCACTTGAAGCTGGTGTCTCTGTAGAATTTAATTAGACTTCGGCCCAACCTCATCTCAACTAAATCAAATAAATTCTAAGAAAGATATTAAGCACAATAAAAAGATTAATCATGCATTGAAAGCTAGAGATTGACTTATTTAATATAATGAGTGTTTTTTGTGCGCACCTGTGTTGAGAAAGGTGAAAAGTGAACCACACGTATGTACATGTTAAAGCGTGCCGAGTCACGACACTCGGGCTTAGGGGGTGTTTGATTCCCACAACTAAAGTTTAGTCCATATTACATCGGATGTTTGAATACATGCATAGAGTATTCAATATAGATCTAAAAAATAACTAATTATACAAATTGTGACTAATTTGCGAGATGAATTTTTTTAAGCCTAATTAGTCCATGATTTGACACTATAGTGCTACAATAAACATGTGCTAATTATGGATTACTTAGGCTTAATAAATTCATCTCGCGGTTTACTGACGGAATCTGTAATTTGTTTTTTTATTAGTATCCGAACACCTCATACGACACCTCATATAACATACGATGTAACACCAACTAAATTTTAGTCCCTACATCCAAACGCCCCCTTATGCTCATGTCAGCTAACTCATGACATGTCACATGGGTGTCACAATGCTAGCAAAAGTTTTATAGCTCGAAATTGTAACCCACGCTTATTGAAGGATGATATAGGGTGTGTTTAGTTCCTCATTTCACCAAAAAAAATTTAGGTTTTGGTCCATCATTTTGTAAAATGGAACTAAACACCATGCAAAATTTTTAGTAAAAAGATATTCATTCTCCATTTTGGATTTTGATCTCAAGAGGCAAAAAAAAAAAAGCGAAAAAGCCAAAAAGCTCTCACCCACCTCATGAAAAATTTGACATTTTGAAGTAGAACTAAACACACCTATAAAAATTTCGGCATTTTACATTTCATCATTTTAAAGTAGTTGACATTTTACATTTTGCATTTCATGAGTAAATAAACACAGCCATAGTCCAACATCGTTGGACGCCTGGGCCTTGGACCAAATTCATGCCATAGGTTTATGTTTGTCATCCATAAAAGCGACGGACAACTAAGCGAAGAACAAAAGAAGAGGAATAAATTATCTTACTTAAATATTAAGCATATAATGTGCATAATCGTGCTCGTGCCTCTCACGGTAAAAAAACAGTTTTAGATAATTACGAGATCATCAATATACTTATTATTTTAAAAGGTAGGTAACCACAAAGTACCGAAGTAGCAGTATATTGCCGTAATTTCCATGCTTGTAGGCTGTTGATTAAACTGTGCTTCGCTGTCAGAATCTGATGCTTACACCTGCTCGGCAGTAAGTCAGCAGTGAAATTTCCATATCCCTATTTTTTTTATCCCCAACAACCGTGGACTGTTTCTCCAACGGGTCAAGCGCGACAGGAGTTCTTGGCTCTTGCTTTCCAACTCCATTTCCGGTCGCTTCCCTCCCCACAGTGGCCCTGTTCTCATTTCCTCGAACTCCGAGCGGGCGGCTGCGATTCCCAGCGTCCGAGCACCAAGCCCCCGGCCTTGGGGCTGCTACGGCGCCGGATGAGCGAGCAAGGCTGTTTCTTGGGCGAGCTGCCGGTCCGTGCAGCGGCGGAGTAGTGGACGGCGGGCCCCA

>SsCNGC8-2B

ATCGAGTGGAAGGGGAAAATATATTACCTTCGCGTAGAGGAACCTGCTGCTGCTTTGTATATTACCCCTTCTTTTGTAAAAAGCGTCGTTTGATCCATACGAACTCCAATGCCCGCTTTGTATATTGTTAATATAATATCACGACTATGAATTTGGCATCATATAAAAACTACTTTTAACTATGAATCCAATGATACTAAATCAAAAAAAAATTATGGTACAACCTACAAATTCTTGGTGCAAGTGTCAGCCAAAGATTCACAAGCTTGAATCTTGGATTGTGTGTGTGCCATAGAGAAAGTATTTTTTGGTCAATCCATTCCATAGACCATTCAAATCCTTAGTTGCAATCAATTTTAAGACAACCTAGCAAGCAATCACGTACACATTGCAACATATCCTACAACTATAGGATCACTTGAAGCTGGTGTCTCTGTAGAATTTAATTAGACTTCGGCCCAACCTCATCTCAACTAAATCAAATAAATTCTAAGAAAGATATTAGGCACAATAAAAAGATTAATCATGCATTGAAAGCTAGAGATTGACTTATTTAATATAATGAGTGATTTTTGTGCGCACCTGTATTGAGAAAGGTGAAAAGTGAACCACACGTATGTACACCTTAAGATGTGCCGAGTCACGACACACGGGCTTATGGGGTGTTTGATTCTCACAACTAAAGTTTAGTCCCTATTACATCGGATGTTTGGACACATATATGGAGTATTAAATATAGACCTAAAAATAACTAATTGCACAGATTGCGACTAATTTGCGAGACAAATTTTTTAAACCTAATTAGTCCATGATTTGACACTGTGGTGCTACAGTAACATGTGCTAATGATGGATTACTTAAGCTTAATAAATTTGTCTCACGATTTATTGACGGAATCTGTAATTTGTTTTTTTTATTAGTATCCGAACACCCCATACGACACCTCATATAACATACGATGTAACACCAACTAAACTTTAGTCCCTGCATCAAACAGGCCTTAGGCTCATGTCAGCTAACCCACGATATGTCACATGGGTGCCACAATGCTAGCAAAAGTTTTATACCTCGAAATTGTAACCCGCGCTTATTGAAGGATGATGTAGTCCAACATCATTGGACGCCTGGGCCTTAGACCAAATTCATGCCATAGGTTTATGTTTGCCATCCATAAAAGCGACGGACAACTAAGCGAAGAACAAAAGAAGAGGAATAAATTATCTTACTTAAATATTAAGCATATAATGTGCATAGTCGTGCTCGTGCCTCTCACGGTAAAAAAAAACAGTTTTAGATAATTACGAGATCATCAATATACTTATTATTTTTAAAGGTAGGTAACCACAAATGAGAAAAGTACCGAAGTAGCAGTATATTGCCGTAATTTCCATGTTTGTAGGCTGTTGATTAAGGGCTTTGATTAGAGGTGTTAAAGTTTAACAAACATGGTAGCATTTTCGTTTTATTTGAATTTTTTTCTTCCAATTTTAGACTAATTAGGTTTAATAGAGTCGTTTCGTAAATTATTCCTAATCTATGCTGACATGTATGTAATTAGTCTATATTCAGTAGTCCATACATTTACCGCAACATTGATGTGATAGAAGTTAAACTTTAACCTTCCTGAACTAAACGGGCCTAACTGTGCTTCGCTGTCAGAATCTGATGCTTACACCTGCTCGGCAATAAGTCAGCAGTGAAATTTCCATATCCCTATTTTTTTATCCCCAACAACCGTGGACTGTTTCTCCAACGGGTCAAGCGCGACAGGAGTGCGCGGTCTCCGTCTTTCTTCTTGGCTCTTGCTTTCCAACTCCATTTCCGGTCCCCCCAGTGGCCCTGTTCTCATTTCCTCGAACTCCGAGCGGGCGGCTGCGATTCCCAGCGTCCGAGCACCAAGCCCCCGGCCTTGGGGCTGCTACGGCGCCGGATGAGCGAGCAAGGCTGTTTCTTGGGCGAGCTGCCGGTCAGTGCAGCGGCGGAGTAGTGGACGGCGGGCCC

>SsCNGC8-3C

TGAGTCATCATGGCAGCACCAACAGCTGCACGTGCAACGTTTTGAGGTGCCACGACAGGAATAAACGACCGTGCAAACGAAACCCGTTGGAGACCCAAAAAAAAGGCATGAAACAGTTGTTTTTGAAGAACACAATTTAAAACATCCCATACAATAACACCATTATTATGGTAAATTCAACTGAAAAAGTGATAGAGAATGCGAGGAGCAATCATCGAGTGGAAGGGGAAAATATATTACCTTCGCGTAGAGGAACCTGCTGCTGCTTACCCCTTCTTTTGTAAAAAGCGTCGTTTGATCCATACGAACTCCAATGCCCGCTTTGTATATTGTTAATATAATATCACGACTATGAATTTGGCATCATATAAAAGCTACTTTTAACTATGAATCCAATGATACTAAATCAAAAAAAAATTATGGTAAAAAATAAAAGAAACAAGAAACCAAGGCCACCCATCACCCGTGCCCATGCAACTGTCAGCCAAAGATTCACAAGCTTGAATCTTGGATTGTGTGTGCCATAGAGAAAGTATTTTTTGGTCAATCCACTCCATAGACCATTCAAATCCTTAGTTGCAATCAATTTTAAGACAACCTAGCAAGCAATCACGTACACATTGCAACATATCTTACAACTATAGGATCACTTGAAGCTGGTGTCTCTGTAGAATTTAATTAGACTTCGGCCCAACCTCATCTCAACTAAATCAAATAAATTCTAAGAAAGATATTAGGCACAATAAAAAGATTAATCATGCATTGAAAGCTAGAGATTGACTTATTTAATATAATGAGTGTTTTTTGTGCGCACCTGTGTTGAGAAATGTGAAAAGTGAACCACACGTATGTACACCTTAAGGCGTGCCGAGTCACGACACACAGGCTTAGGGGGTGTTTGATTCTCACAACTAAAGTTTAGTTCCTATTACATCGGATGTTTGGACACATATATGGAGTATTAAATATAGACCTAAAAATAACTAATTGCACAGATTGCGACTAATTTGCGAGATAAATTTTTAAACCTAATTAGTCCATGATTTGACATTGTGGTGCTACAGTAAACATGTGCTAATGATGGATTACTTAGGCTTAATAAATTCATCTAGCGGTTTACTGACGGAATCTGTAATTTGTTTTTTTATTAAGTATCCAAACACCCCATGCGACACCTCATATAACATACGATGTAACATCAACTAAATTTTAGTCCCTGCATCCAAACGTCCCCTTATGCTCATGTCAGCTAACCCATGACATGTCACATGGGTGTCACAATGCTAGCAAAAGTTTTATAGCTCGAAATTGTAACCCGCGCTTATTGAAGGATGATATAGTCCAACATCATTGGACGCCTGGGCCTTGGACCAAATTCATGCCATAGGTTTATGTTTGTCATCCATAAAAGCGACAGACAACTAAGCGAAGAACAAAAGAAGAGGAATAAATTATCTTACTTAAATATTAAGCATATAATGTGCATAGTCGTGCTCGTGCCTCTCACGGTAAAAAAAAAACAGTTTTAGATAATTACGAGATCATCAATATACTTATTATTTTAAAAGGTAGGTAACCACAAATGAGAAAAGTACCGAAGTAGCAGTATATTGCCGTAATTTCCATGCTTGTAGGCTGTTGATTAAACTGTGCTTCGCTGTCAGAATCTGATGCTTACACCTGCTCGGCAGTAAGTCAGCAGTGAAATTTCCATATCCCTATTTTTTTTATCCCCAACAACCGTGGACTGTTTCTCCAACGGGTCAAGCGCGACAGGAGTTCTTGGCTCTTGCTTTCCAACTCCATTTCCGGTCGCTTCCCTCCCCACAGTGGCCCTGTTCTCATTTCCTCGAACTCCGAGCGGGCGGCTGCGATTCCCAGCGTCCGAGCACCAAGCCCCCGGCCTTGGGGCTGCTACGGCGCCGGATGAGCGAGCAAGGCTGTTTCTTGGGCGAGCTGCCGGTCCGTGCAGCGGCGGAGTAGTGGACGGCGGGCCCCATG

>SsCNGC8-4D

CCCTGTTCGTTTGGACTTATCAGCAGAATCAGCAGCAATCCAACAGTGTTTTTCTCTCACAACAAATCAGCGAACAGTACTTTCAGCACAATAAACCAGCGAAACGAATAGGGTGTAAGGCCCTGTTTGGTTTCTGAATCATTCAGTTTAACTCCCATCACATCAATGTTGCGGCAAATACATGGAGTACTAAATGTAGACTAATTGCATGTATGTCGACACAGATTAGGAGTAATTTACGAGACGAATCTATTAAGTCTAATTAGTTCAAAATTAGAAACTTTTTGTCAAATAAGACAAAAGTGCTACAATGTTTGTTAAACTTTAACACCTCTAACCAAATAGGCCCTAAAAACTACTTTTAACTATGAATCCAATGATACTAAATAAAAAAAATTATGGTACAACCTACTTCCTCCGTCCATAAAAACATGCAACTATGGGTTGCGTGCTAGAAAAAGTAGTTCATGTTTGACCAAGTTTATAGTGAATATTATTTATATTTATGGCTCCAAATCAATTTATTATGAAAATATATTTCATAATTAATCTAATGATATTTATTTGATATCATAAACATTGATACTTTTTTTATCTATAATTAGTCAAAGTTAAGATACTAAAACATGACACTGTGCTAGAATTGCATGTTTTCCTAGACGGAGGGAGTACAAGTGCAAGTGTCAGCCAAAGATTAACAAGCTTGAATCTTGGATTGTGTGTGCCATAGAGAAAGTATTTTTTGGTCAATCCACTCCATAGACTATTCAAATCCTTAGTTGCAATCAATTTTAAGACAACCTAGCAAGCAATCACGTACACATTGCAACATATCTTACAACTATAGGATCACTTGAAGCTGGTGTCTCTGTAGAATTTAATTAGACTTCGGCCCAACCTCATCTCAACTAAATCAAATAAATTCTAAGAAAGATATTAAGCACAATAAAAAGATTAATCATGCATTGAAAGCTAGAGATTGACTTATTTAATATAATGAGTGTTTTTTGTGCGCACCTGTGTTGAGAAAGGTGAAAAGTGAACCACACGTATGTACACGTTAAAGCGTGCCGAGTCATGACACACGGGCTTAGGGGGTGTTTGATTGATTCCCGCAACTAAAGTTTAATCCCTATTACATCGGATGTTTGAACACATGCATATAGTATTTAATATAGATCTAAAAAATAAATAATTGTACAAATTACGACTAATTTGCGAGATGAATTTTTTAAGCCTAATTAGTCCATGATTTGACACTATGGTGCTACAGTAAACATGTGCTAATTATGGATTACTTAGCTTAATAAATTCGTCTCGCGGTTTACTGACGGAATCTGTAATTTATTTTTTTATTAGTATCCAAATACCCCATACGACACCCCATATAACATACGATGTAACACAAACTAAATTTTAGTCCCTGCATCCAAACGCCTCTTATGCTCATGTCAGCTAACCCATGACATGTCAGCATATAATGTGCATAGTCGTGCTCGCGCCTCTCACGGTAAAAAAAACAGTTTTAGATAATTACGAGATCATCAATATATACTTATTATTTTAAAAGGTAGGTAACCACAAATGAGAAAAGTACCGAAGTAGCAGTATATTGCCGTAATTTCCATGCTTGTAGGCTGTTGATTAAACTGTGCTTCGCTGTCAGAATCTGATGCTTACACCTGCTCGGCAGTAAGTCAGCAGTGAAATTTCCATATCCCTATTTTTTTTATCCCCAACAACCGTGGACTGTTTCTCCAACGGGTCAAGCGCGACAGGAGTTCTTGGCTCTTGCTTTCCAACTCCATTTCCGGTCGCTTCCCTCCCCCCAGTGGCCCTGTTCTCATTTCCTCGAACTCCGAGCGGGGGGCTGCGATTCCCAGCGTCCGAGCACCAAGCCCCCGGCCTTGGGGCTGCTACGGCGCCGGATGAGCGAGCAAGGCTGTTTCTTGGGCGAGCTGCCGGTCAGTGCAGCGGCGGAGTAGTGGACGGCGGGCCCCA

>SsCNGC9

GCAGTTGATTCTATCCTCGGCGTACATGGCATCTCACTTTCCCTGTGCAGCAAGTGTACTGAGTCTGATTGATGCAACTTCGTGAGTGGAGAAATGGTTAGCTGTAAACTTACCGATGCAATAATGGAAGCGACGATTTGACTGTTTCGCCCATTGCGGGATTATAGAAGCGACTTTTGAATCAGTGACGACTTCCTAGTCTGAAAGTTTCCGCCCATGCCCATGCAGCAGTCGCCAATGCTGTGGTAAAATGTACTCCCCTTGAGGCCTCGATGACTAGGTATTGTAATTAGTTTCTGCATAGTTTGACTATGCGGATCCAATTCAAAGGTCGGCTAATTGAACAAGAAAAGTACTCGCTCCGATCGTTTATACACGATGAGGACGTAGTGAATTTTTAGAATGGTTATTAAGGGGTTATTTAGTTTAGCTATGAGCTATGAAAAAATTGTAAGAGCATTTCCAATAGTTTCCAAAACCCAATCCTAATTTTTTGGCAAGATTGAAAAAAAACTAGTCTCCAATAACTCCCAATCCCTGCACTTGGGAAATTTGATCCAATTGCGCGCAAATAATACGCCCGTATTCATTGTCCAATCTAGCGGTGGAAGGACACATGGAAAAAATAAAGAGTAGAACAGGGTCCTCAATGCAAATGTACGAGATAGAAAAAAAGTATAAAAGTAGTTGGATGATTTCGAAATAGTCGGAGACTCCTGATGCAAAATTGCATTTTACTCCTTCCATTCTAAATTATAAGTCGTTTTGACTTTTTTAGTTCATCCATTTTGCTATGTATCTAGACATATTATTATATCTAGATGCATAGCAAAATGAATATAATAAAAAAAGTCAAAGCGACTTGTAATTTGGAACGGAGGGAGTATATTTTAGAGCAAGATTTTGAATGCTCCTAACTTTTGCATAGCCACTTGAAAAAATCATTGATTTACCAATTAATTTTTAAGAACGGTAGGAGATGCTCTAAGTTATTTGGTTAAAATAACTATGGAACTTGCCTTTTCTCTTTATGTCTCTTAAAACAGCTATAAAACGTCTAAAAGCAACATCCAAAGCGTTTCTAAAATTATACTGTGTGAAAGAATATGTTTTCGAAAGAACAATTTCTAGTTCTATTTAGTAAAAAAAATTAATTCTGTTCGTTTAGTTGGTATTATGCTTTTTGACAGCAGAAACATTTCAGAAGGTAAACCAAACGTAGCTTAAGAAAATTTATAGAATGTCTTTTGCGTCTTTGCCCCAACGGGCATGTACTAGCAGAGCAGCAGTCTCCGACCACGGTGAGGAACAAACATGGCAGAGCTTAGGATGTATAGAACGCAGGAATTTTAATGGAATCCTACAGAATTTCACAGAGATCGGATCAGTTTAGATAAACATGGGCCTTATCCATCCGACTTATAATTCGTACTTTTCAACTTGTTTTTCAGGTAGAACAGTATTTTTCAGCCAATTTATCAGCACAGCCGAATGAAAACAAAGAAATGTAACATTTTTCCCATTTGACTGTTTCAAACATGCCATTAGACCTTGTTTAAATGTGTATATATTTATCTCAATCCACATGCATTAAAATAAATTAAAGTAAAATTAAACTAAATTCCACGCCATTTCGGTCCGACGCATCCCTGTAGTGTAATAACCGGCCACTGAGCATCTGCACCCACGTGTCATTGCCGATGTATATGAGAACAGGCAGTCACCACTTGCCTTGGTCCACCTCTTCCCTGTCCCTCTGGCTAAAAAGAACCGCATAAAGATCTTTTTTTTCTTGTCTCCGTTCTTCCACTCGAGTTCTTCAGTTCTTTGGAATCATCAGACGGAGCAACTCGAGTTTGCCATGGTGGTGATCTGGAACCGGAAGAGGTGCGCTCGCCCTCTCTGATCCCCTGATTGGCCTCGCGCCCTCGCTCCAGTTGGGTGCGAGATTTGGACGGGACGGGCGGAGGTTGGGAGAGCCGAGGCGGCCGGCATGTTTGG

>SsCNGC9-1T

AAATTGTAAGAGCATTTCCAATAGTTTCCAAAACCCAATCCTGATTTTTGGCAAGATTGAAAAAAAACTAGTCTCCAATAACTCCCAATCCCCGCACTTGGGAAATTTGATCCAATTGCGCGTAAATAATACGCCCGTATTCATTGTCCAATCTAGCGGTGGAAGGACACATGGAAAAAATAAAGAGTAGAACAGGGTTCTCAATGCAAATGTACGATATAGAAAAAAGTATAAAAGTAGTTGGATGATTTCGAAATAGTCGGAGACTCCTGATGCAAAATTGCATTTTACTCATTCCATTCCAAATTATAAGTCGTTTTGACTTTTTTTAGTTCATCCATTTTGCTATGTATCTAGACATATTATTATATATAGATGCATAGCAAAATGGATATAATAAAAAAAAGTCAAAGCGACTTGTAATTTGAAACGGATGGAGTATATTTTAGAGCAAGATTTTGAATGCTCCTAACTTTTGCATAGCCACTTGAAAAAAATCATTGATTTACCATATAATTTTTAAGAACGGTAGGAGATGCTCTAAGTTATTTGGTTAAAATAACTATGGAACTTACCTTTTCTCTTTATCTCTTTTAAAACAGCTATAAAACGTATCTATTTCGATCCATTTGGAAAAGAGGAAAGCTGAAAGCTAAAAGCAACATCCAAAGCGTTTAAAAAATTATACTGTGTGAAAGAATATGTTTTCGAAAGAACAATTTCTAGTTCTATTTAGTAAAAAAAAATTAATTCTGTTCGTTTAGTTGGTATTATGCTTTTTGACAGCAGAAACATTTCAGAAGGTAAACCAAACGTAGCTTAAGAAAATTTATAGAATGTCTTTTGCGTCTTTGCCCCAACGGGCATGTACTAGCAGAGCAGCAGTCTCCGACCACGGTGAGGAACAAACATGGCAGAGCTTAGGATGTATAGAACGCAGGAATTTTAATGGAATCCTACAGAATTTCACAGAGATCGGATCAGTTTAGATAAACATGGGCCTTATCCATCCGACTTATAATTCGTACTTTTTAGCTTATTTTTCAGGTAGAACAGTGTTTTTCAGCCGATTTATCACCACAGCCGAATGGGAACACAGAAACGTAACATTTTTCCCATTTGACTGTTTCAAACATACCCTTAAGCCTTGTTTAAATGTATATATATTTATCTCAATCCATGTGCATTAAAGTGAATTAGAGTAGAATTAAACTAAATTCCACTTCATTTCGGTCCGACGCATCCCTGTAGTGTAATAACCGGCCACTAAGGCTATTCGCACTCATAGACCTCTATATTCATCCTCTATACGTCCCAGTCAGCGAGATTCTCTTCTCTATACTCAATTCTCTCGCACTCATCCATCCTCTATATCATTCTCTATCCAATCTATCCACTATGGGACCCACTTGGCACTTTATCTAAATATATCCATGTGTGATCTAGTTTTAAAGGATTTATTGAGACGGATTTAATGGTGCAATCGAAATTTAATTTGGATTTTTGGTTTGTGAGTTATAGCATTTTTTTCTTTGTTTAGCTAGTGGGGTCCGCCACTGCACAAGTAAGCACCGTACTACTCTAGATATAGAGTGAGAAAAAAAATAGAATATTGTTTTAGAGTACCCAGTGCAGCAGTTTTTACTCTATATTCATATTTAGAATATCCTACTGCGGGTACTCTAAGCCTATACACCCATGTGTCATTGCCGATGTATATGAGAACAGGCAGTCACCACTTGGTCCACCTCTTCCCTGTCCCTCTGGCTAAAAAGAACCGCATATAGATCTTTTTTTTCCTGTCTCCGTTCTTCCACTCGAGTTCTTCAGTTCTTTGGAATCATCAGACGGAGCAACTCGAGTTTGCCATGGTGGTGATCTGGAACCGGAAGAGGTGCGCTCGCCCTCTCTGATCCCCTGATTGGCCTCGCGCCCTCGCTCCAGTTGGGTGCGAGATTTGGACGGGACGGGCGGAGGTTGGGAGAGCCGAGGCGGCCGGCA

>SsCNGC9-2D

TGGCTCTGATATTCTGACACAATGCCAATCTACTTAAGAGCCTCTAGATGTCGCAATTGAGTCTAGTATTGGTCCTTTTCAAATTTAGCAAACACCAATGCACACAGGGAATCCCAATCGGTAACCCGGCCATGCTTCTGGACTGTCTGCAACTAGATTGTCGCGGCCCCAGTGAAATTGAGGGCCGCGAAGTGCGTCTTCAGAGACTCATGAACTGCATAGAGTTCAAAGTACATGACACAATGATCTTTCCATAGGCGCGGGTTATCACCATCAAACTTCGGGAACTCGATCTTGGGAAATGTTGACGGTCCTTAAGTATCAATTATAATTATCAAATAAATAGAGAAAAGGATCCAAATGAAACCAACACCTAGACTTAGGGTTTTATCTGACAGAATTCCATGAGTTTTGCTGTTTATCTATTTCTGCAGGGGGTTATTAGGAAATACGGAAGAAAGGCCCACATGTCGGATTAATGACGGGATATTAACCGAACACGTAATTATCTTACATCTAGAAGAGTCCAGAAGCCACGAGAACGAACGGGAGGCGTAACAGAGCCGGGGACAGGGCGCCCGCCCTAGTCCCTAGGGCGCCCGCCCTGCTGGCTCAGCCAATCAGGCTCCGCCTCGAGGATTATGCTCCACCGACCTAGAGGATCAAGGATAACTANNNNNNNNNNNNNNNNNNNNNNNNNNNNNNNNNNNNNNNNNNNNNNNNNNNNNNNNNNNNNNNNNNNNNNNNNNNNNNNNNNNNNNNNNNNNNNNNNNNNACAGAATTTCACAGAGATCGGATCAGTTTAGATAAACATGGGCCTTATCCATCCGACTTATAATTCGTACTTTTTAGCTTATTTTTCAGGTAGAACAGTGTTTTTCAGCCGATTTATCACCACAGCCGAATGGGAACACAGAAACGTAACATTTTTCCCATTTGACTGTTTCAAACATACCCTTAAGCCTTGTTTAAATGTATATATATTTATCTCAATCCATGTGCATTAAAGTGAATTAGAGTAGAATTAAACTAAATTCCACTTCATTTCGGTCCGACGCATCCCTGTAGTGTAATAACCGGCCACTAAGGCTATTCGCACTCATAGACCTCTATATTCATCCTCTATACGTCCCAGTCAGCGAGATTCTCTTCTCTATACTCAATTCTCTCGCACTCATCCATCCTCTATATCATTCTCTATCCAATCTATCCACTATGGGACCCACTTGGCACTTTATCTAAATATATCCATGTGTGATCTAGTTTTAAAGGATTTATTGAGACGGATTTAATGGTGCAATCGAAATTTAATTTGGATTTTTGGTTTGTGAGTTATAGCATTTTTTTCTTTGTTTAGCTAGTGGGGTCCGCCACTGCACAAGTAAGCACCGTACTACTCTAGATATAGAGTGAGAAAAAAAATAGAATATTGTTTTAGAGTACCCAGTGCAGCAGTTTTTACTCTATATTCATATTTAGAATATCCTACTGCGGGTACTCTAAGCCTATACACCCATGTGTCATTGCCGATGTATATGAGAACAGGCAGTCACCACTTGGTCCACCTCTTCCCTGTCCCTCTGGCTAAAAAAACTGATTATGCATTCTAAAGTTAAAGTAAAAACTAAATTCCACGCCATTTCGGTCCGACGCATCCCTGTAGTGTAATAACCGGCCACTGAGCATCTGCACCACGTGTCATTGCCGATGTATATGAGAACAGGCAGTCACCACTTGCCTTGGTCCACCTCTTCCCTGTCCCTCTGGCTAAAAAGAACCGCATAAAGATCTTTTTTTTCTTGTCTCCGTTCTTCCACTCGAGTTCTTCAGTTCTTTGGAATCATCAGACGGAGCAACTCGAGTTTGCCATGGTGGTGATCTGGAACCGGAAGAGGTGCGCTCGCCCTCTCTGATCCCCTGATTGGCCTCGCGCCCTCGCTCCAGTTGGGTGCGAGATTTGGACGGGACGGGCGGAGGTTGGGAGAGCCGAGGCGGCCGGCA

>SsCNGC10

CGGAGCGGTAGCGGAGACTAATGCGGCTTCATCCTCTTCAGCAACCGGGGCGCTGACACTGGGCTCCACATCCAGATGCGGCGCAAGTACCGGGCGTCATTCACAGGTGCCATGCGATCGCGTTTGGGTCCACGTGGCAACCATCAAGCAGGGGGCACATGGGTGCTCCCGTCGTCCACCTATTTTTTCCCCATTTGTATTATTATTATTATCATCTGAACTTCAACCAAAACGTTGCGCCCCCGGAATGGCAGTCATGAGTGAAGGAGATCTTCAGATTTATTGGGTACTACAATTAGGGGTACTTAAATTGTGATGGAAAATCACCCGCCCCCTATTTAAGGGAATAGATCAACAAAGAAGAAACGGGCTAAACTCCCCCTTGCTGGGCCTCCAGCAAGACACGTGAGCAAGCCAGCCCATATCCCCACTTCATCTGAGGCCGCACTCAGAGGAGTCTCGGAACACCTCCGGTTCGCCCGAGGCGTCCGCTCTGAGGTCTCGGACACAGGCTGACCTCCGGCAACATCACCGCCTCGCCCGAGCCCCAGAGACGGCATTGTCCGTCTCCGACTCGCCCGAGATCGGCCTCTAATGCCGCTCCCCCATTGGCACGCTCGAGACCCCTGTGACATCACCCACGTTCTCGCATCTGCCCAACCGGACAGGCGGGAATGACCGGGGTCAAATCACCACGCCTTCGACTGTAATAGGCATGGGAAAGAGCTGCTGACCACTCTGCCACCCATACTGGCACTATATGCACGATTCGTGTGTGCACAGTGTACGGAGCAACTGCTCTGGGCCACTATTCCACGATCGGATAAGATCACACCACATTTCCTGCCTCCGAGGCCTCGGCCCTCTACGCTGAGTCAGACTACAAGGACAAATACGCCACACCGGCTATAGACCAGGTATGACAAAGCACGTCGCTACAATGCCAAAAGACAGAGACCACTCCTTCAGCAGTGGCACACGTACCAGAACACGTAGTAGCAGTACCAGAAGTATTGACGCCATGCCTCCACAGTACAGACCACGCCGCCACCGGATAAGGCCCTTCCGAGACCTCCAGGCTCCCCCACCGGCAAGGTCGCACCATATGCCAATAATCATGTAACCCTGCACCTCCTTGAGAATATAAAAGGAGCATGCAGGATCATTCCAAAGAAAAAAAGAAAAAAGAGAGAACTCAGTCGAGCACAACTCAACTGAACCACCATAGCAACATAACGCGAGATACGAGTAGGCTACTCTCTTGGACCTTGCGAGGTAACCAGCAGAGTCGCACACACCACCACCACCTTCACCACTCACGCCGTGAGACTTGGGAGCTCCTCCCTCTCTCACATCCGCTTGTAACCCCTACTATAAGCACTTCAGTGCAAGATAATATAGAACTCTCTCCCTCCACTGGACGTAGGGCATTTCTCGGCCCGAGCCAGTATAAATCGCTGTGTCCTTCTTGCATACCATCCGGACTCCGACACACAAACACTCATTACTCATTGGTGCTAGAACCGTCGTTTCTTACGCCGACAAGATTATTCTGTGATCTTCTGCTCCCAAGCTGTCAGTACGAAGAATCGACTAGAACCTGCATGTCACATGATTCGGTGTCCCGTGGAAAAGCGAAACATGGGAGGACAAGCTCGCTTATTCTGCTCTGAAGTTAACTTTTCATTTCAAAATTCCCCTAGCAATATGATTATATATAATATCCTCTATTCCTCCAGCAGTCCAGCTCCATTAAAGAAAATTAAGTTTTGATCCAACTGAAAATTTTGGAGTTATTAGGAATCGATCTCTCCATATGAGTGGAGCATGGACCCTGAGATGTTCTACTTTATGTGCCTAAACAGAGGGGATCAGAATTACAGCATATTTGAAGAGTACCGTGTGCCTGTTGTTTGCTTGTCCATATGAAAACCTCCTCATTACAATTTGCTTTGGTGCCTTAAGTGTAATCACAAGATCCCCGTTTCAGGTTGTTGCAGA

>SsCNGC10-1T

TTCCCCATTTGTATTATTATTATCATCTGAACTTCAACCAAAACGCTGCGCCCCCGGAATGGCAGTCATGAGGGAAGGAGATCTTCAGATTTGTCGGGTACCACAATTAGGGGTACCCAAATTATGATGGAAAATCACCCGCCCCCCCCCCCATTTAAGGGAATAGATCAACAAAGGAGAAACGGGCTAAACTCCCCCTTGCTGGGCCTCCAGCAAGACGCGTGAGCAAGCCAGCCCATATCCCCACTTCATCCGAGGCCTCACTCAGAGGAGTCTCGGAACACCTCCGGTTCGCCTGAGGCCTCCACTCTGAGGTCTCGGACACAGGCCGACCCCCCGGCAACGTCACCGCCTCGCCCGAGCCCCAGAGGCGACATTGTCCGTCTCCGACTCGCCCGAAACCGGCCTCTAATGCCGCTCCCCCATTGGCGCGCTCGAGACCCTCTGTGACATCACCCACGTTCTCGCATCTGCCCAACCGGACAGGCCGGGAATGACCGGGGTCAAATCACCACGCCTTCGACTGTAATAGGCATGGGGAAGAGCTGCTGACCACTCTACCACCCATACTGGCACTATATGCACGATTCGTGTGTGCGCAGTGTACGGAGCAACTGCTCTGGGCCATTATTCCCACGATCGGATAAGATCGCACCACATTTCCTGCCTCCGAGGCCTCGGCCCTCTACGTCGAGTCAGACTACAAGGACAAATACGCCACACCGGCTACAGGACCAGGTATGACAAAGCACATCGCTACAATGCCAAAAGACAGAGACCACTCCTTCAGCAGTGGCACACGTACCAGAACACGTAGTAGCAGTACCAGAAGTACCGATACCATGCCTCCACAGTACAGACCACGCCGCCACCGGATAAGGCCCTTCCGAGGCCTCCAGGCTCCCCCACGGCAAGGTCGCACCATATGCCAACAATCATGTAATCATGCACCTCCTTGAGACTATAAAAAGAGCATGCAGGGATCATTCCGAAGAAAAAAAAGAAAAAAGAGAGAACTCAGTCGAGCACAACTCAACTGAACCACCATAGCAACATAACGCGAGCTACGAGTAGGCTACTCTCCTGGACCTTGCGAGGTAACAAGCAGAGTCGCACACACCACCACCACTTTCACCACTCACGCCGTGAGACTTGGGAGCTCCTTCCTCTCTCACGTCCGCTTGTAACCTTACTACAAGCACTTCAATGCAAGATAATACAGAACTCTCCCCCTCCACTGGACGTAGGGCATTTCTCGGCCCAAACCAGTATAAATCGTCTATGTCCTTCTTGCACACCATCCGAACTTCCGATGCGCAAACACTCATTACTTGTTGGTGCTAGGACCACCGTTTCTTACGCCGACAAGATTATTCTGTGATCTCCTGCTCCCAAGCTGTCAGTACGAAGAATCGACTAGAACCTGCATGTCACATGATTCAGTGTCCCGTGGAAAAGCGAAACATGAGAAGACAAGCTCGCTTATTCTGCTCTGAAGTTAACTTTTCATTTCAAAATTCCCCTAGCAATATGATTATATATAATATCCTCTATTCCTCCAGCAGTCCAGCTCCATTAAAGAAAATTAAGTTTTGATCCAACTGAAAATTTTGGAGTTATTAGGAATCGATCTCTCGATATGAGTGGAGCATGGACCCTGAGATGTTCTACTTTATGTGCCTAAACAGAGGGGATCAGAATTACAGCATATTTGAAGAGTACCGTGTGCCTGTTGTTTTCTTGTCCATATGAAAACCTCCTCATTACAATTTGCTTTGGTGCCTTTAAGTGTAATCACAAGATCCCCGTTTCAGGTTGTTGCAGATGACCTTTTTAGTTCTTATGGCTGAGCACAATGTCGTGGTCACTTTCTGTCCCATAGGCCATAGCTGAAGATCTATTGGCACGCCAGTTTCCACGTCGGTCGTAGCACTCGTTTTTGCTTATAAGTTGTATCGATGAATACATTAATGTCTGTGACCAAAAGCTGTGAGAGATCAC

>SsCNGC10-2C

ACCCAAATTGTGATGGAAAATCACCCCCCCCCATTTAAGGGAATAGATCAACAAAGGAAAAACGGGCTAAACTCCCCCTTGCTGGGCCTCCAGCAAGACGCGCGAGCAAGCCAGCCCATATCCCCACTTCATCCGAGGCCTCACTCAGAGGAGTCTCGGAACACCTCCGGTTCGCCTGAGGCCTCCGCTCTGAGGTCTCGGACACAGGCCGACCCCCGGCAACGTCACCGCCTCGCCCGAGCCCCAGAGGCGACATTGTCCGTCTCCGACTCGCCCGAGACCGGCCTCTAATGCCGCTCCCCCATTGGCGCGCTCGAGACCCCCTGTGACATCACCCACGTTCTCGCATCTGCCCAACCGGACAGGCCAGGAATGACCGGGGTCAAATCACCACGCCTTCGACTGTAATAGGCATGGGGAAGAGCTGCTGACCACTCTGCCACCCATACTGGCACTATATGCACGATTCGTGTGTGCGCAGTGTACGGAGCAACTGCTCTGGGCCACTATTCCCACGATCGGATAAGATCGCACCACATTTCCTGCCTCTGAGGCCTCGGCCCTCTACGCCGAGTCAAACTACAAGGACAAATACGCCACACCGGCTACAGGACCAGGTATGACAAAGCACGTCACTACAATGCCAAAAGACAGAGACCACTTCTTCAGCAGTGGCACACGTACCAGAACACGTAGTAGCAGTACCAGAAGTACCGATGCCATGCCTCCACAGTACAGACCACGCCGCCACCGGATAAGGCCCTTCCGAGGCCTCCAGGCTCCCCCACCGGCAAGGTCGCACCATATGCCAACAATCATGTAACCCTGCACCTCCTTGAGACTATAAAAGGAGCATGCAGGGATCATTCCGAAGAAAAAAGAAAAAAGAGAGAACTCAGTCGAGCATAACTCAACTGAACCACCATAGCAACATAACGCGAGATACGAGTAGGCTACTCTCCTGGACCTTGCGAGGTAACTAGCAGAGTCGCACACACCACCACCACTTTCACCACTCACGCCGTGAGACTTGGGAGCTCCTCCCTCTCTCACGTCCGCTTGTAACCCACTACACAAGCACTTCAGTGCAAGATAATACAGAACTCTCCCCCTCCACTGGACGTAGGGCATTTCTCGGTCCGAACCAGTATAAATTGTCTATGTCCTTCTTGTACACCATCCGAACTCCCGATGCACAAACACTCATTACTCGTTAGTGTTAGGACCGCCGTTTCTTACGACAAGATTATTCTGTGATCTCCTGCTCCCAAGCTGTCAGTACGAAGAATCGACTACAACCTGCATGTCACATGATTCAGTGTCCCGTGGAAAAGCGAAACATGGGAAGACAAGCTCGCTTATTCTGCTCTGAAGTTAACTTTTCATTTCAAAATTCCCCTAGCAATATGATTATATATAATATCCTCTATTCCTCCAGCAGTCCAGCTCCATTAAAGAAAATTAAGTTTTGATCCAACTGAAAATTTTGGAGTTATTAGGAATCGATCTCTCGATATGAGTGGAGCATGGACCCTGAGATGTTCTACTTTATGTGCCTAAACAGAGGGGATCAGAATTACAGCATATTTGAAGAGTACCGTGTGCCTGTTGTTTGCTTGTCCATATGAAAACCTCCTCATTACAATTTGCTTTGGTGCCTTTAAGTGTAATCACAAGATACCCGTTTCAGGTTGTTGCAGATGACCTTTTTAGTTCTTATGGCTGAGCACAATGTCGTGGTCACTTTCTGTCCCATAGGCCATAGCTGAAGATCTATTGGCACGCCAGTTTCCACGTCGGTCGTAGCACTCGTTTTTGCTTATAAGTTGTATCGATGAATACATTAATGTCTGTGACCAAAAGCTGTGAGAGATCACCATGGCATCCGGTGCTTCACGAAATGTCAGGTGAGCGCCTTCCCGCGCGATCAGATAACTGAACAAATCCACGTTACTGAACAAAGGATTCAGCAGGATCAATCAATGTTTATAACGAAAGCCA

>SsCNGC10-3D

CACCTATTTTTCCCCATTTGTATTATTATTATCATCTGAACTTCAACCAAAACGCTGCGCCCCCGGAATGGCAGTCATGAGGAAGGAGATCTTCAGATTTGTCGGGTACCACAATTAGGGGTACCCAAATTATGATGGAAAATCACCCGCCCCCCCCATTTAAGGGAATAGATCAACAAAGGAGAAACGGGCTAAACTCCCCCTTGCTGGGCCTCCAGCAAGACGCGTGAGCAAGCCAGCCCATATCCCCACTTCATCCGAGGCCTCACTCAGAGGAGTCTCGGAACACCTCCGGTTCGCCTGAGGCCTCCACTCTGAGGTCTCGGACACAGGCCGACCCCCGGCAACGTCACCGCCTCGCCCGAGCCCCAGAGGCGACATTGTCCGTCTCCGACTCGCCCGAAACCGGCCTCTAATGCCGCTCCCCCATTGGCGCGCTCGAGACCCTCTGTGACATCACCCACGTTCTCGCATCTGCCCAACCGGACAGGCCGGGAATGACCGGGGTCAAATCACCACGCCTTCGACTGTAATAGGCATGGGAAGAGCTGCTGACCACTCTACCACCCATACTGGCACTATATGCACGATTCGTGTGTGCGCAGTGTACGGAGCAACTGCTCTGGGCCATTATTCCCACGATCGGATAAGATCGCACCACATTTCCTGCCTCCGAGGCCTCGGCCCTCTACGTCGAGTCAGACTACAAGGACAAATACGCCACACCGGCTACAGGACCAGGTATGACAAAGCACATCGCTACAATGCCAAAAGACAGAGACCACTCCTTCAGCAGTGGCACACGTACCAGAACACGTAGTAGCAGTACCAGAAGTACCGATACCATGCCTCCACAGTACAGACCACGCCGCCACCGGATAAGGCCCTTCCGAGGCCTCCAGGCTCCCCCACGGCAAGGTCGCACCATATGCCAACAATCATGTAATCATGCACCTCCTTGAGAATATAAAAGAGCATGCAGGGATCATTCCGAAGAAAAAAAAGAAAAAAGAGAGAACTCAGTCGAGCACAACTCAACTGAACCACCATAGCAACATAACGCGAGATACGAGTAGGCTACTCTCCTGGACCTTGCGAGGTAACAGCAGAGTCGCACACACCACCACCACTTCACCACTCACGCCGTGAGACTTGGGAGCTCCTCCTCTCTCACGTCCGCTTGTAACCTTACTACAAGCACTTCAATGCAAGATAATACAGAACTCTCCCCTCCACTGGACGTAGGGCATTTCTCGGCCCAAACCAGTATAAATCGTCTATGTCCTTCTTGCACACCATCCGAACTTCCGATGCGCAAACACTCATTACTTGTTGGTGCTAGGACCACCGTTTCTTACGCCGACAAGATTATTCTGTGATCTTCTGCTCCCAAGCTGTCAGTACGAAGAATCGACTAGAACCTGCATGTCACATGATTCAGTGTCCCGTGGAAAAGCGAAACATGAGAAGACAAGCTCGCTTATTCTGCTCTGAAGTTAACTTTTCATTTCAAAATTCCCCTAGCAATATGATTATATATAATATCCTCTATTCCTCCAGCAGTCCAGCTCCATTAAAGAAAATTAAGTTTTGATCCAACTGAAAATTTTGGAGTTATTAGGAATCGATCTCTCGATATGAGTGGAGCATGGACCCTGAGATGTTCTACTTTATGTGCCTAAACAGAGGGGATCAGAATTACAGCATATTTGAAGAGTACCGTGTGCCTGTTGTTTTCTTGTCCATATGAAAACCTCCTCATTACAATTTGCTTTGGTGCCTTTAAGTGTAATCACAAGATCCCCGTTTCAGGTTGTTGCAGATGACCTTTTTAGTTCTTATGGCTGAGCACAATGTCGTGGTCACTTTCTGTCCCATAGGCCATAGCTGAAGATCTATTGGCACGCCAGTTTCCACGTCGGTCGTAGCACTCGTTTTTGCTTATAAGTTGTATCGATGAATACATTAATGTCTGTGACCAAAAGCTGTGAGAGATCACCA

>SsCNGC11

GAATGGCAGTCATGAGTGAAGGAGATCTTCAGATTTATTGGGTACTACAATTAGGGGTACCCAAATTGTGATGGAAAATCACCTCCCCCATTTAAGGGAATAGATCAACAAAGGAGAAACGGGCTAAACTCCCCCTTGCTGGGCCTCCAGCAAGACACGTGAGTAAGCCAGCCCATATCCCCACTTCATCCGAGGCCTCACTCAGAGGAGTCTCGGAACACCTCCGGTTCGCCCGAGGCCTCCGCTCTGAGGTCTCGGACACAGGCCAACCCCCGGCAACGTCACCGCCTCGCCCGAGCCCCAGATGCGACATTGTCCGTCTCCGACTCGCCCGAGACCGGCCTCTAATGCCGCTCCCCCATTGGCGCGCTCGAGACCCCCTGTGACATCACCCACGTTCTTGCATCTGCCCAACCGGACAGGCCGGGAATGACCGGGGTCAAATCACCACGCCTTCGACTGTAATAGGCATGGGAAAGAGCTGCTGACCACTCTGCCACCCATACTGGCACTATATGCACGATTCGTGTGTGCGCAGTGTACGGAGCAACTGCTCTGGGCCACTATTCCCATGATCGGATAAGATCACACCACATTTCCTGCCTCCGAGGCCTCGGCCCTCTACGGTGAGTCACACTACAAGGACAAATACGCCACACCGGCTATAGGACCAGGTATGACAAAGCACGTTGCTACAATGCCAAAAGACAGAGACCATTCCTTCAGCAGTGGCACACGTACCAGAACACGTAGTAGCAGTACCAGAAGTACTGACGCCATGCCTCCACAGTACAGACAACGCCGCCACCGGATAAGGCCCTTCCGAGGCCTCCAGGCTCCCCCACCGGCAAGGTCGCACCATATGCCAACAATCATGTAACCCTGCACCTCCTTGAGAATATAAAAGGAGCATGCAGGGATCATTCCGAAGAAAAAAAGAAAAAAAGAGAGAACTCAGTCGAGCACAACTCAACTGAACCACCATAGCAACATAACGCGAGATACGAGTAGGCTACTCTCTTGGACCTTGCGAGGTAACCAGCAGAGTCGCACACACCACCATCACCTTCACCACTCACGCCGTGAGACTTGGGAGCTCCTCCCTCTCCTCTACTGGACGTAGGGCATTTCTCGGCCCGAGCCAGTATAAATCGTCTGTGTCCTTCTTGCACACCATCCGGACTCTCGACACACAAATACTCATTACTCGTTGGTGCTAGGACCGCCGTTTCTTACGCCGACAAGATTATTCTGTGATCTTCTGCTCCCAAGCTGTCAGTACGAAGAATCGACTAGAACCTGCATGTCACATGATTCAGTGTCCCGTGGAAAAGCGAAACATGGGAAGACAAGCTCGCTTATTCTGCTCTGAAGTTAACTTTTCATTTCAAAATTCCCCTAGCAATATGATTATATATAATATCCTCTATTCCTCCAGCAGTCCAGCTCCATTAAAGAAAATTAAGTTTTGATCCAACTGAAAATTTTGGAGTTATTAGGAATCGATCTCTCGATATGAGTGGAGCATGGACCCTGAGATGTTTCACTTTATGTGCCTAAACAGAGGGGATCAGAATTACAGCATATTTGAAGAGTACCGTGTGCCTGTTGTTTGCTTGTCCATATGAAAACCTCCTCATTACAATTTGCTTTGGTGCCTTTAAGTGTAATCACAAGATCCCCGTTTCAGGTTGTTGCAGATGACCTTTTTAGTTCTTATGGCTGAGCACAATGTCGTGGTCACTTTCTGTCCCATAGGCCATAGCTGAAGATCTATTGGCACGCCAGTTTCCACGTCGGTCGTAGCACTCGTTTTTGCTTATAAGTTGTATCGATGAATACATTAATGTCTGTGACCAAAAGCTGTGAGAGATCACCATGGCATCCGGTGCTTCACGAAATGTCAGGTGAGCGCCTTCCCGCGCGATCAGATAACTGAACAAATCCACGTTACTGAACAAAGGATTCAGCAGGATCAATCAATGTTTATAACGAAAGCCA

>SsCNGC11-2B

GCGGTAGCGGAGACTAATGCGGCTTCATCCTCTTCAGCAACCGGGGCGCTGACACTGGGCTCCACATCCAGATGCGGCGCAAGTACCGGGCGTCATTCACAGGTGCCATGCGATCGCGTTTGGGTCCACGTGGCAACCATCAAGCAGGGGGCACATGGGTGCTCCCGTCGTCCACCTATTTTTTCCCCATTTGTATTATTATTATTATCATCTGAACTTCAACCAAAACGTTGCGCCCCCGGAATGGCAGTCATGAGTGAAGGAGATCTTCAGATTTATTGGGTACTACAATTAGGGGTACTTAAATTGTGATGGAAAATCACCCGCCCCCTATTTAAGGGAATAGATCAACAAAGAAGAAACGGGCTAAACTCCCCCTTGCTGGGCCTCCAGCAAGACACGTGAGCAAGCCAGCCCATATCCCCACTTCATCTGAGGCCGCACTCAGAGGAGTCTCGGAACACCTCCGGTTCGTCCGAGGCCTCCGCTCTGAGGTCTCGGACACAGGCCGACCCCCGGCAACATCACCGCCTCGCCCGAGCCCCAGAGGCGATATTGTCCGTCTCCGACTCGCCCGAGACCGGCCTCTAATGCCGCTCCCCCATTGGCACGCTCGAGACCCCTGTGACATCACCCACGTTCTCGCATCTGCCCAACCGGACAGGTCGGGAATGACCGGGGTCAAATCACCACGCCTTCGACTGTAATAGGCATGGGAAAGAGCTGCTGACCACTCTGCCACCCATACTGGCACTATATGCACGATTCGTGTGTGCACAGTGTACGGAGCAACTGCTCTGGGCCACTATTCTCACGATCGGATAAGATCACACCACATTTCCTGCCTCCGAGGCCTCGGCCCTCTACGCTGAGTCAGACTACAAGGACAAATACGCCACACCGGCTATAGAACCAGGTATGACAAAGCACGTCGCTACAATGCCAAAAGACAGAGACCACTCCTTCAGCAGTGGCACACGTACCAGAACACGTAGTAGCAGTACCAGAAGTATTGACGCCATGCCTCCACAGTACAGACCACGCCGCCACCGGATAAGGCCCTTCCGAGACCTCCAGGCTCCCCCACCGGCAAGGTCGCACCATATGCCAATAATCATGTAACCCTGCACCTCCTTGAGAATATAAAAGGAGCATGCAGGATCATTCCAAAGAAAAAAAGAAAAAAGAGAGAACTCAGTCGAGCACAACTCAACTGAACCACCATAGCAACATAACGCGAGATACGAGTAGGCTACTCTCTTGGACCTTGCGAGGTAACCAGCAGAGTCGCACACACCACCACCACCTTCACCACTCACGCCGTGAGACTTGGGAGCTCCTCCCTCTCTCACATCCGCTTGTAACCCCTACTATAAGCACTTCAGTGCAAGATAATATAGAACTCTCTCCCTCCACTGGACGTAGGGCATTTCTCGGCCCGAGCCAGTATAAATCGCCTGTGTCCTTCTTGCATACCATCCGGACTCCCGACACACAAACACTCATTACTCATTGGTGCTAGAACCGTCGTTTCTTACGCCGACAAGATTATTCTGTGATCTTCTGCTCCCAAGCTGTCAGTACGAAGAATCGACTAGAACCTGCATGTCACATGATTCGGTGTCCCGTGGAAAAGCGAAACATGGGAGGACAAGCTCGCTTATTCTGCTCTGAAGTTAACTTTTCATTTCAAAATTCCCCTAGCAATATGATTATATATAATATCCTCTATTCCTCCAGCAGTCCAGCTCCATTAAAGAAAATTAAGTTTTGATCCAACTGAAAATTTTGGAGTTATTAGGAATCGATCTCTCCATATGAGTGGAGCATGGACCCTGAGATGTTCTACTTTATGTGCCTAAACAGAGGGGATCAGAATTACAGCATATTTGAAGAGTACCGTGTGCCTGTTGTTTGCTTGTCCATATGAAAACCTCCTCATTACAATTTGCTTTGGTGCCTTAAGTGTAATCACAAGATCCCCGTTTCAGGTTGTTGCAG

>SsCNGC12

AACTAATCTAATGATACTTATTTTGTATCATAAACATTAGTATTTTTTATATAAATTTATTTAAAGTTCAAACTGTTTGACTTCTTAAAAAGTGAGAATTGCATGTTTTTTTACGGAGCGAGTACATTATAATGCGTCGTCAAGAAAGATTTTTTTTTTAAAAGACGATGACGACGACGAAGAATCCAGCAGGTCTCCATCCTGCACCGGCCACGACCCACAGGTGCGGTCGCGGCGCCTCAGCTGCTGGCTGCTGCTAGCGACACTTCCACCACAACCATGTTCAAAACTTCAAACTGACTAGACGGTCCACAGATCTACACATCCCTCGTGCCCTGGTGTCAGGTGACAAAAGTTCACAAACCTAGCACGCCACGCCTCCGCCTCCGCGTCCGCTCGTGCTAAACGAGCCGAGACGTCTTTTTGAGTCCTCGCTCCTTCTCCGCTCTCTGCCATCTCACTCCCTGTCTCTGCTTAGCGGCGAAGCCAGAGAAACGGGAGTAATAGATTAAAGAAAAAGAAGAGAGAGAACTCGCTCTCGCCCGCTGTGCGAATTGAACAGCTCGAAAGCGATTTGCTGTTGCCTGCTGCTGCGTGCGTTTCGGCTGCTGATCCCGAGCACTTCCTCGCATCGCAGCGAACAGTTCAGGTTAGCCACTCCGACCTTCCTTTACCTATCGCTGTGTGTGTCTCGGAAGATTGCAGCTGTGGTTGCGGTGGGAGTTGATTTTTGGTTGCTTATGTTTGTGCAAAATCTGGGGGGTTTCCGACGCGCTGCAAAGCTCGCGCAGCGCGAGTCTGGAGTAATTTCTGGGATTGCTTCCGGTTCCATAATTTCCTTACGAGAGGTGGCGCCGCGGGTCGACACGTTTTGGATGGATTTATTGTCCTTTTTTTTTCCGCGCATTTTCCAAATCTCATCACTGGAGGATGGGAGGTTGAAGTTGCTTTATCGGGACTTTGTTTATTTTTTGCATCTCACGGCTGTAATTTTTGTCGTGGTCCTTTTCCCATTTGGGGTTTACGGGGATGCTCTGGTAATCTCTTTAAGCAGGCGGCTATTGTTTCATCGGATTCCGTTTATGCGCTCTCTACTTGCGCGAAGCGGCCTCTAGGGCTAACAAATTGCTTCTGTTCCTGATCGTCTCTCTCCCCCACCTCCCTCAACCAGTTGTAATGGGTAACCAGAATCCTCGTTAGACGCTAAATCGCGTCAGTTAGGTTCTGTTGATTAGCGGTCTCATGTGCATGTACTGCCTCCCTCCCAAAATAGATGCAATTGGGGTTTTAGATTGAGTCAAAATAAGTTAGGTTTGATTAAATATATTTCATGGAGTACATTTTGTGCATATATTTGGCCAAAGTTGAGATAGATTGACTTGATAGAAAGCTAGAATTGCTCAATTGCATCCTTTTTTTGGTTGGAGTACTGCACACACAACTCACGCACCTTTTAACTGGCTCATGTTTACTGTAATGTGCACATTATGAGATCCATAGTTTTCTATAACTCTTCAAACCTTATTTTTCATGCTGTTGCGTTTGCAGGGTCAGCAGAATTTTAGATGAAGACTTGATTTCTCCTTTCAGATAAAGGTAGGTCTTAGCAGTGTTAGTATTAATGCAACTTGTTTTTGTTTTGTTTATATTGCTCATATTCTATTTTCTTGGAATTGTTTTATGTTGTTTCAGCAGTTGCCATTTATGTGCCTTTGCAAGCTAATGGTAGTGATAATTACGAAGTCTTATTTATTTCGATGGGTCATTTTTAACCTTGGACACCACATAATTATTCTTCTGTTGTTTTCTCACATTTTTTACTGTTATATCCCACTGTAATTGACCAGTAAATATTGGCTAGCCTGGATATCCATGGAATTCTTACTTTCTAAAGAAGGACAATCTCATTTGTTTATAAAGGATACATGCATGCTTGCAGAGAATCTATATTTTGGCTGTTTAATTGAAAGTTATGTGCTAATGTAATGGATCTGTGCTGCA

>SsCNGC12-2D

CATTAGTATTTTTTATATAAATTTATTTAAAGTTTAAACTGTTTAACTTCTTAAAAAGCGAGAATTACATATTTTTTTTACGGAGCAAGTACATTATAATGCGTCGTCAAGAAAGATTTTTTTTTTTAAAAAAAGACGACGACGACGACGAAGAATCCAGCAGGTCTCCATCCTGCACTGGCCACGACCCACAGGTGCGGTCGCGGCGCCTCAGCTGCTGGCTGCTGCTAGCGACACTTCCACCACAACCATGTTCAAAACTTCAAACTGACTAGACGGTCCACAGATCTACACATCCCTCGTGCCCTGGTGTCAAGTGACAAAAGTTCACAAACCTAGCACGCCACGCCTCCGACTCCGCCTCCGCGTCCGCTCGTGCTAAACGAGCCGAGACGTCTTTTTGACTCCTCGCTCCTTCTCCGCTCTCCGCCATCTCACTCCCTGTCTCTGCTTAGCGGCGAAGCCAGAGAAACGGGAGTAATAGATTAAAGAAAAAGAAGAGAGAGAACTCGCTCTCGCCCGCTGTGCGAATTGAACAGCTCGAAAGCGATTTGCTGTTGCCTGCTGCTGCGTGCGTTTCGGCTGCTGATCCCGAGCACTTCCTCGCATCGCAGCGAACAGTTCAGGCTAGCCACTCCGACCTTCCTTTACCTATCGCTGTGTGTGTCTCGGAAGATTGCAGATGTGGTTGCGGTGGGAGTTGATTTTTGGTTGCTTATGTTTGTGCAAAATCTGGGGGGTTTCTGCGGGGGTTTCCGACGCGCTGCAAAGCTCGCGCAGCGCGAGTCTGGAGTAATTTCTGGGATTGCTTCCGGTTCCATAATTTCCTTACGAGAGGTGGCGCCGCGGGTCGACACGTTTTGGATGGATTTATTGTCCTTTTTTTTCCGCGCATTTTCCAAATCTCATCACTGGAGGATGGGAGGTTGAAGTTGCTTTATCGGGACTTTGTTTATTTTTTGCATCTCACGGCTGTAATTTTTGTCGTGGTCCTTTTCCCATCTGGGGTTTACGGGGATGCTCTGGTAATCTCTTTAAGCAGGCGGCTATTGTTTCATCGGATTCCGTTTATGCGCTCTCTACTTGCGCGAAGCGGCCTCTAGGTCTAACAAATTGCTTCTGTTCCTGATCGTCTCTCTCCCCCACCTCCCTCAACCAGTTGTAATGGGTAACCAGAATCCTCGTTAGACGCTAAATCGCGTCAATTAGGTTCAACTTGATTAACGGTCTCATGTGCATGTACTGCCTCCCTCCCAAAATAGATGCAATTGGGGTTTTAGATTGAGTCAAAATAAGTTAGGTTTGATTAAATATATTTCATGGAGTACATTTTGTGCATATATTTGGCCAAAGTTGAGATAGATTGACTTGATAGAAAGCTAGAATTGCTCAATTGCATCCTTTTTTTGGTTGGAGTACTGCACACACAACTCACGCACCTTTTAACTGGCTCATGTTTACTGTAATGTGCACATTATGAGATCCATAGTTTTCTATAACTCTTCAAACCTTATTTTTCATGCTGTTGCGTTTGCAGGGTCAGCAGAATTTTAGATGAAGACTTGATTTCTCCTTTCAGATAAAGGTAGGTCTTAGCAGTGTTAGTATTAATGCAACTTGTTTTTGTTTTGTTTATATTGCTCATATTCTATTTTCTTGGAATTGTTTTATGTTGTTTCAGCAGTTGCCATTTATGTGCCTTTGCAAGCTAATGGTAGTGATAATTACGAAGTCTTATTTATTTCGATGGGTCATTTTTAACCTTGGACACCACATAATTATTCTTCTGTTGTTTTCTCACATTTTTTACTGTTATATCCCACTGTAATTGACCAGTAAATATTGGCTAGCCTGGATATCCACGGAATTCTTACTTTCTAAAGAAGGACAATCTCATTTGTTTATAAAGGATACATGCATGCTTGCAGAGAATCTATATTTTGGCTGTTTAATTGAAAGTTATGTGCTAATGTAATGGATCTGTGCTGCAGTGTTTCAAAA

>SsCNGC13

CAGGACACTTGTCTGATTTCTGTCAATGCCATTGGATTTTTCATACCTATTTAGTAGTCTTTGTCGTTCAGATGATCGTGTAATTTCTTCAAGTTTTTTGTCGCATAATATAAGAATGCATATTCGTTTTTGTTTCGCATGATTGCACCTGTCAATGTTACGTGGGGCCTGGCTGCAAGGTGTGTGTGGACAGTGCTTATTTGCACCGGTTGGTCAATGATAGAAATTATTACTTATATCCTTTCCAAATCAATGGATGGGTTATTGATATACTATTGTTTATCCTTGCAAAACGATTCAAGTTATTATTCTAAAGGGTCTATCTAACTGACTGATCAACAATCGACATGTTTAGTCGTTTAGAGCTCAGACGTTCCCACATGTTCCTTTTGTTATACAAGAGTTGCTAGCCACCCCTTTCCTTTCTACATTACTTTTTGATGTTTTCCCTATGCAGGGTGGATCCAAGATTCAAAACCTGTAGAAGTGGCCCCTTTTTGTGAGAAGGAGAGTCGAAATCCTGTCCTGTAGGTTGCGCTTCTTGCATCCCTATCACTGCACTACGTGCACGTTCTCTATAGGGCGAAGCTTATTGATTTAAGTCAATTAACACAAGCATACAATCAACATATATAATTGCAAATTTTGTGAAGGGGATTAACTGTATTTTGGCAGAAGCTGTGGGTCCATCTCACTGTCCCTACGTATTTGTCTTCAAAGAGAAAGAAGGGAGCCATGTCAATGTTCACAAAAAAGAATTTTTATTAGTATACTATATCAAAAAAAATGGCAGCCACAAATTGAACAACGCTTTTTCTGAGAAAAAAAAAGGAATTGTATTGAGGGCTTTAATTATCACACATGCCAATCATGCATCTGCATGAAGGATCTTGAAGGTGGAATATAATAGTTTTCTTTACGTTTTTGTATGTTCTCGAGATTTCCTTTTATGTCAAGGTTCGCAAAGAAACATATAGATTTGCTTTGCTTTACTTTATAGCTTTTGTTTTCTCAACAAGGTGGTCCATCAAATTCTGCAGGTCATCTTCTGGCTGGTTATACCAAAGTTAATTAGGGAAGAGCAAGTTAAGCTTAGAATTCCACGTGGACAAGTTGTCGCGGCCGCTTCTATAGTGTCACCTAAAAGCGGCCGCGACAACTTGTCCACGTGGAATTCTAAGCTCTAGGAAACTATTGTCATAATAATCTGCTACATGTTCAGAATATTCTTTCTTTCTTTCTTATACTGTTTATCTGAATAAACAAGACACCAAACTGCTACCCCAATTCCACTAAAAAATTCTAGCCTTGTCTAGCCTTGTTCCATGCATGTATTTCTTTACATATTTTTTTAATGCTGTTGCCCTATTATGTTCAACAACTGCATATTGGTAAACTTGTACAATTCCTTGAATTGCTTGGCAGTACTTTTGGTAACGATCTGGCCCCTACAAGCAATGGTATTGAGGTGATATTCAGCATAATCAATGTCCTCAGTGGCCTGATGCTCTTCACATTGCTGATCGGAAACATACAGGTAAAACAGTGTCATTTCTGGTCCTTGCGTGATTGTATCACACCGCTTCACTAACTACAAGTCTACAACTTACTGAATCTGCAGGTGTTTCTGCACGCCGTCCTGGCAAGGAAGAGGAAGATGCAGCTGCGGTTCCGGGACATGGAATGGTGGATGAGACGGAGGCAGCTGCCATCCCGGCTGAGGCAAAGGGTCCGCAAATACGAGCGCGAACGCTGGGCCGCCGTCACGGGAGACGAGGAGATGGAGATGATCAAGGACCTGCCTGAAGGACTCAGGCGGGACATCAAGCGCTACCTCTGCCTCGAGCTGGTTAAGCAGGTACAGTCTCTGTCTCTCGTAGTCTCATAAAGTTACTGAAGTTGTTCTGATTTTTTTAACAGTTTTTTTTATAAAAAATGTTCAGTTTTTTTTTACATGAATCATGAATGTGCGATGAAATTGTTAGAAAATTTTCCCTTGCC

>SsCNGC13-1P

TATAGGACGAGAGGAGGCCAAGGCGACGGGGAGTCTTGAAATGATGTTTTGTCGATCCCTGTTGGGGTGACTATAGCGACGTGCCGTAGAAATGGGGGGCCATCCCTGGGCACGTGATGTCGTAGAAGGTGAGGTGCTGAAAAGACCCGGAGCGCCGTGCCATACCGTCCTTTCTTCCCTGTTGCGCTGTGTCGAGGGTCGAGGCCTGGTCGAGGTAGGGTCGAGGCCGAGCGGATAAGTCCGAATCCCAGGAATTACGGCGTACGGGCTTTGTCGTATCCTCCCTCGAGGGTCGCTAGTGATGCATAGTGTTAGGTATGGTGGGCACAGTGACGTTTTTCACTTCCCATCACGGGATGGGCGGTAAAACAGTGGAAACTGACACTGGTATTGTTCCCACGTCGACCCAGGCCGCGGCCATGCCGGTAGGTGGTAGTTGGTAGTCGTATTAAATGCCTTGACTTTTCGTCTCGTGGTAAGTGGAGACAGAGGGGGTCGAGCGCCTCGACCCGGGCTCACGCGAGTCGGAGCGCTCCCGGGGGGCGGTCGAGCCCCGTGCGGGTCGGAGCGCGGTCGAGGCCCCCCGTCGAGGGGGCCTCGTACGAGTCGGAGACAGGACGCGGTCGAGGCATTCCGTCGAGCCGACCTCGTACGAGACGGACCTGCCGCGCGTAGTGGGCTGTGCTTTGTCGCGACTTAGTCGTTTTAACTTTTATTATCGTTGAATGTGGGTACCCCTATAGTATTGGTACCGACATAATGATATTTATTTAATATTATAAATATTGATAATTTTTAACCTATAATTGATATGTGCTAGAATTGTGTTTTCTTTTTTTGAACGGATAGAGTATTAAAAAAGATAATAGCAGCTTTCATAGATGACAACCATTTCAGGCCCAGGCAGGCTCCCGAGCTAGGCCCCACCGGGCACCGGCTCAGGCTATGGACCAAGCATGCGAATTCTTAGCCCAGGCTCCAGGCCCACATGCACCTCCGCACCTGTACGAGAGTATTCCACCTCGCTCACATGAACCCAGCCCATGCACCGCGAGCTCACGTCCAGGTCCAGCTGCAGAGGTCCGCGCCACCGCGGTCACCAGGCCACGCTCGCCGCATAAAACCAAAAGAAAAGAAAAAAAATGCATTGGTACTCGCAGGCCTACTTGGTCGTCGCGGCGCGGCATCACCGTCGTACGGAGCCGGAGACTTGCCACAAGCCCACTCTCTCCGGTCTCCGCCGCACAAGAGCAGAAGAAGAAAGAAAACTCCGGTGCTCCGCGTAGCTTCGGAAACACGGCCCTGTTGTAGCCATCTGGTGCCTGGTGCCACCATAGAACGTTCGTTTTCCGTAAATCGACGTTCCGCTTAGCTATGGCTAGTACAGTATATATCAAAATACTAGCCCATCTCATGATTCGTGTTAGGTATACATGCATGGTGTTAATGAGAAGTGAGACAGCCATTCGTTGTTGACGGAGCAGCTTAGCTTCATTACTTAAACCGGGGAAAATGGCACGCATATATCGGCGTCGGGTACAAAATCGGATCATCATCATCATCGAACCCCTAGCGCGATCAATGATTATTCGGTGATGTGCTCGGCTCACCAGTCACCTGTGCCAGGAGCCGAGTGGCCCCACTACGAACAATTGCTCCCGTCAATCTGGTAGGTGTAAACGTGTAGGCGTAGGCGCCCCGAGGCGAGGACGGGAGGTCCGAGGGCTCGGCTGCTCGGCATCGCACAAGGACGGTGCACGTACGCGCCACGTCGGCACGTCCTTGGGCCCCGTCACAGTTGTACCCTCCGCCACTCTCGTCTTTACTTGCCCCATCCATCCGTCCCACGCCGAGCGCCAGAAGCAGGCTACCTCCCGCTCCGCCGCTCGCTAGTCACTAGCTACTGGACCGCGCCTCCCTGGCTCCCTCCACCGCGACGTAGCGCGCGGCCAGCTACCGGAGCCGCCCTTTCATGGTCCCCGCCGTGGTCGGGACGAGATG

>SsCNGC14

GTAGAGCACGCATGCATGGCGACAAACGCCGGTTAGGTTGATTCCGTCACCAAGCACGTACGTGCGTGCGTACGCGCCGCGCGTGCACGACGCGACGAGGAGGAGGTGAGCTACGTACGCTCTAGCTAGCGGCGTATAGCTGCTTCCTGCTCGCTATATACCCGTCCATCACGCGTCGAGTCCGGCCGACGCCGTCCTCTCTGCTCCACCACCCACATGCCACGGCCTCACCACCTGGCCTGCCCGGCTGGCCACGCGTAGTCGTCGTCACTACTCACACTCAGTAGCAGCATCCACGCCATCGATCGGCCATGCCAGCCGAGCTCTCGCCGCGCCTGGCCGCGGCCACCTCATCCGCGGCCTCGTCGTCGTCGTCGCCGTCCCCTCACGGCGCGCTGCGGATCAGAGCCGGCGAGCAAGACGACAGCAGGCTGCCTCCGCCGCCACCGCCGACGGCGGCCACGGCGTTGGCAGGCAGGAAGAAGCGCCGGAGCGGGGCGTGGTGGGCGACGGAATGGGACCGCGCGTACCTGCTTGCCTGCGCGGCGGGGCTGATGGTCGACCCGCTCTTCCTGTACACCGTGTCCGTCAGCGCGCCGCTCATGTGCGTCTTCCTCGACGGCTGGTTCGCGGCCGCGGTCACCGCGCTCCGGTGCGCGGTTGACGCCATGCACGCCTCCAACCTGCTGCTGCGGCTCCGGGAAGCGTGCTCATTACCGAGGCGGGTAGACACGGACGAGGAGGAGCCGCAAGCGCAACCGGGGCGCGACGACGCACGCGGCGGCGGGGGCGGCGGCGTGCCGAAACGGGGGAGGTCCAAGAAGGGGGTTTTCTTGGACGTGCTGGTCATCCTACCGGTGATGCAGGTGCGCCGCTGGTCTCTATCTGTGCATGATCCTCGTGTCTTCTTTGAAAATTGAAATGCTAGTGCTTTCATCTGTCTCTTCTTGGGCAAAAACGATAGAAAAGGAGAAGAATTTGTTGGTGCATACATGATGAATTTAGCTGGGGCGGACTTATTGCCCGGCGACCTCAACGAACACGCAATGGAACGCAACGGAATTTTGGTGTTATCTGAAAGAGTGAAAATATTTTTTTCCCTATAACAAATACTCCGTACTCATCATAGTTAGACTTTCCGGAGCCCGCCATTTTAGTATGATTTTGCTCGGTGGACAATGTTCCCTACTCCATACTGACAGATTGGAACCCTTGCCTTGTCCAAAAGAAGATCACTGAAACAACGCAGAGTTTTGGAGCCTTCGACATTGTCATCGTCCATTAGTCCCTCATAAACCATACTCCTACCTTCCACACCGTTGCAGCATGCTGAATGCAGCATCGCATTTCCCCATCACATTCTGCCGAGCAAATGGCTGCATGTGATCCAAGACTTTACCGTTTACATCGTCCTGCTGGGGACACCAACAACACATAGCATTCAACCCATTAACATTCTCAAGAACGAAACAAAGATCACTAACGGATTACTCAGAAACCTTCCAACCGCGTTGCCTAAGATCAAGCCACAGGCCAGACTTCACATCCTGCTAAGTGCCGAGCAAATAGCTGCATGTGATCCGCCGCGCCTCCGAGCACTGGCCACGTCCCACCTCCGCCATCTTTCTGGCGACTGATGCCGCTCTGGCGCTCTGCGCCCAGTAACGTGCCGCGACCTGGGATGCCCATTATTTCCAGCGGTTTTAGAGGGTCATTTCAAGCAGAGATCTGCGCACGTACGAAAGTCGGTTTCGTTTATCGGCGTGGCGTTGCAGGATGGGACCATTGTGCTTGCGCAATTCAGTAGCCTGCAATGCATGCTGGACTATACAGATTGGGCCCGTTCATCAATCGATGTGAACCAACAATGCGCTCCATCAATCAATGCACAGATTTAAACTTCCTTCCAACGAGGCCCAAACGACCCCCACTTGGAACTGTGAACTGTGGGCGCCGGCCTCGTCATCATCTTTTCAGTGGTACTACCACGCATGTCAGCCA

>SsCNGC15

GGTTGTCCAATCAGTAGGTCGAAACCCCATATATCAAAGATATAAAAGTTCAAATGAACCATGGAGCCTTCTACCATAAAGGGTAGGACATTAATAATTCCAAGACTGGGGACTAATCGTCCCGAAGATTCCTTTATGACCTTTGTTGTGGGGGTTAAAACAAGTTTCCCAAATAAATTAAGTGCAAAAGCTTCGGACATAATATTGATCCCCACAACAGGATTATAGAGAGCGTTAAATTGATCAGAATTATAAGCACAGCGTATAGTTATAGAGGGTGTGTCCACTCGGATTACTTCAGAGGAAAGCTCTGACTCCTCTAACCATTCGCTACTCATAACCGAGATAAGCTCTCTCAATTGACGTTCACTTGGTAAACAAATGCTAAATTGGCCGTTTTGGGGTCGGTCTATAGAATGGTAGTTTGAAATGTTTCCAAAATCGGCAAAAGATCGGATTCTATATAGCATGAAATCAGATGGTGAATTTCTTCCTTTTGTGGCTCAAACTGGGATAGCTAAAGTAGATGAAAATTTGGCAGAGTGTCTACTTCGGCTTCGTAGGTTTTATCATGGAGGGCATTATCCATCTCAGCTTCTAGGATTCTATTCAAGATCCTTAGCTTCATCAGTAGGGATACGAAAGAAAGAACCTCTAGACATGTATGTAGCATTTGTTTATTATCTTTCTGAAGACCTCGAAAAAATGAAAAAAAGAATAGGTCTTCAAAAGTTTTGGACCAGATTCTAAAAGATCAGAAAAACGTTTCCAGGATTTCCCCAAAGTTTCATTATCTTTTTGTTTAAAAGATAGGACTTCGAGTCTAAGGTCACCAGTACGGTCAAGGGAATAAAAATCTAGACAAAAGTTGGCTCTTAAAACTCCCCATTCACCTTGCTGTTGACTTACCTTCTGACTATACCATCGTCTAGCTTCTCCTCTTAAGAAAAAGGAAAAAGCTTCCAACGTAAAGTCTTATCAGAGATGCCATCAATGCGAAGACAATCGCATGTCTGCTCAAAATCTCTAATATGAAGGTAAGGGTTTTCGTCTTCCTTTCCCGAAAAAGATAAATTTTGAATCATCGCAATCAACCTTGAACTTAACTTATACTGGGATGTTTGGATAGGCTGTGATGACTCCCATGGTAAAAGTTCAGTTTCCGAAGGTGTTGCAAATTGATAGATGATAGAATCTTGGTTCTCCATGGTAAGAAAAAGAAAAGATAAGGAAAAAATAAAGAAAGATAAAAGTATAGGTACAAGCTAGTAGTAAGATTCAAGGTTATCTCAGCAAACCGTCTTTCTCCCCGGCAACGGCGCCAGAAATGCTTGTTGATATTTGGTAACGCAATTAGGAAATAGGATCCGCANNNNNNNNNNNNNNNNNNNNNNNNNNNNNNNNNNNNNNNNNNNNNNNNNNNNNNNNNNNNNNNNNNNNNNNNNNNNNNNNNNNNNNNNNNNNNNNNNNNNGTCTTCTCTCGCTTCACCCTATACTGACTGATGTGTCCATCAAAGCATATTGCCCCCTCGAGGACGGTGAATGGAACGAAGTCAATAGCATCCTAGCTACTCCTTGCCCAACCACCGCATCCTCCAAGACTAACAAGGCAAATGTGCCCAGGCTTACCACTGATGTGTCCATCAAAGCATATTGTCGTGAATGCGCGGGCATCTTACCACTAATGGCTTCTCGGCGAAAGACGACGCCTGCGATGCCATGATTCTGTGACAGCTAGACATGCCATTCTTGGTTGCAGGCGGTGGGCGCGTGCTGGTACCTGCTCGGCGCGCAGCGGGCCACCAAGTGCCTCAGGGAGCAGTGCGCCCAGGCCGGAGCGGGTGCGCGCCCTGGGCGCTGGCGTGCGCGGAGCCGCTCTACTACGGGCGCAGCGTGAACGTCGGGGCTGACAGGCTCGCCTGGGCCGGCAACGCCACGGCCAGGGGCACGTGCCTCGACAGCGCCGACAACTACCAGTACGGGCCTACCAGTGGACGGTCA

>SsCNGC16

CTTCCCGGTTGAACTGCCGGTTCCCTTGGCGGCCTAGTGCTTGCGATCCAGCGACGCGACGCCAAGTCGCCAGACGCCGGCGAGGTAGCAACGAGACACAGGTTCAAAGGGAAAAGACAATGCCAAGCCTAAATATGTGGTCAGTGAAATTTAGAGATCTTCGTTGAGTTTAGAGTTTTTTTTTTGTTACAAGAAAACCAGGAATAATTATGTAGCTGAAGAATAATTCAACAGGACATCCACATGACCACATATAGTTTGAGATTGCCAATCATGCATGGTCTATAATCATGCATTGTACTGTACGTGCTAACGTGCGAGACTGGTGTCGCCCAAATACTAAGGTCTTGTTTAGATGCACCTCAAAAGTTAGATTTTTTTCACTCTTTCTCCATCACATCAATATTTGGACCCATGCATGAAGTAGTAAATGTAGGTAAAAAATAACTAATTACACAGTTTAGTTGGAAAATCACGAGATGAATCTTTTGAGCCTAGTTAGTCCACGAGTGGACAATATTTGCCAAATAAGACGAAAGTGGTACTATTCATCAGGTTCAAATTATTTGCAATCTAAACGTGGCCTAAGTTGCACAGGGCACAGTTTGGAGTTTTCCTAATTGCATTACCCTGCCCAAACTGTTCTTGCCAGGAACATTATTCCTTCTAACAACTGCGTCCTGCCATGAACCGATGATCGATCTCATGTCGCTACCACTGCACTGGCCGGTAGACCGGCACGGCGCGACGCGATCGGCCGCACGGCACAGAGCGGTGACACGAGTGGGGCTTGCGTGTCATATATAGTTTCTGCTTGTCCTGACGCACAATCAAATATACCTGTATTAAAAGTTAAGGATACCGTACAAAGACATGTACACATACATACAGGTACATTCGAGAAGGATACAGAAGGCAGCTGCATCGACCGTTTTACAGAGGCCCCTATAAGGAACAAAGAAAAATACAAATGAAGCCCTAAATCCTCGGTCGAATCCTCCAAACTCCGCCGCCGCCGGCCGCCGCCGGAGATCCTTCGCCGTGAACCCCAGGTCAGAAGAAGCCCTATTGCCTCAAGGCTTTGGAAGTAGCCGCACTATACACTCGCACCGAAGAGCAAGATGAAGCAAACGCTGAAAAAACATTGGCCGGGGACGCACCCCGAGCTTCCTCGACCATGGACAGGGACTCGCCATCGACGACCGAAGCCGCCGCAGAAAACTCGAGCCTTATCCACTCTACAACCATCCACATGACCCAAAGCTGCCTCTTTATCCTCAGACCAAGTGGCTAGAAGACGCACCTCAAGACGATGCACCGCCAGCCGAAGACTCCTAAGTAGATCTCCACGCCCTCTCCGTTGATGCCAGAACGGAGCTCACCAGGGCGAGGAAGAGGCCAGAAGAGCTTATTCCCCTACGCCATCGCTGCCTCAGCCTCGCCGACGCCGCCAAGAAAAGCAAGCCACCGAGGATTGACAACCCCCGGCAAACTCTAGCTTCACGGACAATAAACGACGGCGAGCAAAACCTAGGGCTAGTTAGGTAGCTAGCTAACCTATGTACATCCACACGCCGATTCCCCGGTCCACCTCCATCTCCAGCGCCTAACCGGCCGCCGGAGAGGGAAAGGGCCGGTAGAATTGCTGCCGGAAACTCTCGTCGCCCTCTTTTCGCCGCATCTGACCCTGTCCGTGGCAGCGTGGCCCCGAATGCTCGTGCCGCGCGCAGCCATTGGCACGTACGCAGGCCCGTGTCCATGCGCATCGTCCGGTCAGATAGGGCCACGCCACGGTCCACGGCCTCTTCATCTTGCCACCAACCCGGCCGGGCACTGTCCACCACACCGCCATGGATTTTGTTACTCGTTGGTAGATTCAGTGAAAGCGTGTCGTCTGTTCCGGACTTCCGGTGTCTGCCGGCCTGTTTGGAGTTTGGTCCTGACAGTGTATATGGCGCATGCATGCATTGCAGGTGGTGGTGTGGGTTGCGGCGCCGGCGA

>SsCNGC16-2B

AATTGTTCCCTTTGTTGTTTTTATTGGTTGCTGATGTTGTATTCGCGATGCGATAATTGAAACTTTTGTATAGCCCTACTTCCGGTGCTTTTTTTTCCAATGGATCGATGGCCCGGACAAGTTTGATCCAAGGATTCTCCTTTTCTACCCCGGCATTGATCATTGCAAGCGTGAGGTGTTTACTCGCTGGGTTCCGCCCCCTCCAAACCCTCCTCCTATGACGGACGAAGAGAAGGCCGTAGCATCGGCACGGCGGCTCGAGGATCCTCCAAAGTGCCTTTGCGGAGAGCAAGCCGTGATAAACCCACGCAATGAACAAGAGTTCATTTGTCCTCTACGACATGAGGTAAGGTTTGAGTGTACCTTGGAATGTCACGTTCTATATGTGTGTTTTACTGTTTTGATTTTGTAGGATCATGATATGCCAAAGTGCCGTTTCAAGGAATGGATCTATGGTCCAAAGAGCCATTGGCCGGAGCCGGAGAAGAATGTGGAGGTGCCAGACTGGAAGAAGAAGAGGAGGTCTATTGCCCCTCCTGTGATGTGCAAATGTGGTGTTGAAGCTAGCTACGGCCTTGTCCCTTCAGGCCTTGGTATAGGGCACTTCTGTGGCCACATGATCGATTATGACGAGGTTTGCTAATACTGTTGACAGTTGGCGGTAATGGTTTATTTTGTTAACTCTGGTACGCTAACTTTGTATTCATCTATTTCTTGCAACAGAGCACTCAGAAATGCAAGTGGGAATCATATTATGATGTGTATAATTTCAAGAATGAGTATAAGACAATGGTAGCAGTTCGCAATAGCAGAGGATATCCTGCAAGCTACGTCACAGATTTCGTTAAGAGCCACAAGAAGAAAATGCTACGACGGGCCCAAGAGCTACGTGTTCGTAACCCGGAGAGTATTGCGTGGAAGAAGTGGTACGAGCAAAAGAAGAAGGATGTAGAGGAGTACCAGGCACGGAAGGCTGAAGAGGATGCAAGGAAGGCTGCAGAGGAGGCTGCGAGAGCAGAGATGCAAGGCTTGAACGATACTATTGACTCATTATGTGCGAGTGAGTCATTGCGTTTTTGGAAAGTCAAACTTGTTTGTTTTACTGTCAATTAACTCATTGCAAAGATCTAGTTAATTGTTTGCATTTTGTACTTGCAGAGATTGGATGCACCGGCGGCTGGGAAGCCGATATGGCTCGTGCTAGGTACACCGCAAGTCGTCCGATTGTGGTTGACGAGGAGGCCGAGCCGGAGGAAGACGACGACACCGGGAGGATAGGCGAGCTCATTCGTCTAGCAGAACAATTGGGCTACCAGGAGGATGTTGCAGTACACGAGGCCCAGGCAGCGTACCAGAGCCAACAGACACCGTTGTTCGACTCATGGGGGCCAACAGACATGACCCAAGAAGAGGCCGAGTTGTACTCTCAGGCCGCGGACGAGGCGGAGGCGGCTTACTACCGGCGTCAGGCCTCTGAAGCCAAGTCAGTCGAGGCCAGCAAGGGTAAGGAGGTCGTAGTGGCGGGTTTGGCATACCCCGCCATGATCCGTGGCGGGTTTGGCATACCCCGCCACCCATCATGGCGGGGTACGCCCCGGCATCCCGCCACGTCACCGTGCCACGTCACCGTGCACCCCGCCACCATGCATGGCGGGGTACAGTGCATTTGCCCCGCCATGCATGGTGGCGGGGCCAAACGGGTTAGATTTAAAATTTTTTTGAAAGACGGGTTAAATTTAAAATTTGTTTCAAATTTGGGCTAAAAATAAAAAAAAATTCTCCCTTGGCGGCCTAGTGCTTGCGATCCAGCGACGCGACGCCAAGTCGCCAGACGCCGGCGAGGTAGCAACGAGACACAGGTTGAAAGGGAAAAGACAATGCCAAGCCTAAATTGAGAGGTATATACTGGCATTCGCATCAGTTCATGTTGTTTCACACCGTCAATTTGACGAATCACTCGTCCTTCCTCCGCTGTTGGGCTCAGTTGTATCGCCATCGCCA

>SsCNGC16-3C

TTAGCTAAAACAGCTTCTGCTTCATCGGAAGCTATTCTATGGCATATTAGTATATTATAAATATTCTTTCTATTCTAAACTATAAATTGGTCAGGTTTTGTAAATATATAATTTTCATGATATATCTTGATATAACAAATATCTGGGTGCATAGCAAAAGCTACTCCCTCCATCCCAAATTATAAAACGTTTGACTTTTTTATCTCAAGTTTGACCACTCGTCTTATTCAAAAAATTTGTACAAACATAGTCAAATTTAAGTCATTCTTGAGGAACTTTTATTAATATACCAAGTCACGATAAAAGAATTGATATTTTGCACAAATTTTTGAATAAGACGAGTGGTCAAACTTGGTGTAAAAAAAGTCAAACGTCTTATAATTTGGGATGTATGGAGTATGTAACTAGAAATGCTAGAACAATTTATAATTTTGAAGGATTAAGTAATGGATTTAAGAAAAAAATAATACTATCTTAATACTTATTTTTTCATTTCAATTTAGTATAACTTATGTAATGCAATCAACCAAATAAACGCTTTACTCACTTGTAAAGAAACAACAAGCTAATGAATGTCTTCAACCTATCCCTGCACAGTCAGTATATGCGAGTCAAACTCCCTAGGCAGTGAATCAGACACGCTTGTTTGGTTTACGCTCTGAGAAAATCATCTGACCGAGACGAAGGCCTGGTTGGTTCCACCCCTAAAGTTTAGTGGGCATTAAAGTTTAGTGATGTTTCTAAAGTTTAGTGACTCCATGTTTGATTATAGTGACTAAAGACTTCTAAATCAGCTGTTATATGTATATTTTTGTCACTTTTAGCTACTCTTATGACAAAAGCCACTAACTTTAGTGGGGGTGTTTGGCATTTTACCCCTCCTAAAGTTTAGTAGTCACTAAAATGCCAAGCACCTCCACTAGTCATAAAAATGTCAAACACCCCACTAAAGTTTAGTAACCAAGGGGTAACTAAAAGTAACTAAAATGTACGTGAGACAGCTGTTTTAGTGGGTGAAACTAAACATGAGCCACTAAACTTTAGTGGTCACTGACTTTAGTGGGTGACTGAAGTTTAGTCTAACCTCTAGGAGGAGTGAACCCAACGTGGCCTAATAAAAGGCTAAAACGGCCGATTTTGAGTGCCTGTAGGCTGTAGTTGGATGAAACTACCTGAAAGTCAGAAACCATTAAGGTAGTGTTTAGATGGTGAAGTTGGTGTAAAGTTTCTATAGCACTTTTCGTTGTTATTTGGTAAAATTTATTCAATCATGGACTAACTAGGTTCAAAAGATTCGTCTCGACATTTACAGACAAACTGTGCAATTAGTTATTTTTTTACCTATATTTAATACTCCATACATGCGTCTAAAAATTCGATGTGACAGGGAATCTCGTAAAATTTTTAGAATTTTGAAGGAAGTAAACACTGCCTAAGGCCTCCAATCAATCAAACATCTCCTATATAGTTGGATGATGTGCCCAACCAAACATTTGCTGAGTGGCGAAACGCGCTTAGCTTCCACTGCTCCACAGGCTCATATCCTCTCCTGTTGCGGCATCCACGGCCAACTCAGCGCAATCAAGGGCAGATGATCTGTGTATAGCGCTCGCCATCTCTTGATTTTCCCATTGGTCCCCTGAATTCGCTGACCAACAGTCCTGTCCGCCATGGACGCCGAAAGAAGCTCTGCACGCAGCACCACGCCTTTTGGTCTGCCGTCCGCTCGCCACTGCTAGATGCGACGACGTCTGCGCTGCCTTCCCGGTTGAACTGCCGGTTCCCTTGGCGGCCTAGTGCTTGCGATCCAGCGACGCGACGCCAAGTCGCCAGACGCCGGCGAGGTAGCAACGAGACACAGGTTGAAAGGGAAAAGACAATGCCAAGCCTAAATTGAGAGGTATATACTGGCATTCGCATCAGTTCATGTTGTTTCACACCGTCAATTTGACGAATCACTCGTCCTTCCTCCGCTGTTGGGCTCAGTTGTATCGCCATCGC

>SsCNGC16-4D

AAAAACTTAGTTTTTTTTTGCTAGAGGTGTAACTAAAGACAACAAAAACTATATCAAATAAAACACGGTCACTAACCTATAGGATCGGCCAGTACGGGAGGCTAATCTCATCCACCCCCACCCCTCACCCCTACTATACCTGAGGGTCATCCCCCTTCTAAAAGTTTTAGGAGTTTTCCTTCATAAAATCATGCATGTTTCGGAGGACCACATGTAATCCATGTGATATGCAGTTTTGCCTGGTTTTTCTTAGAAACGGGCTTTTGCTTAGAAACGGTTATCAAAGAAAATATGGTCACTAACCTATAGGATCGGCCAGTACGGGAGGCTAACCTCATCTAGTCGAACCCCTCACCCCTACTATACCTAAGGGTCATCCCCCTCCCAAAAGTTCTAGGAGTTTTCCATTACAAAATCATGCATGTTTTGGAGGACCACATGTAATCCATGTGATATGCAGTTTTGCTAGGGTTTTCTTAGAAACGGCAGTTTTGCTTTGGAACGGCCGTTTAAATGTTTGCAAAAAAGTTATCGAAATATATCCATGTGGGGTCTTGTTTTGAAGAGTTTATTGCGAAGAACATGGTGCAATTGGATTTTAATTACGATACTTGGTTTAGGTGCTACGACTTTTTTTTCTATTTCAAAAGAAATAGTTAGTGAGAGATAAATGAGACTTAGTGGGGGTAACTGATTTGATGACGTCACATGACATCACTTTTCAAGATTGGACCAATCAAATTGCATAGAATCACTTAGTGGGTCTTAGCTTTATAGGAGGTTTAGATATGTATACTTTAGTCACTTTTAGCTACTCCTTTATGACTAAAAGCCACTAAGCTTTAGTGGGGTGTTTGGCATTTTACCCCTCCAAGAGTTTAGTAGTCACTAAAATACCAAACACCCCCGCTAGTCACAAAAATATCAAACACTCCACTAAAGTTTAGTAACTAAGGGGTAGCTAAAAGTGACTAAAATATATATGAGACATCTGCTTTAGTGAGTGAAACTAAACAAAAGTCACTAAACTTTAGCGGTCACTGGTTATAGTGTGGGTGGAACCAAACAGACCAAGTAACTAAAAGTGACTGATAACCTCTAACCTCCAGGAGGGGTGAACCAAACGTGGCTAAAACGGCCGATTTTGAGTGCCTGTAGGCTGTAGTTGGATGAAACTATCAGAAAGTCAGAAACCATTAAGGCCTCCAATCAATCAAACATCTCCTATATAGTTGGATGATGTGCCCAACCAAACATTTGCTGAGTGGCGAAACGCGCTTAGCTTCCACTGCTCCACAGGCTCATATCCTCTCCTGTTGCGGCATCCACGGCCAACTCAGCGCAATCAAGGGCAGATGATCTGTGTATAGCGCTCGCCATCTCTTGATTTTCCCATTGGTCCCCTGAATTCGCTGACCAACAGTCCTGTCCGCCATGGACGCCGAAAGAAGCTCTGCACGCAGCACCACGCCTTTTGGTCTGCCGTCCGCTCGCCACTGCTACTTCCTACGTTCCAAATTATAAGACGTTTGACTTTTTTTTTTGACCTCAAGTTTGACCATTCGTCTTATTCAAAAAATTTGAACAAATATAGTCAAATTTAAGTCATTCTTAAAGAACTATCATTAATAAAGCAAGCCACAACAAAAGAAGTGATGTTTTGTAAAATTTTTTGAATAAGACGAGTGGTCAAACTTGGTGTAAAAAAGTCAAACGTCTTGTAATTTGAGATGATAGATGCGACGACGTCTGCGCTGCCTTCCCGGTTGAACTGCCGGTTCCCTTGGCGGCCTAGTGCTTGCGATCCAGCGACTCGACGCCAAGTCGCCAGACGCCGGCGAGGTAGCAACGAGACACAGGTTGAAAGGGAAAAGACAATGCCAAGCCTAAATTGAGAGGTATATACTGGCATTCGCATCAGTTCATGTTGTTTCACACCGTCAATTTGACGAATCACTCGTCCTTCCTCCGCTGTTGGGCTCAGTTGTATCGCCATCGCCA
